# Synthesis of Biaryls via Decarbonylative Palladium-Catalyzed Suzuki-Miyaura Cross-Coupling of Carboxylic Acids

**DOI:** 10.1016/j.isci.2019.08.021

**Published:** 2019-08-17

**Authors:** Chengwei Liu, Chong-Lei Ji, Zhi-Xin Qin, Xin Hong, Michal Szostak

**Affiliations:** 1 College of Chemistry and Chemical Engineering and Key Laboratory of Auxiliary Chemistry and Technology for Chemical Industry, Ministry of Education, Shaanxi University of Science and Technology, Xi'an 710021, China; 2 Department of Chemistry, Rutgers University, 73 Warren Street, Newark, NJ 07102, USA; 3 Department of Chemistry, Zhejiang University, Hangzhou 310027, China

**Keywords:** Chemistry, Catalysis, Organic Chemistry

## Abstract

The biaryl motif is a building block in many drugs, agrochemicals, and materials, and as such it is highly desirable as a synthesis target. The state-of-the-art process for biaryl synthesis from ubiquitous carboxylic acids is decarboxylative cross-coupling involving loss of carbon dioxide (CO_2_). However, the scope of these methods is severely limited, mainly due to specific substitution required to promote decarboxylation. The present report implements a decarbonylative version with loss of carbon monoxide (CO) that enables to directly engage carboxylic acids in a Suzuki-Miyaura cross-coupling to produce biaryls as a general method with high cross-coupling selectivity using a well-defined Pd(0)/(II) catalytic cycle. This protocol shows a remarkably broad scope (>80 examples) and is performed in the absence of exogenous inorganic bases. In a broader context, the approach shows promise for routine applications in the synthesis of biaryls by carefully controlled decarbonylation of prevalent carboxylic acids.

## Highlights

• First decarbonylative Suzuki cross-coupling of carboxylic acids via Pd catalysis
• Rapid synthesis of functionalized biaryls from ubiquitous carboxylic acids
• Mechanistic insights from DFT studies point at the origin of high selectivity
• CO loss as a strategy for expanding access to aryl metals (cf. CO_2_ loss)

## Graphical Abstract

**Figure:**
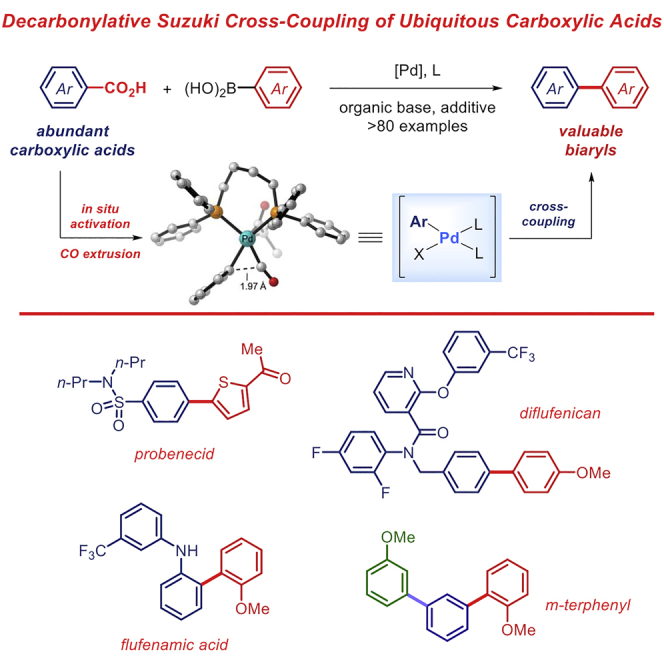

## Introduction

**Figure 1:**
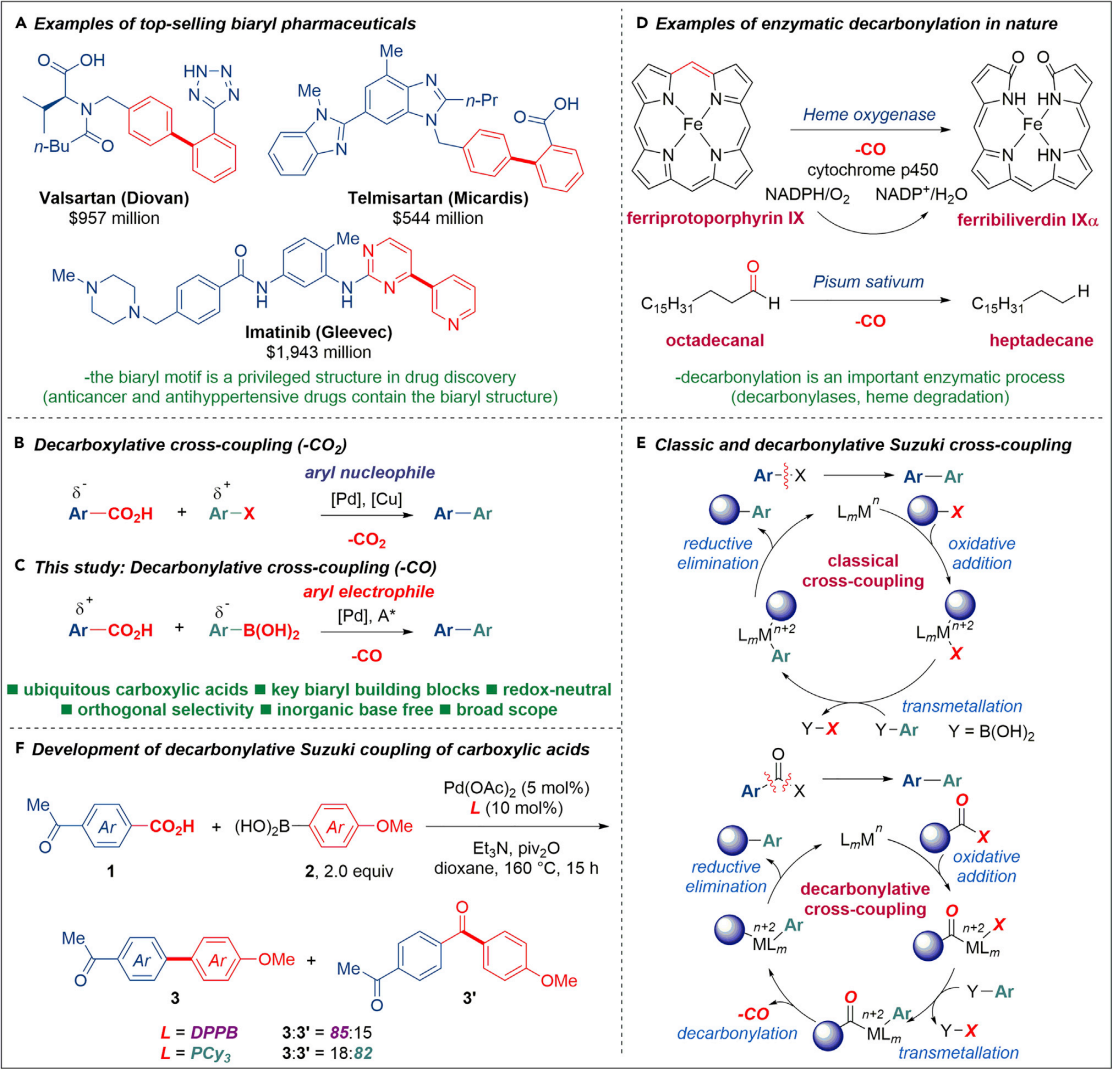
Background and Reaction Development (A) Examples of top-selling pharmaceuticals containing the biaryl structure. (B) Enzymatic decarbonylation in nature. (C) Decarboxylative cross-coupling of carboxylic acids (loss of CO_2_): current state of the art. (D) Proposed decarbonylative cross-coupling of carboxylic acids (loss of CO). (E) Mechanism of the classic and decarbonylative Suzuki cross-coupling. (F) Development of decarbonylative Suzuki cross-coupling. Dppb, 1,4-bis(diphenylphosphino)butane; PCy_3_, tricyclohexylphosphine; piv, pivaloyl.

The biaryl motif is a privileged subunit in chemical science (Hassan et al., 2002, Horton et al., 2003, Burke and Marques, 2015). The importance of biaryls is highlighted by the wide presence in pharmaceuticals, functional materials, and natural products in both industrial and academic research (Brown and Boström, 2016, Yet, 2018). The biaryl architecture is at the heart of widely prescribed antihypertensive and anticancer agents, which, in addition to the huge economic benefit, save the lives of millions of patients annually (Figure 1A) (Urquahart, 2018). The tremendous success of the conventional Suzuki-Miyaura cross-coupling of aryl halides has provided multiple avenues to generate biaryl architectures of key significance to the chemical industry (Miyaura and Suzuki, 1995, Lennox and Lloyd-Jones, 2014, Molander et al., 2013, Colacot, 2015). Since the 2010 Nobel Prize in Chemistry (Suzuki, 2011), more than 12,000 publications address the improvements to the conventional Suzuki-Miyaura cross-coupling, signifying the great advantage of implementing this transformation (Scifinder, 2019). Although effective, the conventional Suzuki-Miyaura cross-coupling of aryl halides suffers from major limitations, including (1) the use of less available aryl halides, (2) the requirement for stoichiometric inorganic base to trigger transmetallation, and (3) generation of toxic halide waste.

The major breakthrough in using ubiquitous carboxylic acids as substrates for the synthesis of biaryls was achieved in 2006 involving the extrusion of carbon dioxide (–CO_2_, Figure 1B; Gooßen et al., 2006). In this carefully engineered design, the use of a copper(I) co-catalyst lowers the decarboxylation barrier and delivers aryl nucleophiles to [Ar–Pd–X] intermediates (X = Cl, Br). Despite severe limitations mainly with respect to the reaction scope, this seminal report has sparked new interest in decarboxylative cross-couplings of ubiquitous carboxylic acids as advantageous substrates in homogeneous catalysis (Gooßen et al., 2008, Dzik et al., 2012). Recent years have witnessed the development of unconventional precursors for the biaryl synthesis, including aryl ethers (Tobisu et al., 2008), acetates (Guan et al., 2008), pivalates (Quasdorf et al., 2008), carbamates (Quasdorf et al., 2011), sulfamates (Quasdorf et al., 2011), and ammonium salts (Blakey and MacMillan, 2003, Tasker et al., 2014). Further progress has been realized in using aroyl precursors, including anhydrides (Gooßen and Paetzold, 2004), esters (Muto et al., 2015), amides (Shi et al., 2016, Ji and Hong, 2017), and acyl fluorides (Malapit et al., 2018) under Rh and Ni catalysis. In an alternative direction, the combined use of photocatalysis and Ni catalysis has effectively addressed the limitation of cross-coupling of C(sp^3^) centers (Tellis et al., 2014, Zuo et al., 2014), whereas fundamental studies on ligand design have tackled the challenge of enantiodivergent (Zhao et al., 2018) and conjunctive (Zhang et al., 2016) Pd-catalyzed Suzuki cross-coupling. However, none of these methods have the key advantage of directly engaging the pervasive carboxylic acid functional group in the Suzuki-Miyaura cross-coupling to generate highly useful biaryls.

This report implements a decarbonylative version of Suzuki-Miyaura cross-coupling with loss of carbon monoxide that enables to directly engage carboxylic acids in a redox-neutral pathway to generate biaryls with high selectivity using a well-defined Pd(0)/(II) catalytic cycle (–CO, Figure 1C) (Zhao and Szostak, 2019). As (1) significantly more carboxylic acids than aryl halides are commercially available and (2) carboxylic acids form an intrinsic part of advanced bioactive products and functional materials, undoubtedly the direct Suzuki-Miyaura cross-coupling of carboxylic acids as electrophilic components represents a modular approach to precisely construct biaryl building blocks. Furthermore, the orthogonal properties of carboxylic acids and the exploitation of carbon monoxide loss (CO versus CO_2_, carbon dioxide) offer unique opportunities for catalysis. The C–C bond formation by cross-coupling of boronic acids is a fundamental reaction in organic synthesis that has found widespread application in various areas of chemistry. This report demonstrates the first example of a general utilization of ubiquitous carboxylic acids in the Suzuki cross-coupling for the synthesis of biaryls.

## Results and Discussion

**Figure 2:**
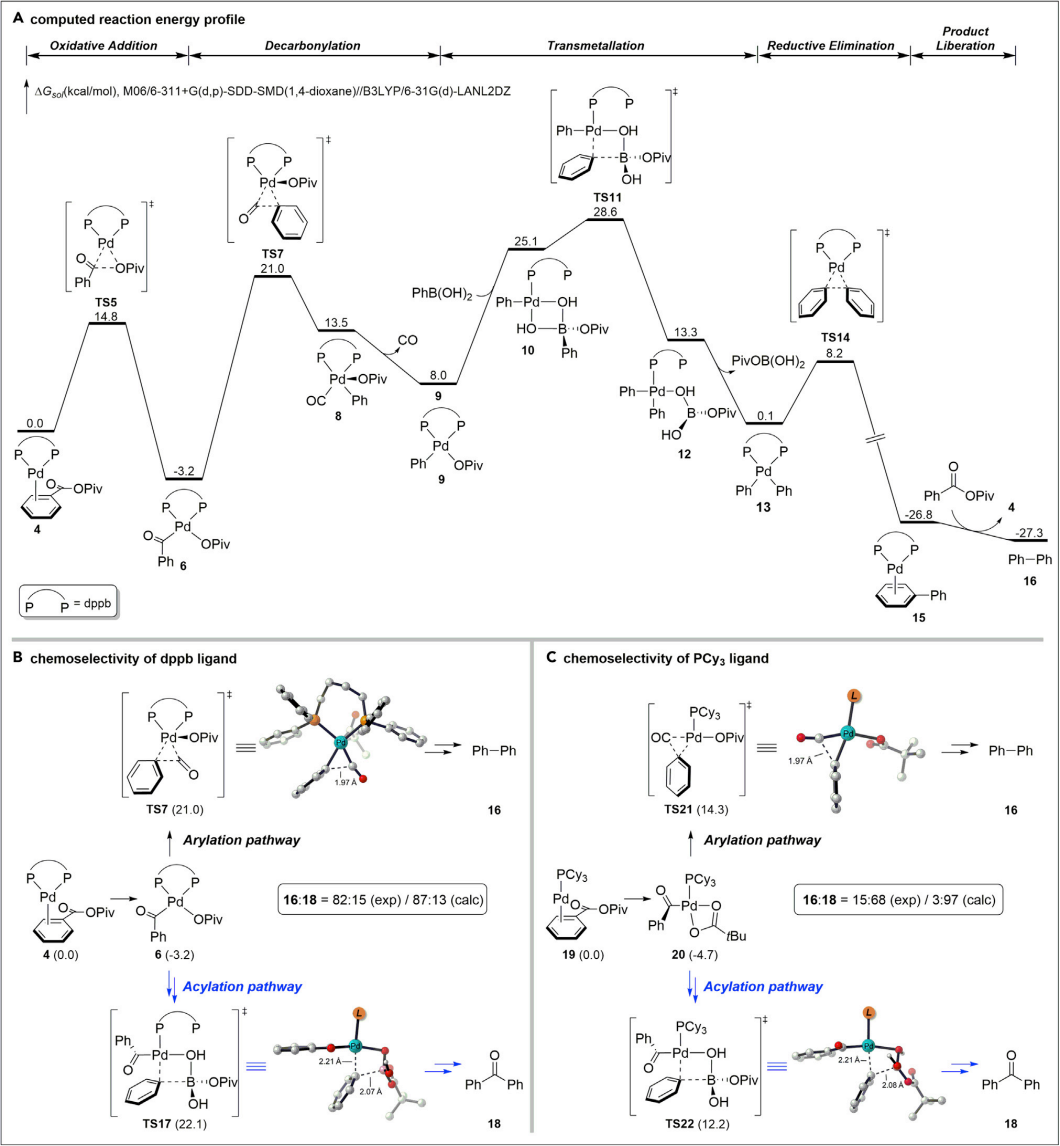
DFT-Calculated Reaction Energy Profile and Chemoselectivities of Pd-Catalyzed Decarbonylative Suzuki-Miyaura Cross-Coupling of Benzoic Pivalic Anhydride (A) Computed reaction energy profile. (B) Chemoselectivity of dppb ligand. (C) Chemoselectivity of PCy__3__ ligand. All energies are in kcal/mol. Hydrogens are omitted for clarity in the transition state diagrams. DFT calculation details are provided in Transparent Methods in the Supplemental Information.

We anticipated that carboxylic acids can be galvanized into the decarbonylative (Murphy et al., 2015, Ryter and Tyrrell, 2000, Cheesbrough and Kolattukudy, 1984) Suzuki-Miyaura manifold (Figures 1D and 1E) through *in situ* activation, a process that is reminiscent of the classical activation of carboxylic acid derivatives in organic synthesis and has been utilized to great effect in decarboxylative cross-couplings of C(sp^3^) electrophiles (Qin et al., 2016, Edwards et al., 2017, Fawcett et al., 2017). We targeted Pd catalysis and *in situ* activation as two key design elements to execute high catalytic efficiency, modularity, and practical significance. Studies showed that oxidative addition of a C–O bond of anhydrides occurs with high selectivity (Gooßen et al., 2008, Dzik et al., 2012); however, unselective decarbonylation and transmetallation lead to ketone products. Given this challenge, we hypothesized that a union of a sterically hindered O-acyl group and a bidentate ligand would favor decarbonylation (*vide infra*, **TS7**, Figure 2B), providing a simple and practical access to biaryls directly from carboxylic acids. Extensive optimization identified two catalytic systems that led to vastly different outcomes in the cross-coupling of 4-acetyl-benzoic acid with 4-MeO(C_6_H_4_)-B(OH)_2_ (2.0 equiv.) as the model reaction (Figure 1F and Supplemental Information): (1) Pd(OAc)_2_ (5 mol %)/1,4-bis(diphenylphosphino)butane [dppb] (10 mol %), piv_2_O (2.0 equiv.), Et_3_N (2.0 equiv.), dioxane, 160°C: biaryl: ketone = 85:15 selectivity (82% yield of the biaryl); (2) Pd(OAc)_2_ (5 mol %)/PCy_3_ (10 mol %), piv_2_O (2.0 equiv.), Et_3_N (2.0 equiv.), dioxane, 160°C: biaryl: ketone = 18:82 selectivity (68% yield of the ketone). Selected key optimization results are presented in Table 1. It is noteworthy that an inorganic base is not required (entries 3–6), establishing a practical parallel to the Ni(0)-catalyzed method (Malapit et al., 2018) and that there is a good correlation between the efficiency and the ligand bite angle (entries 9–15) (Miyaura and Suzuki, 1995, Lennox and Lloyd-Jones, 2014). Note that the absence of piv_2_O resulted in no reaction, in agreement with our design (not shown).

**Table 1:** Summary of Optimization and Control Reaction Conditions


Entry	Variation from Standard Conditions	Yield (%)^a^^,^^b^
1	No change	82 (15)
2	No H_3_BO_3_	49 (9)
3	Na_2_CO_3_ instead of Et_3_N	52 (15)
4	K_2_CO_3_ instead of Et_3_N	51 (23)
5	Added Na_2_CO_3_	80 (12)
6	Added K_2_CO_3_	71 (13)
7	Pyridine instead of Et_3_N	43 (6)
8	DMAP instead of Et_3_N	43 (<2)
9	PPh_3_ instead of dppb	24 (61)
10	PCy_3_HBF_4_ instead of dppb	15 (68)
11	DavePhos instead of dppb	<2 (<2)
12	dppp instead of dppb	<10 (<2)
13	dpppe instead of dppb	44 (21)
14	BINAP instead of dppb	27 (27)
15	XantPhos instead of dppb	26 (3)

Standard conditions: Carboxylic acid (1.0 equiv.), Ar–B(OH)_2_ (2.0 equiv.), Pd(OAc)_2_ (5 mol %), dppb (10 mol %), Et_3_N (2.0 equiv.), piv_2_O (2.0 equiv.), H_3_BO_3_ (2.0 equiv.), dioxane, 160°C, 15 h.

GC< gas chromatography; NMR, nuclear magnetic resonance; dppb, 1,4-bis(diphenylphosphino)butane; piv, pivaloyl; Et_3_N, triethylamine; DMAP, 4-dimethylaminopyridine; dppp, 1,3-bis(diphenylphosphino)propane; dppe, 1,2-bis(diphenylphosphino)ethane; BINAP, 2,2′-bis(diphenylphosphino)-1,1′-binaphthalene.

a Determined by GC/^1^H NMR.

b Yields of the ketone product are shown in parentheses. See Table S2 in Supplemental Information for details.

At this point, extensive density functional theory (DFT) studies were conducted to provide insight into the origin of the reaction selectivity and determine the reaction pathway (Figure 2, for the Cartesian coordinates, see Data S1, related to Figure 2). Note that the reaction is efficient in the absence of an inorganic base (Lennox and Lloyd-Jones, 2013, Malapit et al., 2018), which implies generation of the transmetallation-active [Ar–Pd–X] intermediate that could directly engage in transmetallation with a boronic acid under functional-group-tolerant inorganic-base-free conditions. The computed reaction energy profile with Pd/dppb catalyst is shown in Figure 2A (see the Supplemental Information for DFT calculation details). From the substrate-coordinated complex **4**, the acyl C–O bond cleavage via **TS5** generates the LPd(acyl) (OPiv) intermediate **6**. This acylpalladium intermediate undergoes decarbonylation through **TS7**, and subsequent CO extrusion leads to the arylpalladium species **9**. From **9**, the boronic acid coordinates to allow the transmetallation via **TS11**, leading to intermediate **12**. In **TS11**, the pivalic leaving group acts as an intramolecular base, which transfers the boronic acid to the corresponding boronate and promotes the transmetallation process. This suggests that the overall transformation does not require an external base, which is consistent with the experimental conditions. Therefore, the design of anhydride not only controls the desired C–O bond activation but also plays a critical role in the base-free transmetallation. After the transmetallation, **12** dissociates PivOB(OH)_2_ to form intermediate **13**, which undergoes C–C reductive elimination through **TS14** to generate the product-coordinated complex **15**. Final product liberation of **15** produces the biaryl cross-coupling product and regenerates the active palladium catalyst. Based on the computed free energy profile, the acylpalladium species **6** is the on-cycle resting state, and the transmetallation step via **TS11** is the rate-limiting step with a 31.8 kcal/mol overall barrier (**6** to **TS11**).

The mechanistic model provides a rationale for the ligand-controlled chemoselectivity of competing arylation and acylation. The computed chemoselectivities of dppb ligand are included in Figure 2B. From the acylpalladium intermediate **6**, decarbonylation and transmetallation determines the chemoselectivity (**TS7** versus **TS17**) if the CO extrusion is considered irreversible from a reaction kinetics perspective. Our computations indicate that **TS7** is 1.1 kcal/mol more favorable than **TS17**, which agrees well with the experimental observations that dppb ligand leads to arylation product. In contrast, for PCy_3_ ligand, the acylation pathway is more favorable by 2.1 kcal/mol (**TS21** versus **TS22**, Figure 2C). The detailed free energy profile of PCy_3_ ligand is included in the Supporting Information. This reversed selectivity is due to the denticity change of the ligands. Bidentate dppb ligand favors the decarbonylation step, because **TS7** has two phosphine coordinations, whereas **TS17** only has one phosphine coordination. This change of ligation does not exist for monodentate PCy_3_ ligand because both transition states **TS21** and **TS22** have one phosphine coordination, which is why the chemoselectivity is reversed. Therefore, the ligand denticity is a useful approach to control the chemoselectivity in the Pd-catalyzed Suzuki-Miyaura cross-coupling of carboxylic acids (see the Supplemental Information for additional studies on the mechanism).

Synthetically, the key advantage of this approach is that carboxylic acids are directly engaged in the synthesis of biaryls without separate preactivation steps. The released by-products in the process are CO and a mild organic acid pivOH (p*K*_a_ = 5.0), which alleviate the potential side reactions, while at the same time this approach obviates toxic and more expensive activating reagents (e.g., TFFH [tetramethyl fluoroformamidinium hexafluorophosphate]) (Malapit et al., 2018) and, importantly, is performed on the benchtop using commercially available, air- and moisture-stable reagents, which supersedes previous methods using air-sensitive Ni(0). This results in a broadly applicable gateway to the Suzuki-Miyaura cross-coupling of carboxylic acids under redox-neutral conditions.

**Figure 3:**
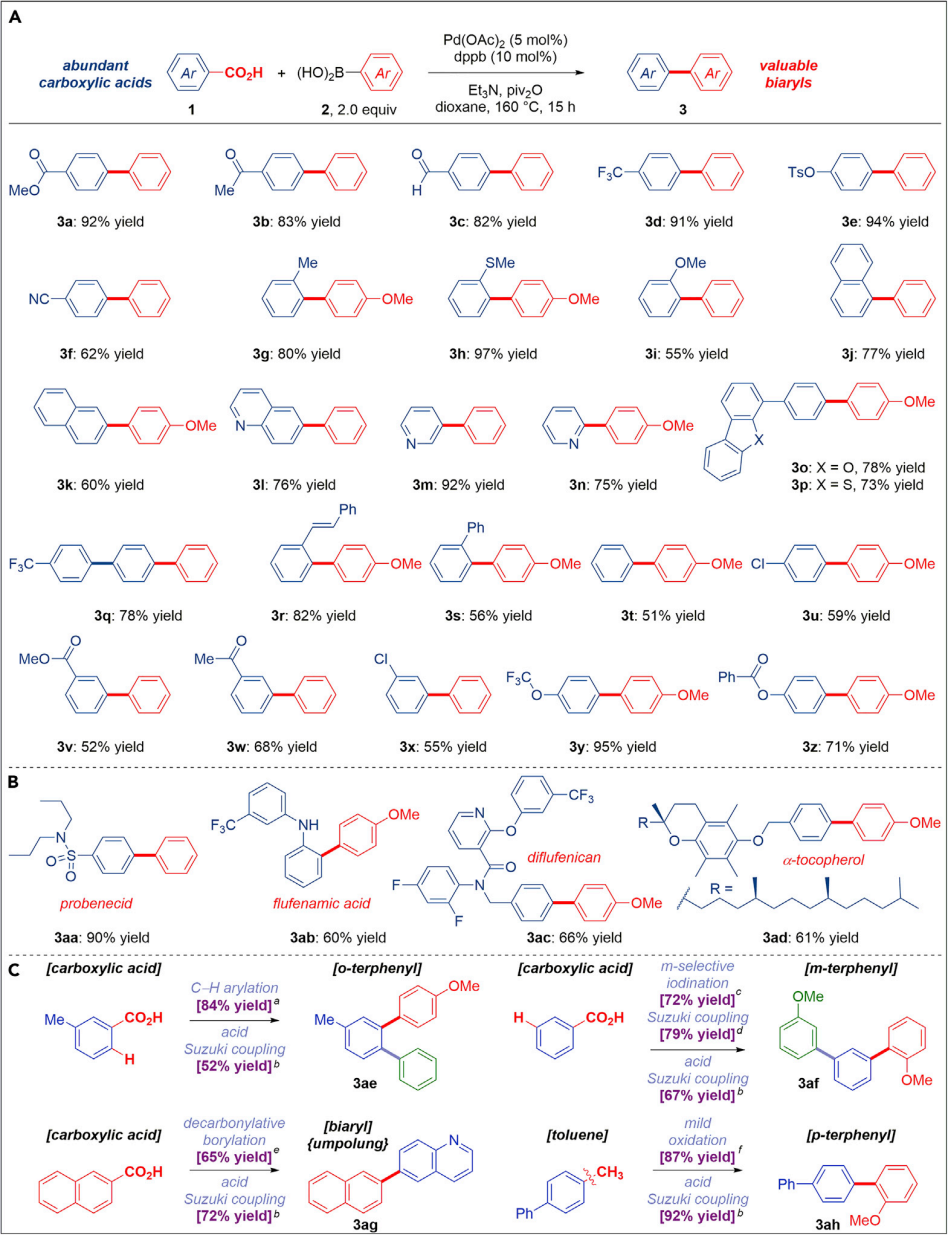
Scope of the Decarbonylative Suzuki-Miyaura Cross-Coupling of Carboxylic Acids: Carboxylic Acid Scope (A) Scope of carboxylic acids. (B) Late-stage functionalization. (C) Sequential cross-coupling. Conditions: Carboxylic acid (1.0 equiv.), Ar–B(OH)_2_ (2.0 equiv.), Pd(OAc)_2_ (5 mol %), dppb (10 mol%), Et_3_N (1.5 equiv.), piv_2_O (1.5 equiv.), H_3_BO_3_ (1.5 equiv.), dioxane, 160°C, 15 h. dppb, 1,4-bis(diphenylphosphino)butane; piv, pivaloyl.

The scope of this process is remarkably broad. In all examples, carboxylic acids were used directly without any preactivation steps. As shown in Figure 3A, a wide range of carboxylic acid substrates are compatible, including tolerance to many functional groups that might be exploited in a myriad of downstream transformations. Esters (**3a**), ketones (**3b**), aldehydes (**3c**), trifluoromethyl groups (**3d**), tosylates (**3e**), and nitriles (**3f**) provide the biaryl products in high yields. Steric substitution, including *ortho*-alkyl (**3g**), *ortho*-thiomethyl (**3h**), *ortho*-methoxy (**3i**), as well as 1-naphthyl (**3j**), proved compatible. Note that decarboxylative biaryl syntheses typically require an activating substituent to favor decarboxylation (Gooßen et al., 2006, Gooßen et al., 2008, Dzik et al., 2012), whereas this is not needed in the present process. Polyaromatic (**3k**) and heterocyclic substrates (**3l–3p**), such as naphthalene, quinoline, pyridines, benzofuran, and benzothiophene, gave the cross-coupling adducts with high selectivity. Notably, owing to the activating role of carboxylic acids in the conventional cross-coupling strategies (Miyaura and Suzuki, 1995, Lennox and Lloyd-Jones, 2014), the present process can be readily utilized in the synthesis of terphenyls, including push-pull compounds (**3q**), and conjugated stilbenes (**3r**), which are widely exploited in the synthesis of functional materials (Beller and Blaser, 2012). Furthermore, electronically unactivated carboxylic acids (**3t**) as well as reactive functional groups, such as chloro (**3u**), ester (**3v**), ketone (**3w**), trifluoromethyl ether (**3y**), and phenolic ester (**3z**), also delivered the corresponding biaryls in good to excellent yields. The latter example is particularly noteworthy as it highlights compatibility of the present process with highly activated phenolic esters, which can be reacted under forcing Ni catalysis (Muto et al., 2015). This unique selectivity is predicated on selective activation of carboxylic acid derivatives enabled by transition metal catalysis (resonance energy, PhC(O)–Opiv = 5.1 kcal/mol versus PhC(O)–OPh, 9.3 kcal/mol, barrier to rotation) (Zhao and Szostak, 2019).

The synthetic potential of this method is showcased in the direct functionalization of pharmaceuticals and bioactive natural products (Figure 3B), including probenecid (**3aa**), flufenamic acid (**3ab**), diflufenican (**3ac**), and tocopherol (**3ad**), highlighting the potential impact of the present protocol for late-stage introduction of biaryl architectures directly exploiting the carboxylic acid functional group. The utility of this direct cross-coupling strategy is further emphasized by the unique capacity of carboxylic acids to act as traceless activating groups (Figure 3C). To this end, metal-catalyzed C–H functionalizations directed by a carboxylic acid (**3ae**) as well as metal-free electrophilic halogenation (**3af**) significantly expand the pool of carboxylic acid precursors available for cross-coupling (Twilton et al., 2017, Knappke and Jacobi von Wangelin, 2010). The combination with decarbonylative borylation (Liu et al., 2018) to furnish organoboranes directly from carboxylic acids (**3ag**) and valorization of toluenes (**3ah**) (Figure 3C) offers a new opportunity for adopting in synthetic processes.

**Figure 4:**
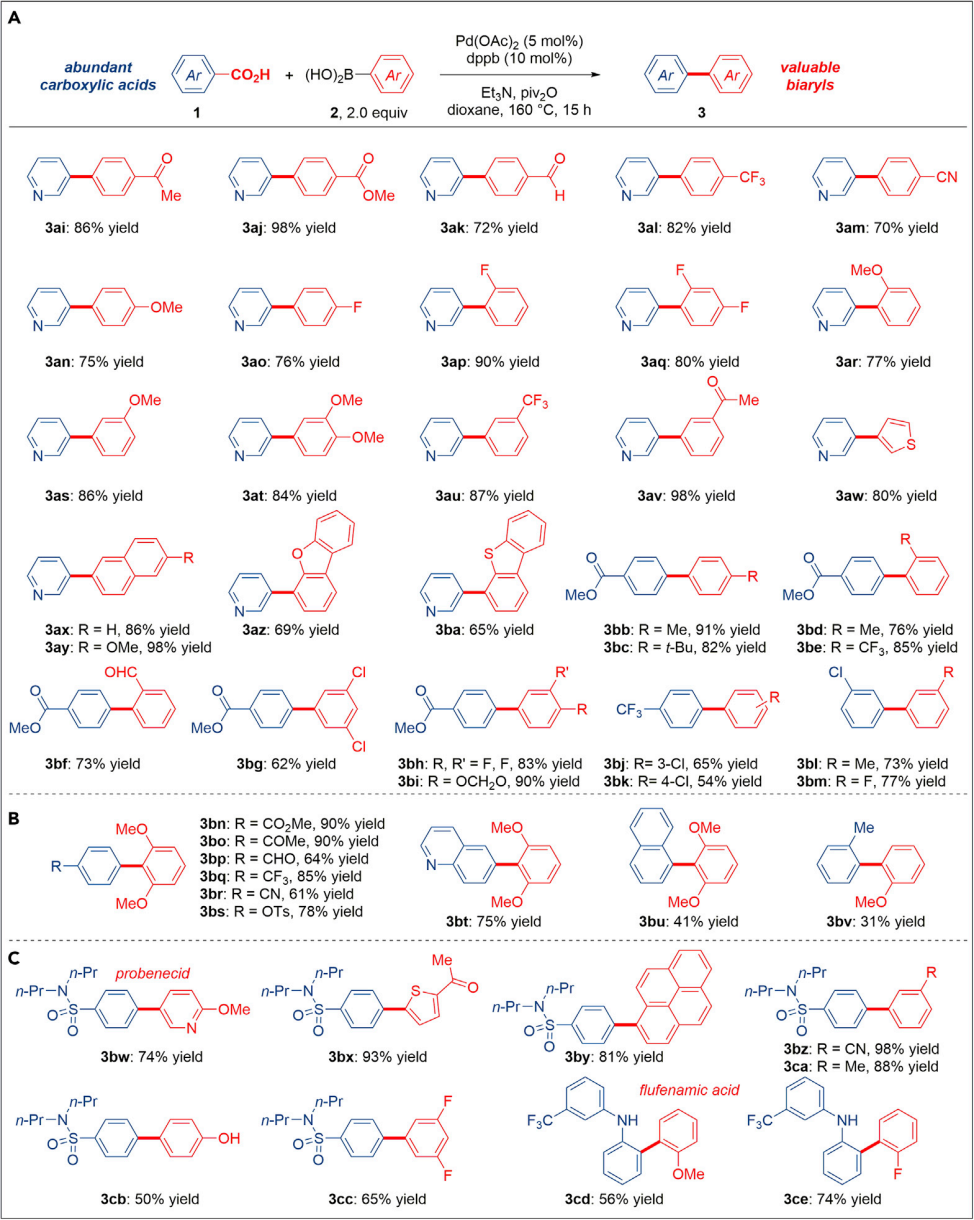
Scope of the Decarbonylative Suzuki-Miyaura Cross-Coupling of Carboxylic Acids: Boronic Acid Scope (A) Scope of boronic acids. (B) Cross-coupling of ortho-substituted boronic acids. (C) Late-stage functionalization. Conditions: Carboxylic acid (1.0 equiv.), Ar–B(OH)_2_ (2.0 equiv.), Pd(OAc)_2_ (5 mol %), dppb (10 mol %), Et_3_N (1.5 equiv.), piv_2_O (1.5 equiv.), H_3_BO_3_ (1.5 equiv.), dioxane, 160°C, 15 h. The extension of scope/conditions in passing from Ni to Pd, including functional group tolerance to sulfonates, phenols, anilines, ortho-biphenyls, trifluoromethylethers, and benchtop setup using air-stable catalysts and reagents, low catalyst loading (see Scheme S6), as well as a simple one-pot procedure should be noted.

The scope of the method with respect to the boronic acid coupling partner was also investigated, as shown in Figure 4. Pleasingly, we found that a wide range of aryl boronic acids are amenable to this biaryl Suzuki-Miyaura cross-coupling process, including deactivated electron-deficient boronic acids bearing an array of sensitive functional groups poised for further modification, such as ketones (**3ai**), esters (**3aj**), aldehydes (**3ak**), and nitriles (**3am**). Furthermore, electron-rich boronic acids that could lead to a competing ketone formation (**3an**) (Malapit et al., 2018) as well as fluorinated (**3ao-3aq**) (Campbell and Ritter, 2015) and sterically hindered boronic acids (**3ar**) are effectively coupled in this protocol. Substitution at the unconjugated 3-position was well-tolerated (**3at-3av**). Moreover, we found that various heterocyclic as well as polyaromatic substrates cross-couple in this redox-neutral protocol with high efficiency (**3aw-3ba**). The utility of this method is further demonstrated in the direct synthesis of biaryls bearing electrophilic carbonyl (**3bb-3bi**) and halogen handles (**3bj-3bm**) for subsequent manipulation by the traditional nucleophilic addition or cross-coupling strategies.

Studies were conducted to determine steric limits of the current protocol (Figure 4B). *Ortho*-substituted biaryls are important structural motifs in biologically active products and functional materials. We found that 2,6-disubstitution on the boronic acid component is well-tolerated, including various useful functional groups on the carboxylic acid cross-coupling partner (**3bn-3bs**). The steric limits of the present protocol are reached with tri-*ortho*-substituted biaryls (**3bu**) as well as with 2,2′-bis-*ortho*-substituted biaryls (**3bv**). These results bode well for future catalyst optimization efforts to promote decarbonylative coupling toward multiply *ortho*- substituted biaryls. Finally, to further demonstrate the powerful opportunity in late-stage derivatization of pharmaceuticals (Blakemore et al., 2018), we conducted a series of direct reactions with probenecid (**3bw-3cc**) and flufenamic acid (**3cd-3ce**) that allow for selective modification of the active core. Clearly, the ubiquity of the carboxylic acid unit in biologically active molecules highlights the advantage of the direct decarbonylative biaryl cross-coupling strategy.

Several additional points are to be noted. (1) In analogy to the classical Pd-catalyzed Suzuki-Miyaura cross-coupling of aryl halides electron-rich boronic acids couple preferentially, whereas electron-deficient electrophiles are more reactive, consistent with facility of metal insertion. (2) Sterically hindered electrophiles and boronic acids are more reactive, consistent with decarbonylation favored by steric demand of acylmetals. It should be noted that more electron-deficient carboxylic acids are also likely to undergo faster oxidative addition. (3) The reaction is scalable (86% yield on gram scale) and efficient at low catalyst loading (81% yield, 0.25 mol% [Pd]). (4) Finally, the reaction setup can be further simplified by using commercially available precatalyst (PdCl_2_(dppb), 5 mol%, 70% yield). These facts bode well for a broad spectrum of applications in various aspects of synthetic chemistry.

Furthermore, it is worthwhile to note that the vast majority of biaryl products reported here cannot be synthesized using currently available methods engaging ubiquitous carboxylic acids. Typically, only ortho-substituted or electronically biased benzoic acids are suitable substrates for decarboxylative Suzuki cross-coupling, whereas the present method could be employed for any functionalized position on the benzene ring of carboxylic acids as well as for electron-donating, electron-neutral, or electron-withdrawing carboxylic acid substrates. In the same vein, decarboxylative Suzuki cross-coupling typically requires bimetallic catalysis, whereas the present catalytic system only needs palladium single metal catalyst as a consequence of well-controlled decarbonylation. The absence of an exogeneous base represents a significant advantage because it enables much broader scope and generality. The prevalence and orthogonal nature of carboxylic acids enable the preparation of biaryls that are not easily accessible by other cross-coupling methods using halides or pseudohalides as cross-coupling partners. The use of palladium represents a significant advantage because it enables much broader tolerance and is more universally applicable than nickel. As a key design strategy, the present method involves a one-pot process directly involving ubiquitous carboxylic acids in which all reaction components are combined at the same time, which enables operational simplicity and rapid testing not available by other methods.

In conclusion, decarbonylative biaryl synthesis from carboxylic acids represents a powerful tool for the synthesis of complex biaryls using ubiquitous and orthogonal carboxylic acid cross-coupling partners. This decarbonylative strategy embodies a complementary approach to the traditional loss of carbon dioxide. The broad substrate scope, operational simplicity, and the potential to apply in complex molecule synthesis make it evident that decarbonylative cross-couplings (Stephan et al., 1998, Zhang et al., 2018, Meng and Szostak, 2015, Liu et al., 2019) will likely have a major impact in the modern era of organic synthesis. Future studies will focus on expanding the scope of the present protocol and mechanistic investigations of decarbonylative cross-coupling protocols involving carboxylic acids.

### Limitations of the Study

Tetra-substituted biaryls as well as aryl bromides are not suitable, which supports similar rate of the oxidative addition step of C–Br and C–O bonds. H_3_BO_3_ is required for the efficient biaryl synthesis, which supports O-protonation and prevents protodeboronation. Although cross-coupling of electron-rich arenes is feasible (**3h**, **3i**), this also shows some limitations of the method. Future studies will focus on the development of more active catalyst systems to expand the substrate scope of the decarbonylative coupling.

## Methods

All methods can be found in the accompanying Transparent Methods supplemental file.

## Supplemental Information

Document S1. Transparent Methods, Figures S1–S186, Schemes S1–S6, and Tables S1 and S2

Data S1. The Cartesian Coordinates from the DFT Studies, Related to Figure 2
