# Supplementary material for: Synthesis of Biaryls via Decarbonylative Palladium-Catalyzed Suzuki-Miyaura Cross-Coupling of Carboxylic Acids
Source: iScience. 2019 Aug 17;19:749–59. doi: 10.1016/j.isci.2019.08.021 (PMC6731188; doi:10.1016/j.isci.2019.08.021)
Supplement: Document S1. Transparent Methods, Figures S1–S186, Schemes S1–S6, and Tables S1 and S2 [file mmc1.pdf]

ISCI, Volume *19*

Supplemental Information

Synthesis of Biaryls via Decarbonylative Palladium-Catalyzed Suzuki-Miyaura Cross-Coupling of Carboxylic Acids

Chengwei Liu, Chong-Lei Ji, Zhi-Xin Qin, Xin Hong, and Michal Szostak

Figure S1. DFT-calculated reaction energy profile of the Pd/PCy3-catalyzed Suzuki-Miyaura cross-coupling of benzoic pivalic anhydride with C–O bond cleavage of pivalic acid, related to **Figure 2**

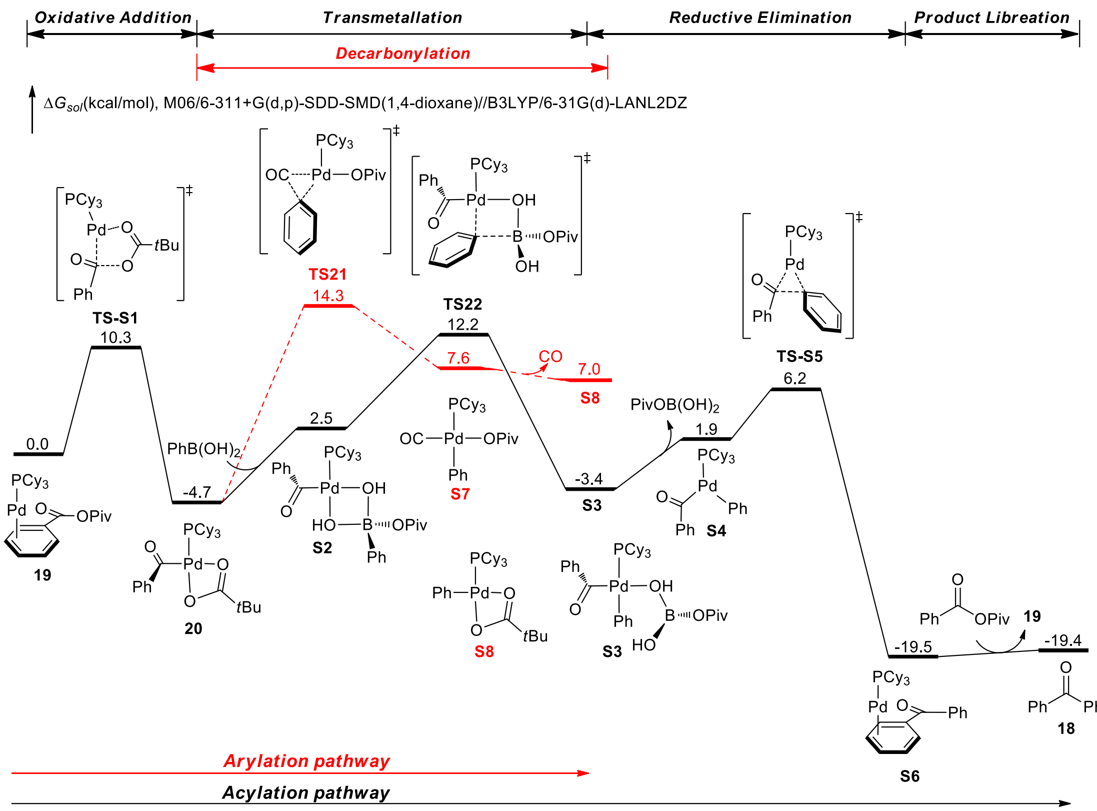

Figure S2. ^{1}H NMR spectrum of **3a**, related to **Figure 3**

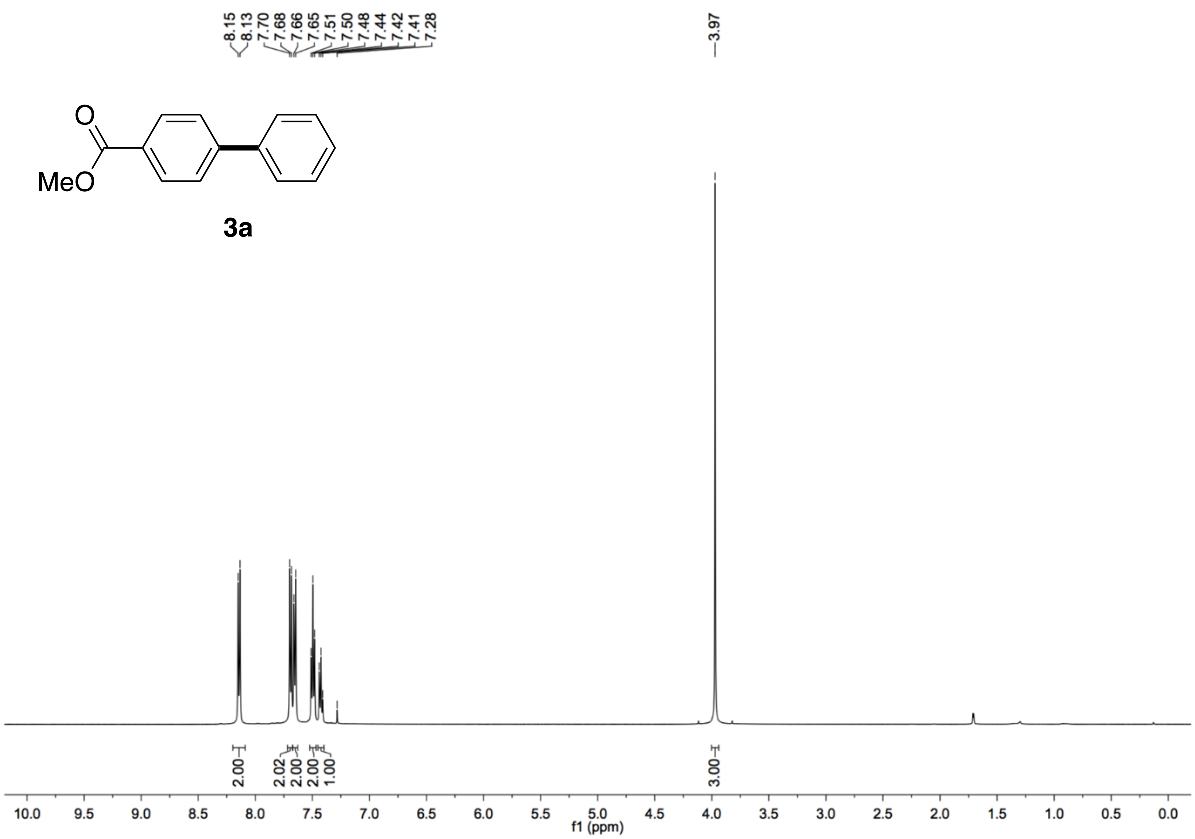

Figure S3. ^{13}C NMR spectrum of **3a**, related to **Figure 3**

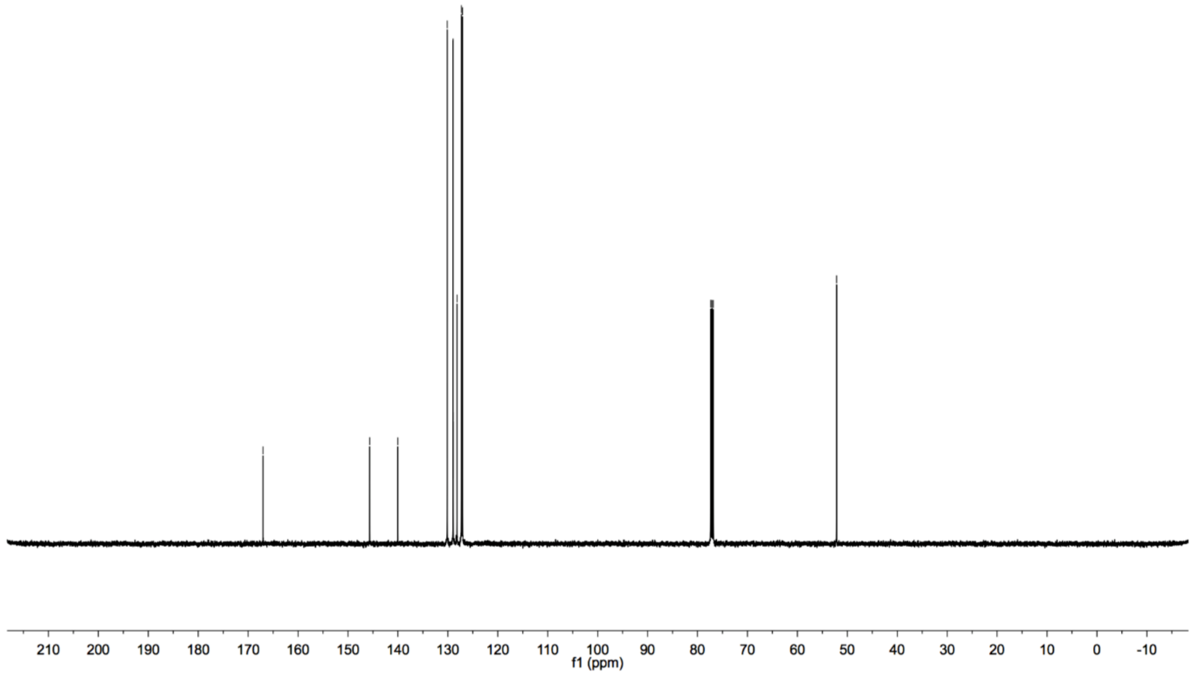

Figure S4. ^{1}H NMR spectrum of **3b**, related to **Figure 3**

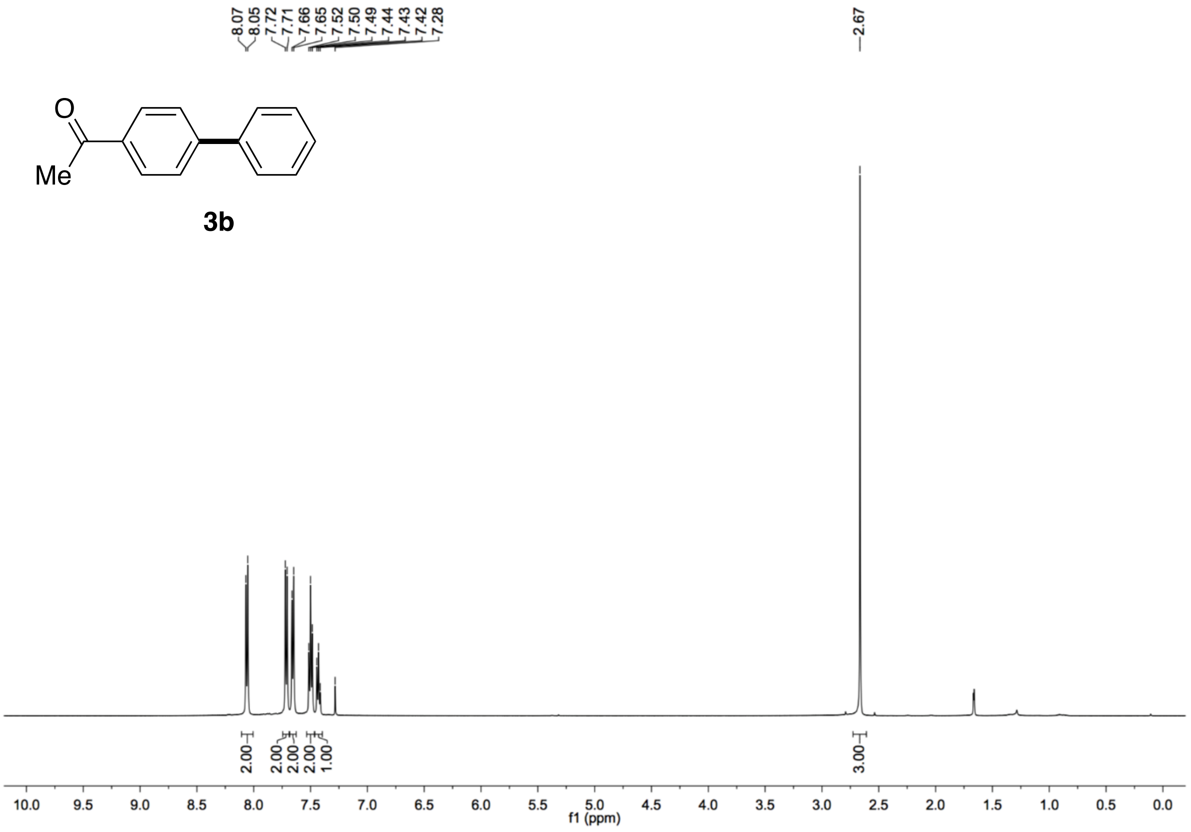

Figure S5. ^{13}C NMR spectrum of **3b**, related to **Figure 3**

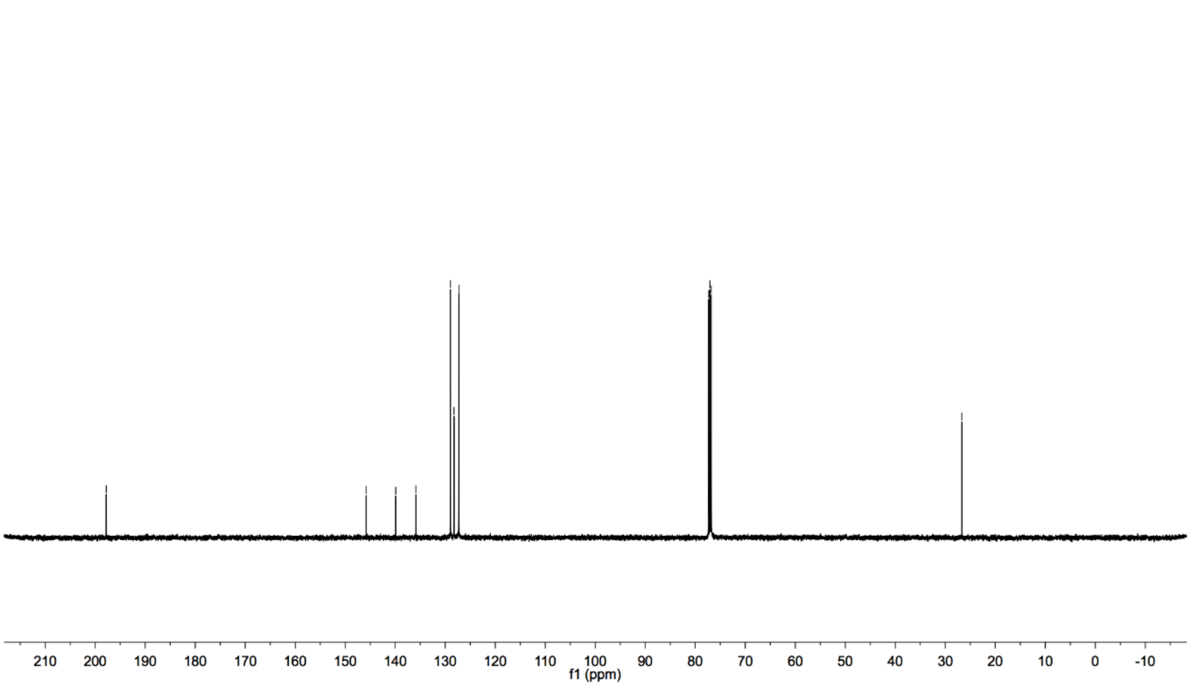

Figure S6. ^{1}H NMR spectrum of **3c**, related to **Figure 3**

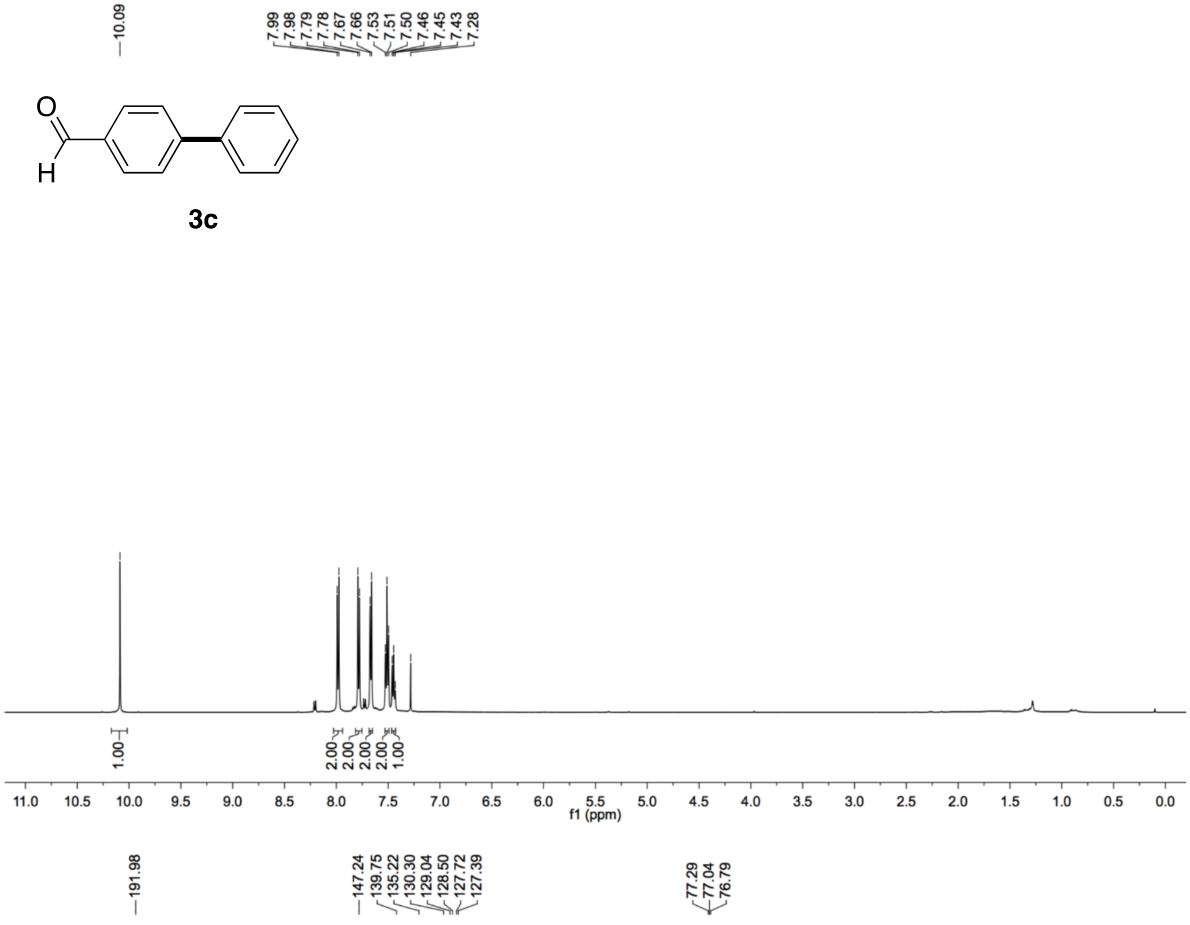

Figure S7. ^{13}C NMR spectrum of **3c**, related to **Figure 3**

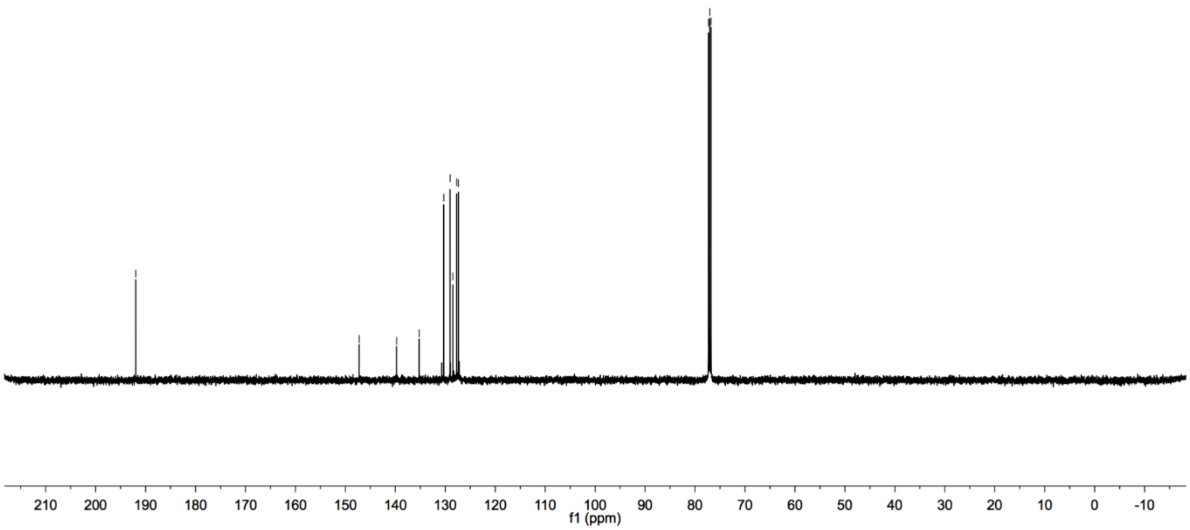

Figure S8. ^{1}H NMR spectrum of **3d**, related to **Figure 3**

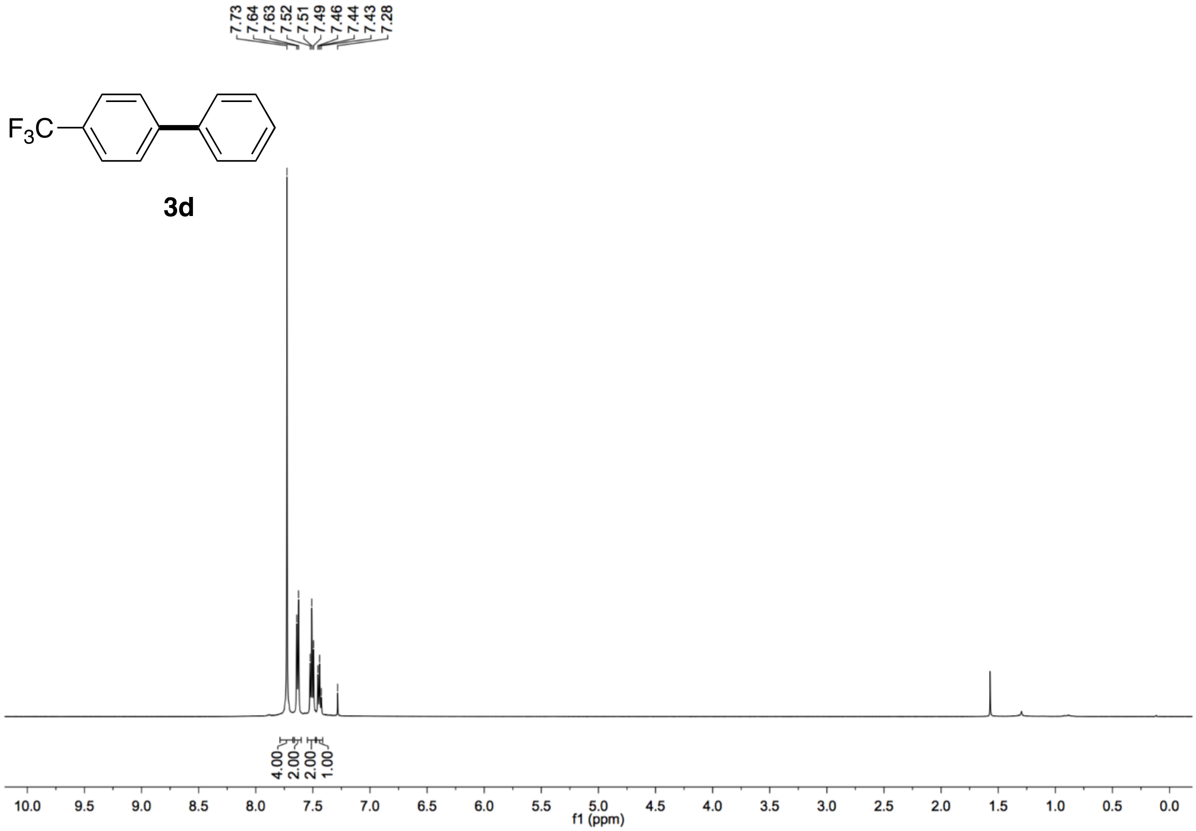

Figure S9. ^{13}C NMR spectrum of **3d**, related to **Figure 3**

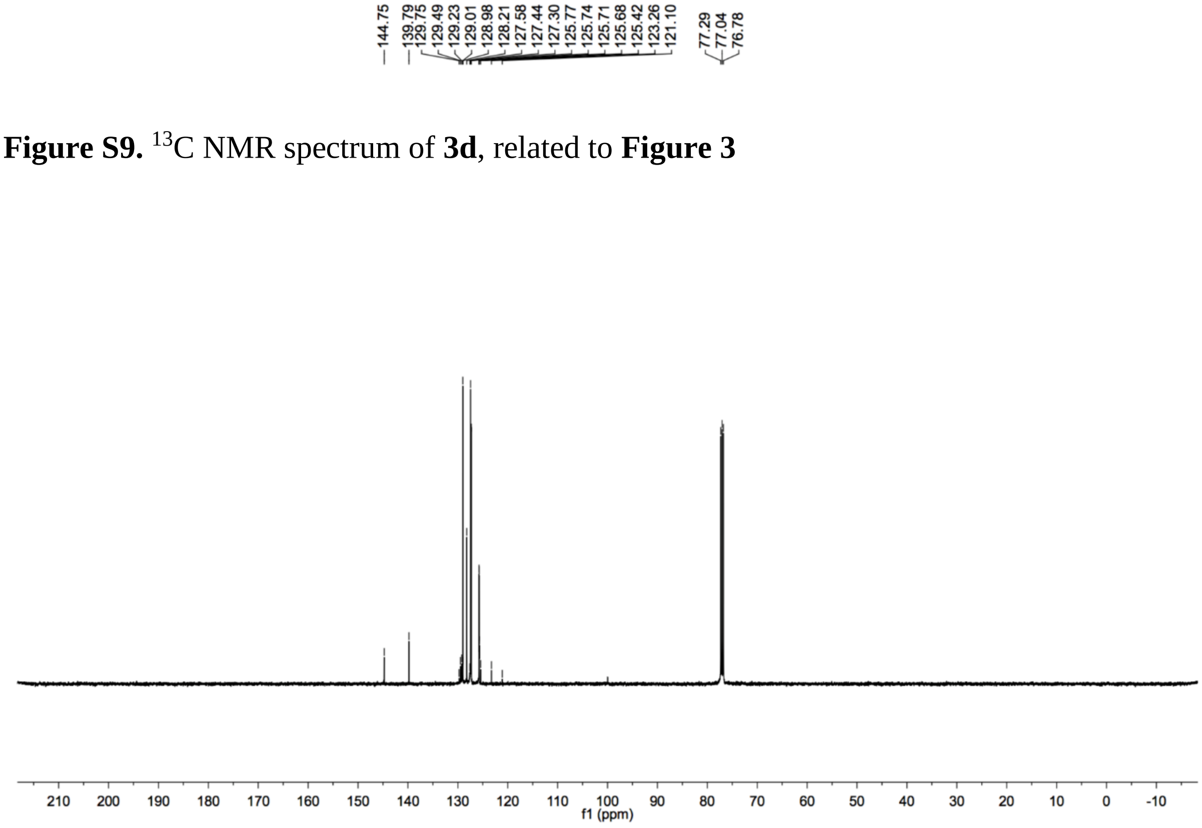

Figure S10. ^{19}F NMR spectrum of **3d**, related to **Figure 3**

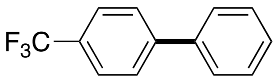

3d

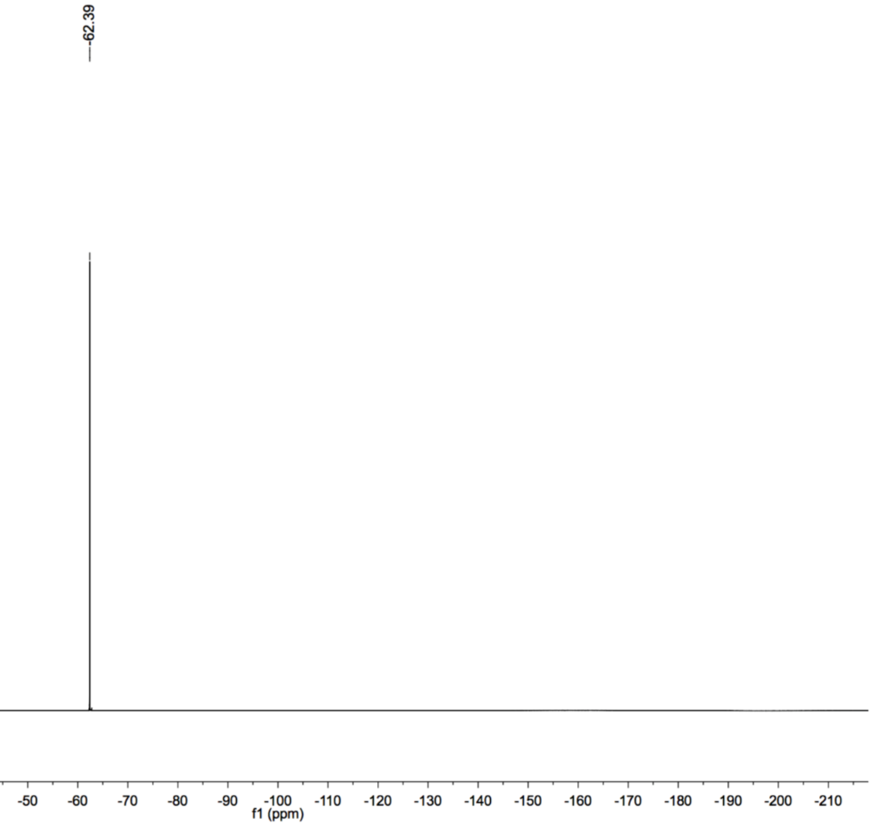

Figure S11. ^{1}H NMR spectrum of **3e**, related to **Figure 3**

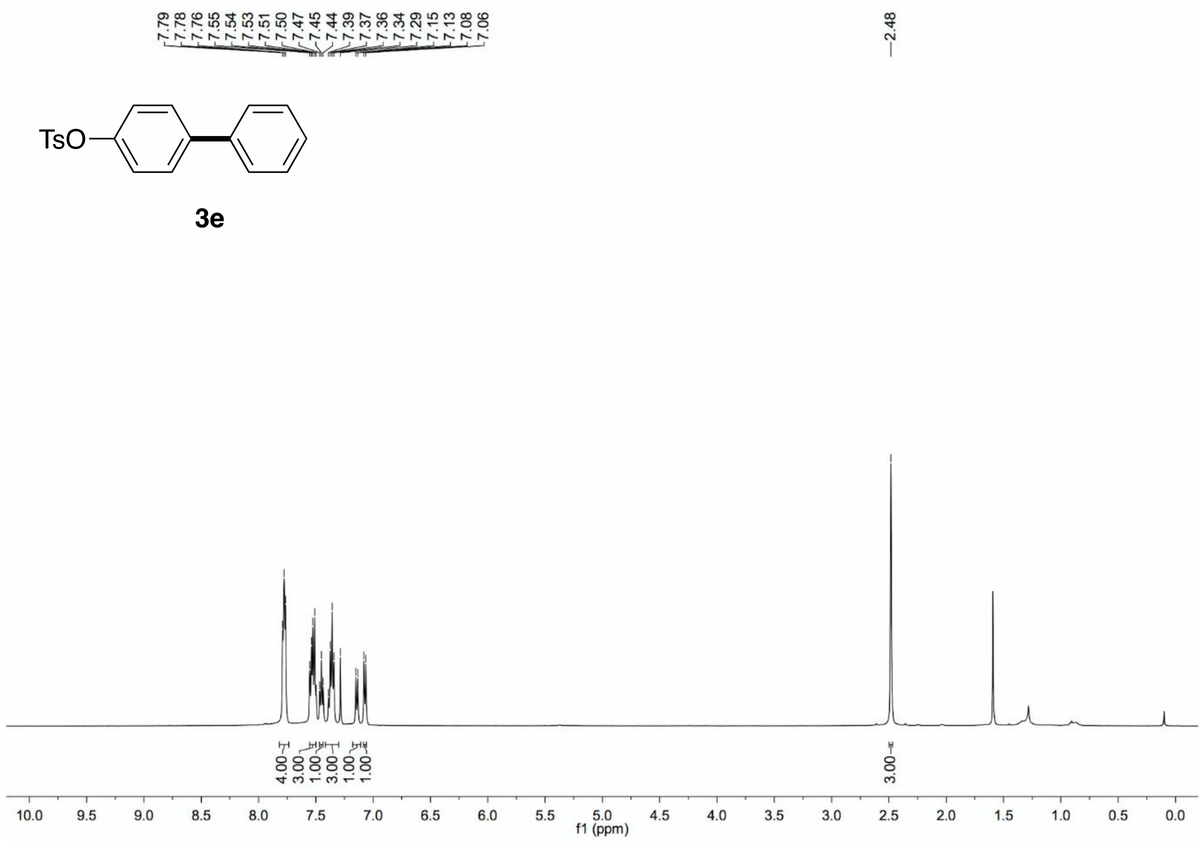

Figure S12. ^{13}C NMR spectrum of **3e**, related to **Figure 3**

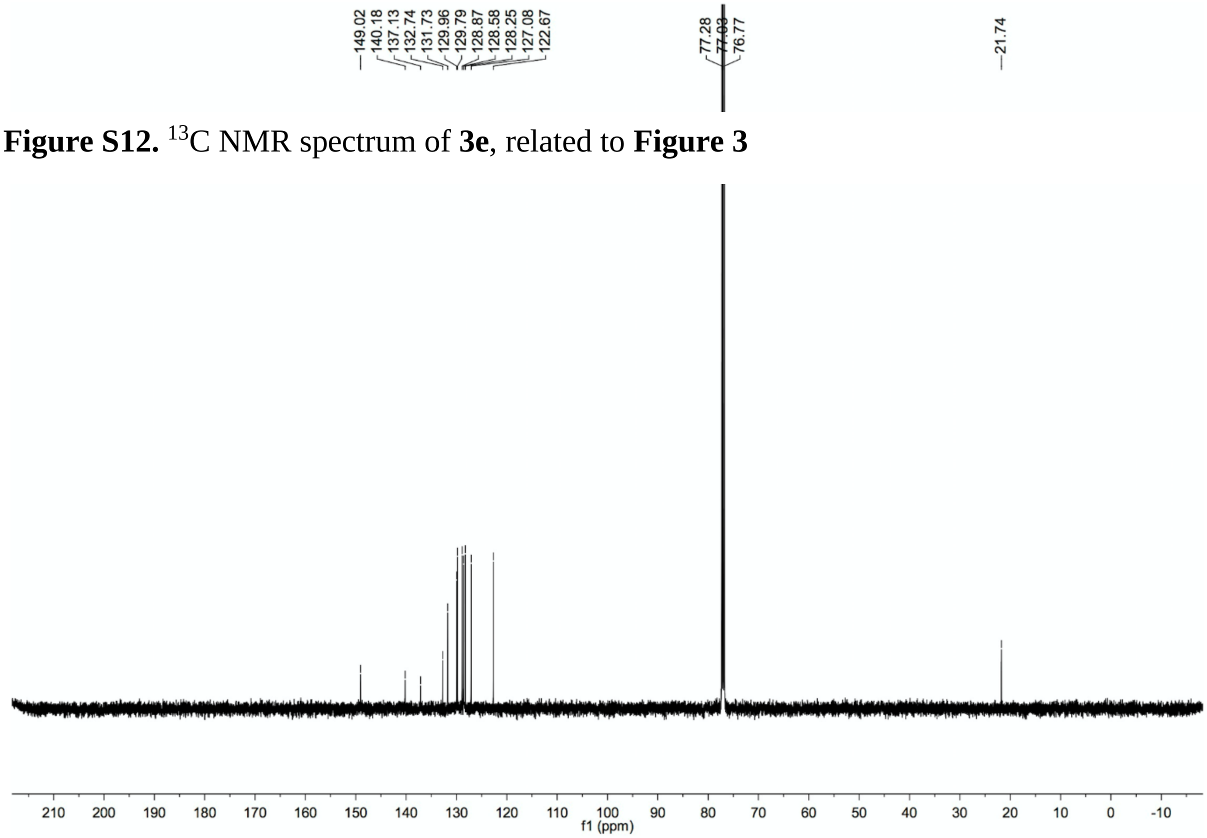

Figure S13. ^{1}H NMR spectrum of **3f**, related to **Figure 3**

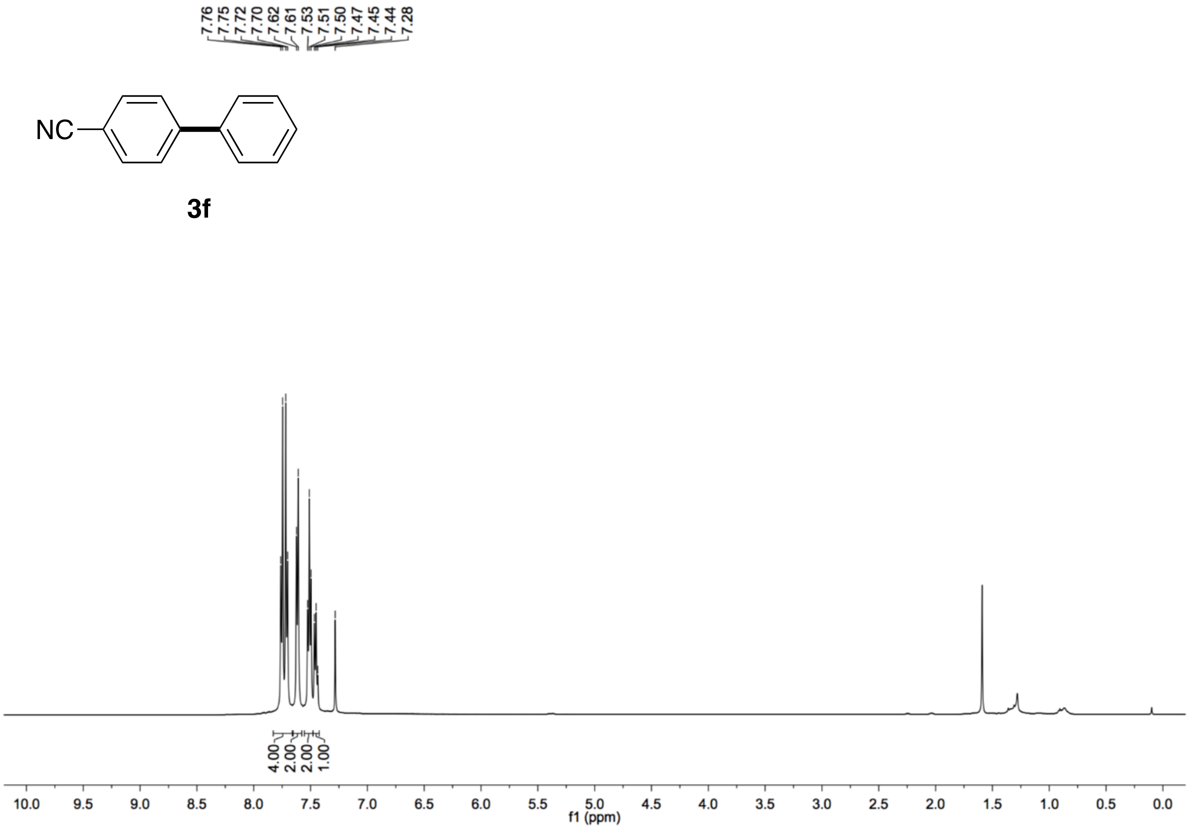

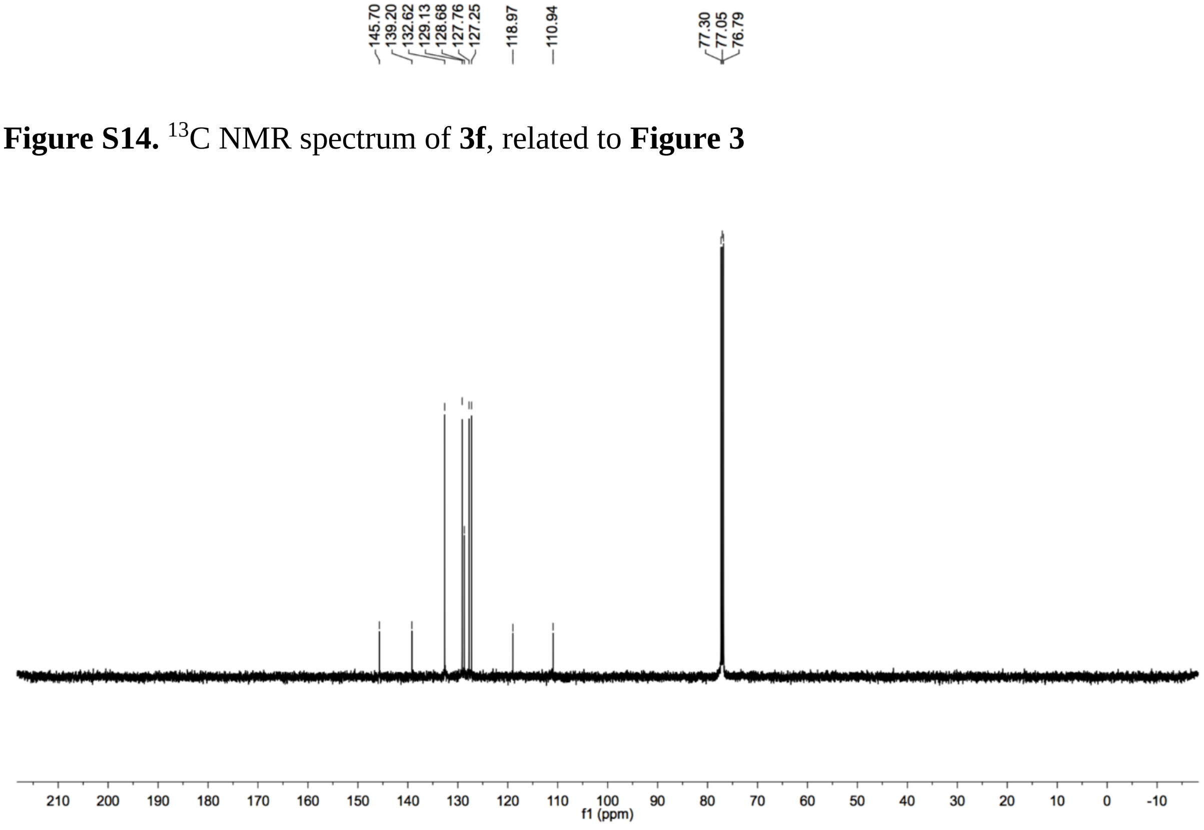

Figure S14. ^{13}C NMR spectrum of **3f**, related to **Figure 3**

Figure S15. ^{1}H NMR spectrum of **3g**, related to **Figure 3**

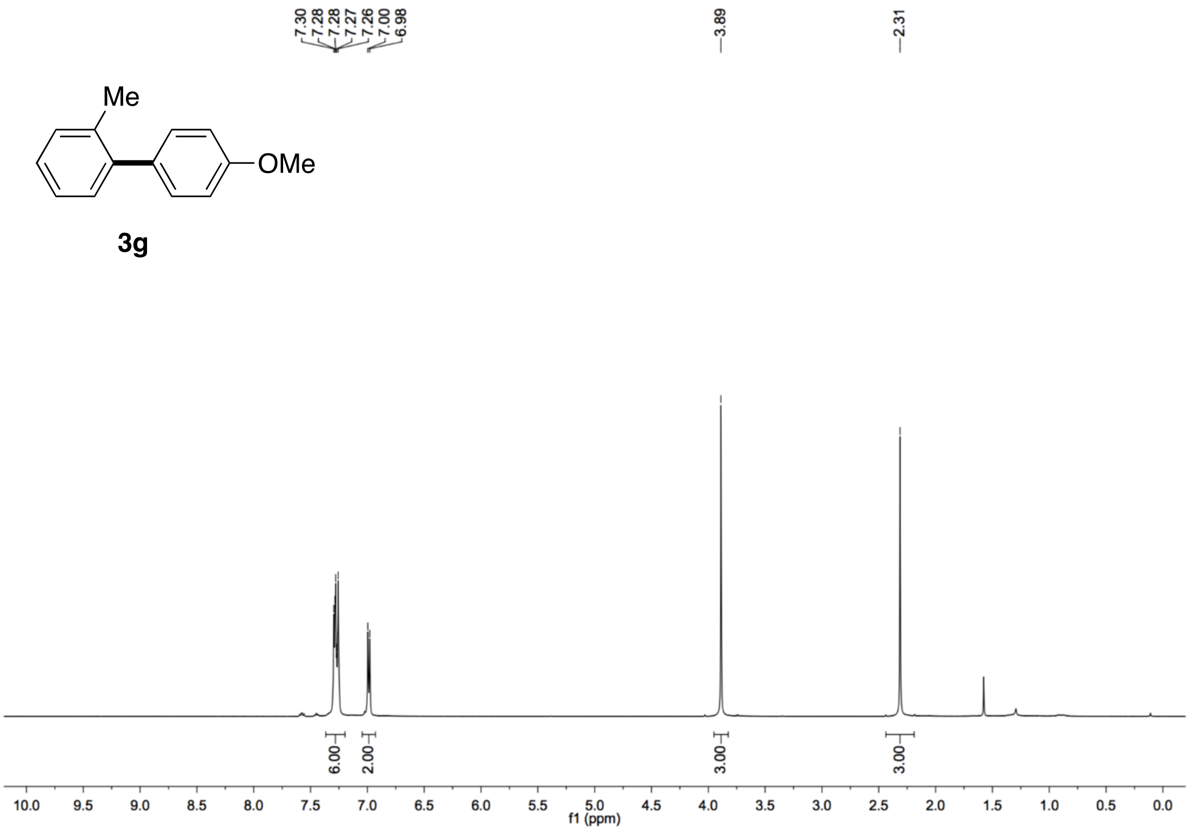

Figure S16. ^{13}C NMR spectrum of **3g**, related to **Figure 3**

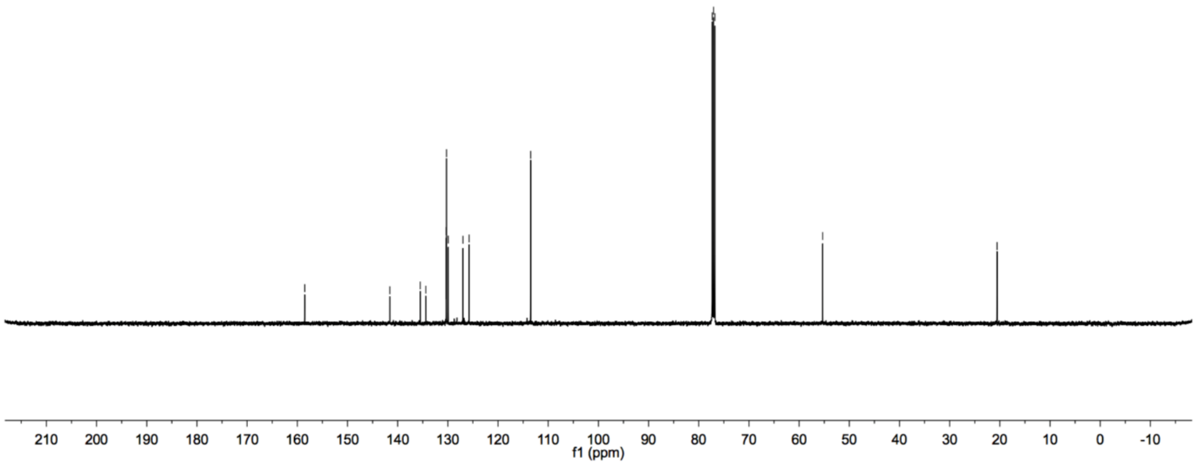

Figure S17. ^{1}H NMR spectrum of **3h**, related to **Figure 3**

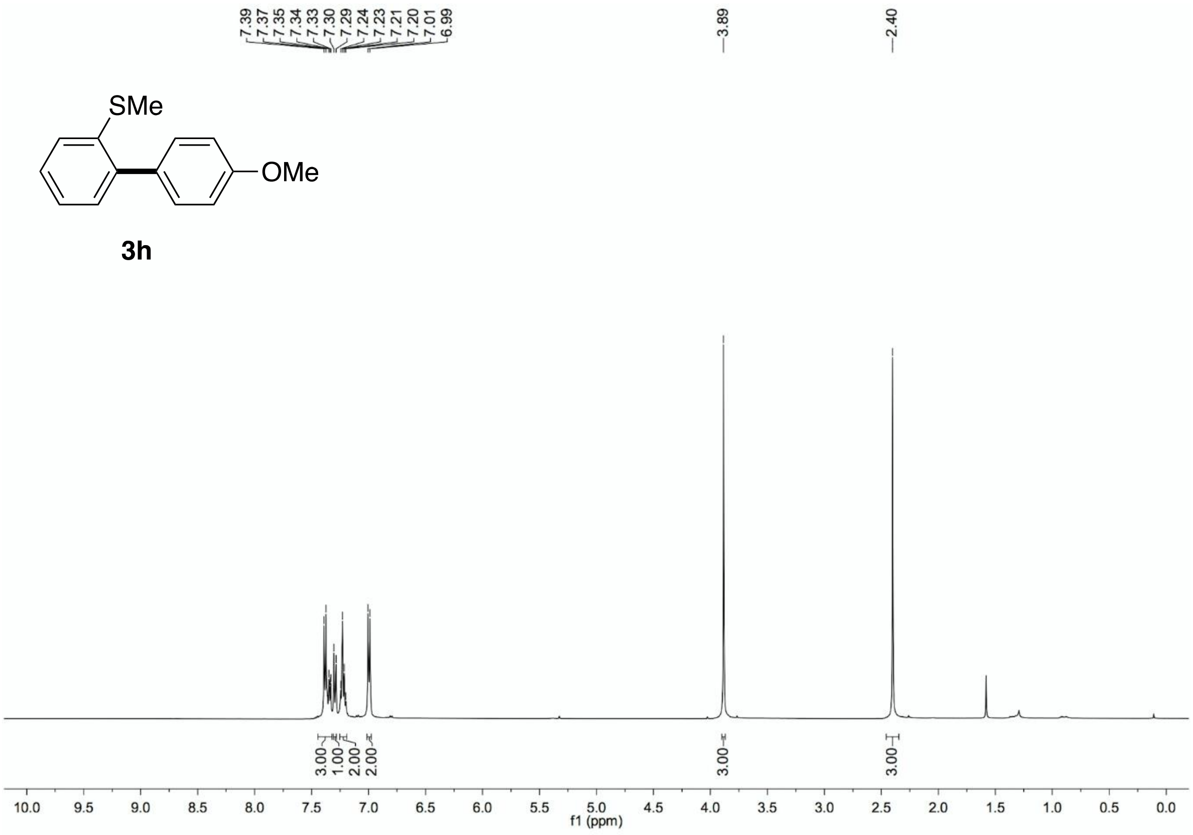

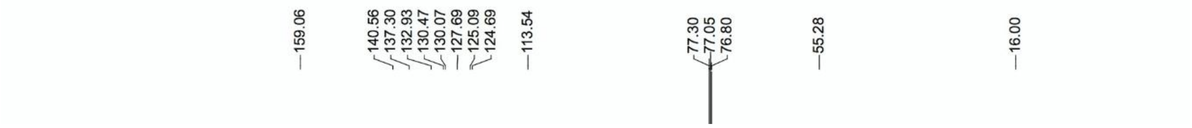

Figure S18. ^{13}C NMR spectrum of **3h**, related to **Figure 3**

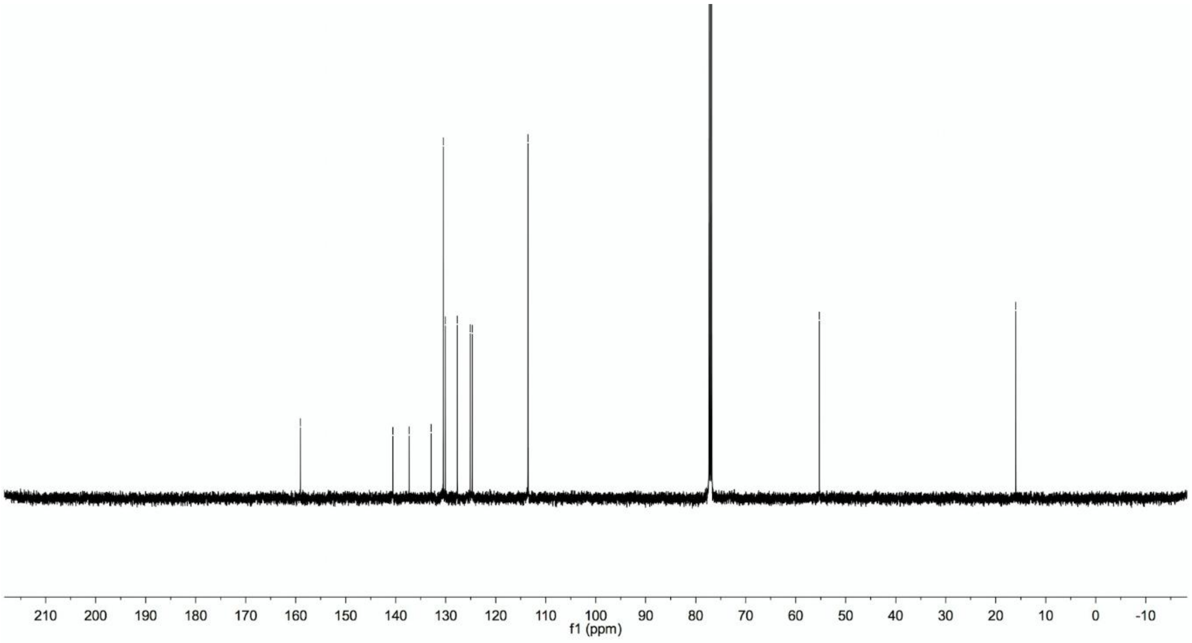

Figure S19. ^{1}H NMR spectrum of **3i**, related to **Figure 3**

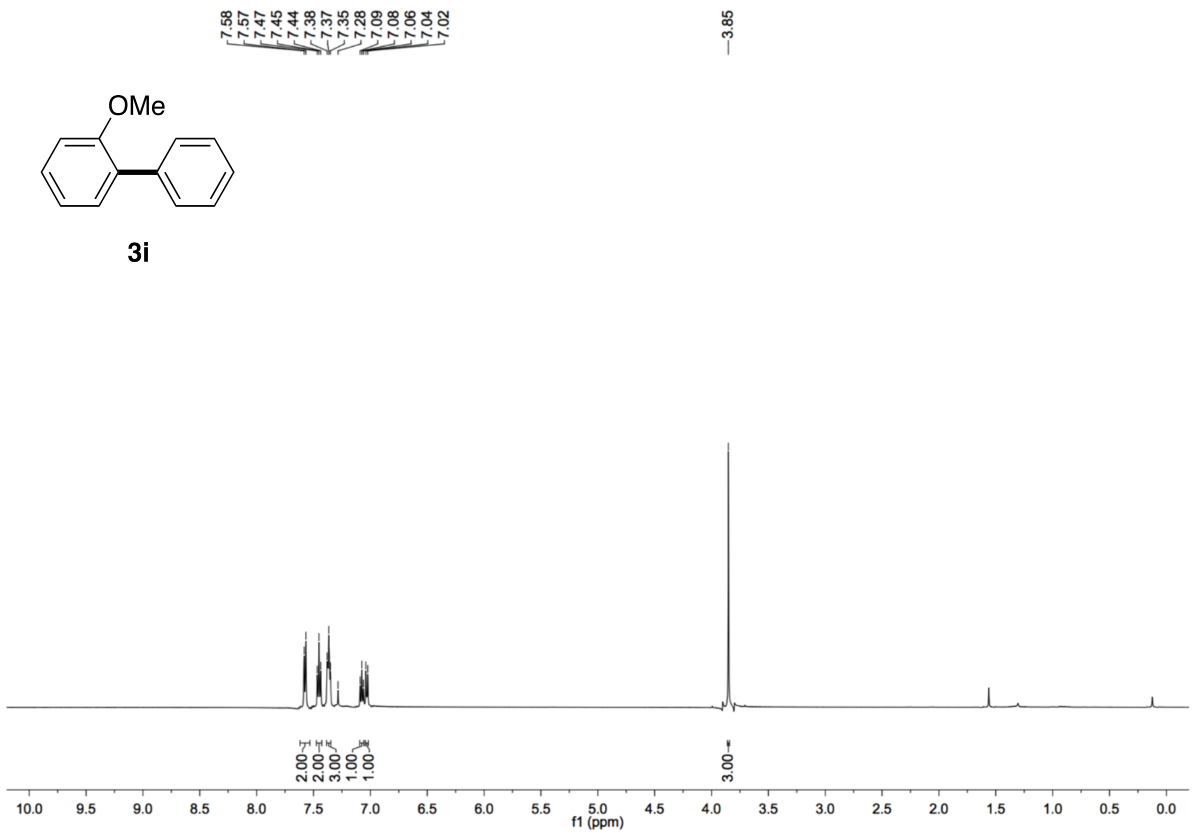

Figure S20. ^{13}C NMR spectrum of **3i**, related to **Figure 3**

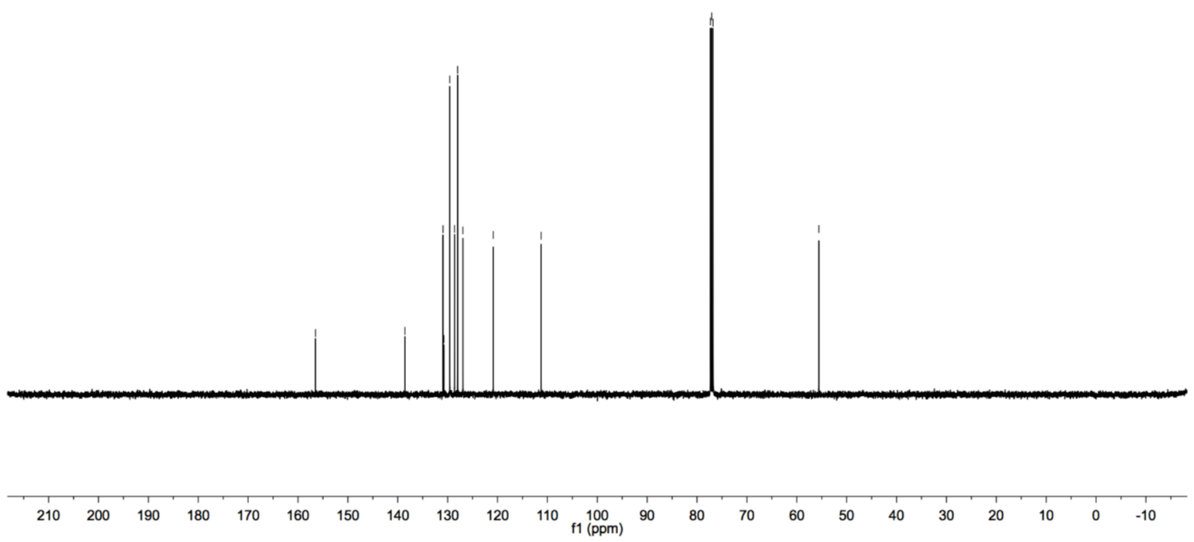

Figure S21. ^{1}H NMR spectrum of **3j**, related to **Figure 3**

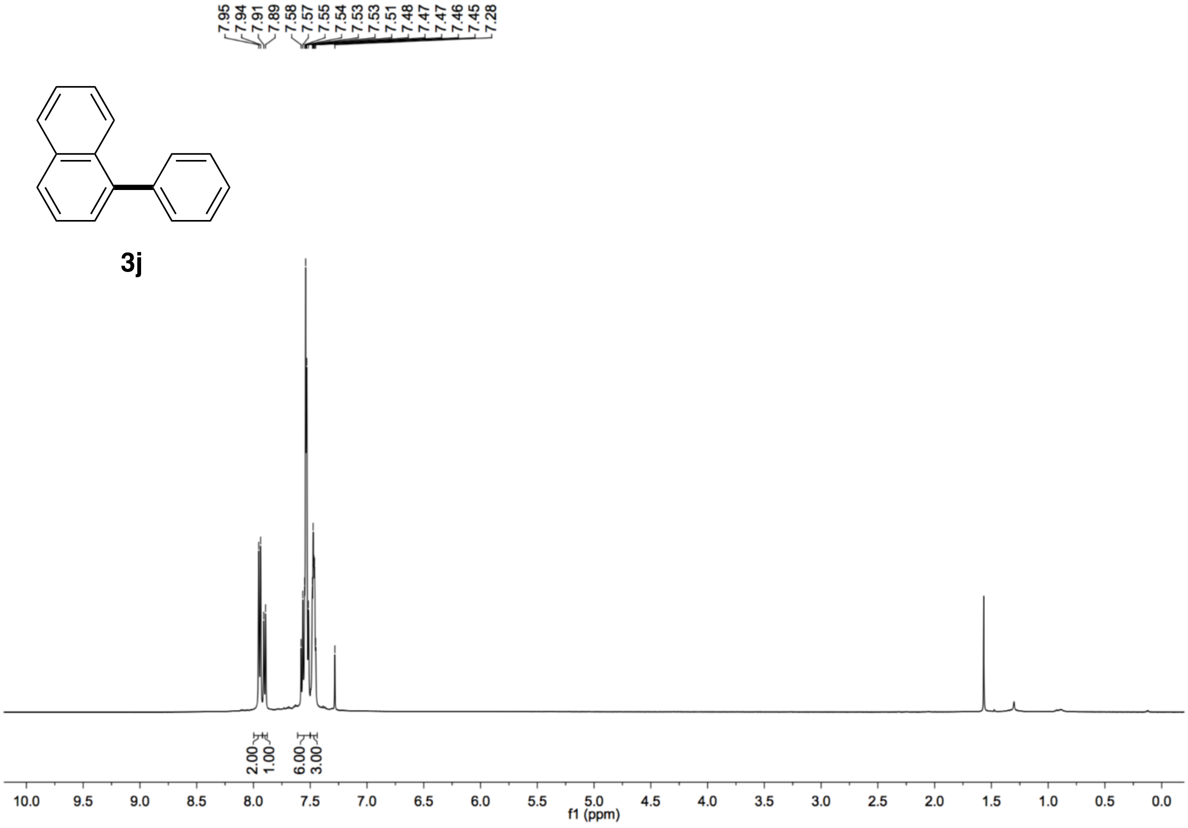

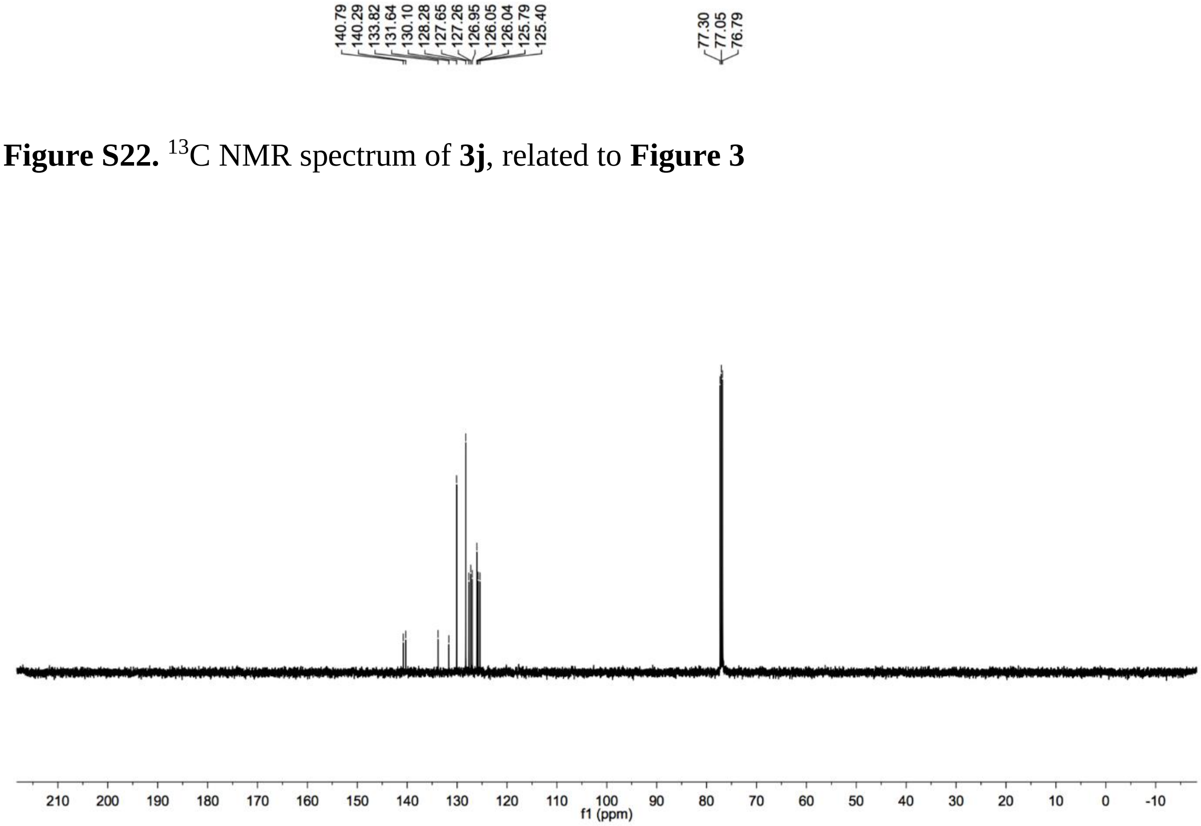

Figure S22. ^{13}C NMR spectrum of **3j**, related to **Figure 3**

Figure S23. ^{1}H NMR spectrum of **3k**, related to **Figure 3**

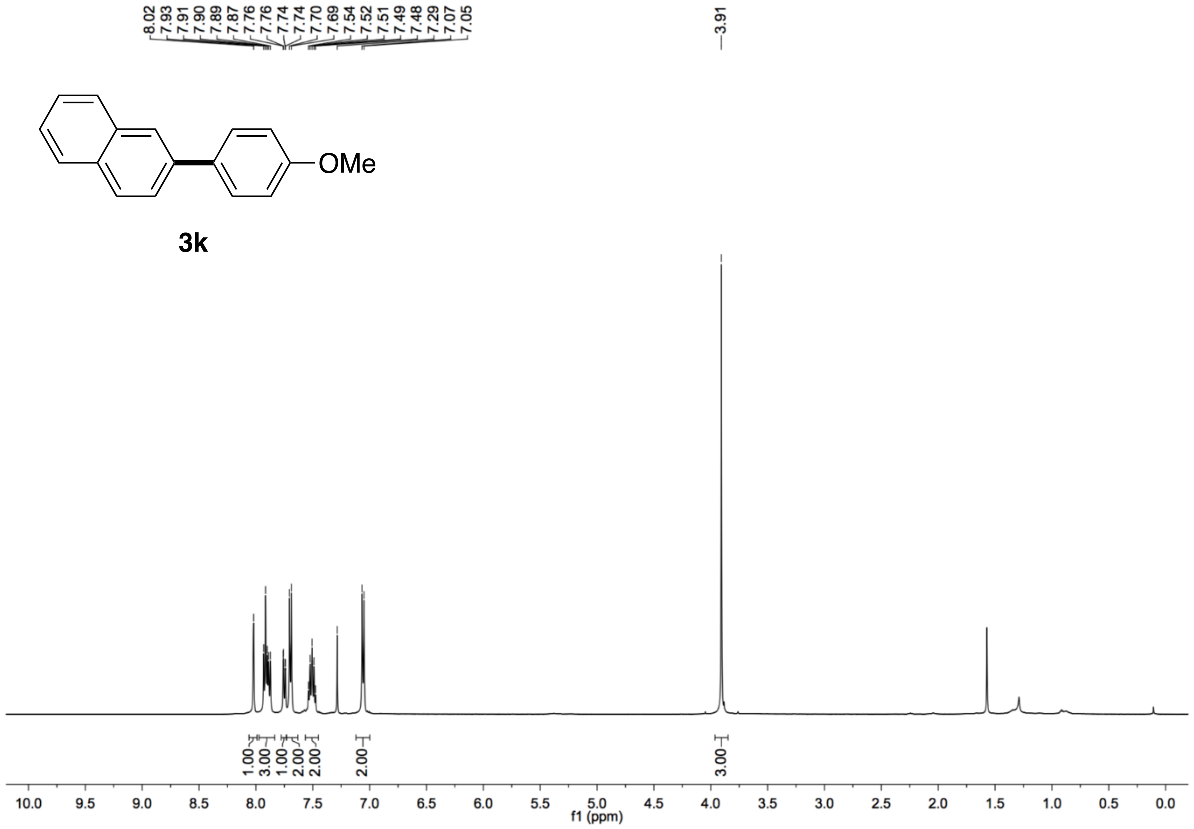

Figure S24. ^{13}C NMR spectrum of **3k**, related to **Figure 3**

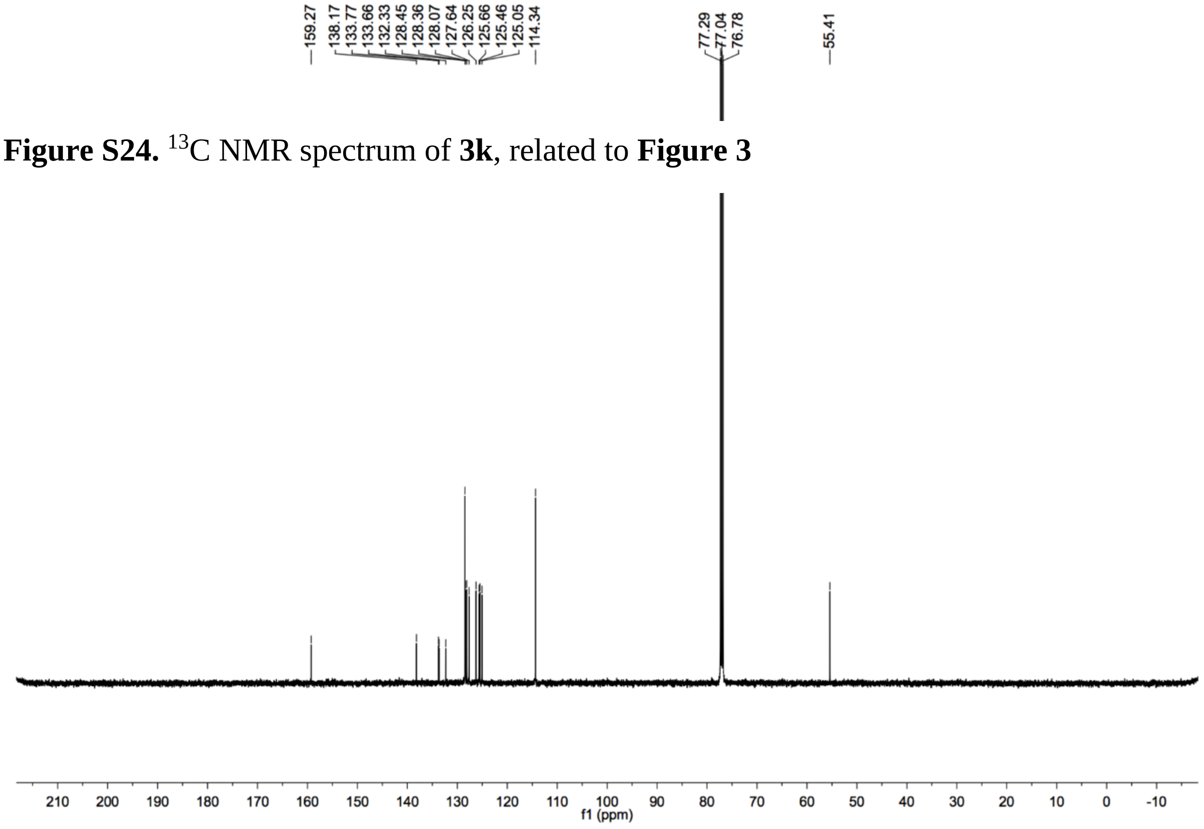

Figure S25. ^{1}H NMR spectrum of **3l**, related to **Figure 3**

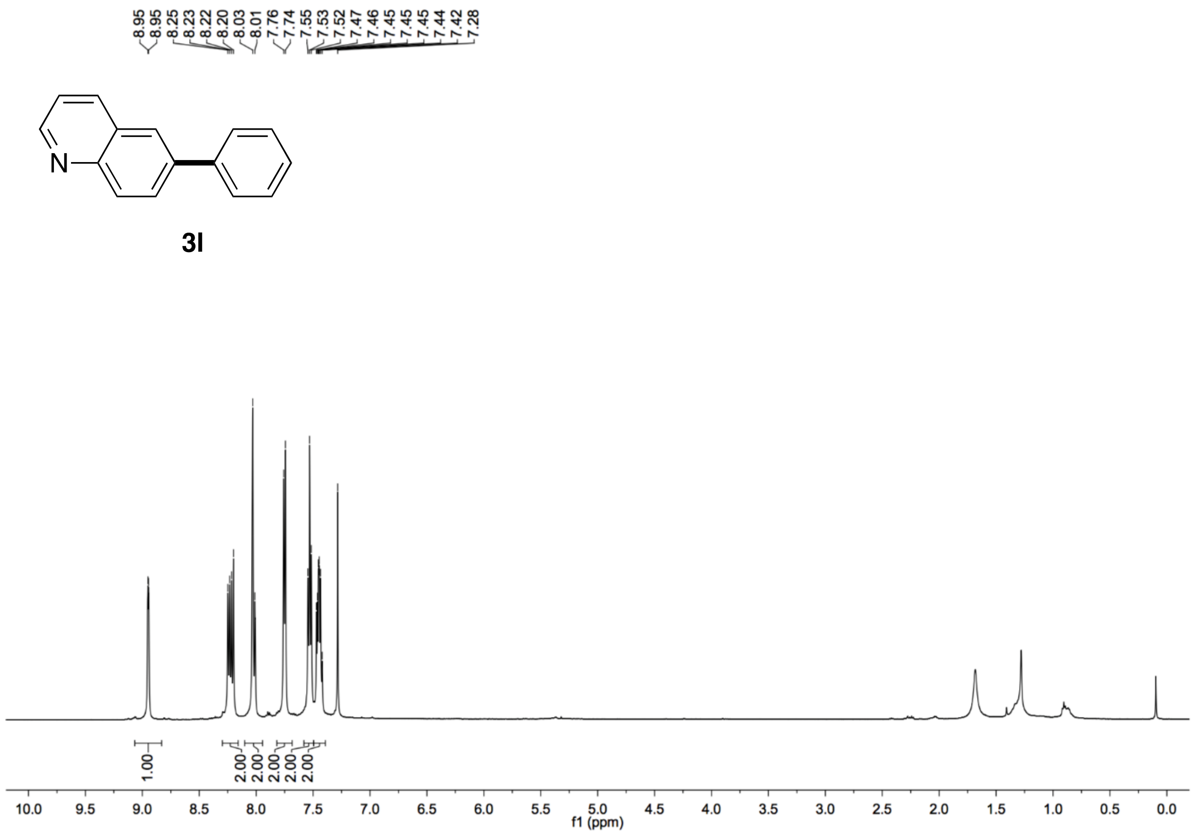

Figure S26. ^{13}C NMR spectrum of **3l**, related to **Figure 3**

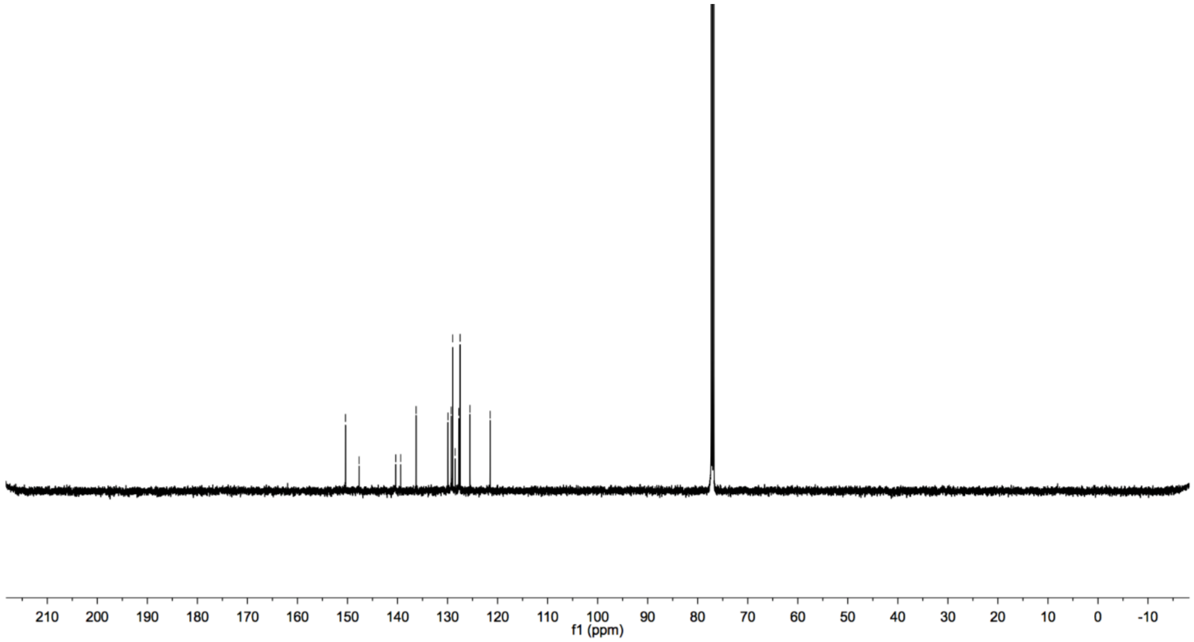

Figure S27. ^{1}H NMR spectrum of **3m**, related to **Figure 3**

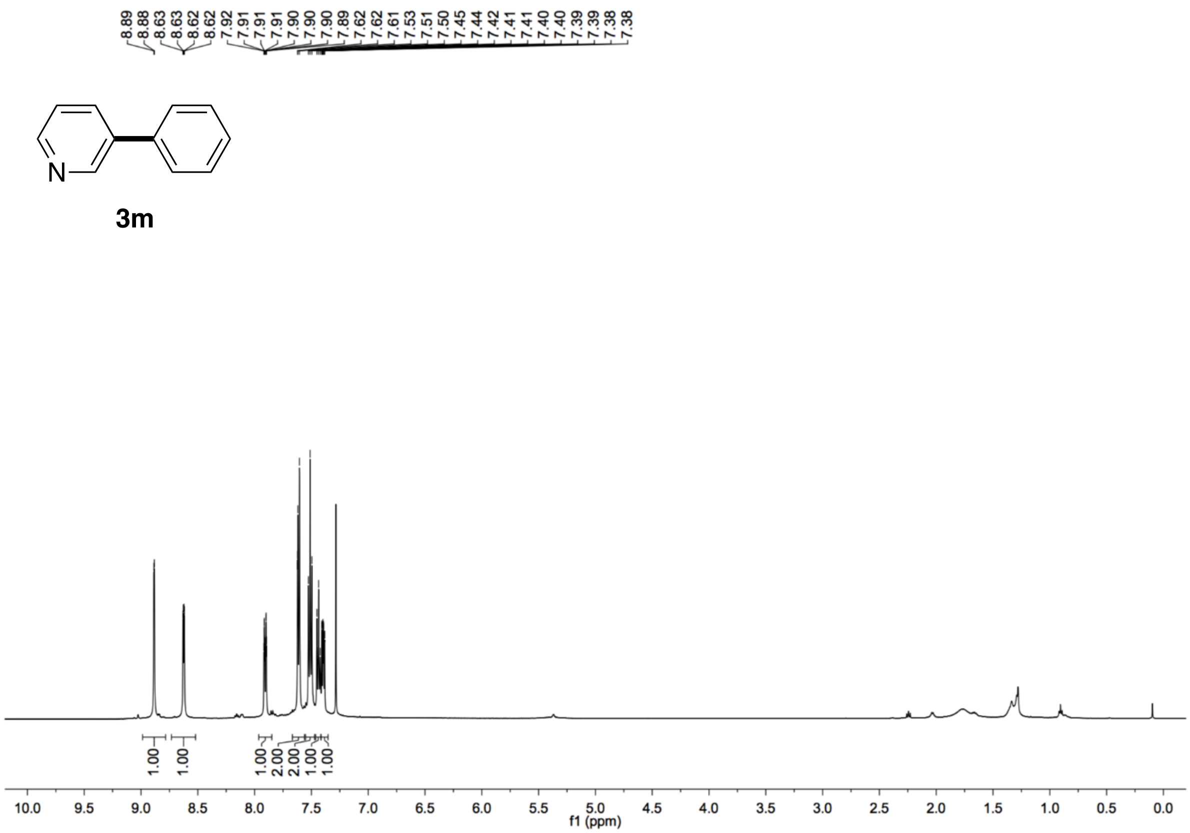

Figure S28. ^{13}C NMR spectrum of **3m**, related to **Figure 3**

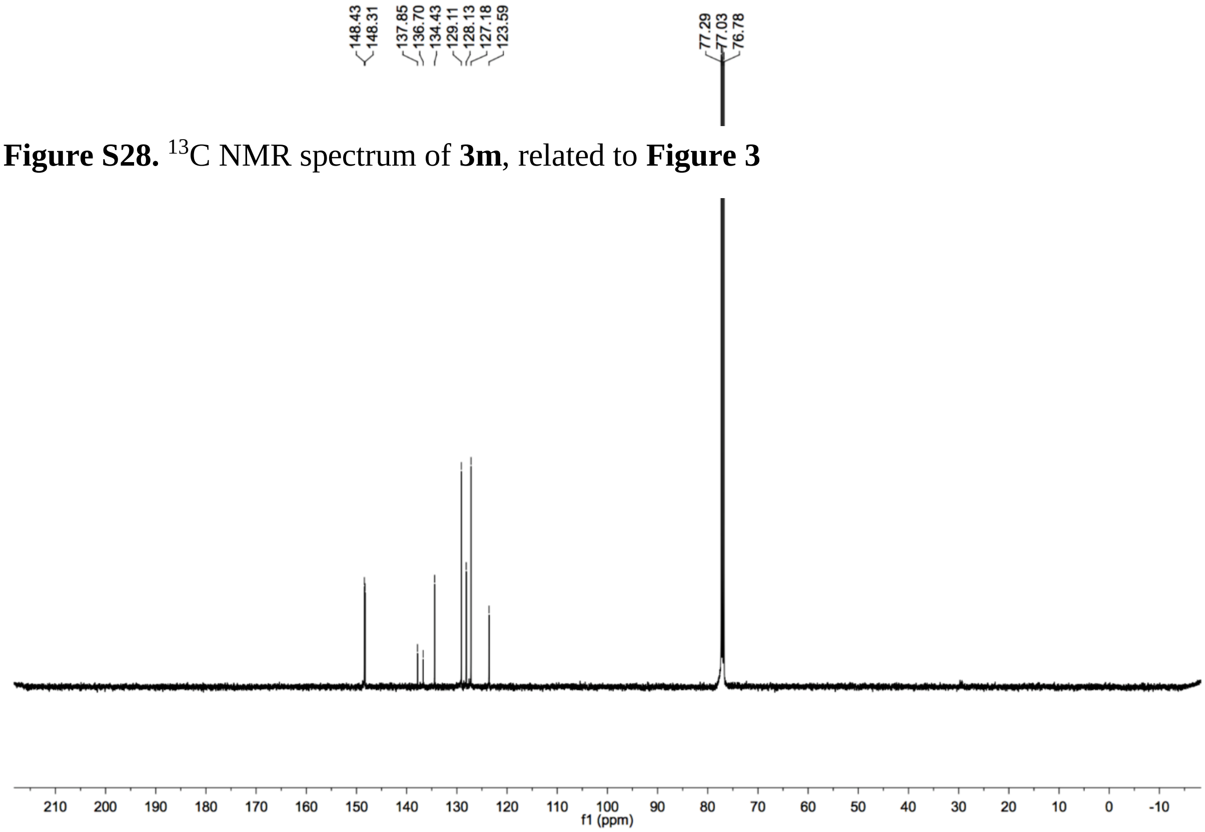

Figure S29. ^{1}H NMR spectrum of **3n**, related to **Figure 3**

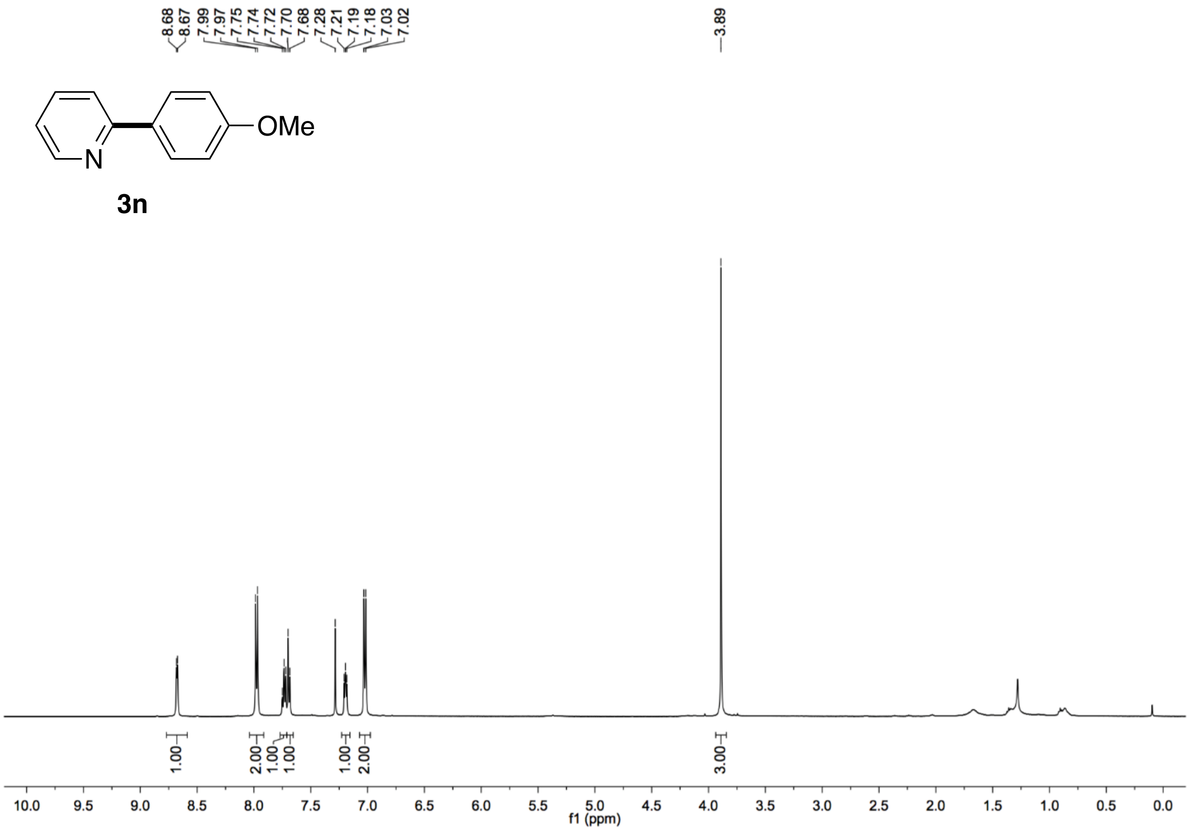

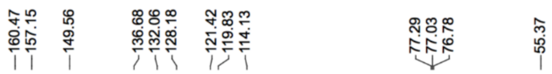

Figure S30. ^{13}C NMR spectrum of **3n**, related to **Figure 3**

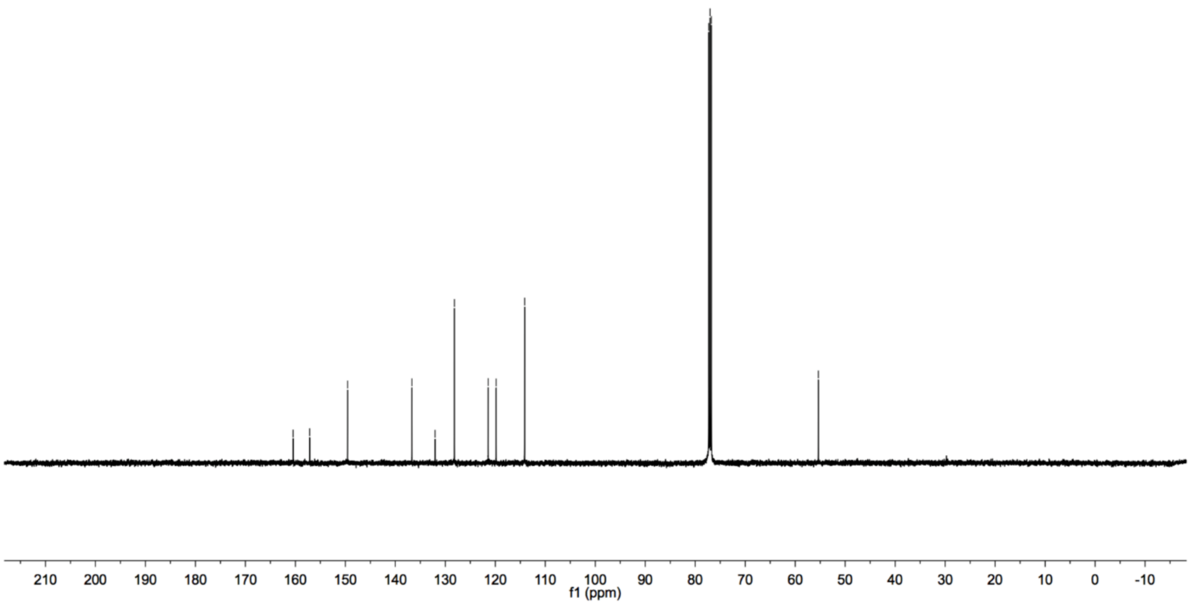

Figure S31. ^{1}H NMR spectrum of **3o**, related to **Figure 3**

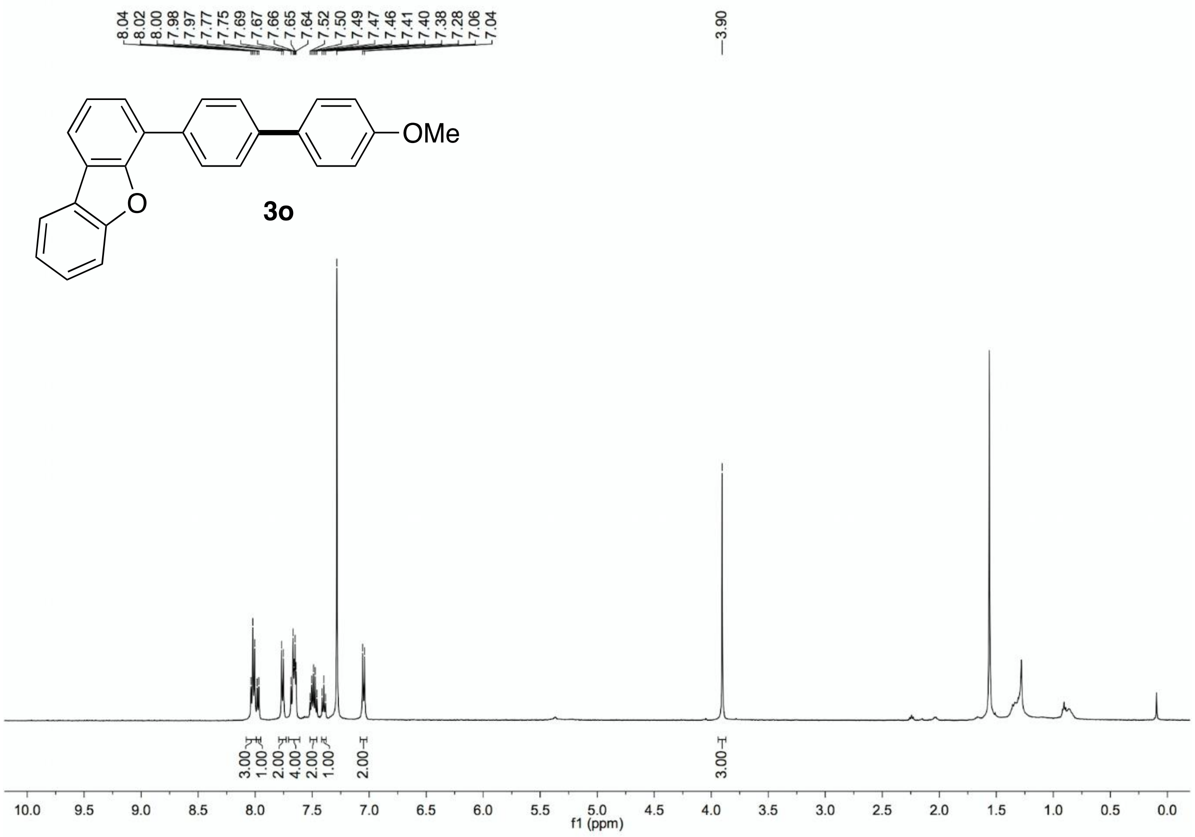

Figure S32. ^{13}C NMR spectrum of **3o**, related to **Figure 3**

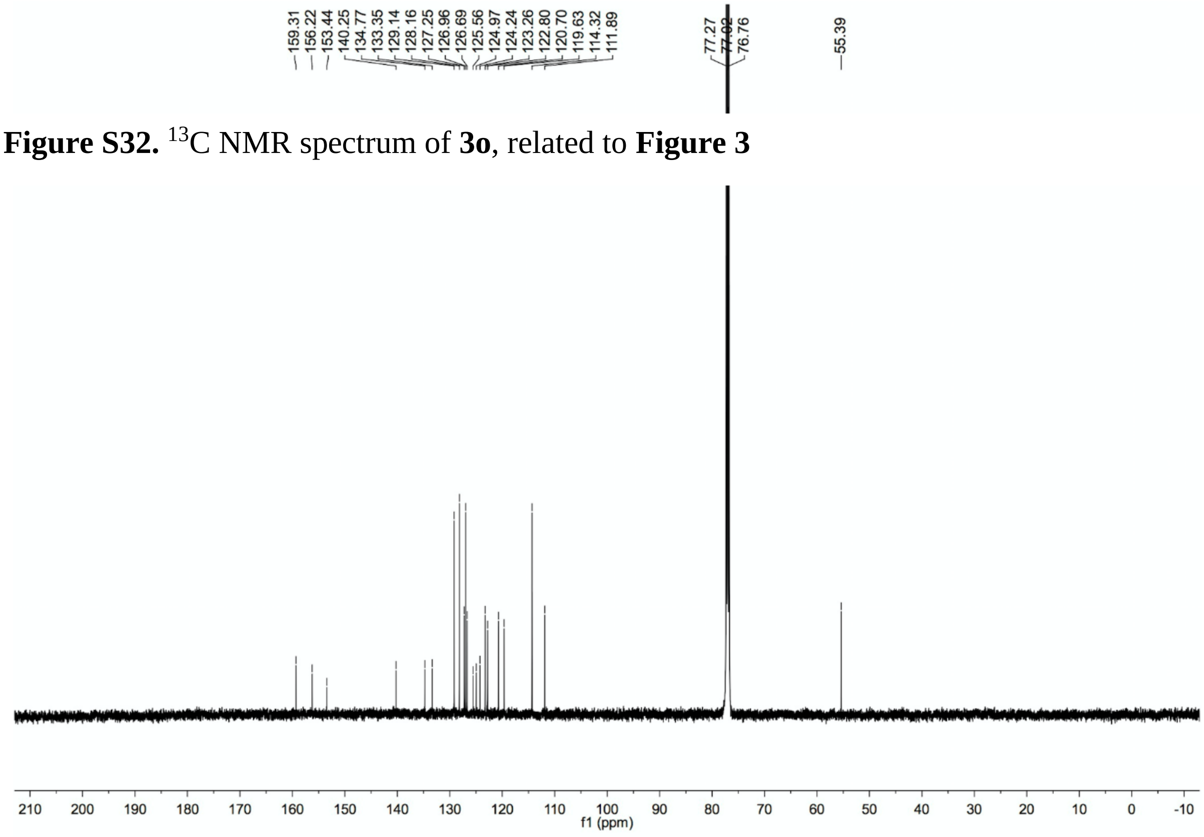

Figure S33. ^{1}H NMR spectrum of **3p**, related to **Figure 3**

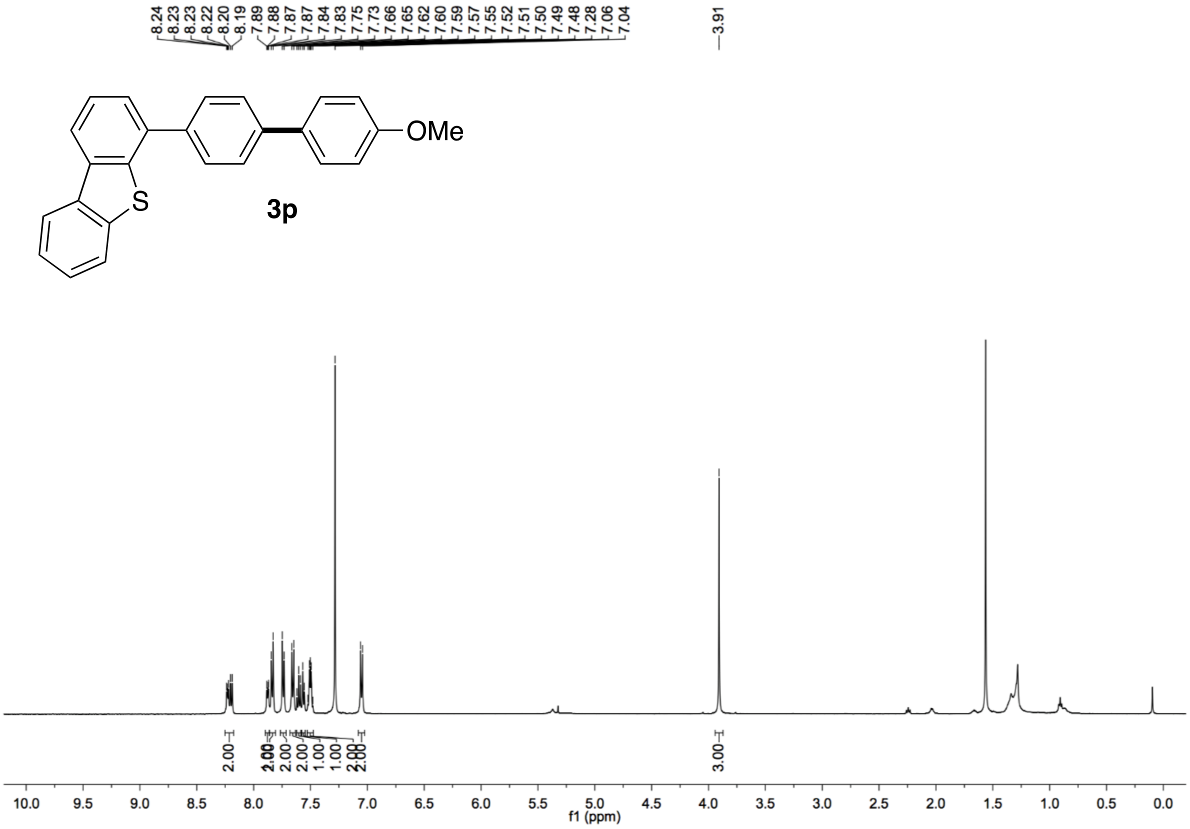

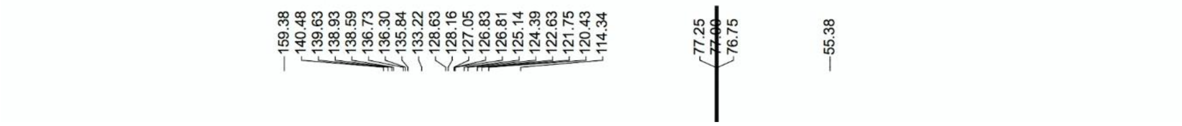

Figure S34. ^{13}C NMR spectrum of **3p**, related to **Figure 3**

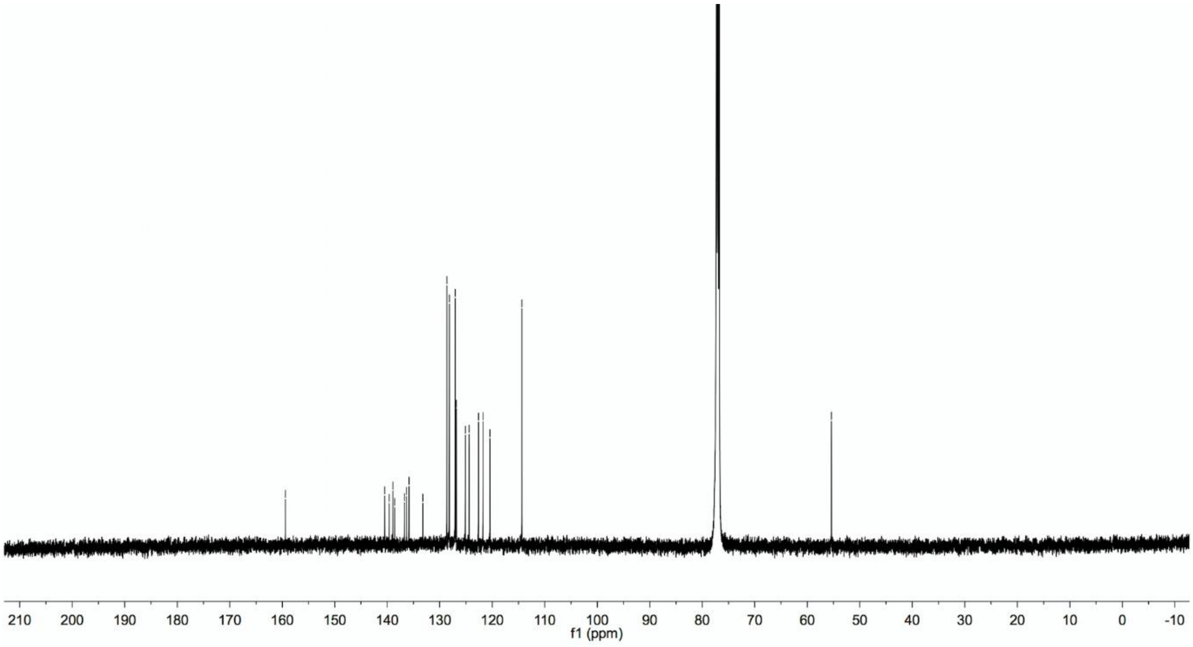

Figure S35. ^{1}H NMR spectrum of **3q**, related to **Figure 3**

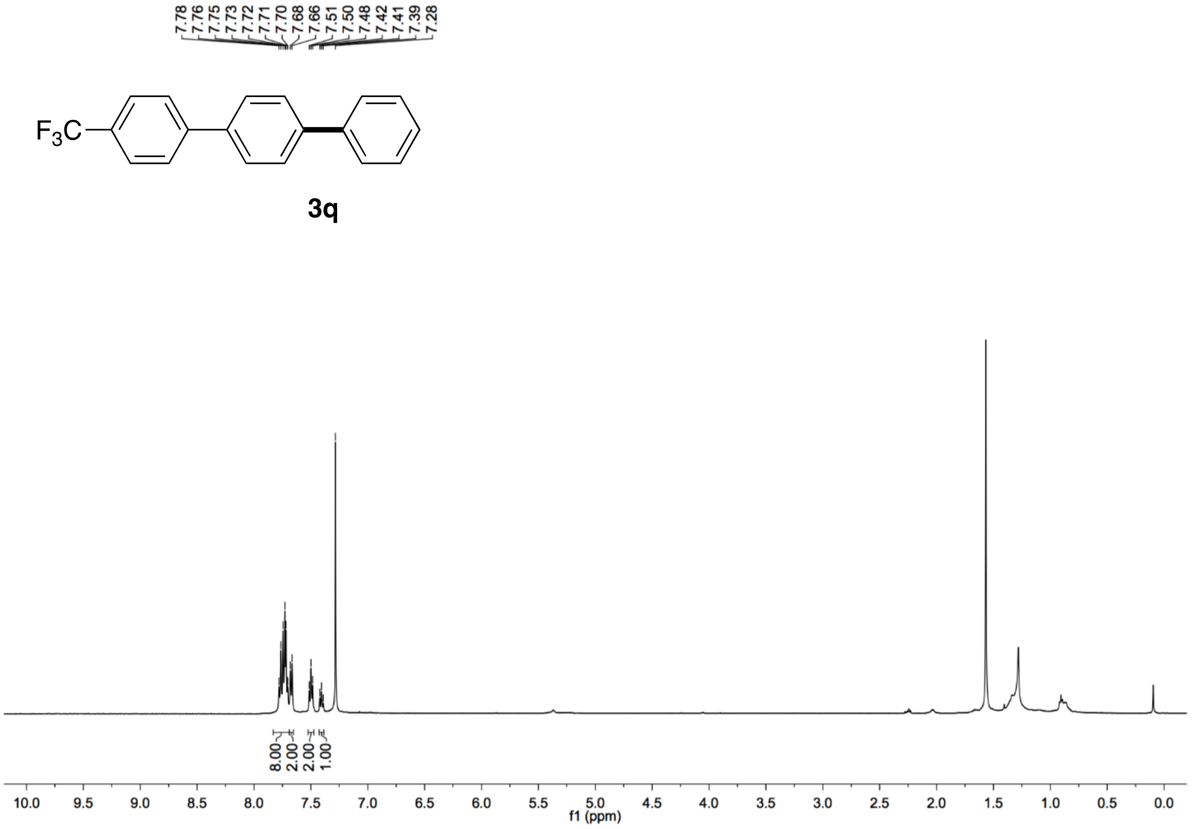

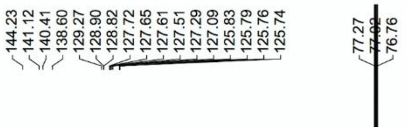

Figure S36. ^{13}C NMR spectrum of **3q**, related to **Figure 3**

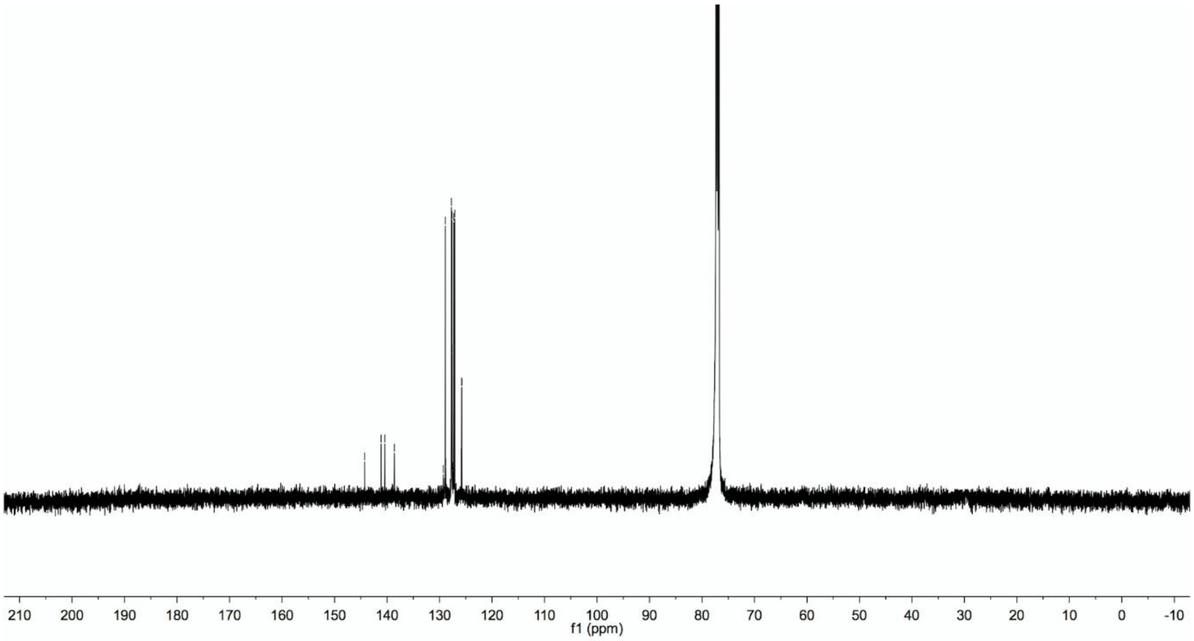

Figure S37. ^{19}F NMR spectrum of **3q**, related to **Figure 3**

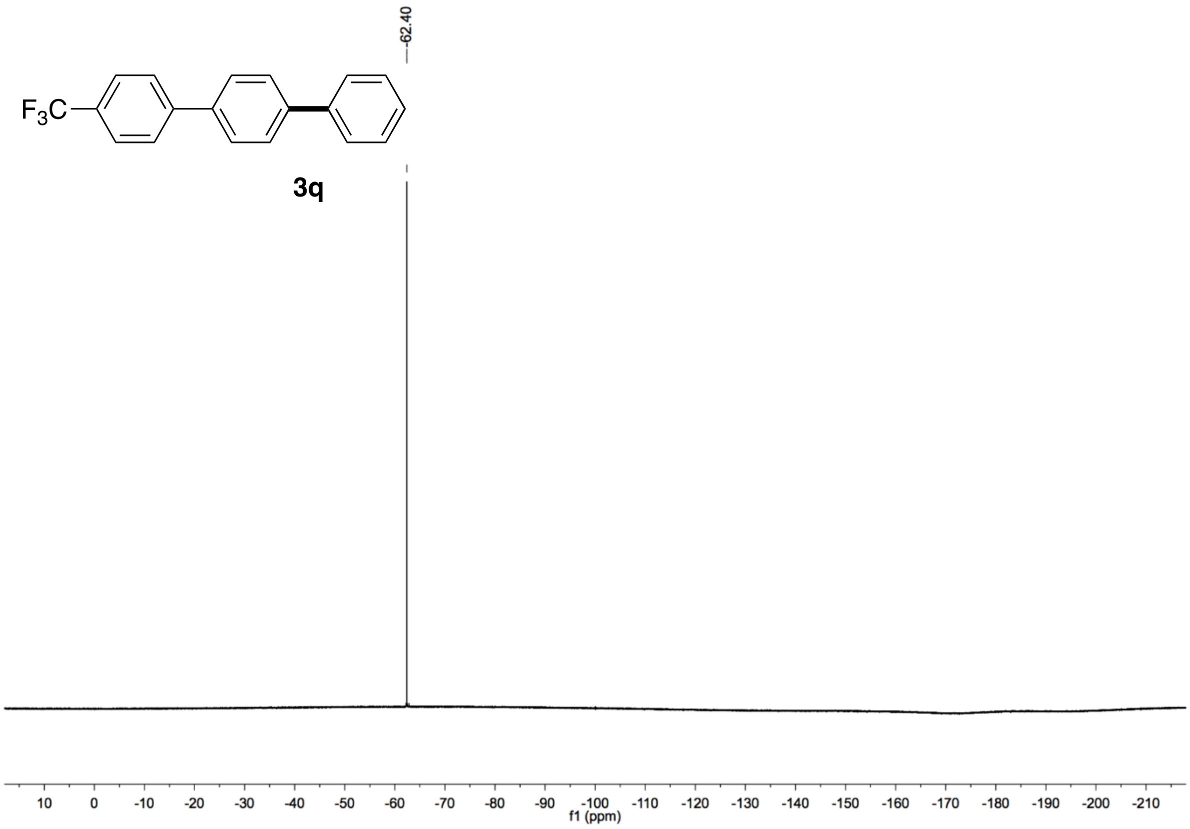

Figure S38. ^{1}H NMR spectrum of **3r**, related to **Figure 3**

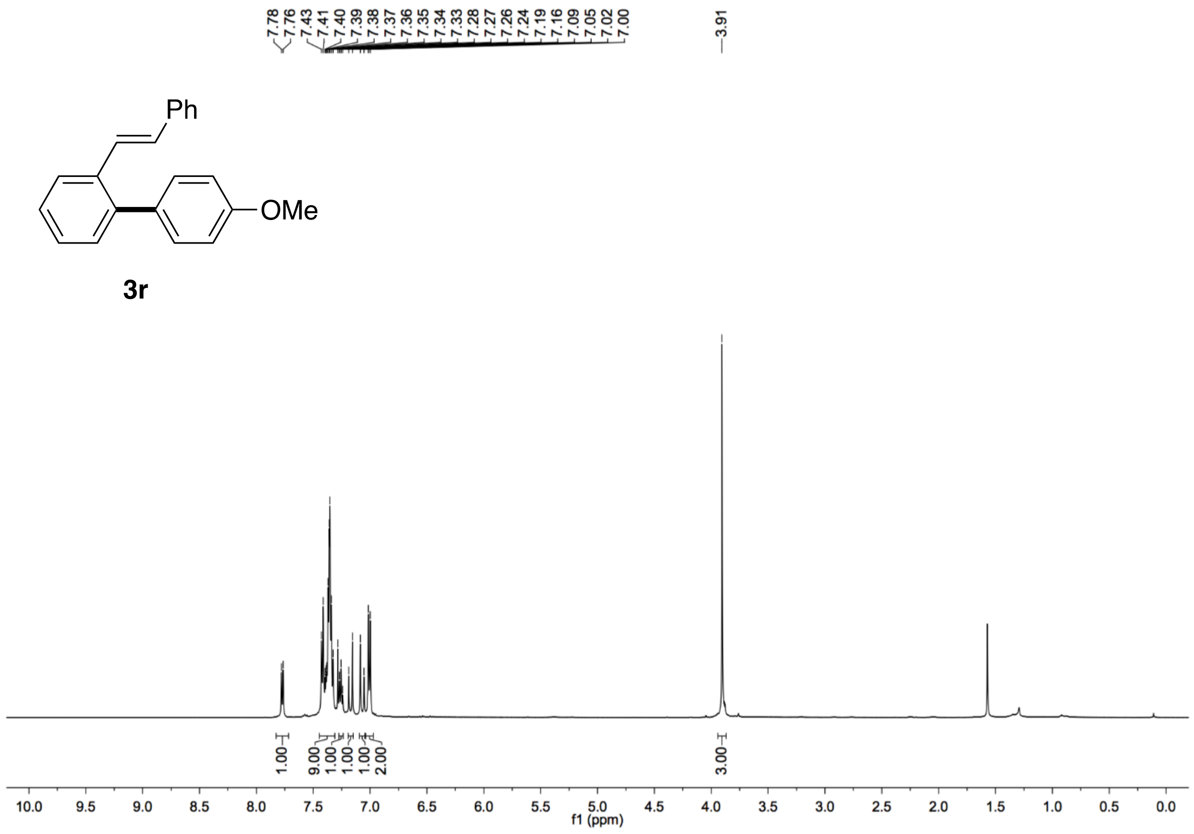

Figure S39. ^{13}C NMR spectrum of **3r**, related to **Figure 3**

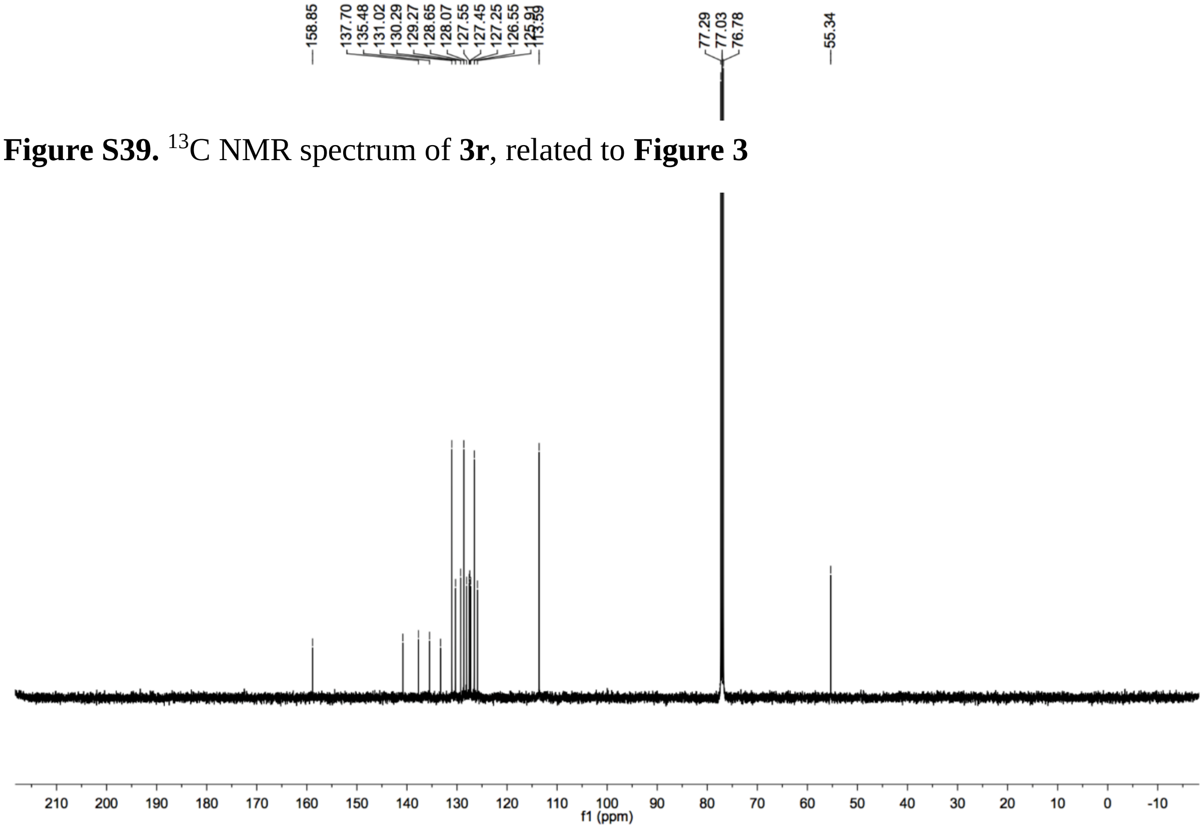

Figure S40. ^{1}H NMR spectrum of **3s**, related to **Figure 3**

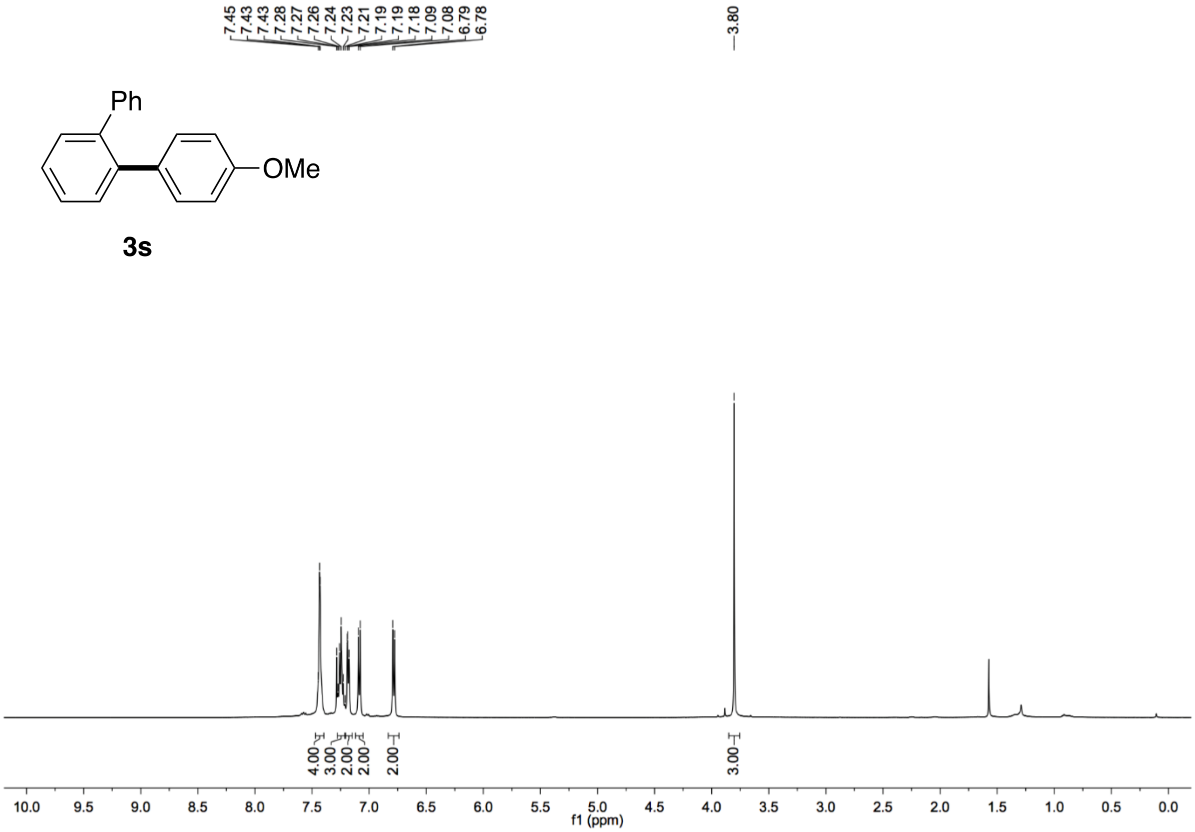

Figure S41. ^{13}C NMR spectrum of **3s**, related to **Figure 3**

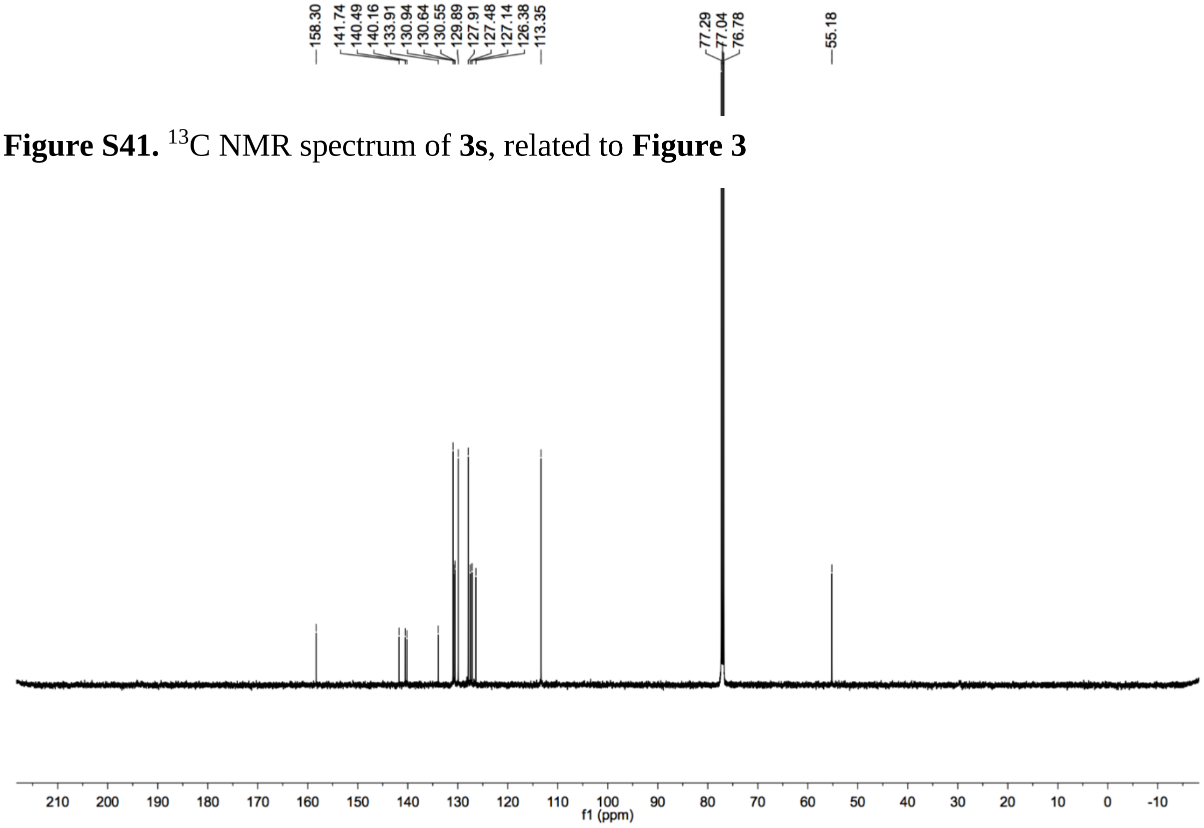

Figure S42. ^{1}H NMR spectrum of **3t**, related to **Figure 3**

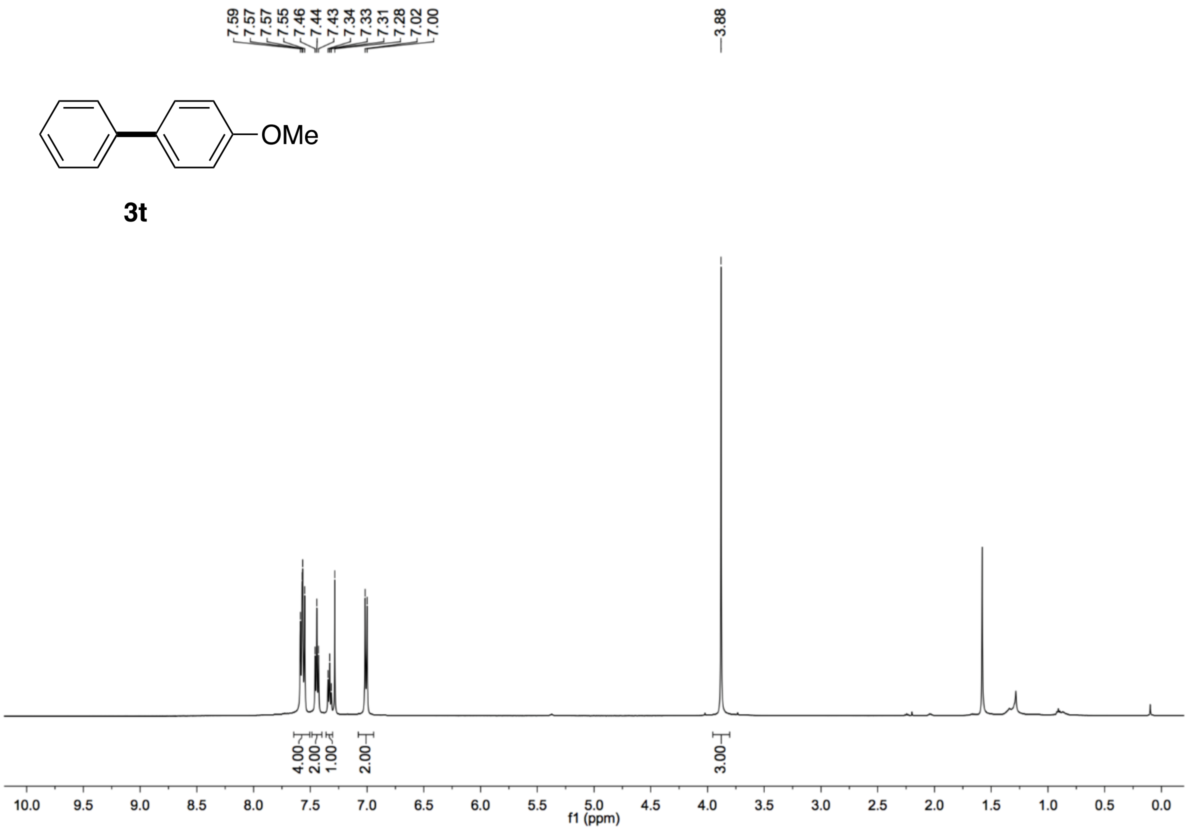

Figure S43. ^{13}C NMR spectrum of **3t**, related to **Figure 3**

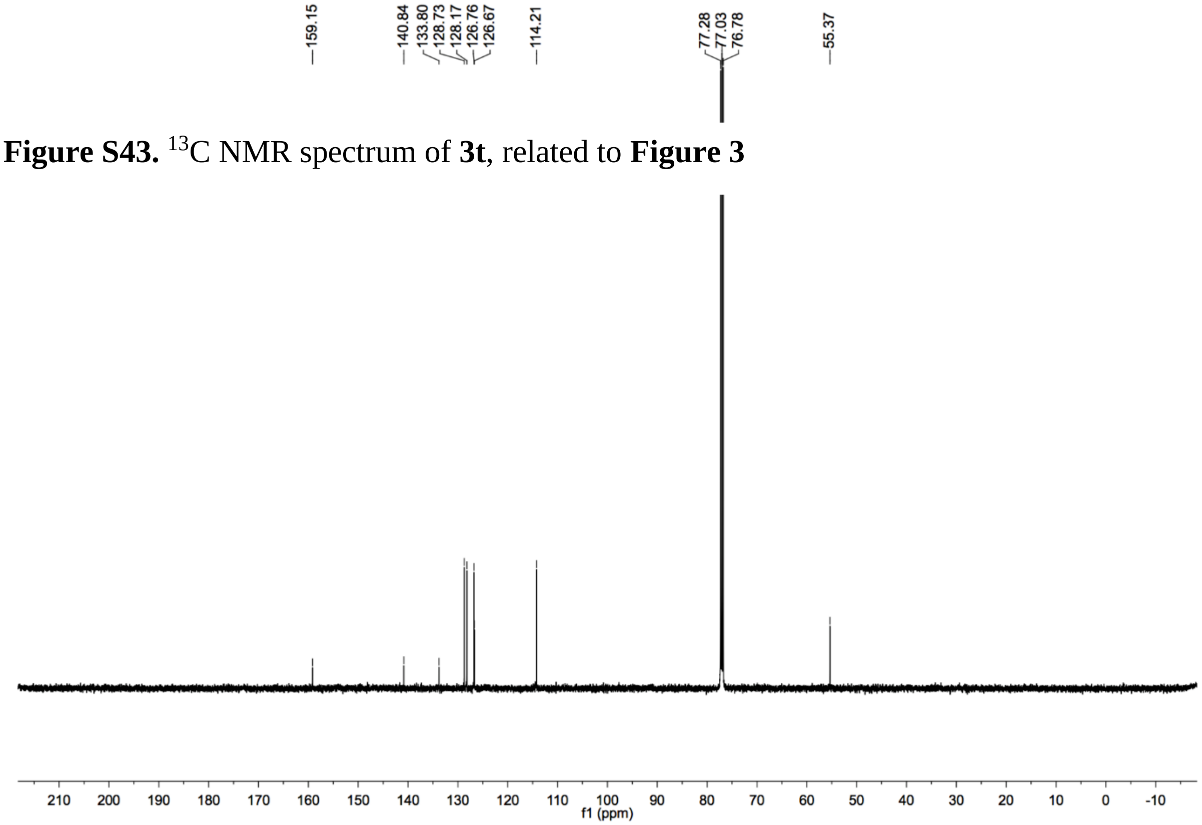

Figure S44. ^{1}H NMR spectrum of **3u**, related to **Figure 3**

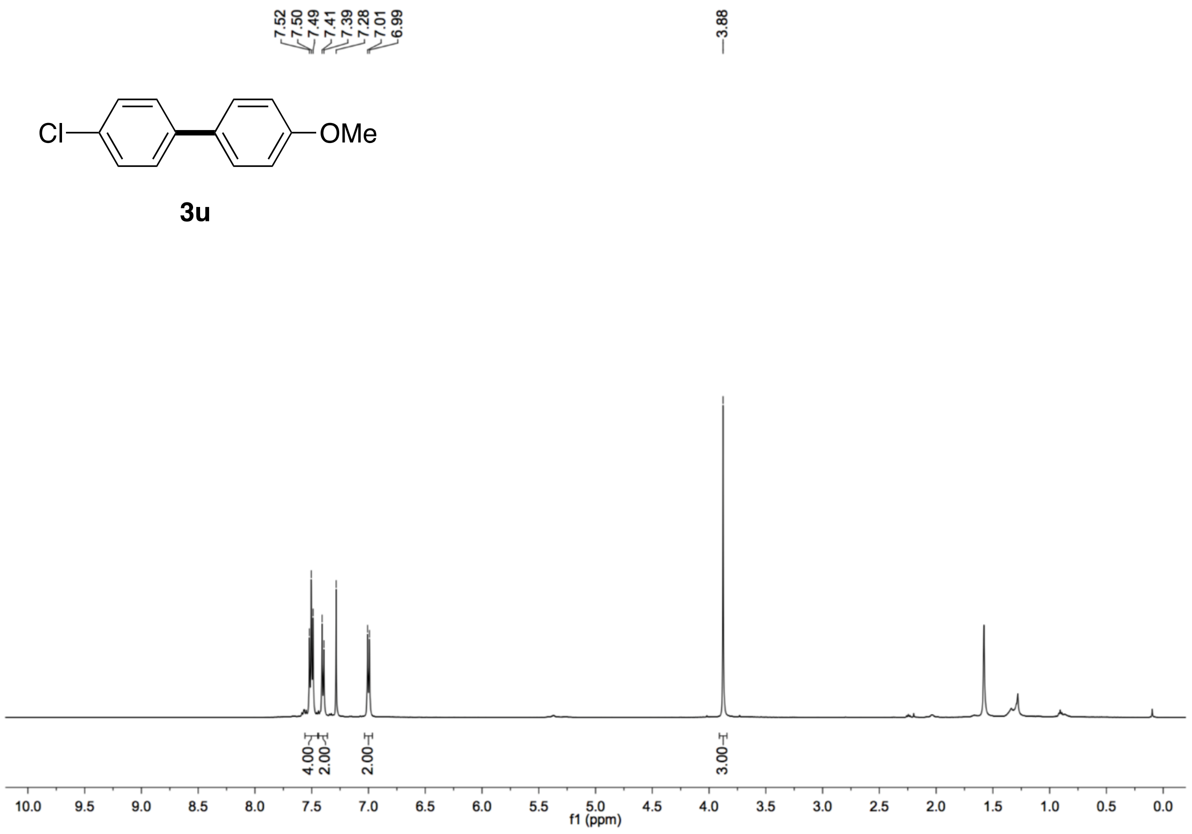

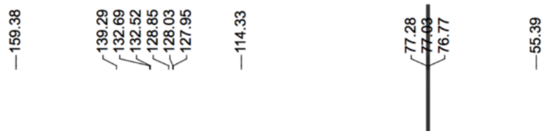

Figure S45. ^{13}C NMR spectrum of **3u**, related to **Figure 3**

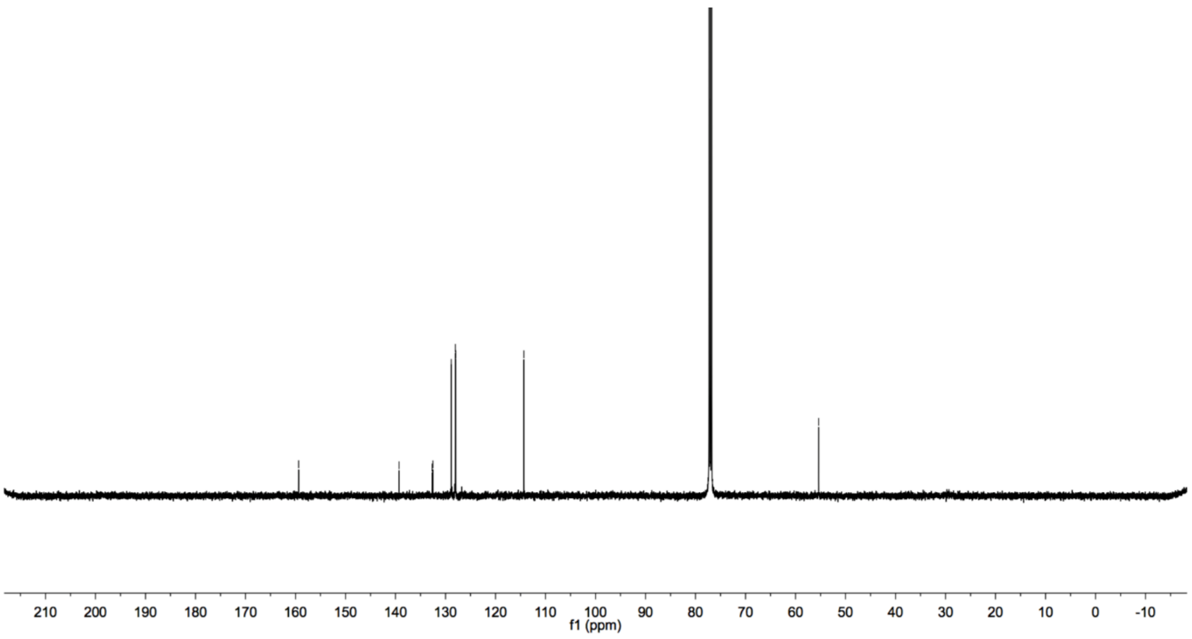

Figure S46. ^{1}H NMR spectrum of **3v**, related to **Figure 3**

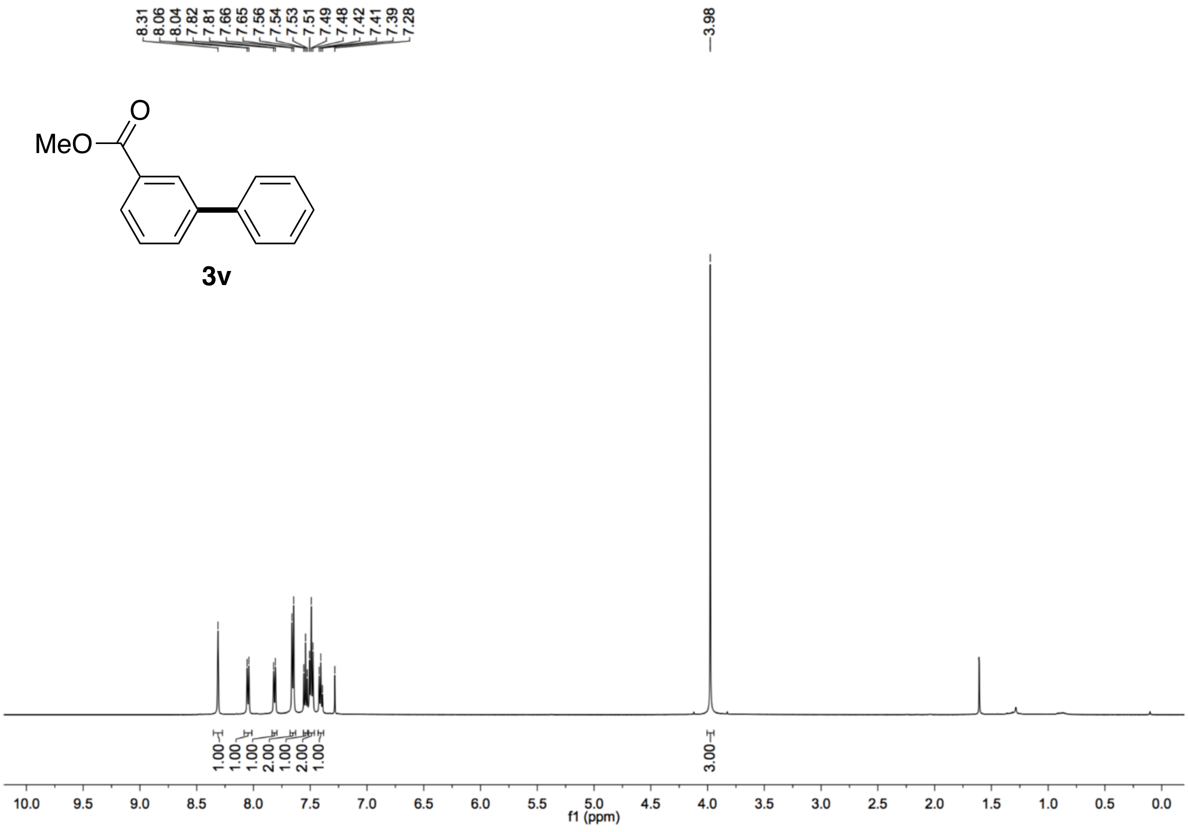

Figure S47. ^{13}C NMR spectrum of **3v**, related to **Figure 3**

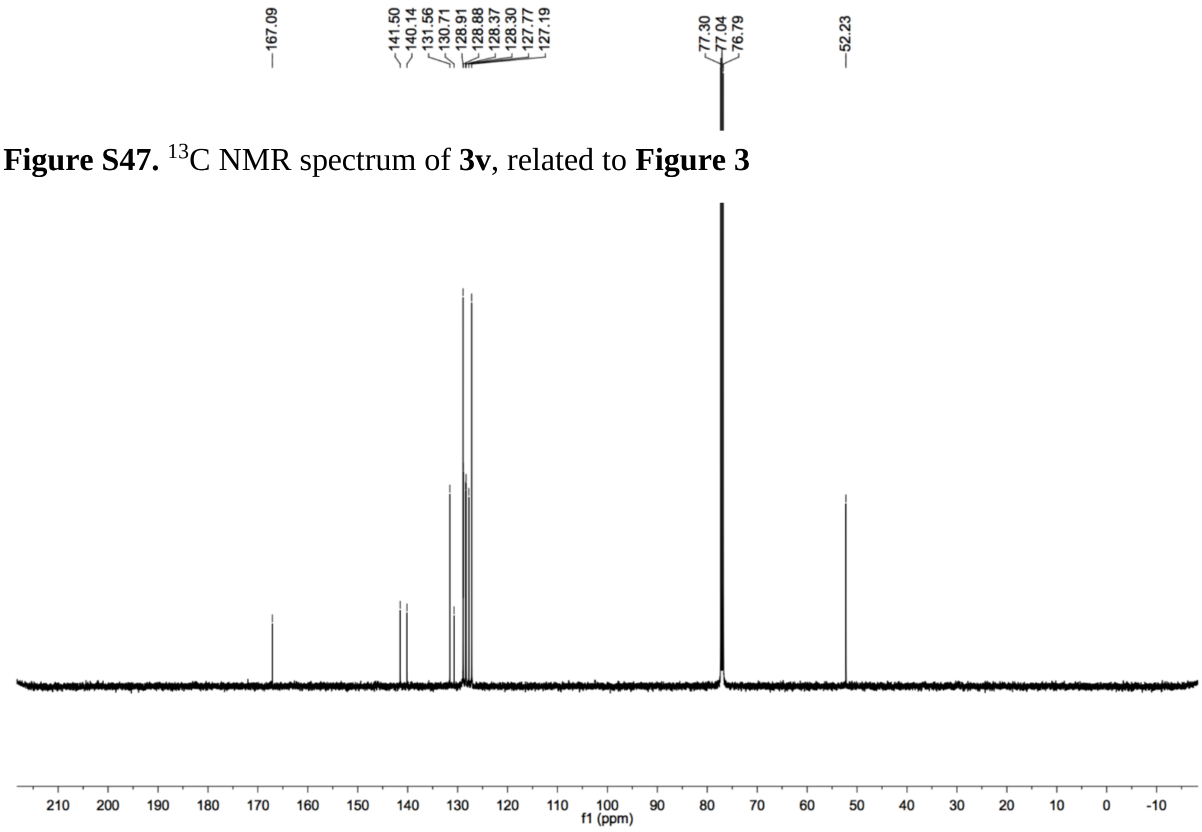

Figure S48. ^{1}H NMR spectrum of **3w**, related to **Figure 3**

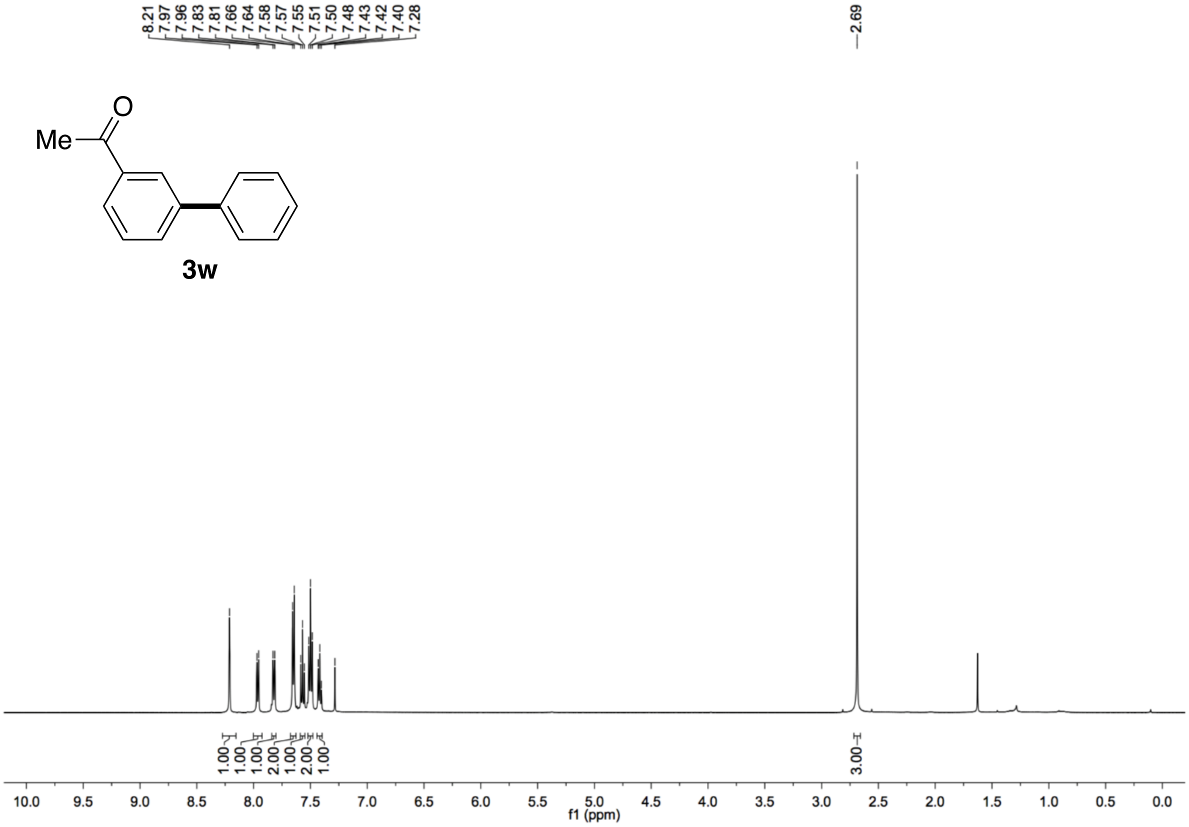

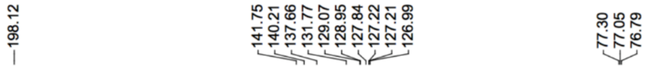

Figure S49. ^{13}C NMR spectrum of **3w**, related to **Figure 3**

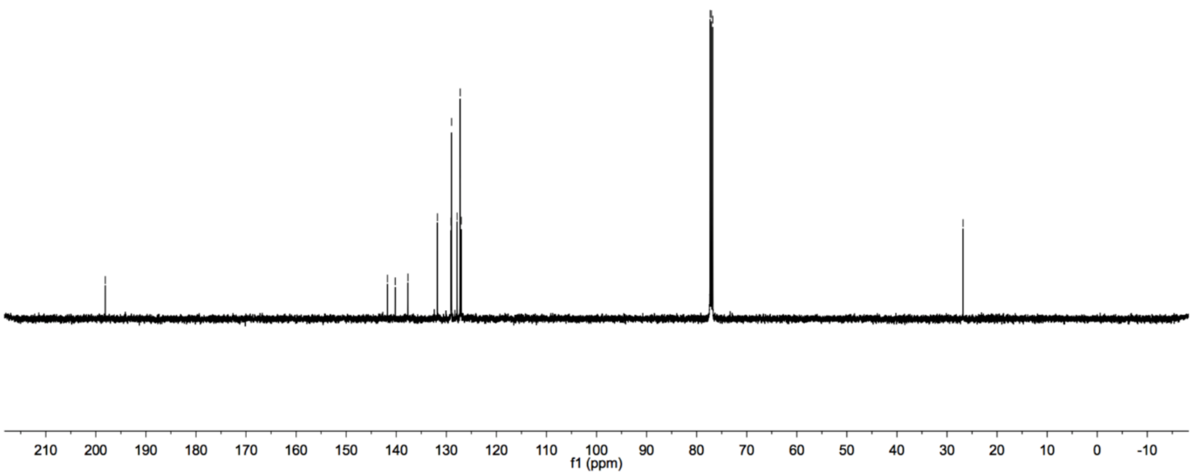

Figure S50. ^{1}H NMR spectrum of **3x**, related to **Figure 3**

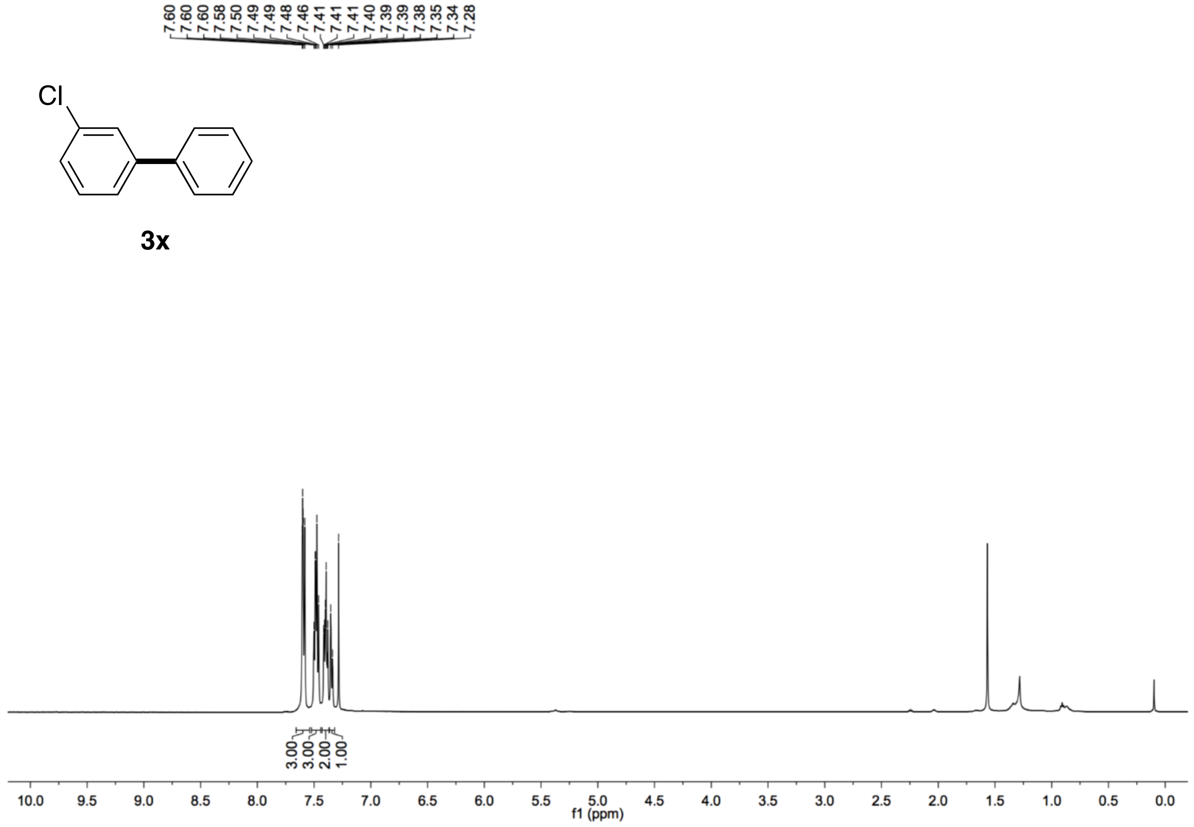

Figure S51. ^{13}C NMR spectrum of **3x**, related to **Figure 3**

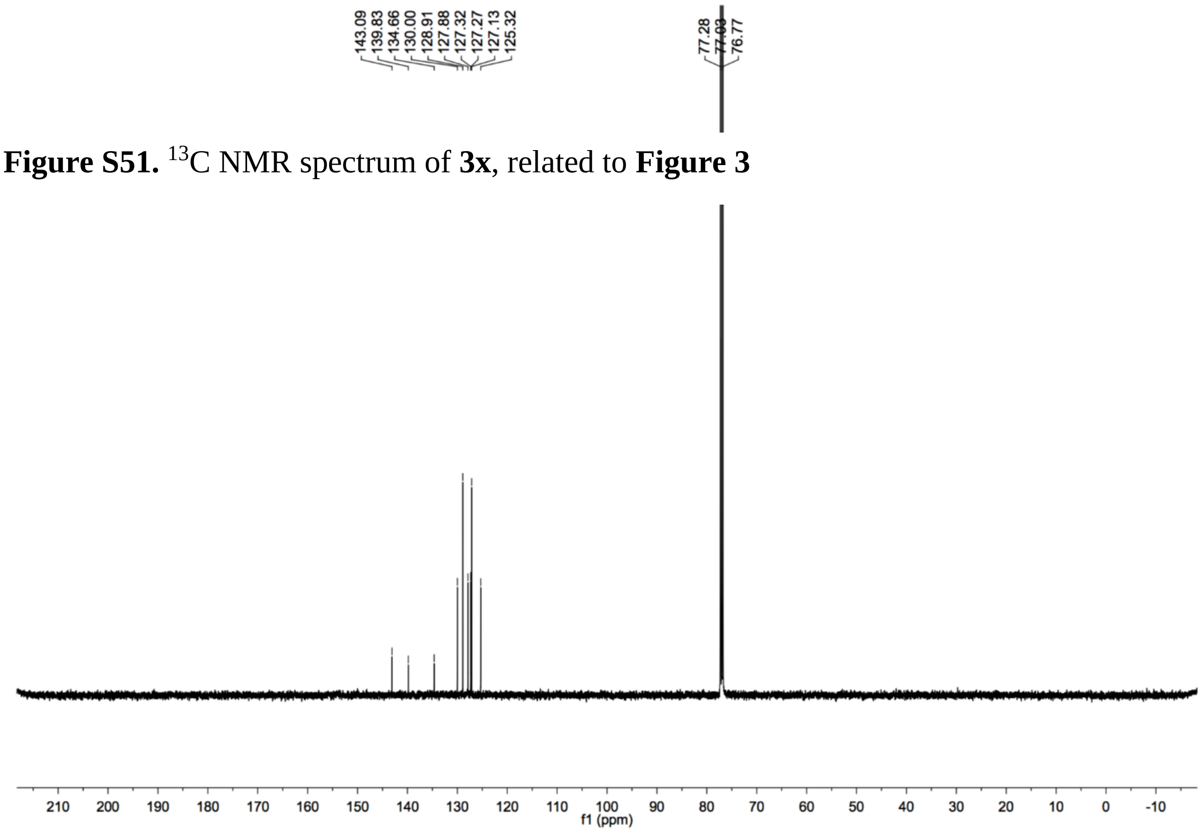

Figure S52. ^{1}H NMR spectrum of **3y**, related to **Figure 3**

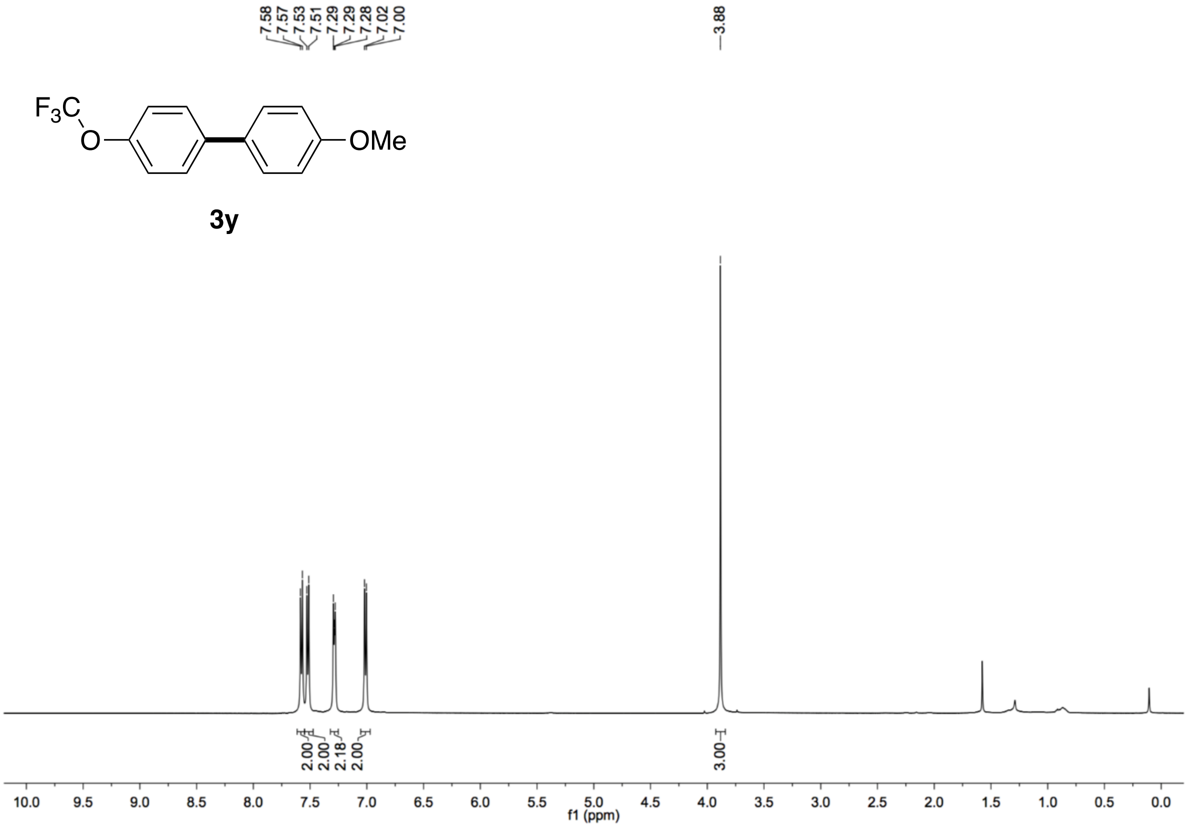

Figure S53. ^{13}C NMR spectrum of **3y**, related to **Figure 3**

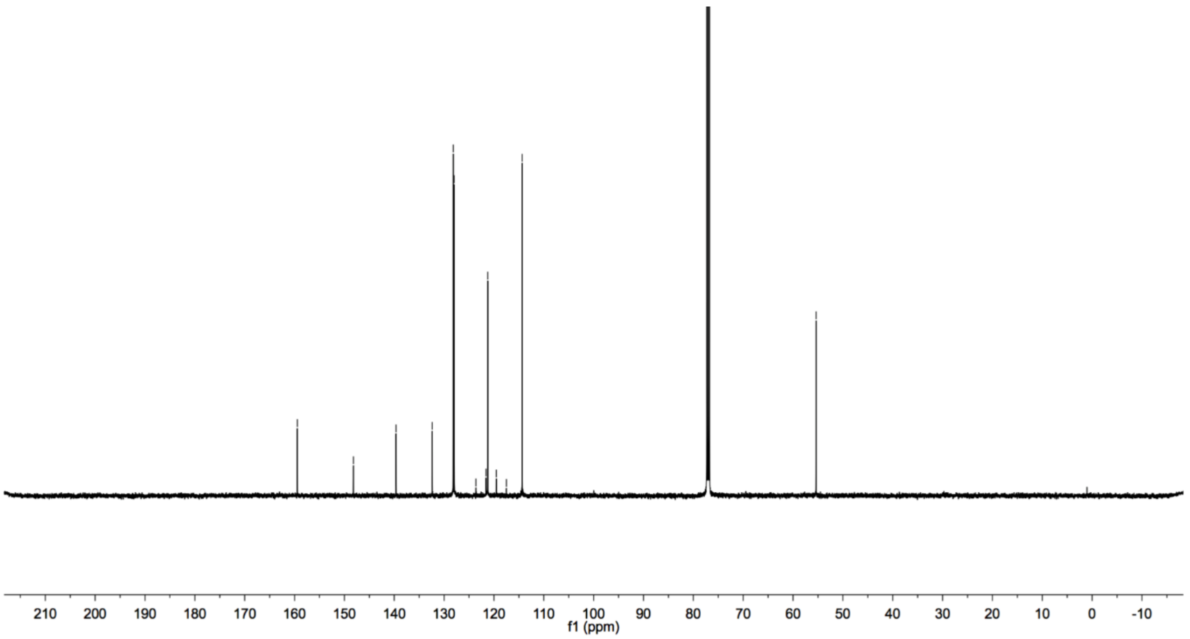

Figure S54. ^{19}F NMR spectrum of **3y**, related to **Figure 3**

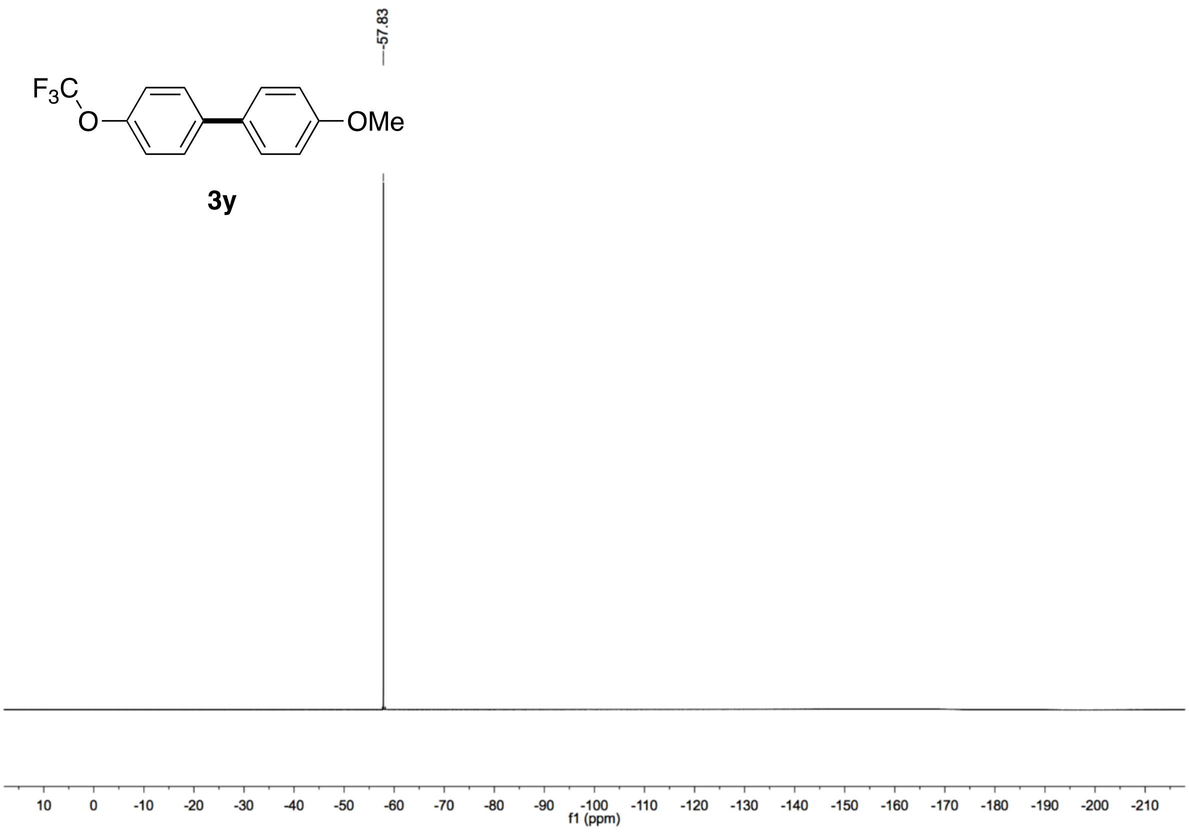

Figure S55. ^{1}H NMR spectrum of **3z**, related to **Figure 3**

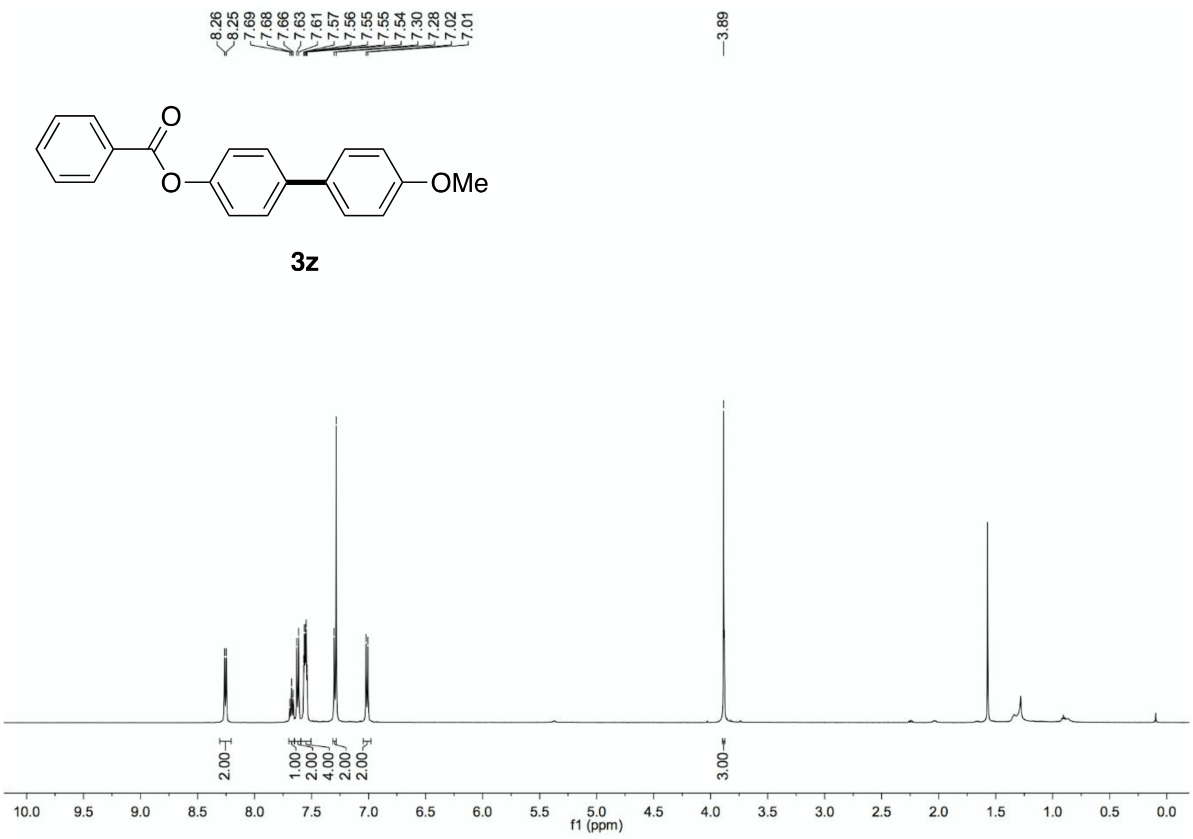

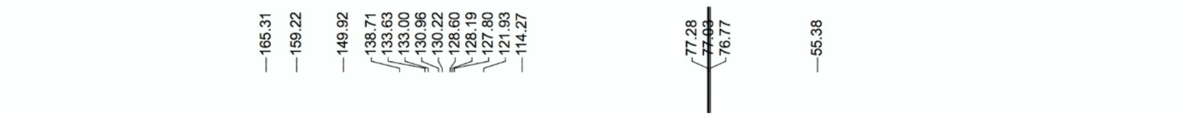

Figure S56. ^{13}C NMR spectrum of **3z**, related to **Figure 3**

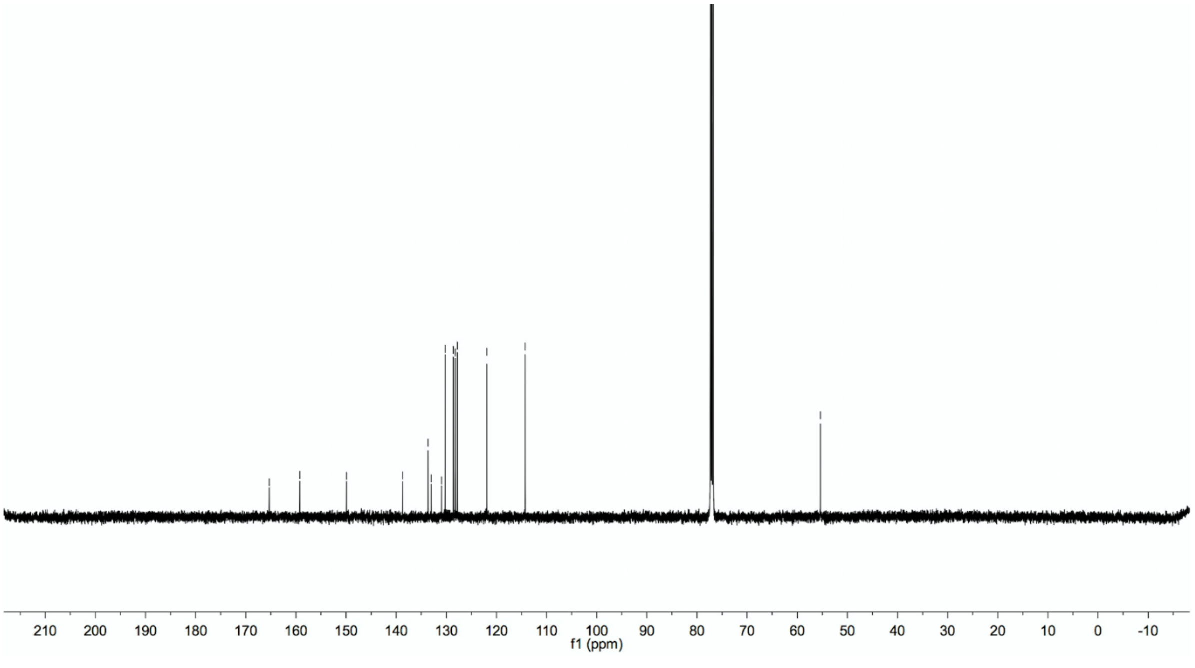

Figure S57. ^{1}H NMR spectrum of **3aa**, related to **Figure 3**

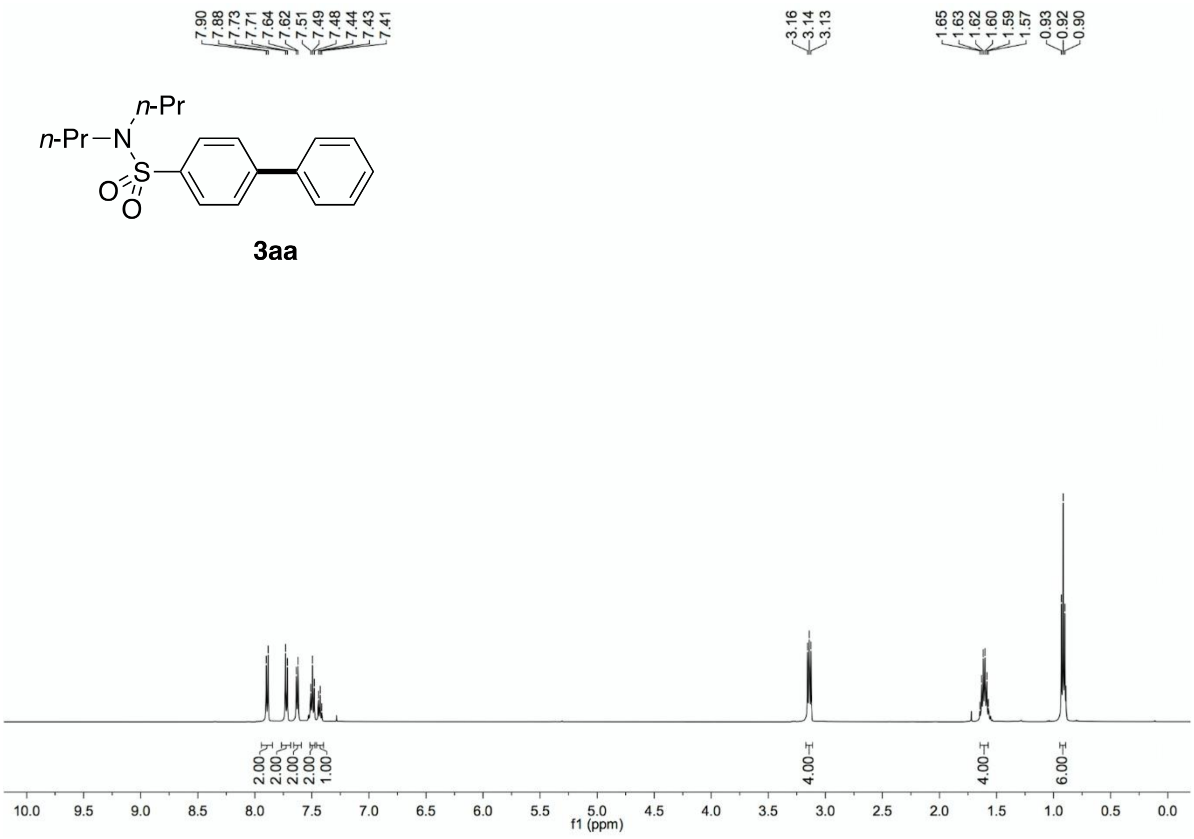

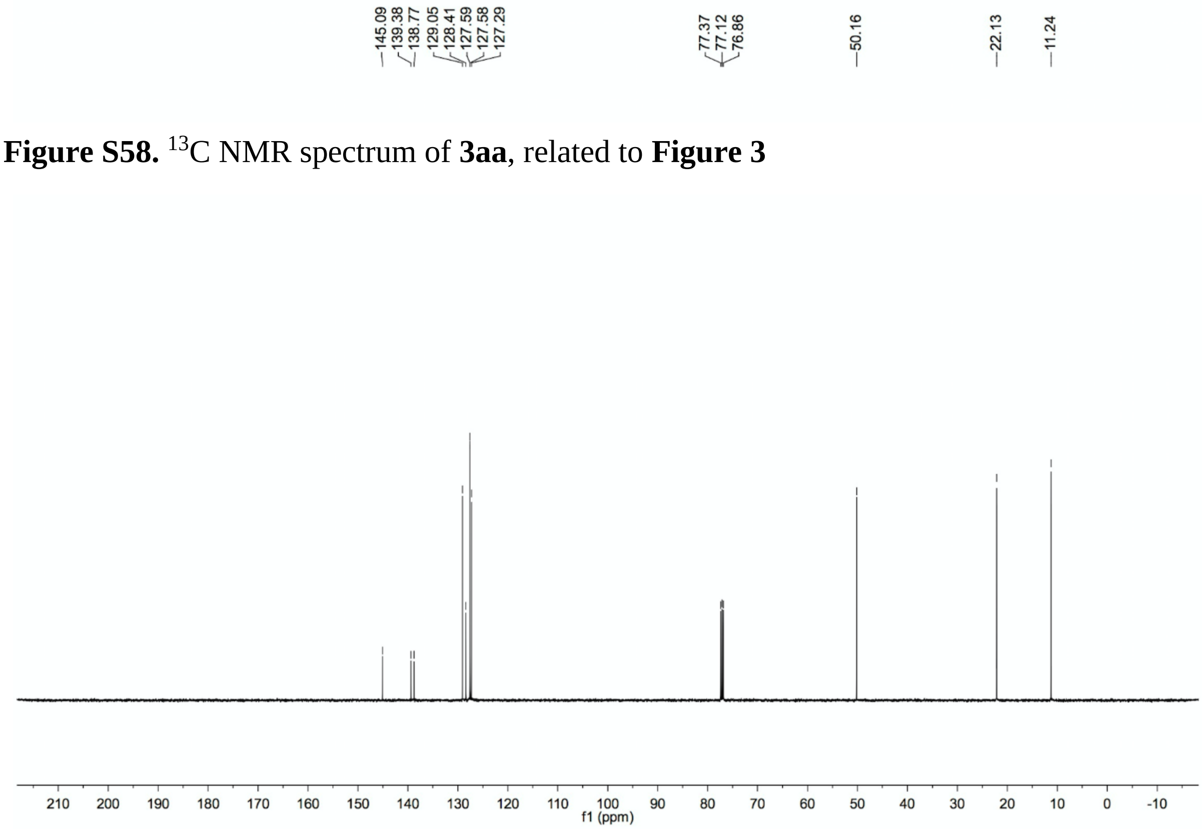

Figure S58. ^{13}C NMR spectrum of **3aa**, related to **Figure 3**

Figure S59. ^{1}H NMR spectrum of **3ab**, related to **Figure 3**

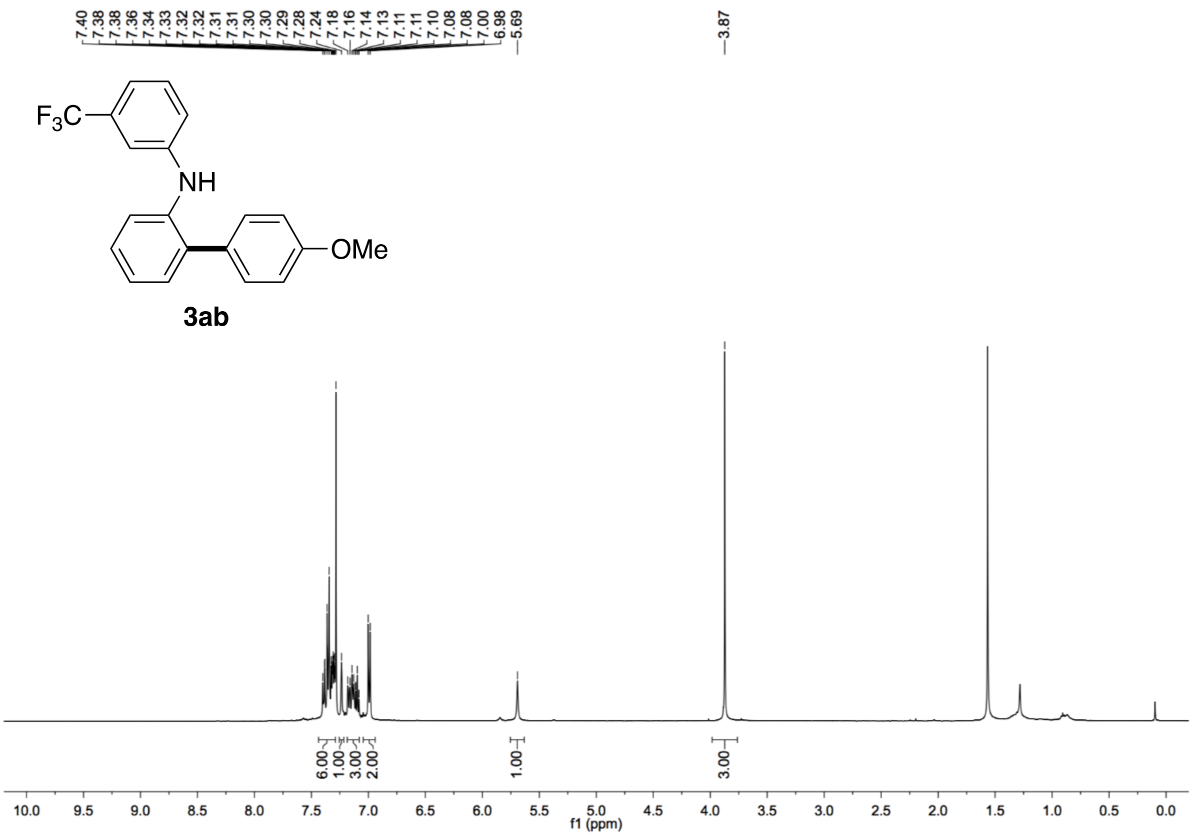

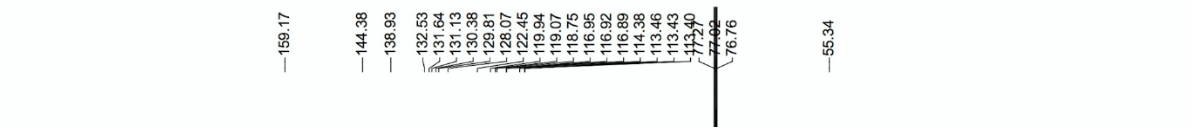

Figure S60. ^{13}C NMR spectrum of **3ab**, related to **Figure 3**

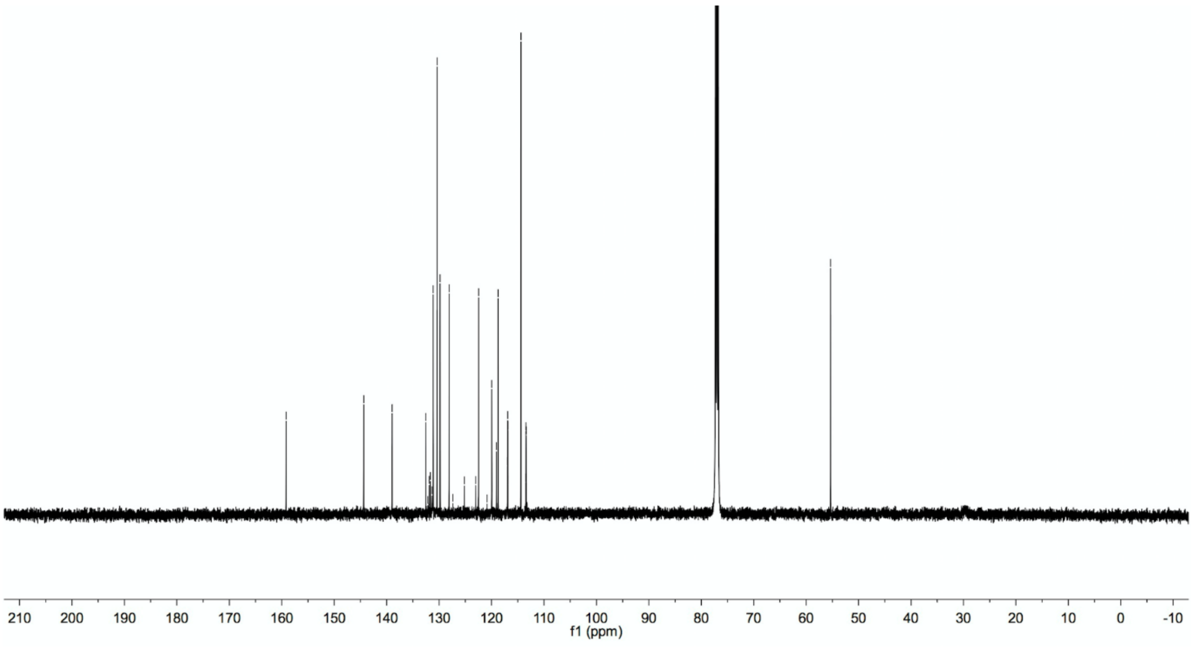

Figure S61. ^{19}F NMR spectrum of **3ab**, related to **Figure 3**

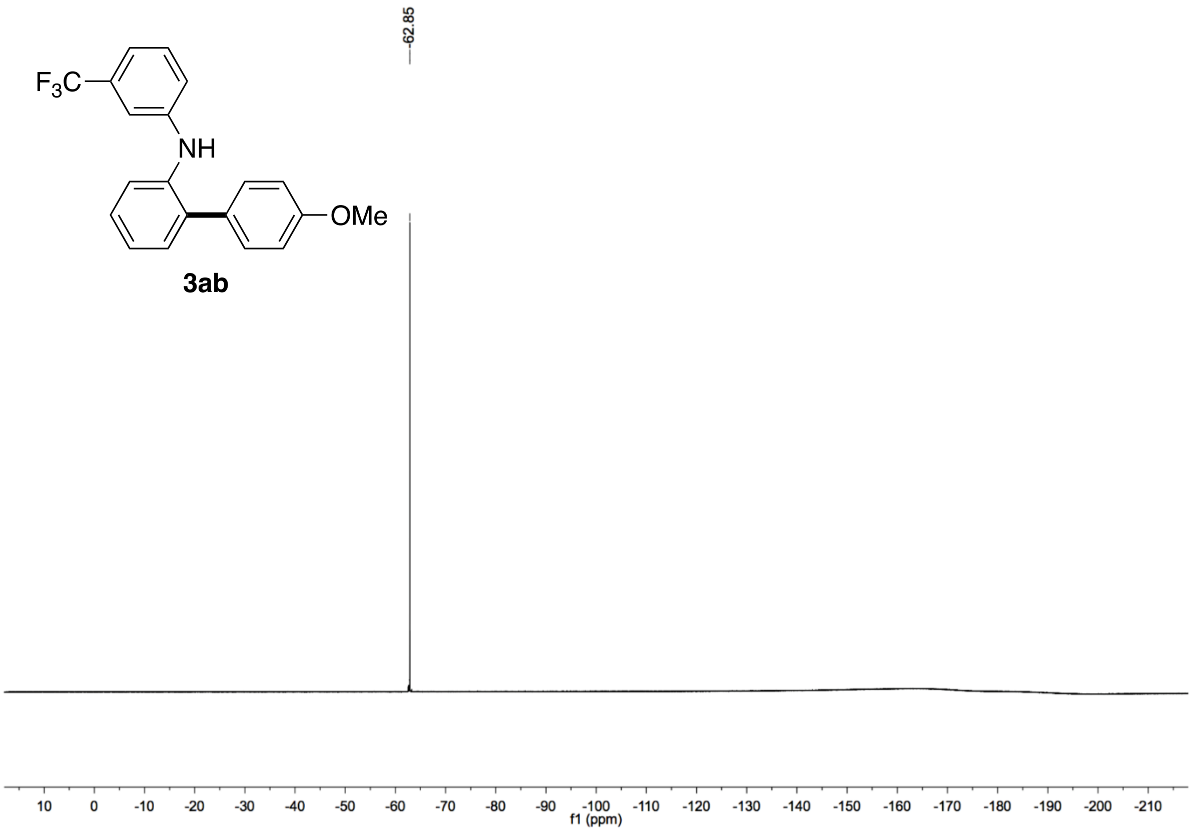

Figure S62. ^{1}H NMR spectrum of **3ac**, related to **Figure 3**

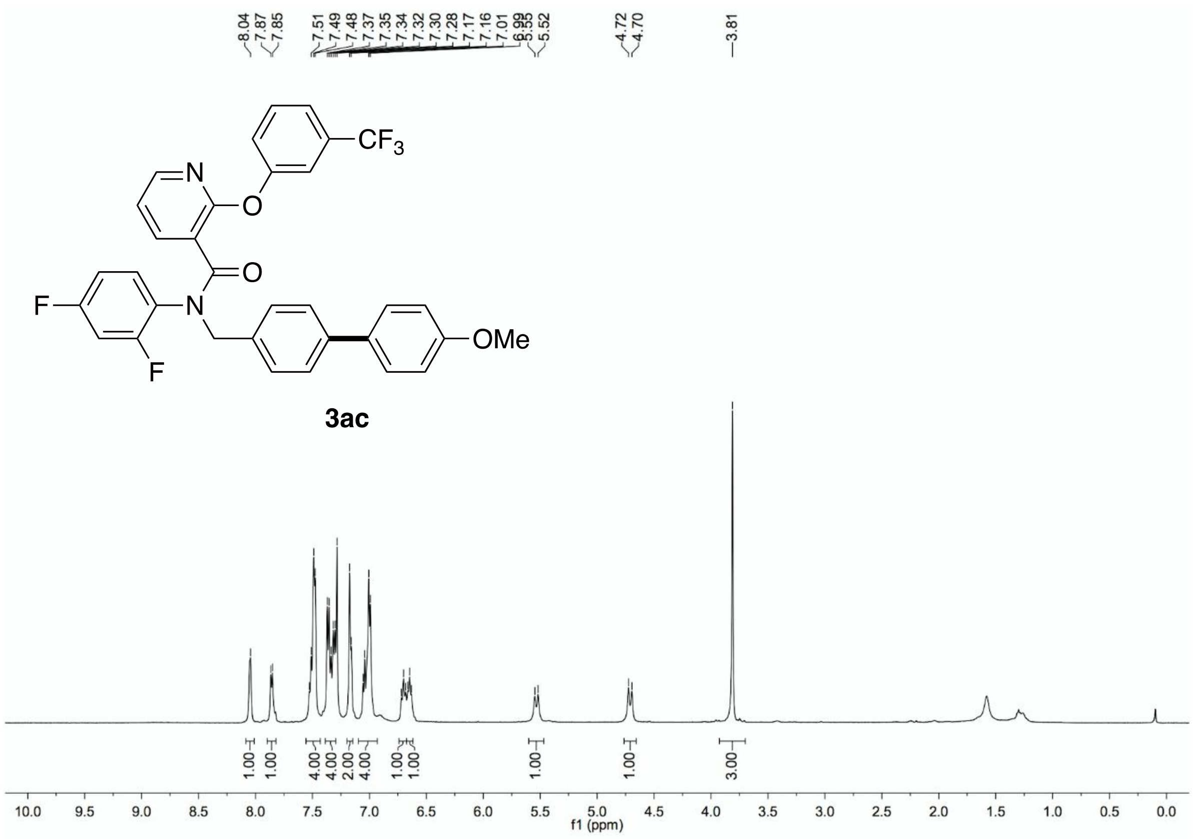

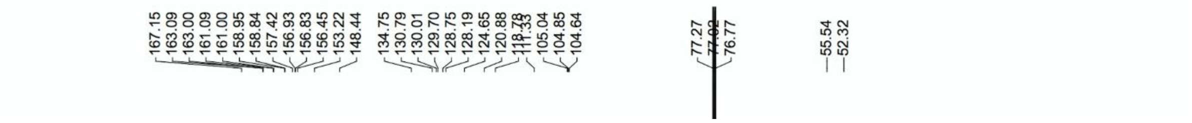

Figure S63. ^{13}C NMR spectrum of **3ac**, related to **Figure 3**

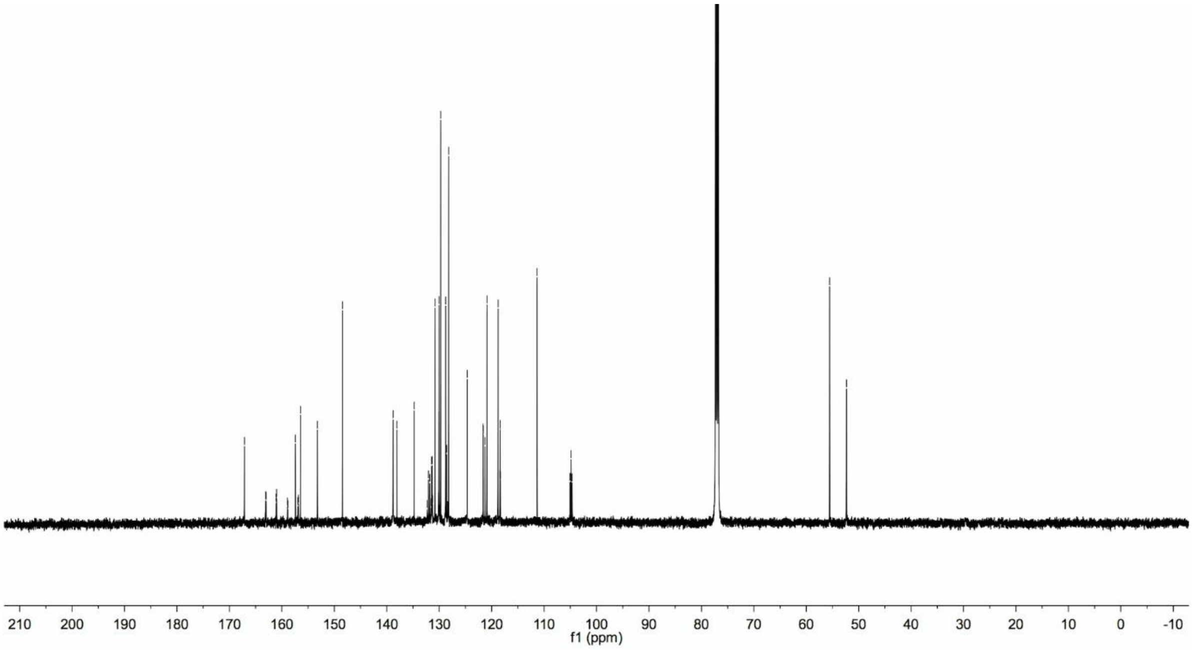

Figure S64. ^{19}F NMR spectrum of **3ac**, related to **Figure 3**

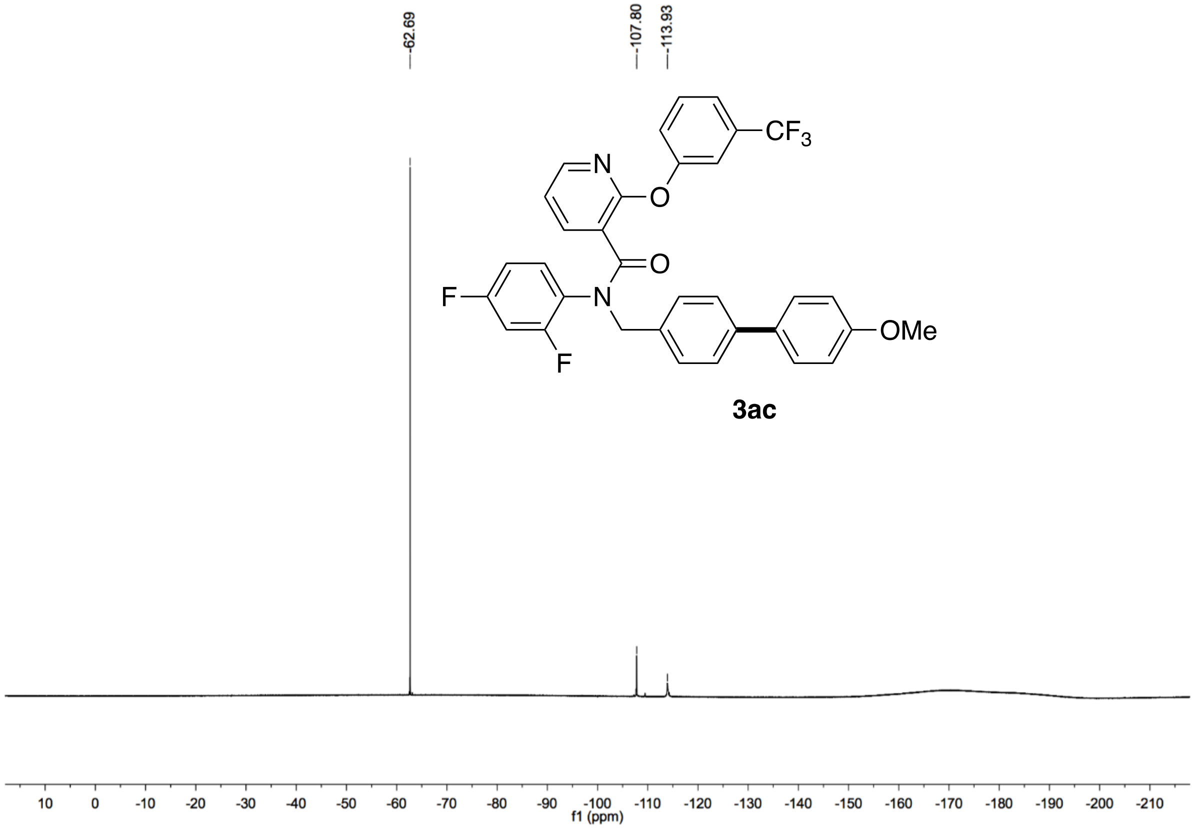

Figure S65. ^{1}H NMR spectrum of **3ad**, related to **Figure 3**

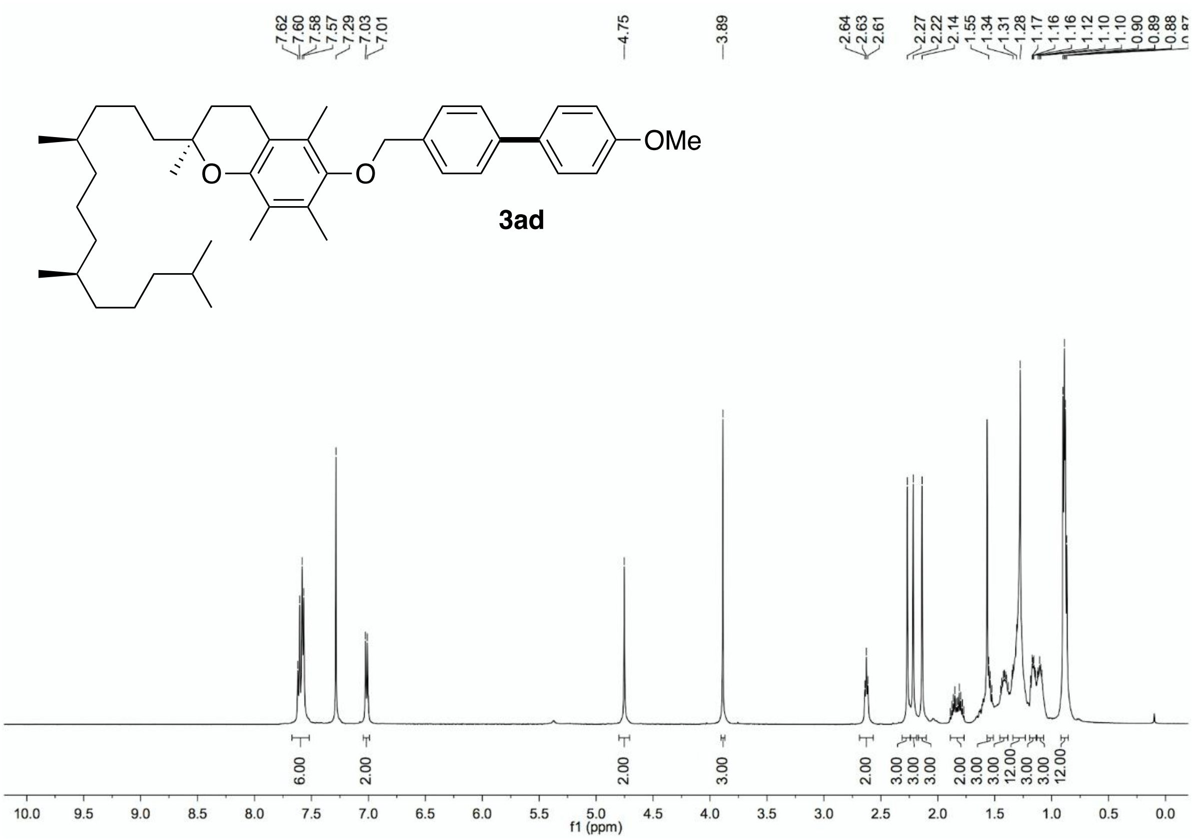

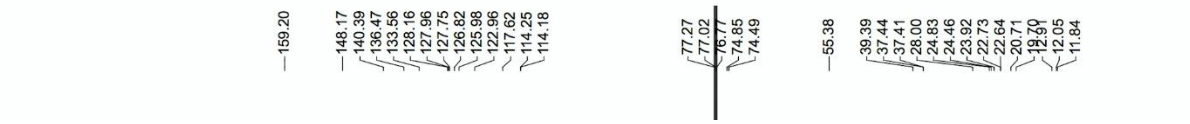

Figure S66. ^{13}C NMR spectrum of **3ad**, related to **Figure 3**

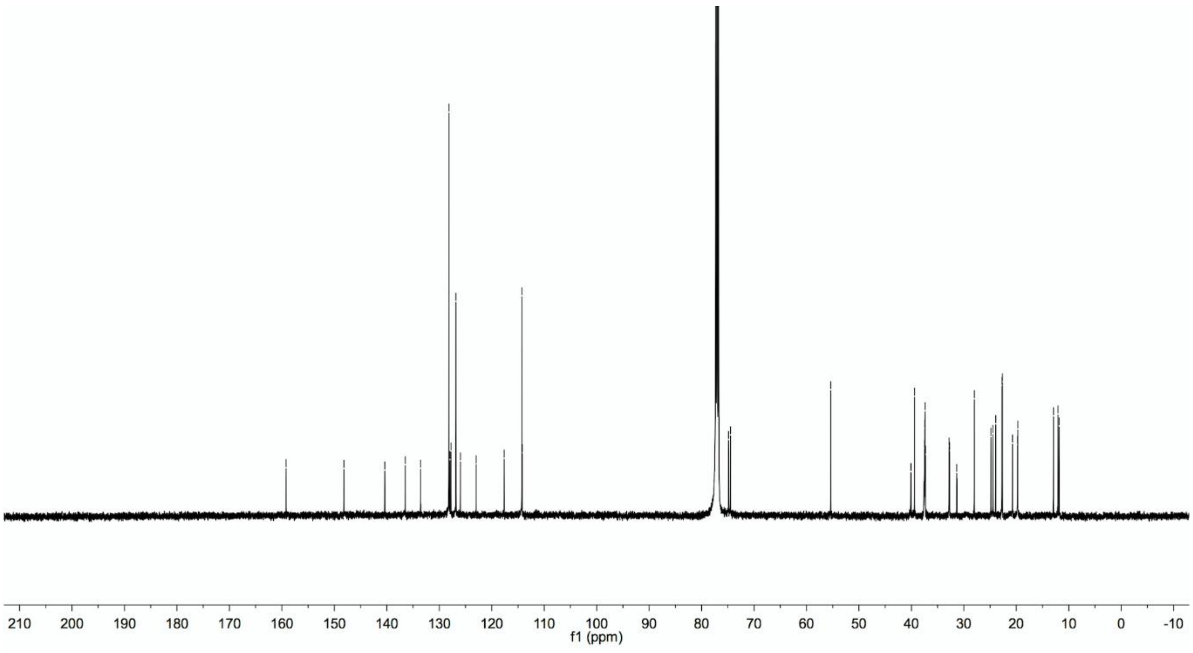

Figure S67. ^{1}H NMR spectrum of **3ae**, related to **Figure 3**

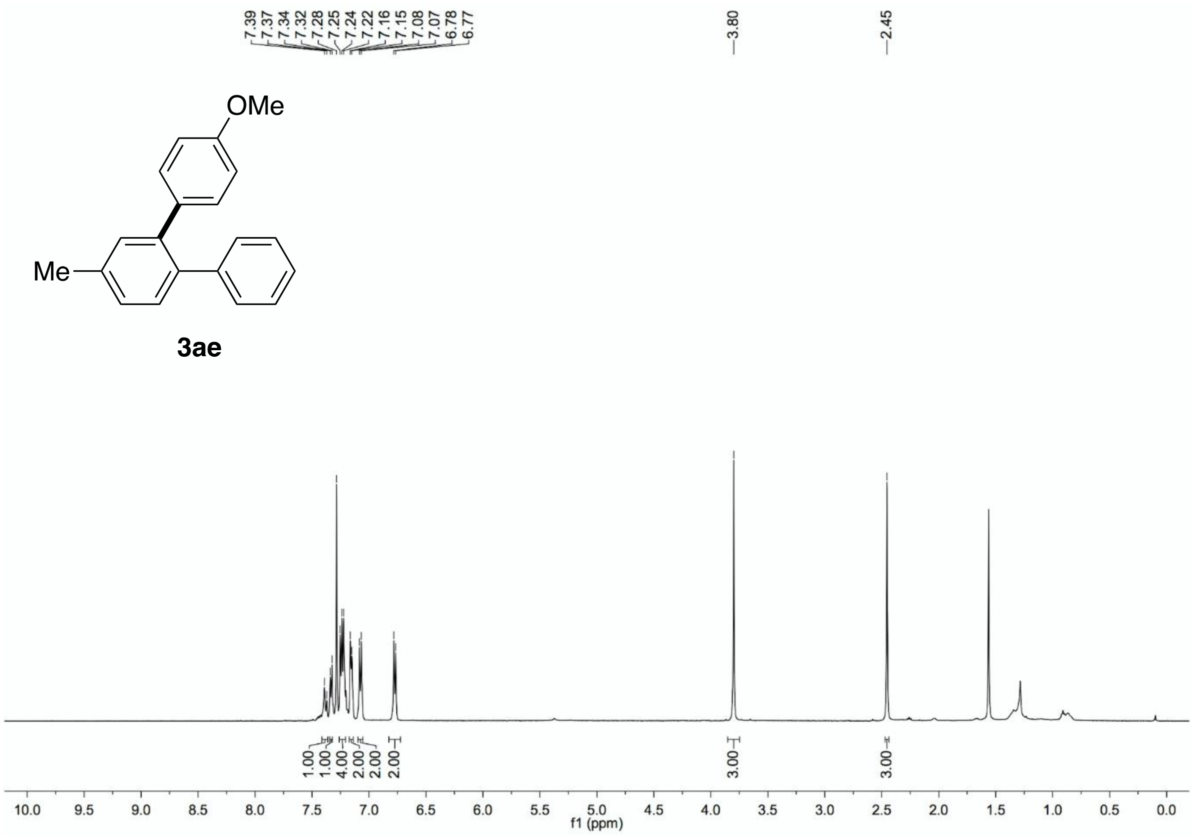

Figure S68. ^{13}C NMR spectrum of **3ae**, related to **Figure 3**

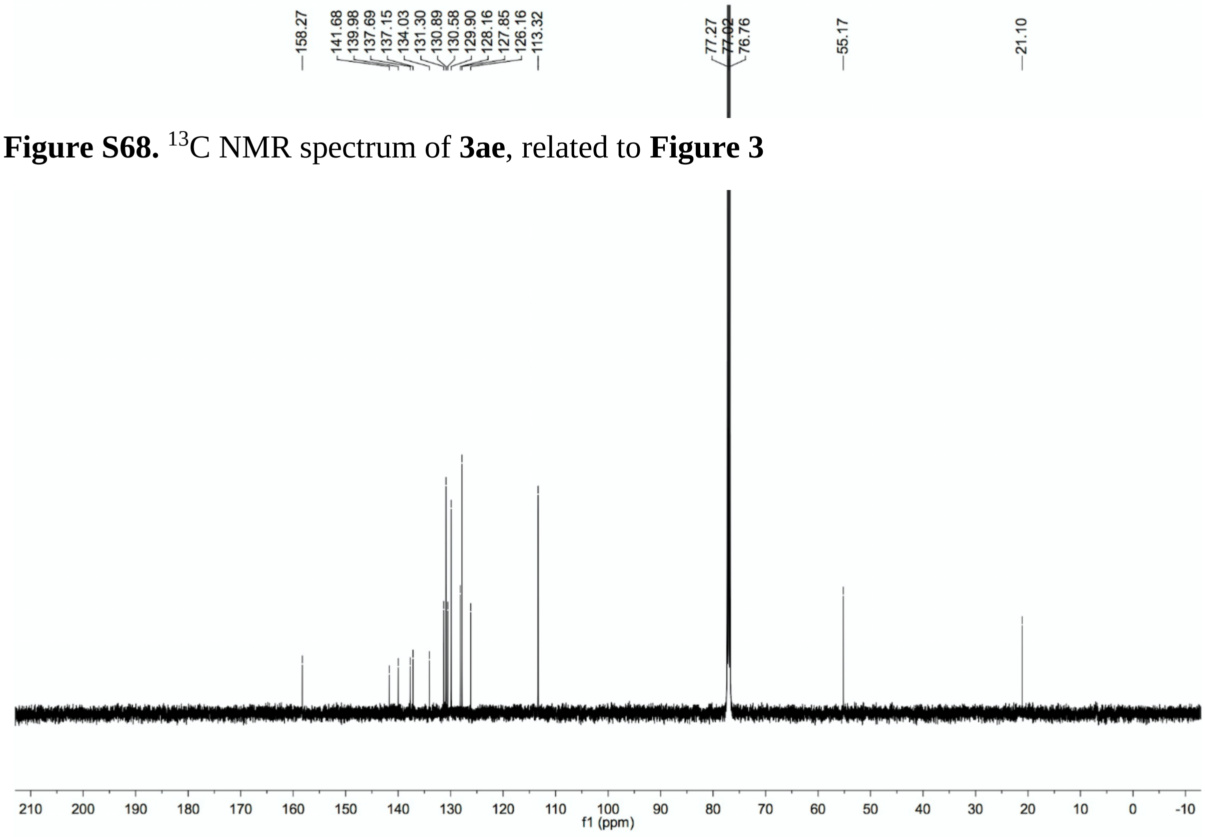

Figure S69. ^{1}H NMR spectrum of **3af**, related to **Figure 3**

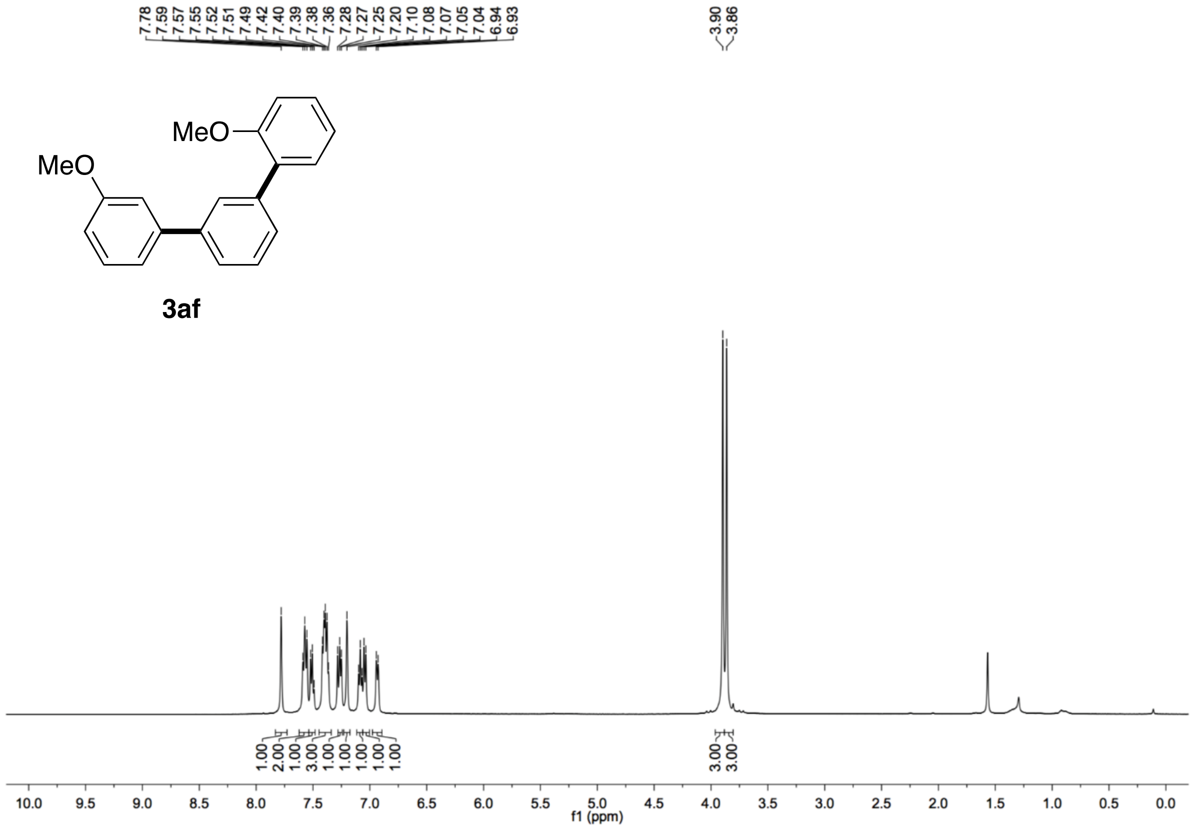

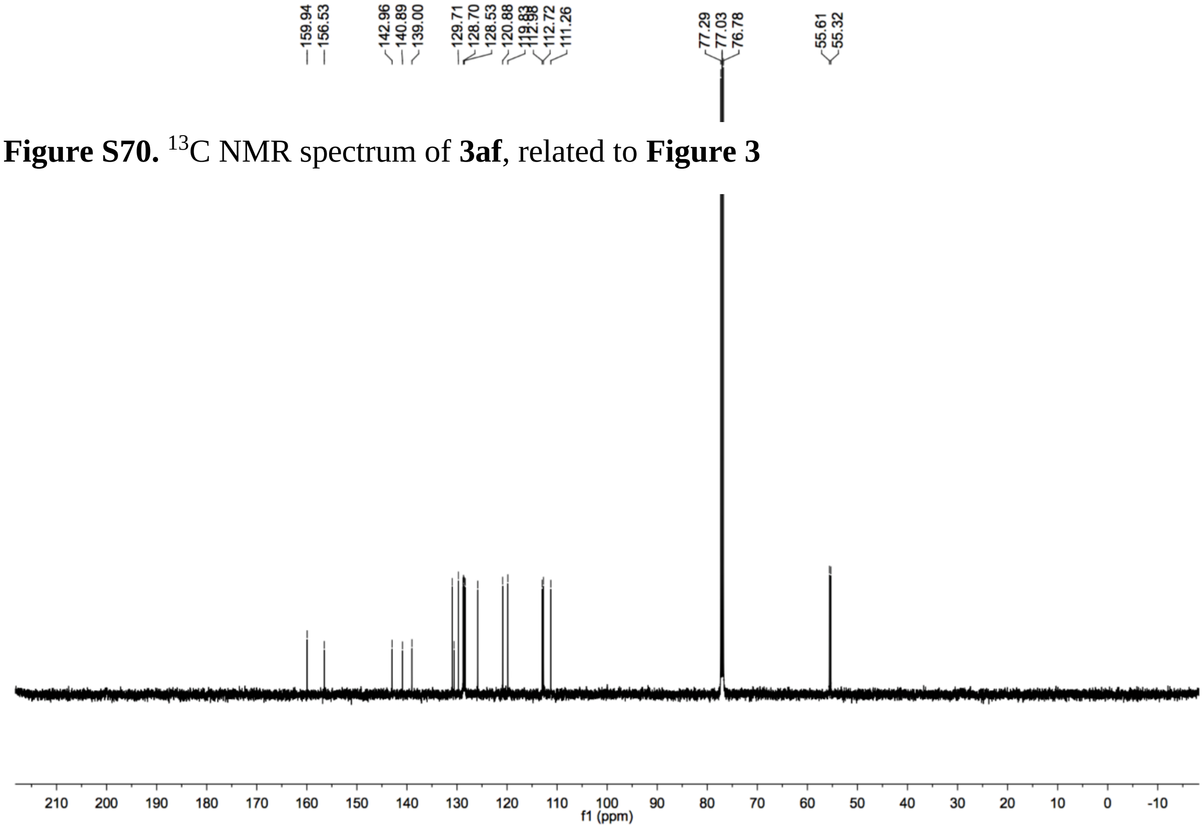

Figure S70. ^{13}C NMR spectrum of **3af**, related to **Figure 3**

Figure S71. ^{1}H NMR spectrum of **3ag**, related to **Figure 3**

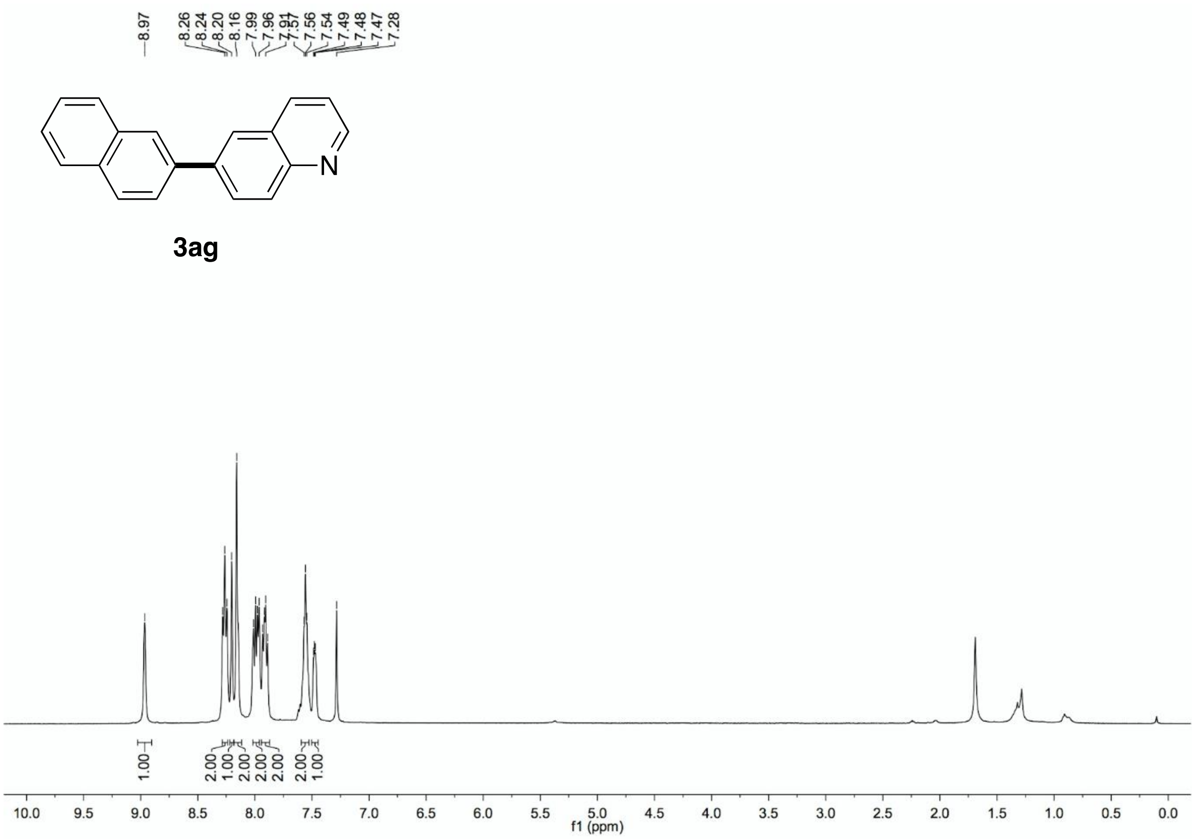

Figure S72. ^{13}C NMR spectrum of **3ag**, related to **Figure 3**

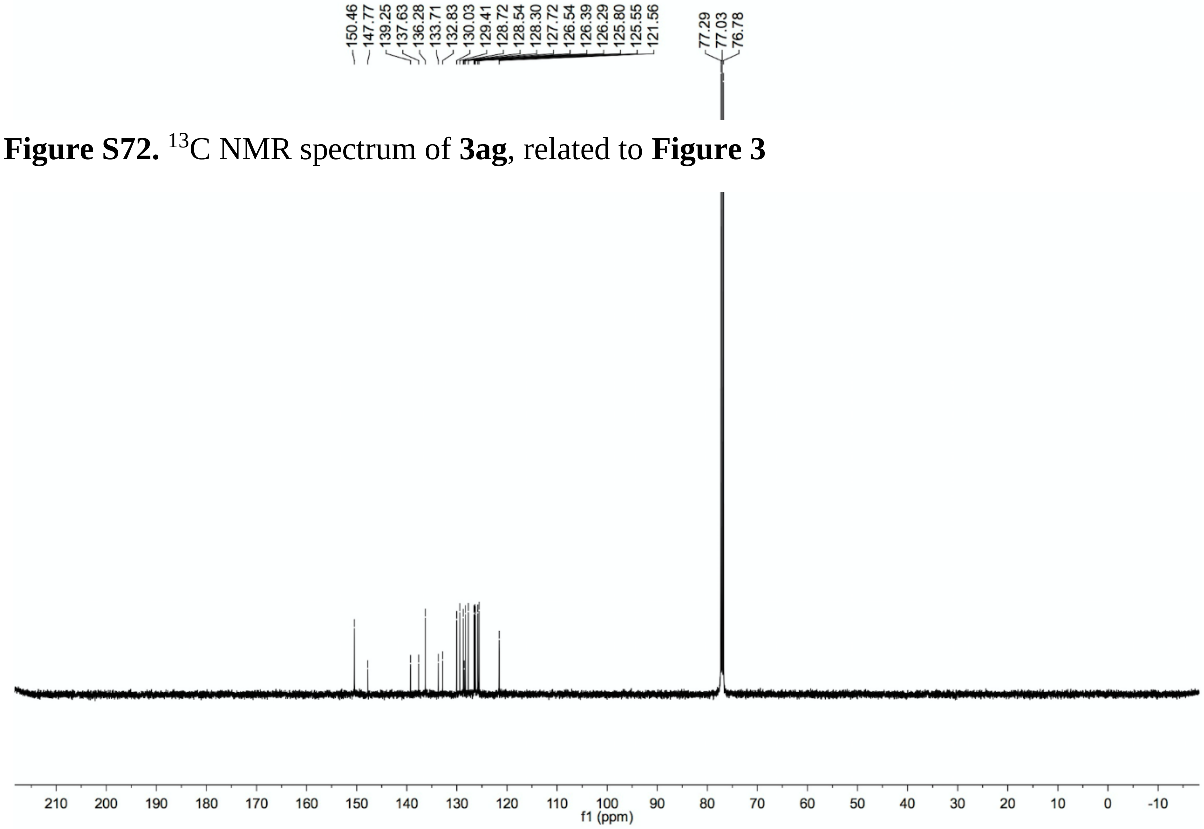

Figure S73. ^{1}H NMR spectrum of **3ah**, related to **Figure 3**

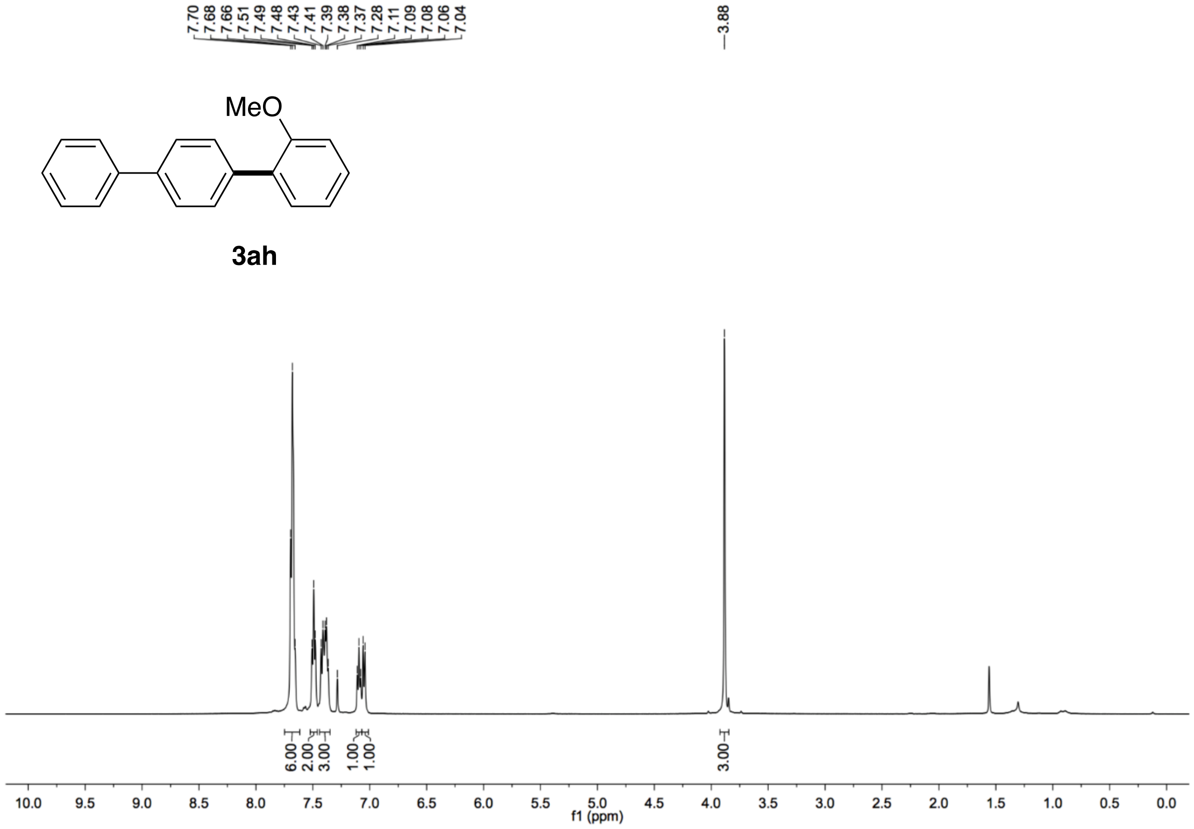

Figure S74. ^{13}C NMR spectrum of **3ah**, related to **Figure 3**

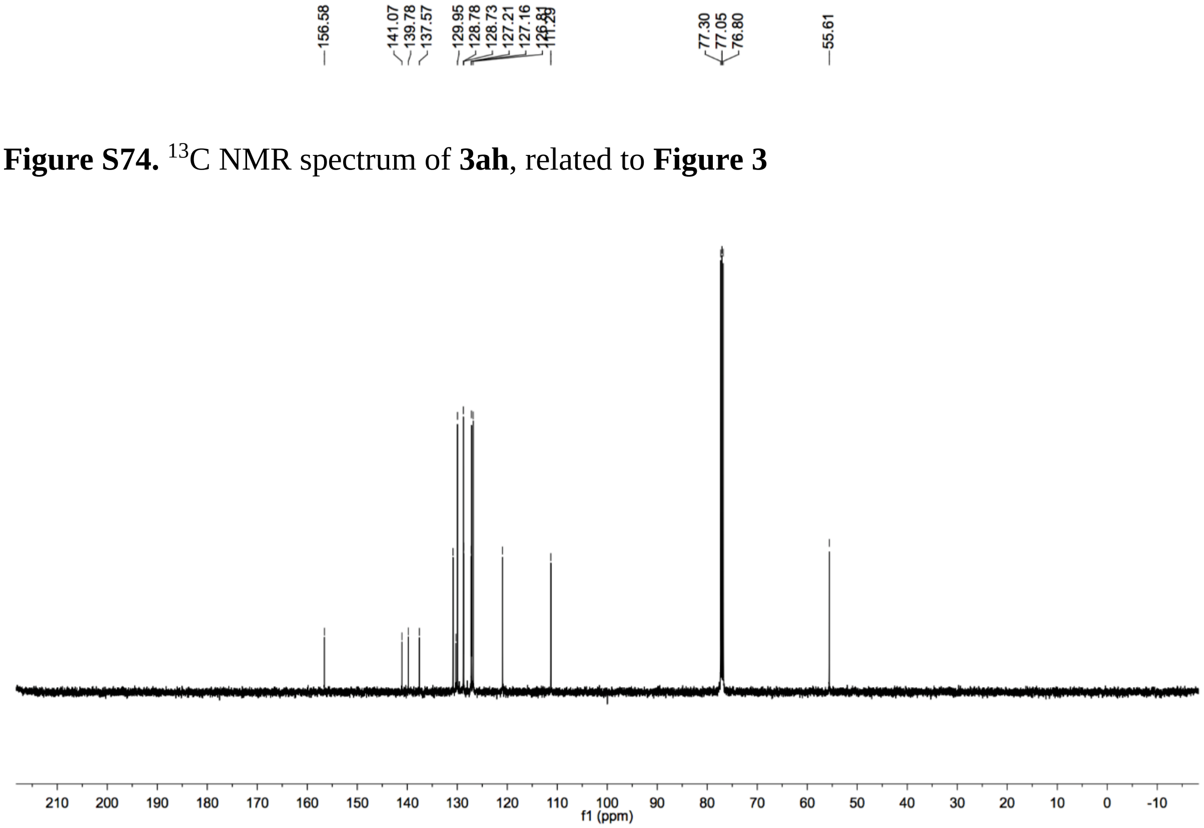

Figure S75. ^{1}H NMR spectrum of **3ai**, related to **Figure 4**

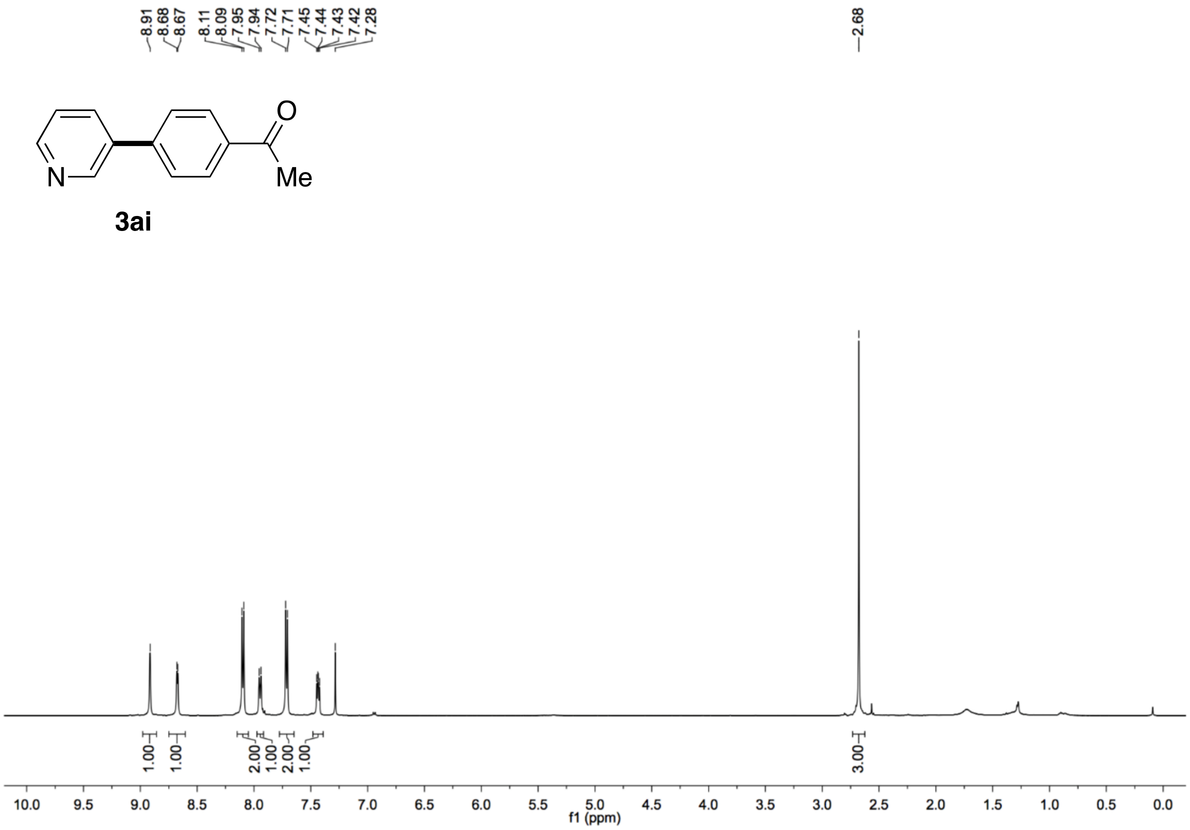

Figure S76. ^{13}C NMR spectrum of **3ai**, related to **Figure 4**

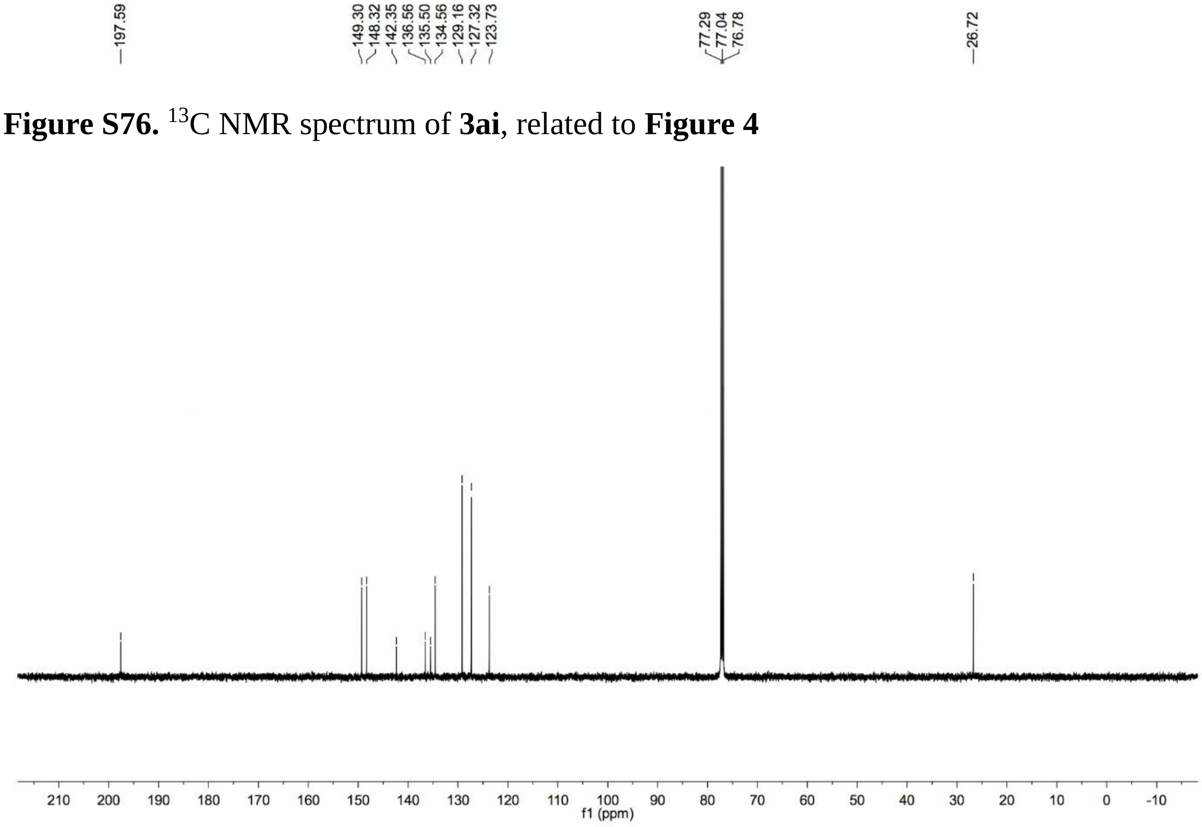

Figure S77. ^{1}H NMR spectrum of **3aj**, related to **Figure 4**

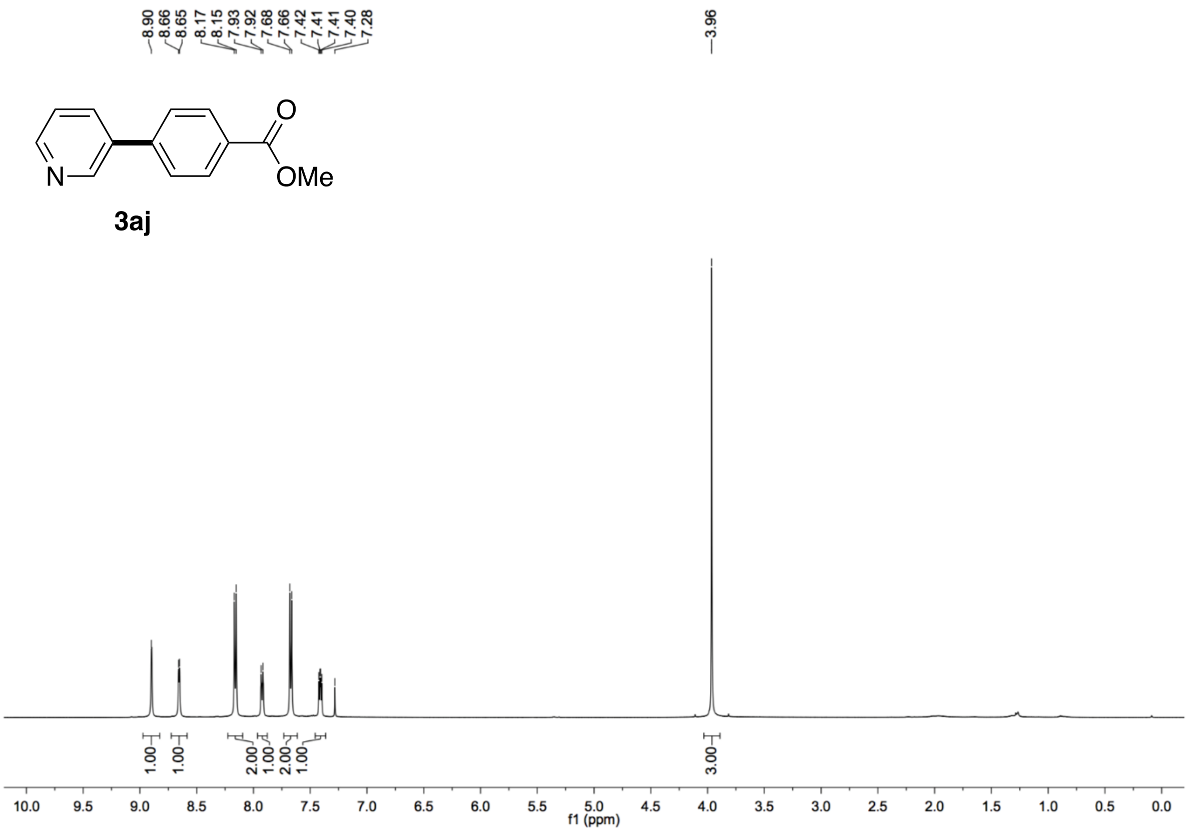

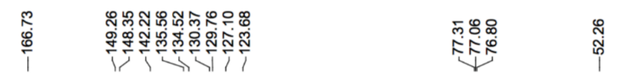

Figure S78. ^{13}C NMR spectrum of **3aj**, related to **Figure 4**

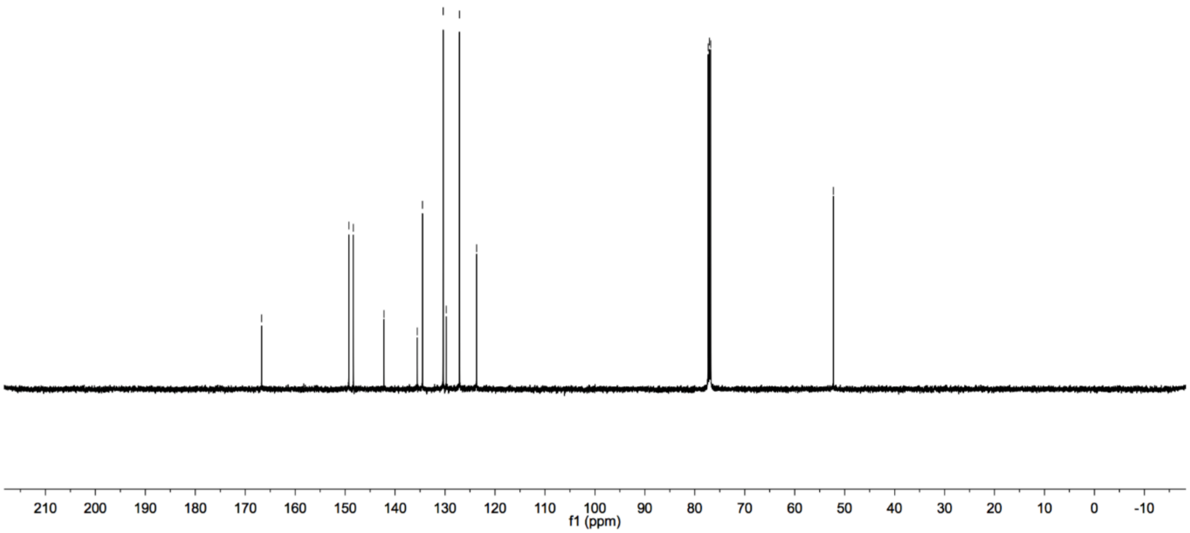

Figure S79. ^{1}H NMR spectrum of **3ak**, related to **Figure 4**

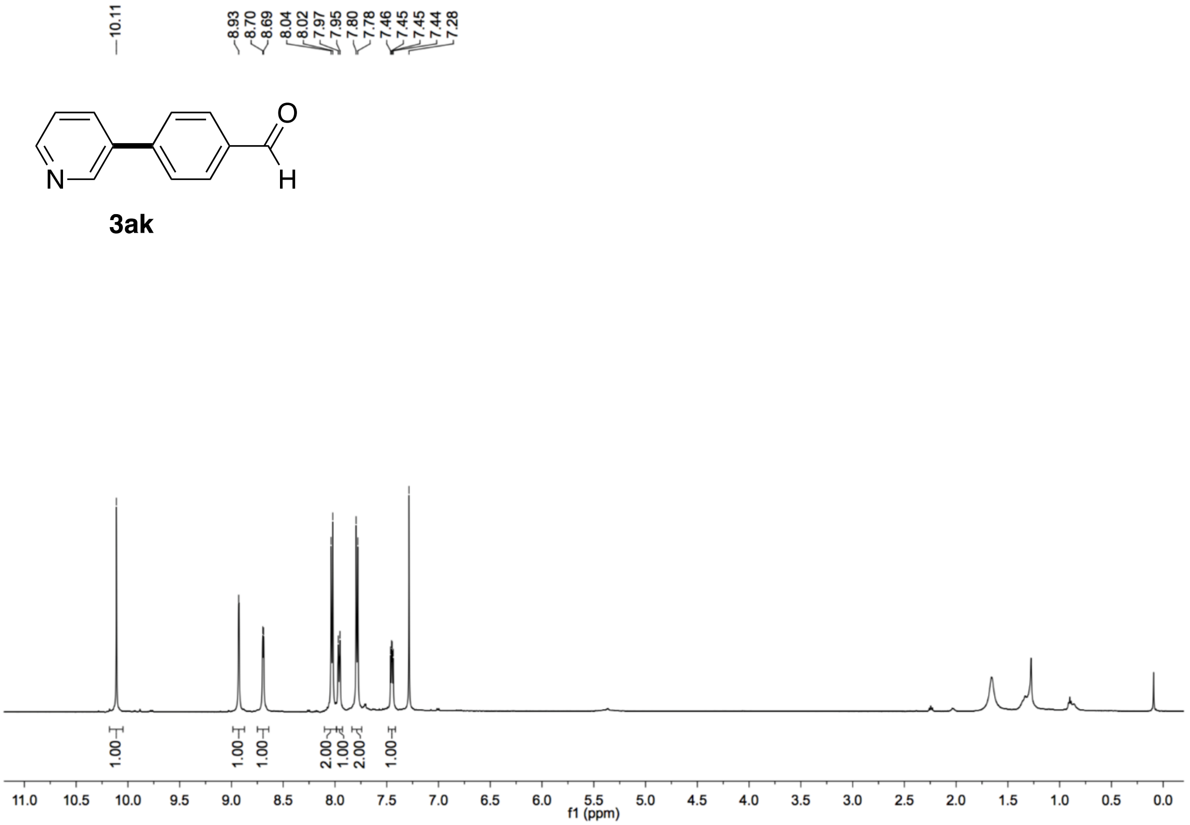

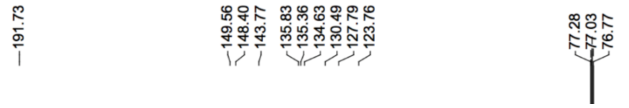

Figure S80. ^{13}C NMR spectrum of **3ak**, related to **Figure 4**

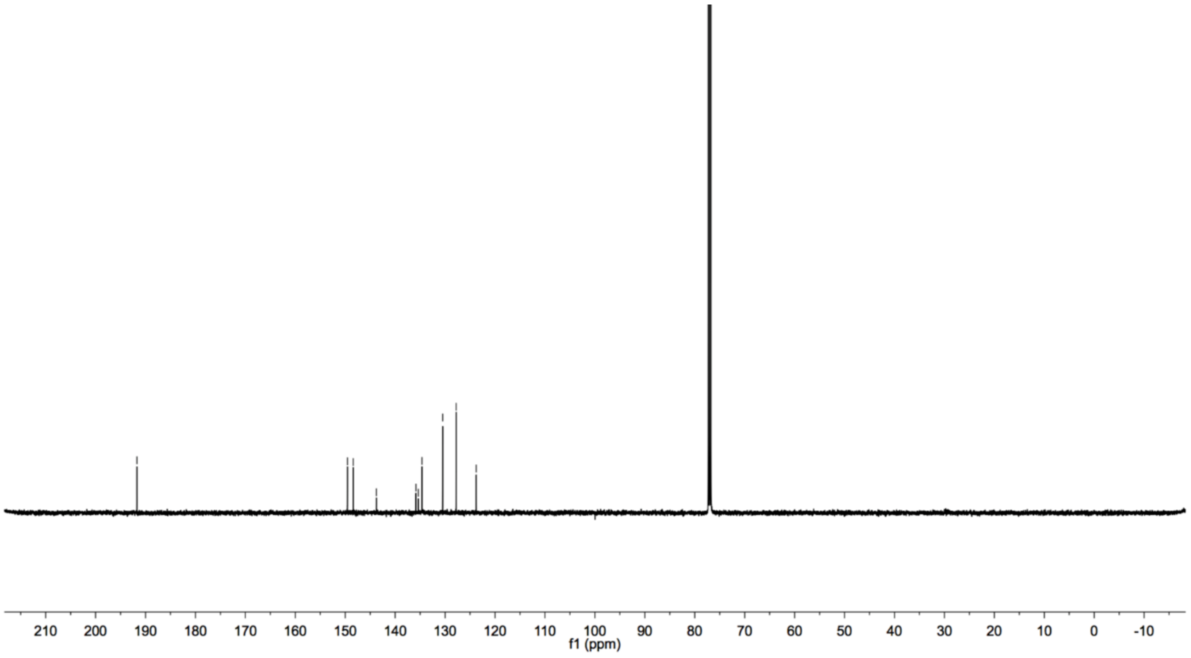

Figure S81. ^{1}H NMR spectrum of **3al**, related to **Figure 4**

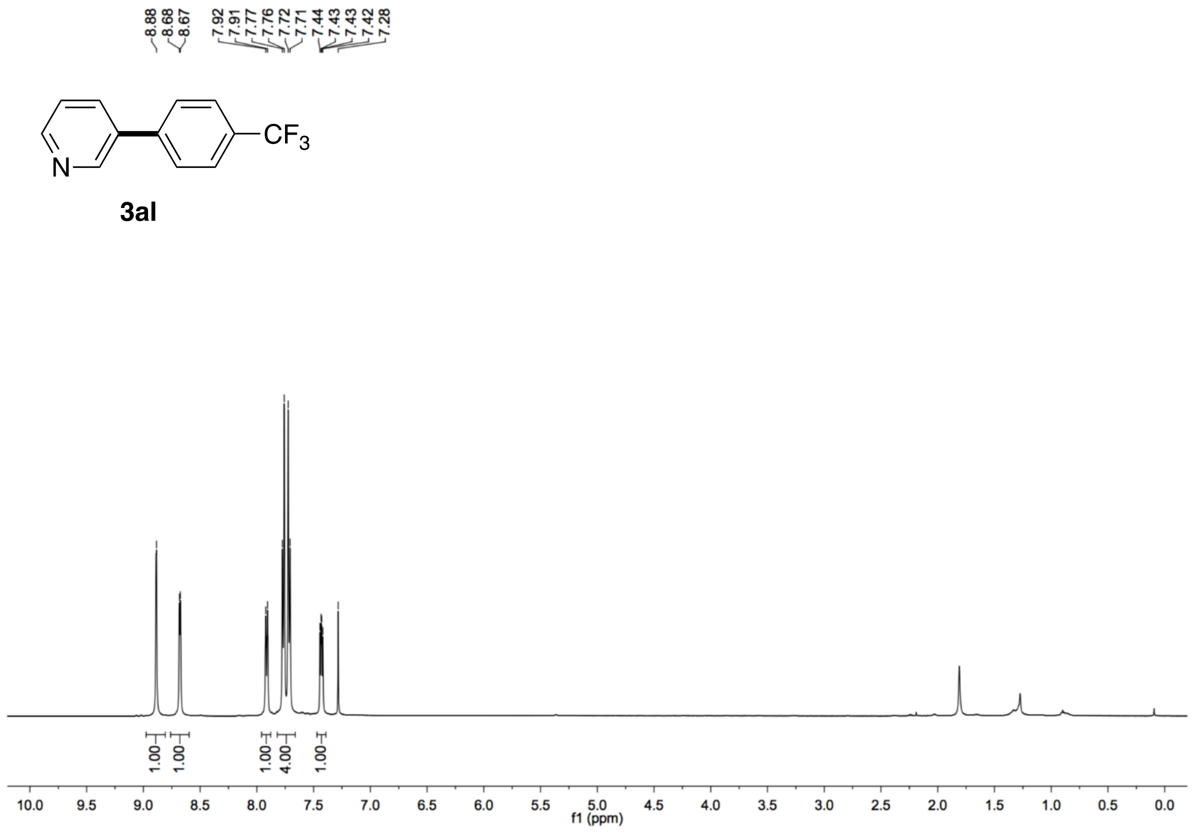

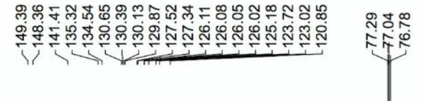

Figure S82. ^{13}C NMR spectrum of **3al**, related to **Figure 4**

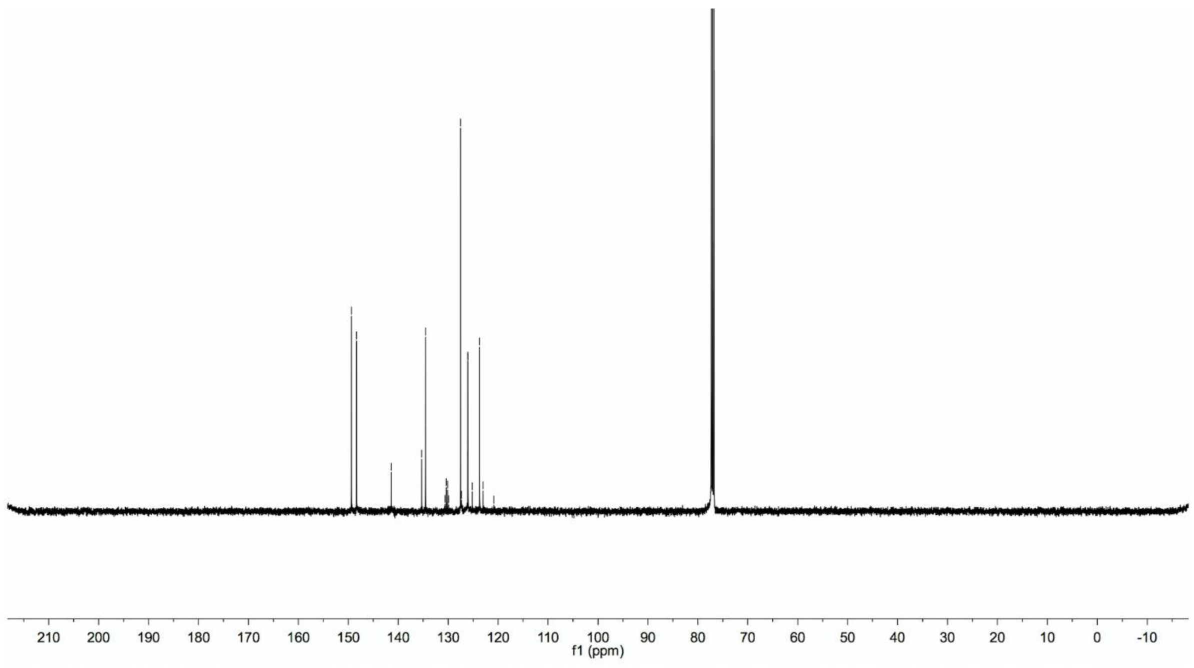

Figure S83. ^{19}F NMR spectrum of **3al**, related to **Figure 4**

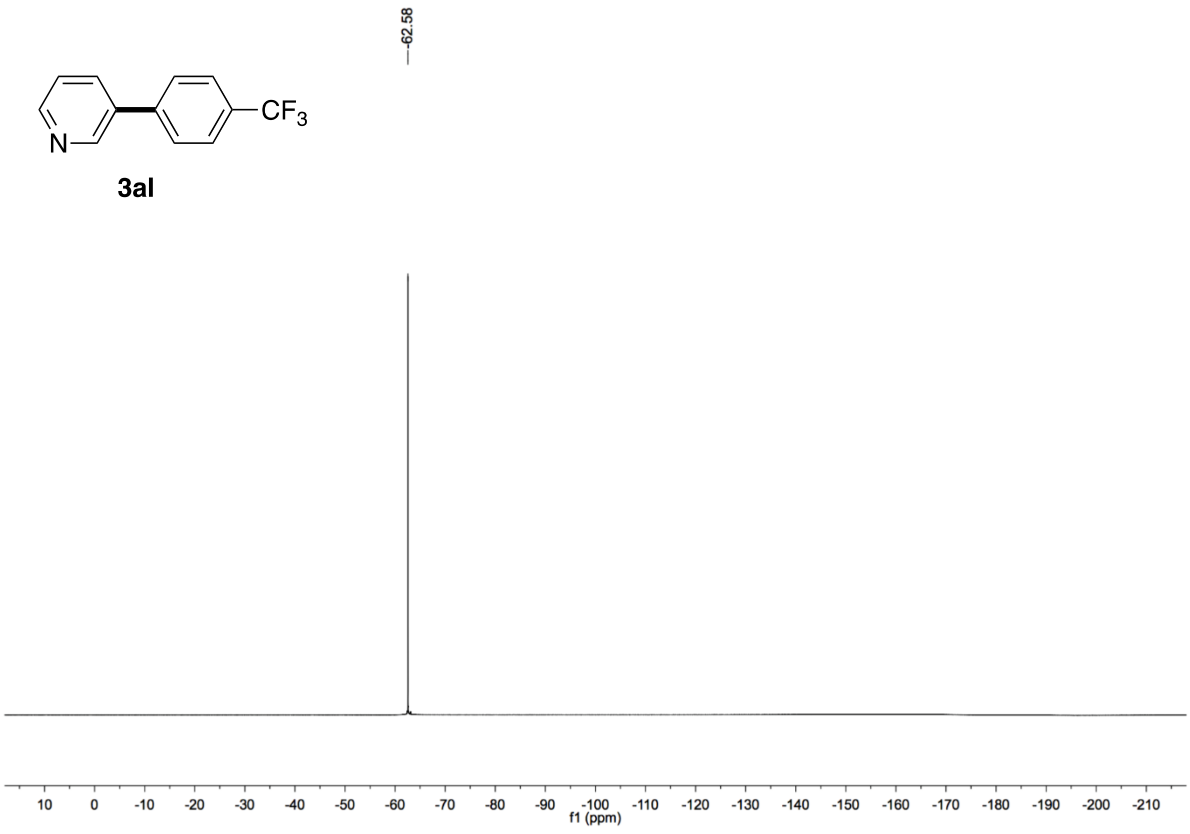

Figure S84. ^{1}H NMR spectrum of **3am**, related to **Figure 4**

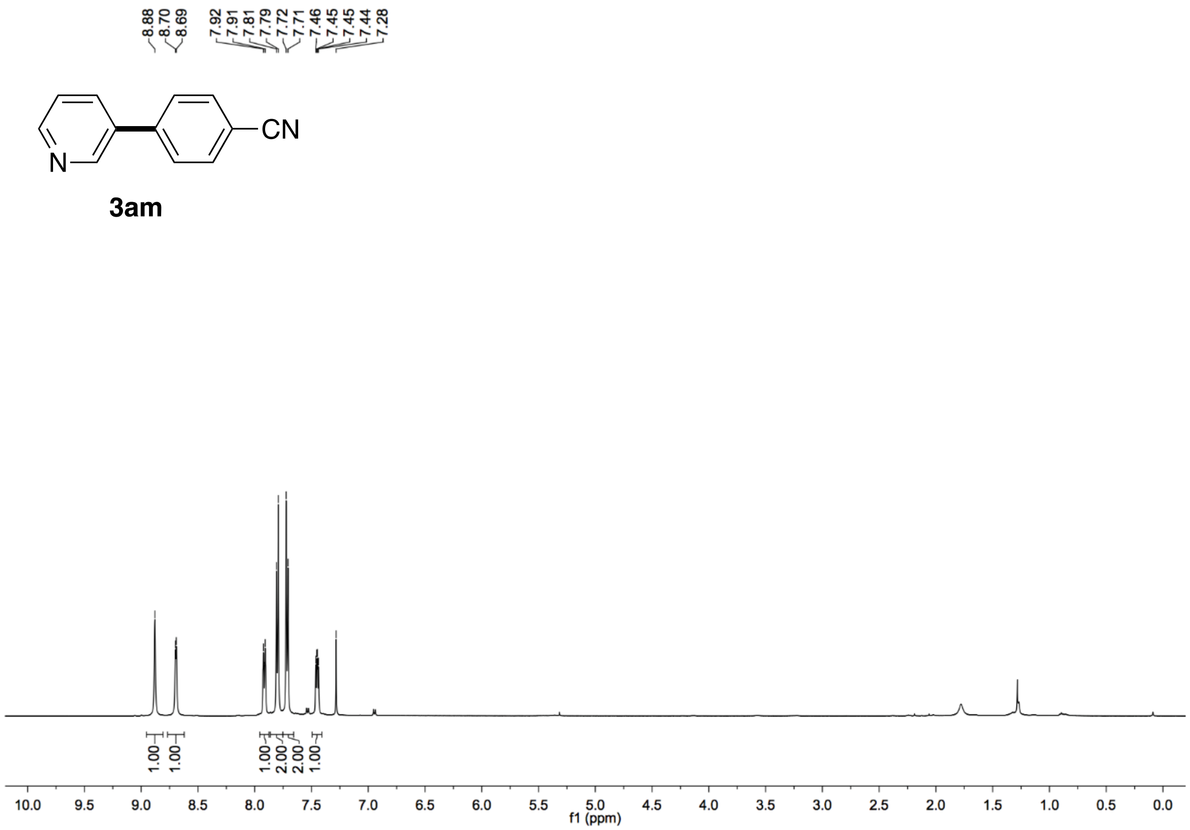

Figure S85. ^{13}C NMR spectrum of **3am**, related to **Figure 4**

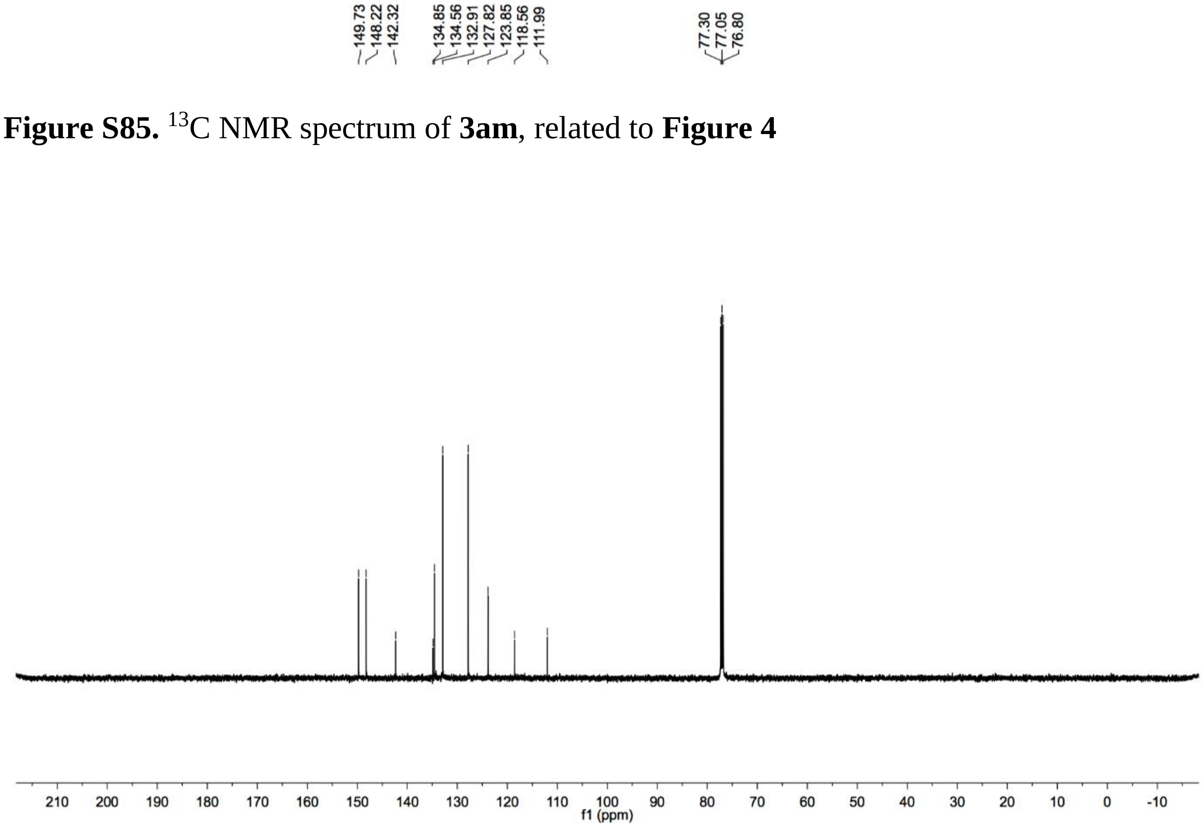

Figure S86. ^{1}H NMR spectrum of **3an**, related to **Figure 4**

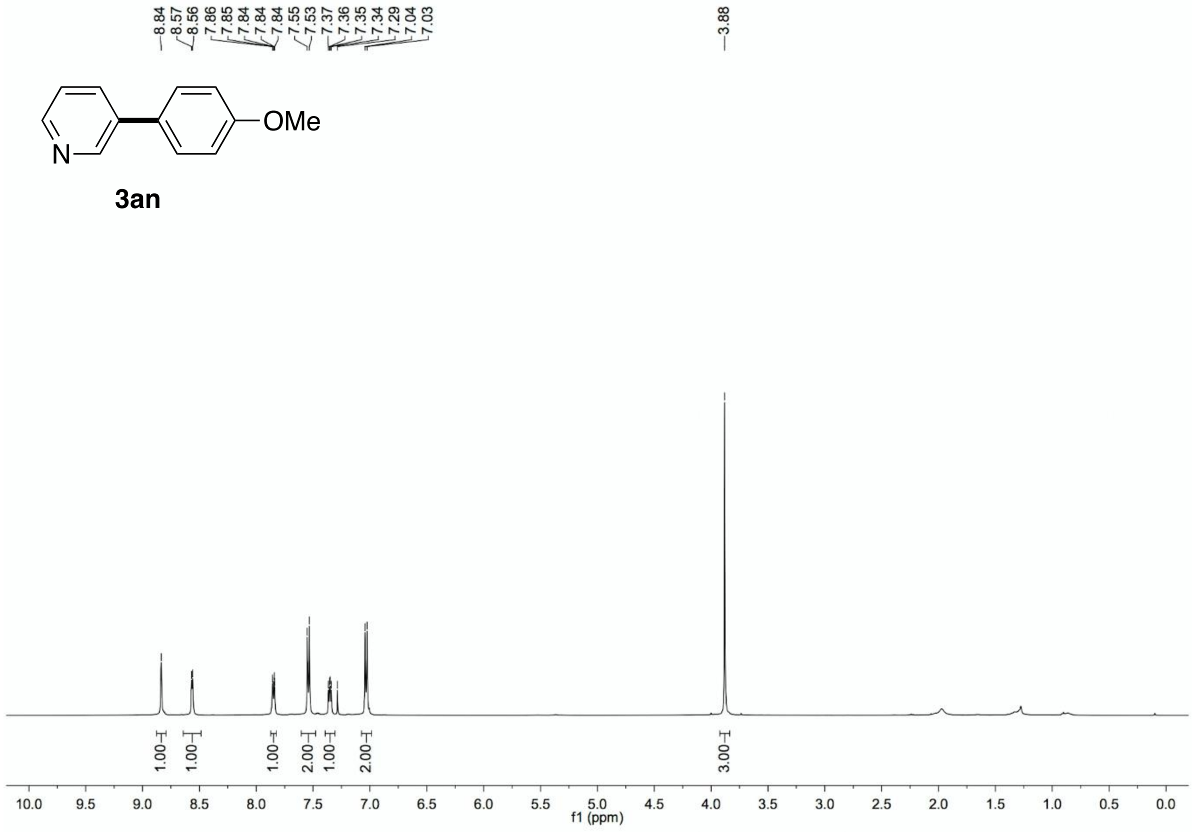

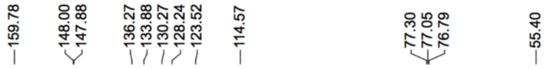

Figure S87. ^{13}C NMR spectrum of **3an**, related to **Figure 4**

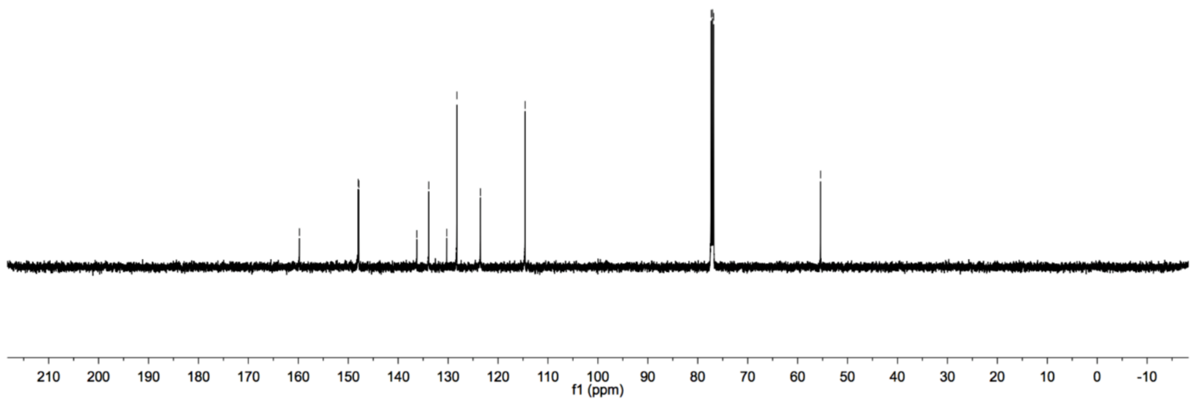

Figure S88. ^{1}H NMR spectrum of **3ao**, related to **Figure 4**

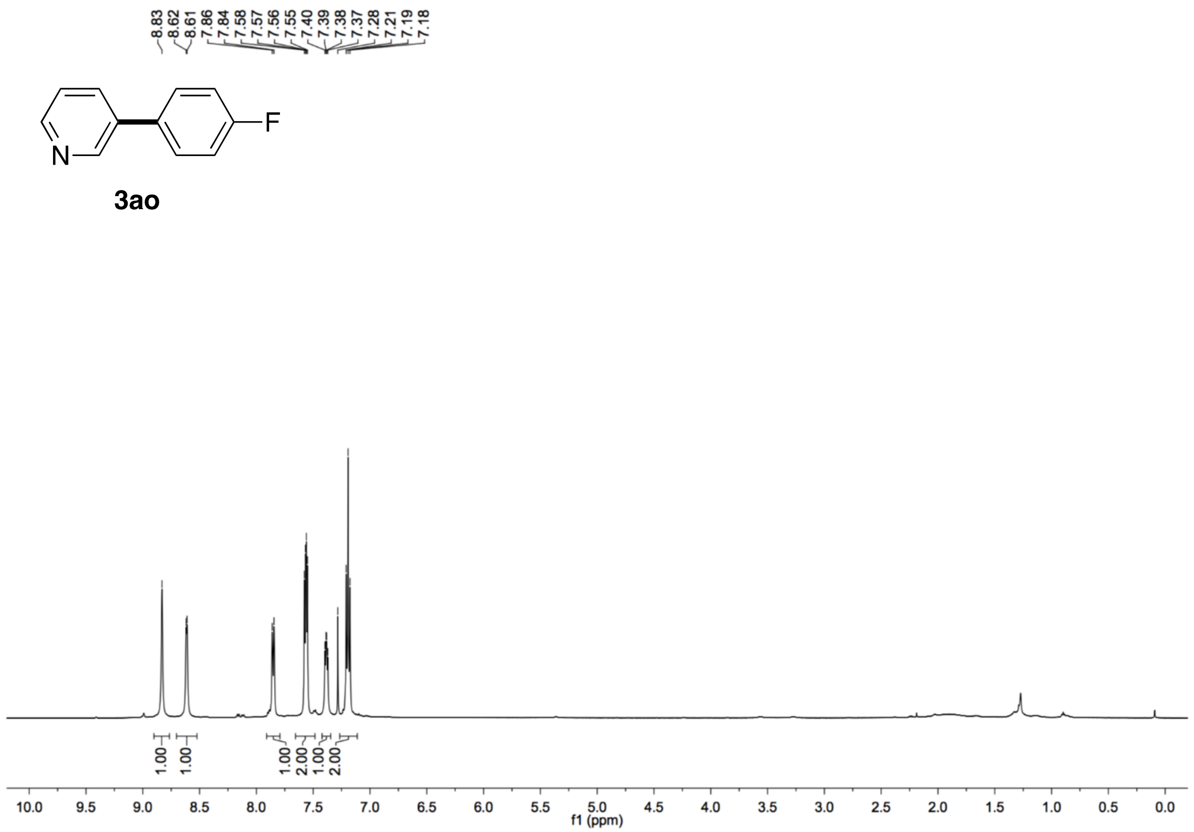

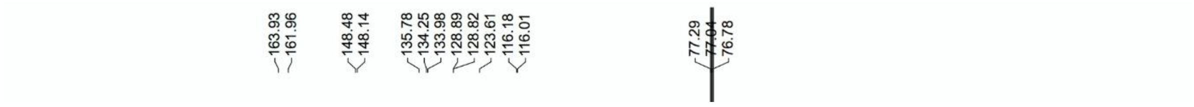

Figure S89. ^{13}C NMR spectrum of **3ao**, related to **Figure 4**

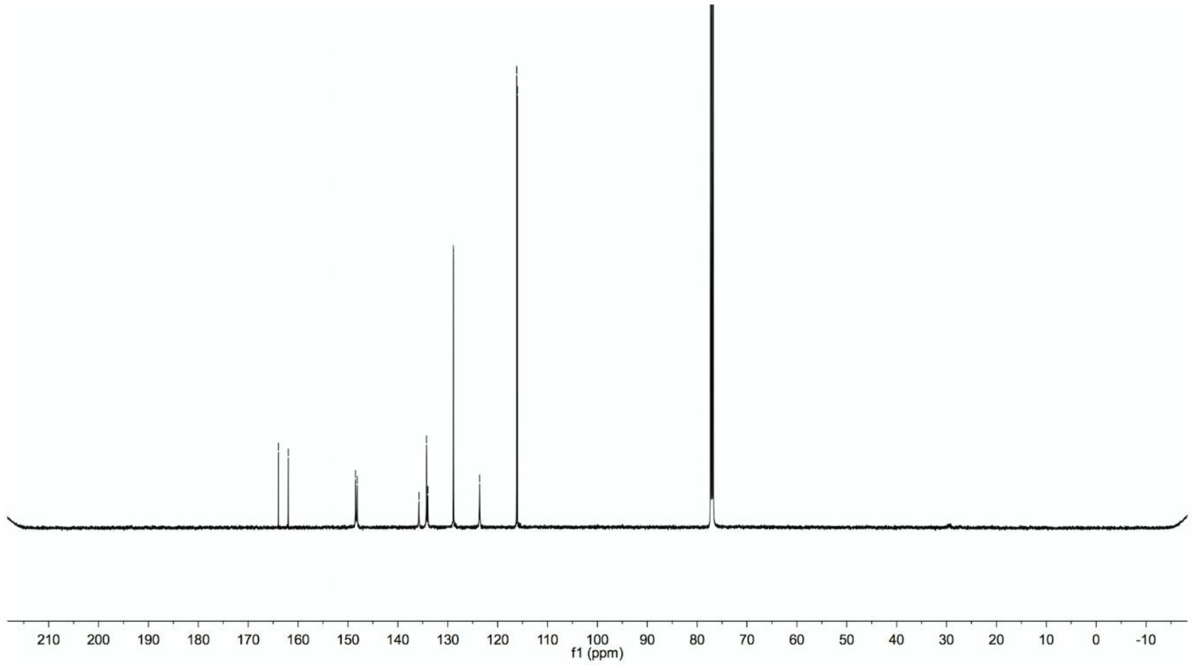

Figure S90. ^{19}F NMR spectrum of **3ao**, related to **Figure 4**

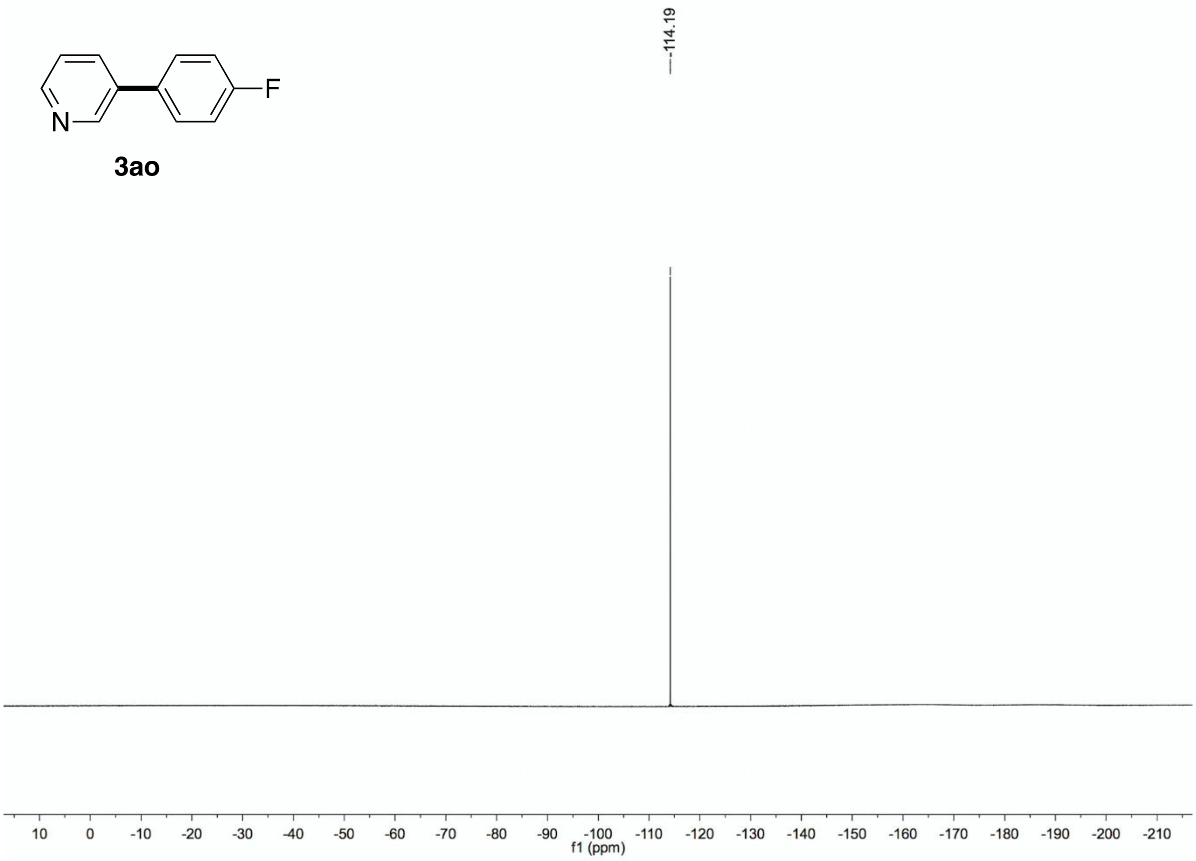

Figure S91. ^{1}H NMR spectrum of **3ap**, related to **Figure 4**

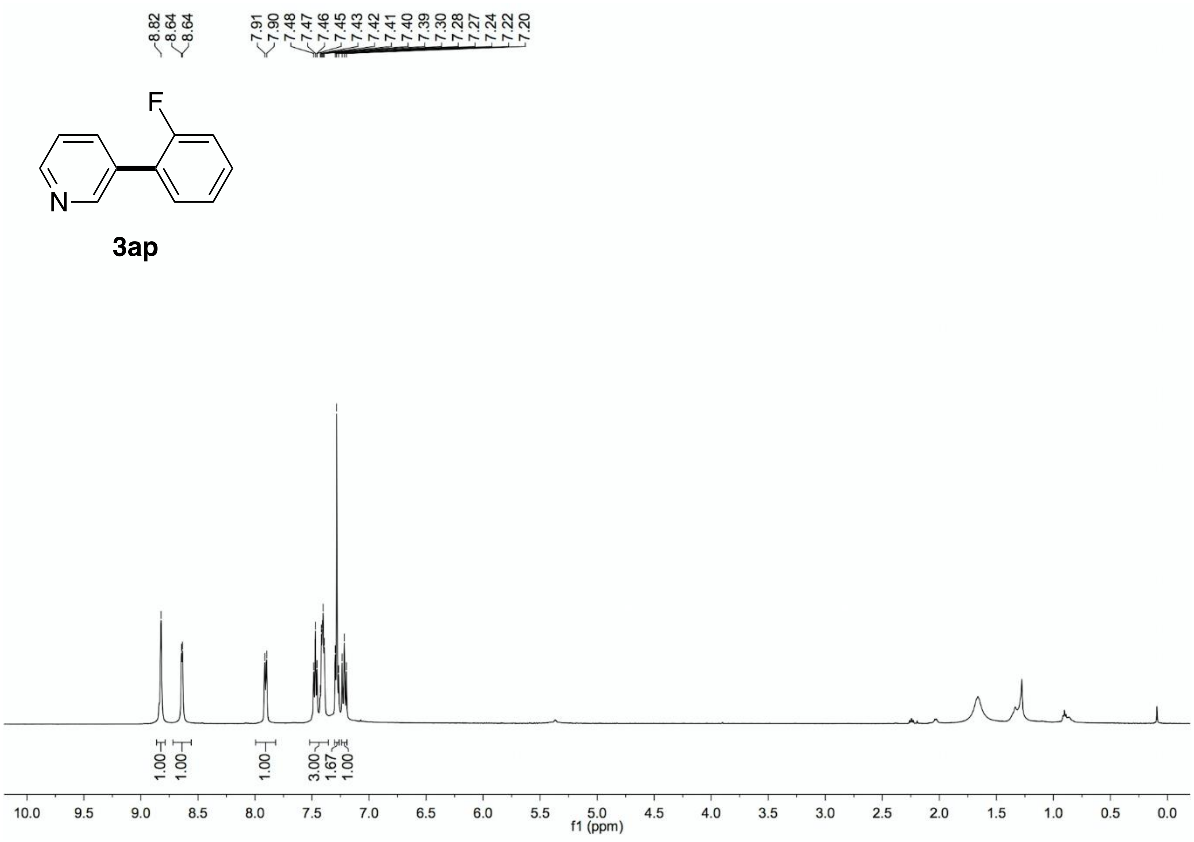

Figure S92. ^{13}C NMR spectrum of **3ap**, related to **Figure 4**

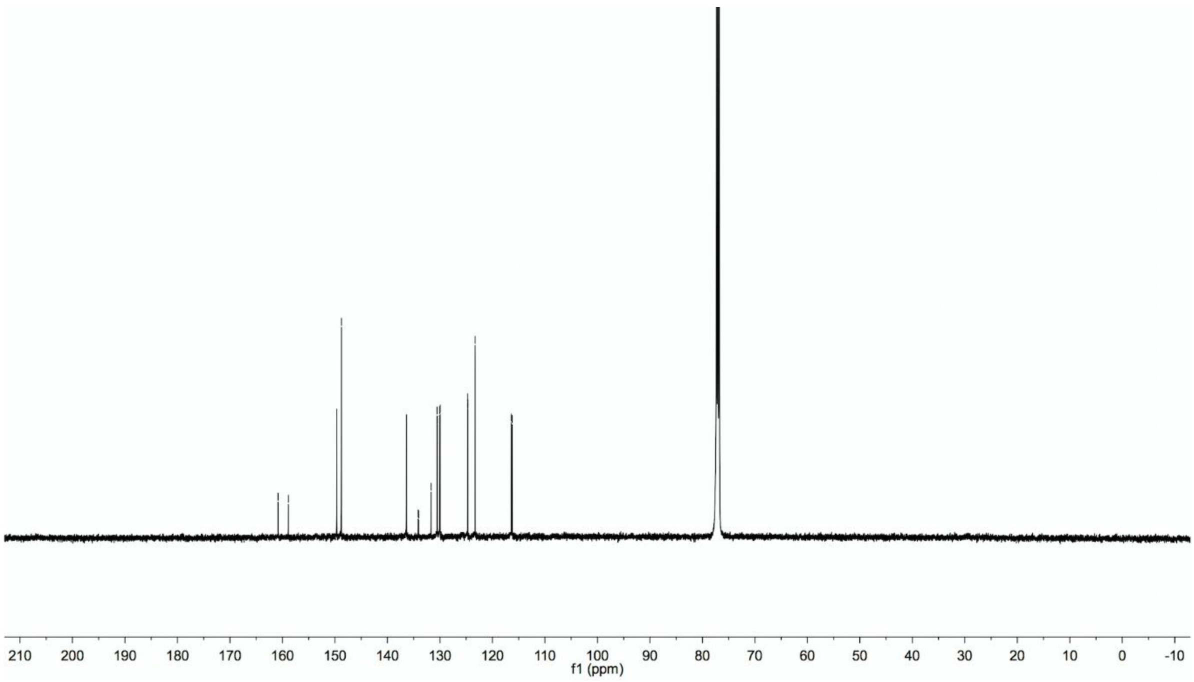

Figure S93. ^{19}F NMR spectrum of **3ap**, related to **Figure 4**

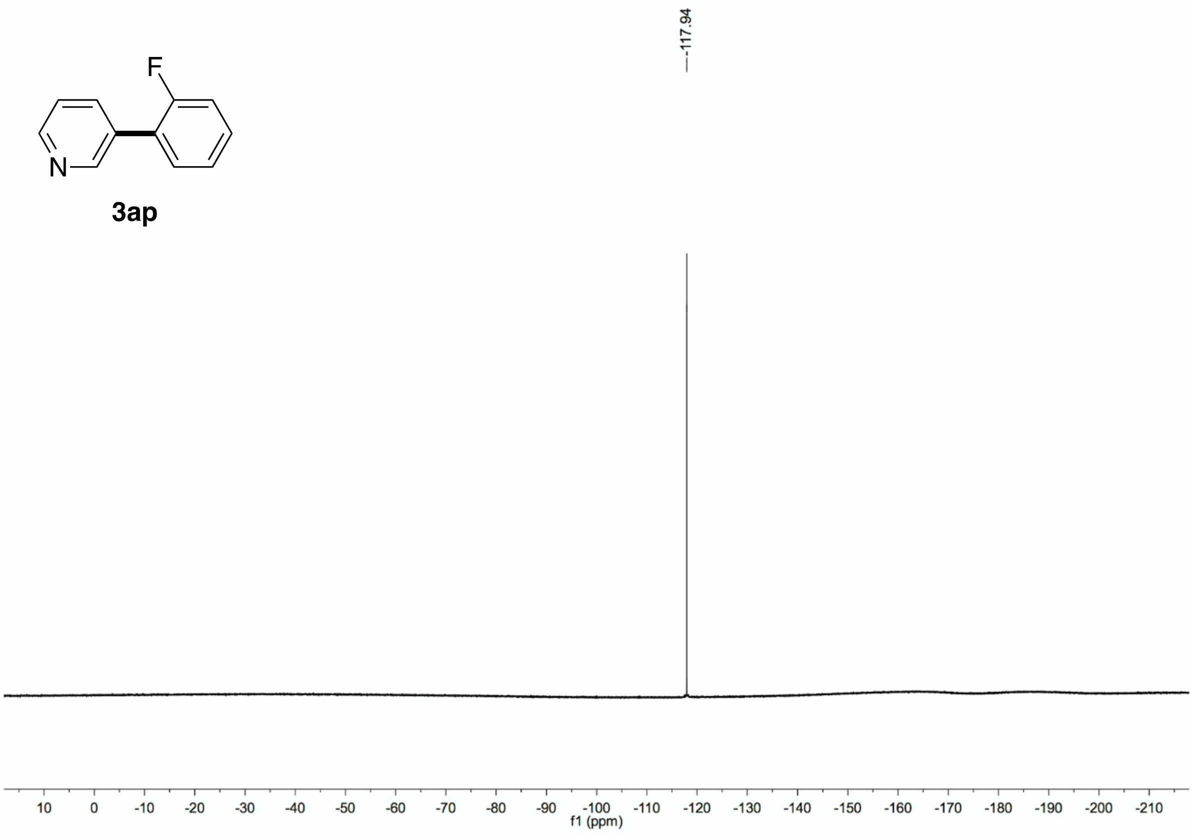

Figure S94. ^{1}H NMR spectrum of **3aq**, related to **Figure 4**

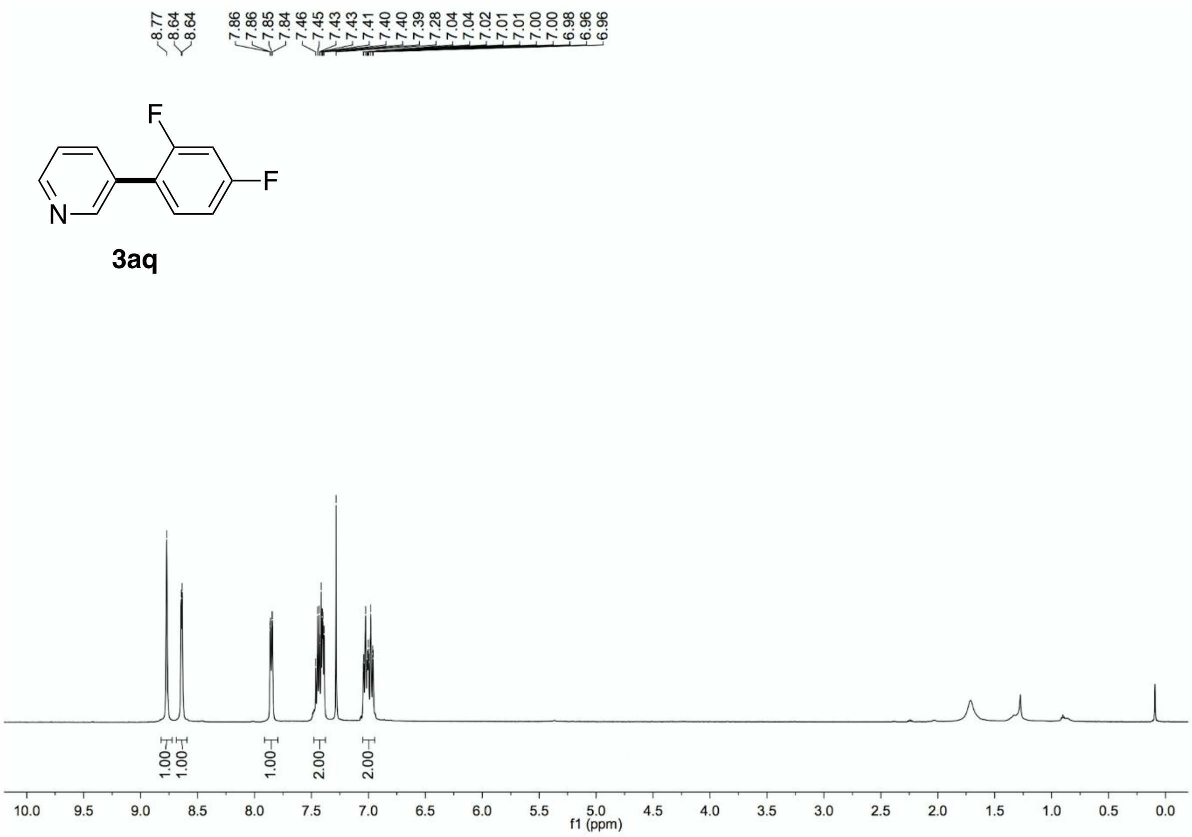

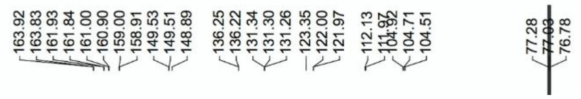

Figure S95. ^{13}C NMR spectrum of **3aq**, related to **Figure 4**

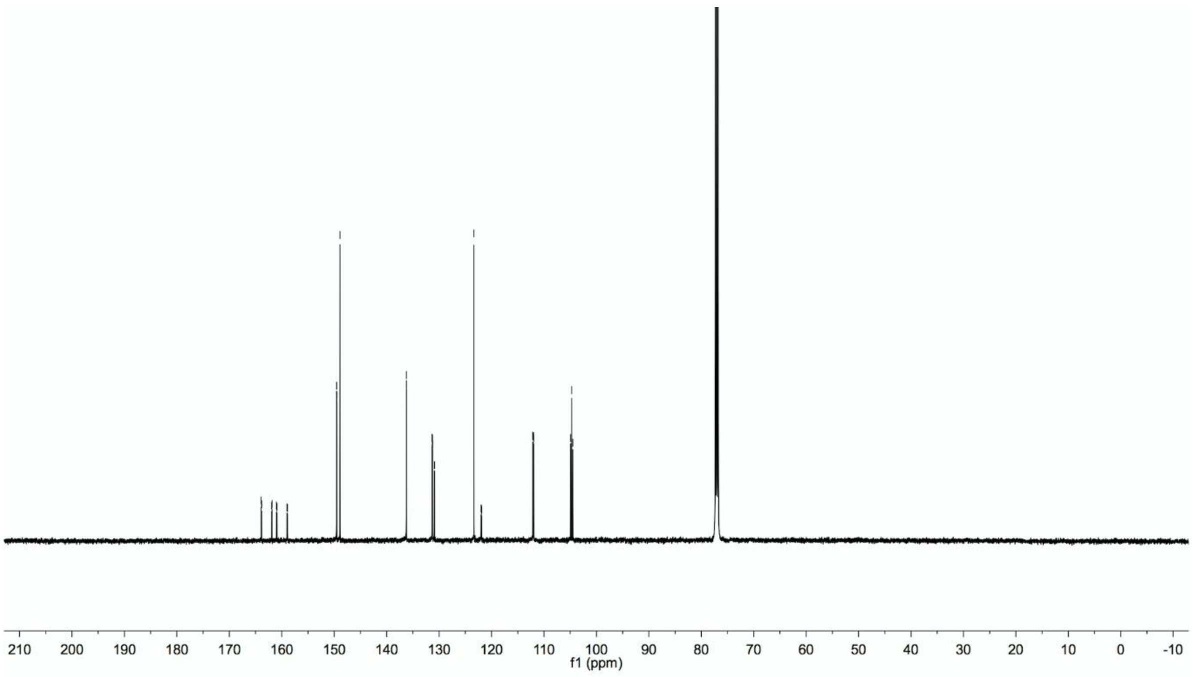

Figure S96. ^{19}F NMR spectrum of **3aq**, related to **Figure 4**

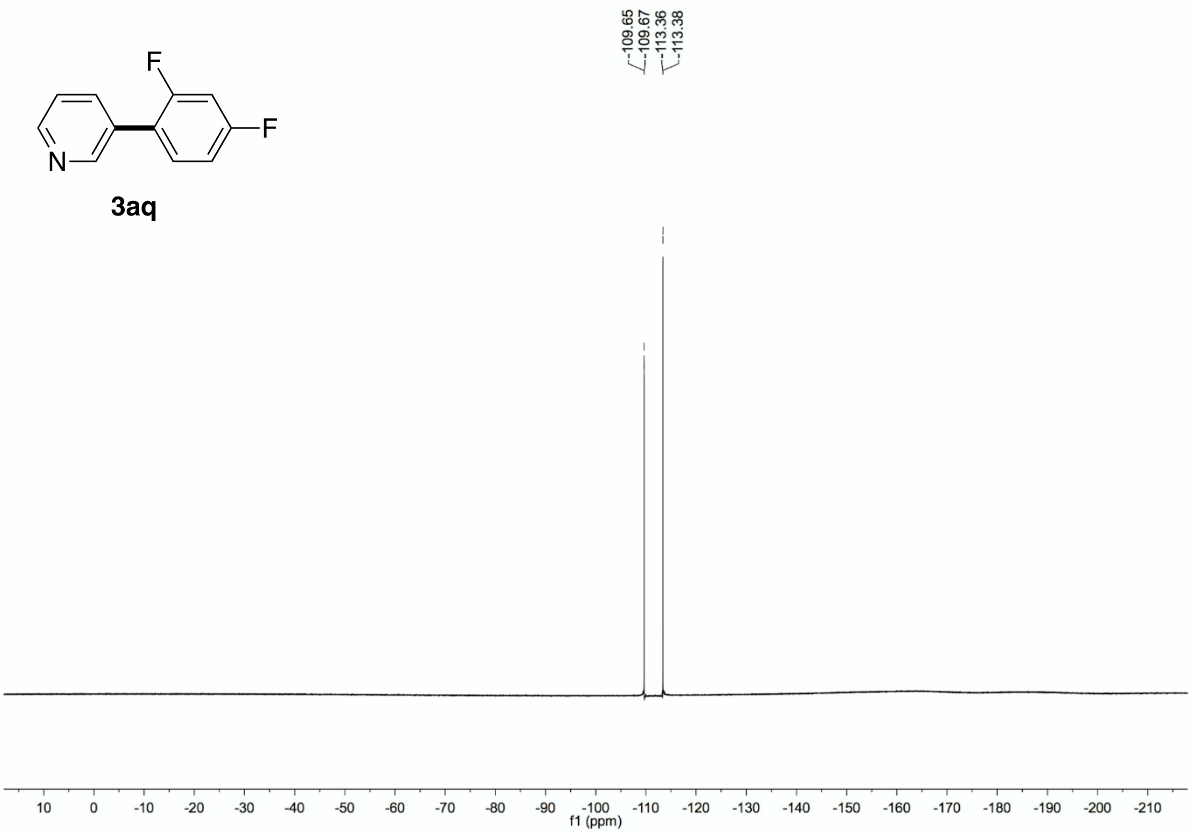

Figure S97. ^{1}H NMR spectrum of **3ar**, related to **Figure 4**

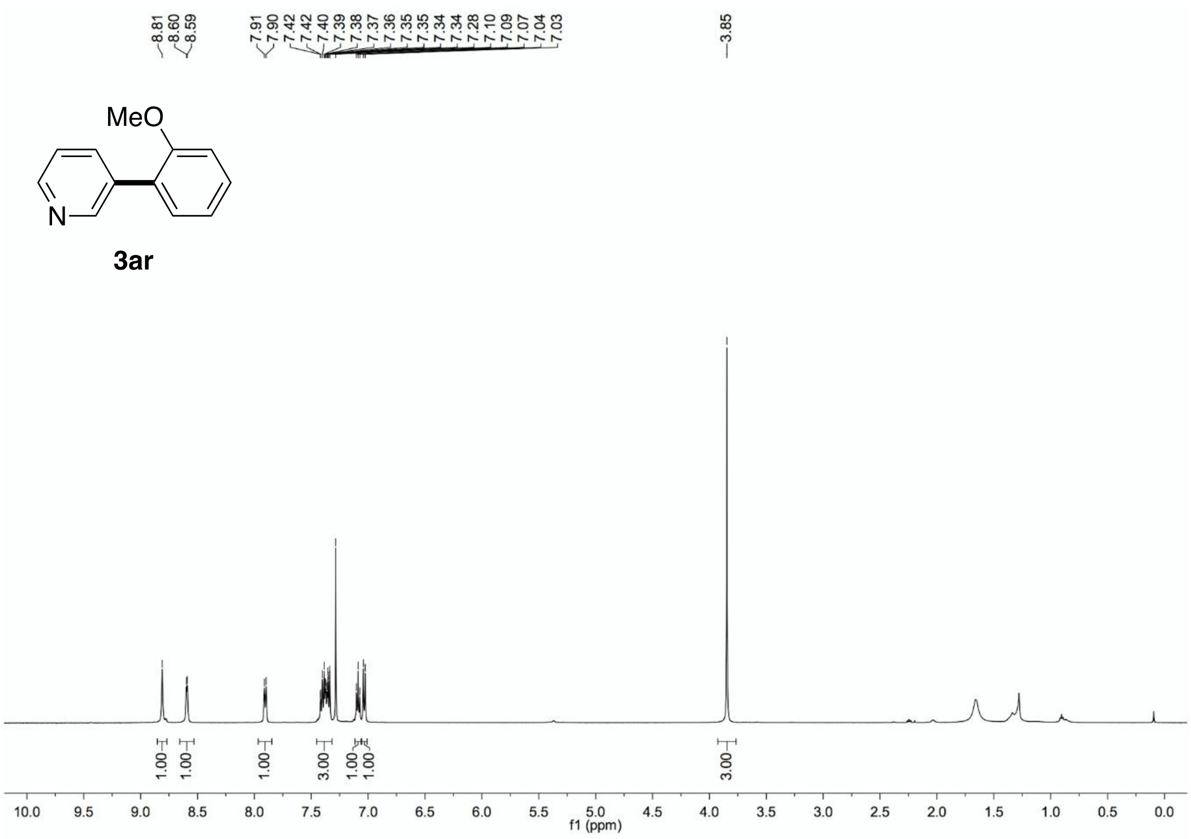

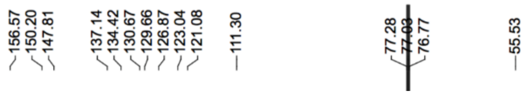

Figure S98. ^{13}C NMR spectrum of **3ar**, related to **Figure 4**

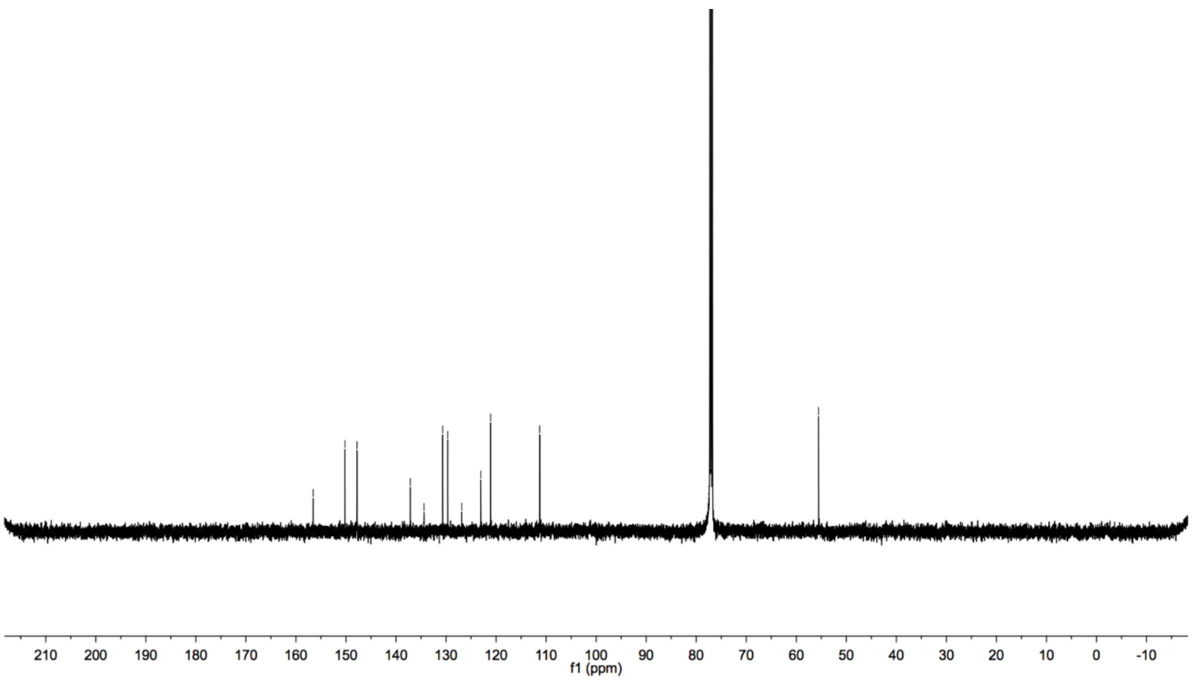

Figure S99. ^{1}H NMR spectrum of **3as**, related to **Figure 4**

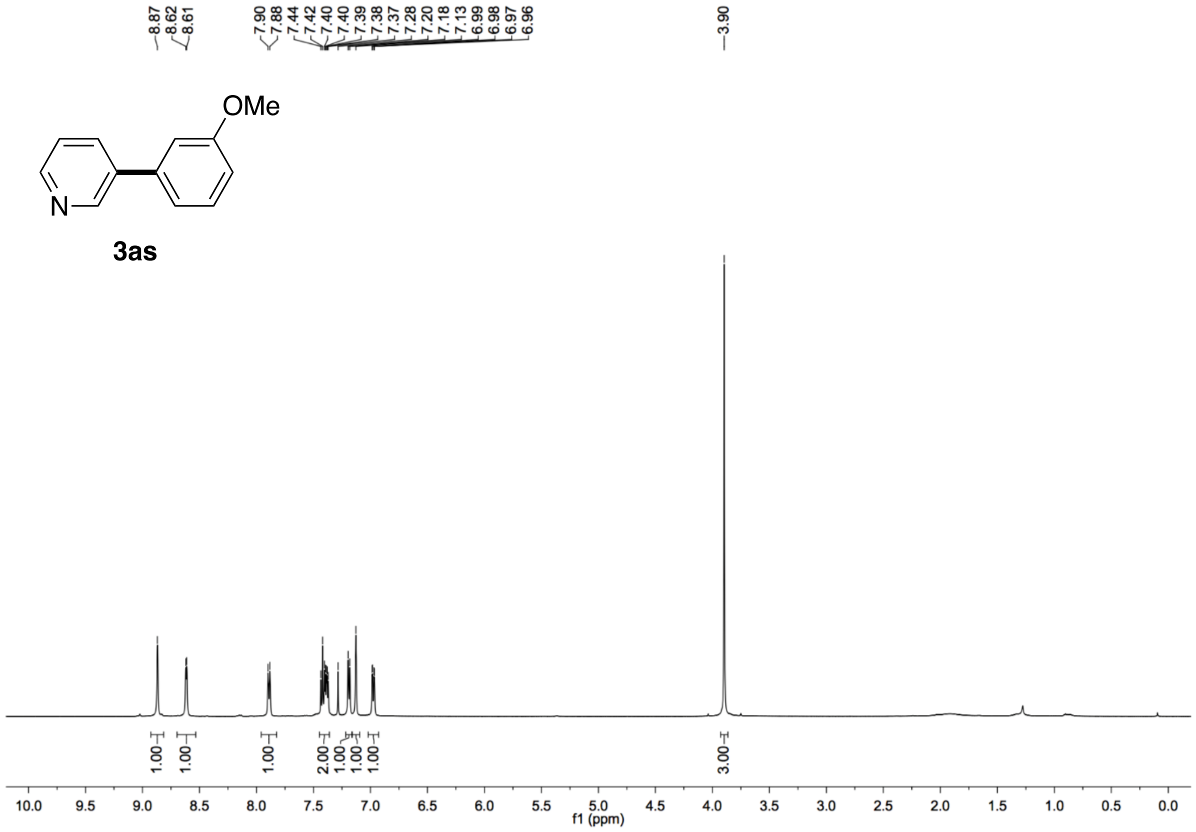

Figure S100. ^{13}C NMR spectrum of **3as**, related to **Figure 4**

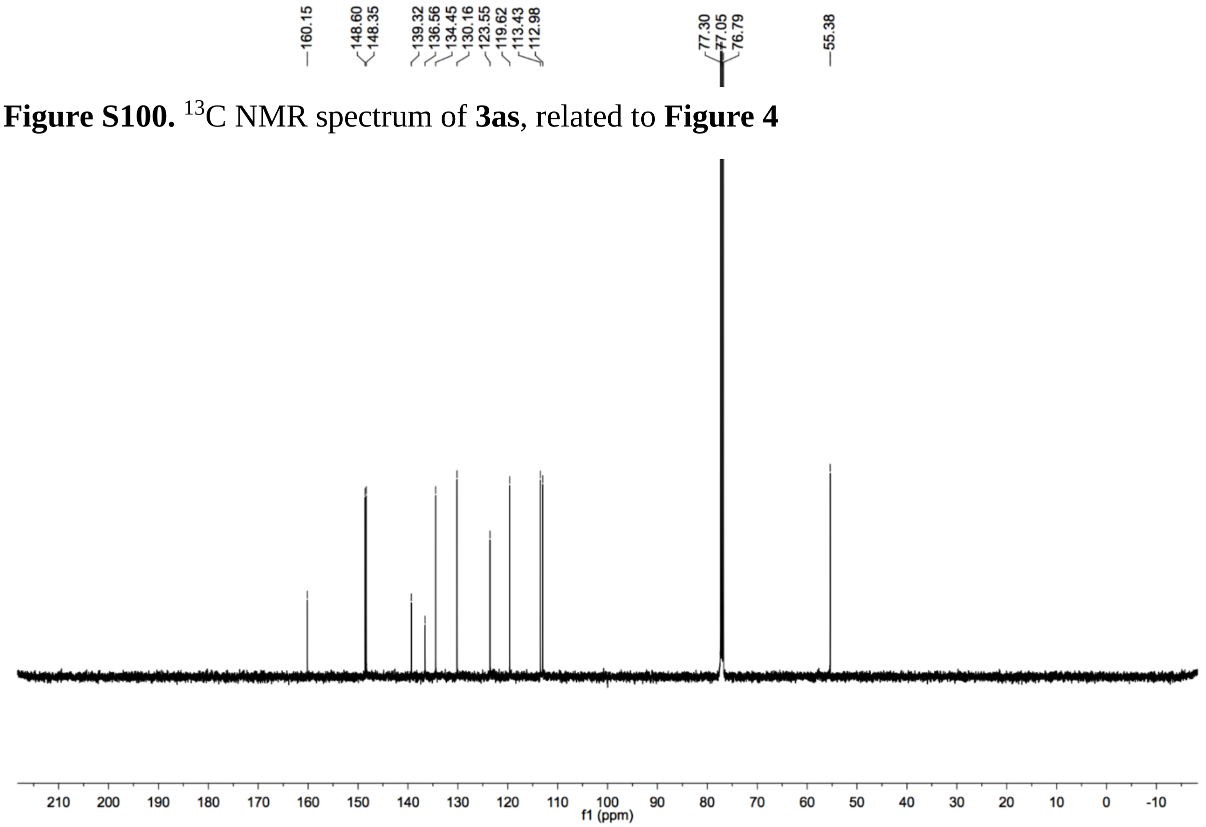

Figure S101. ^{1}H NMR spectrum of **3at**, related to **Figure 4**

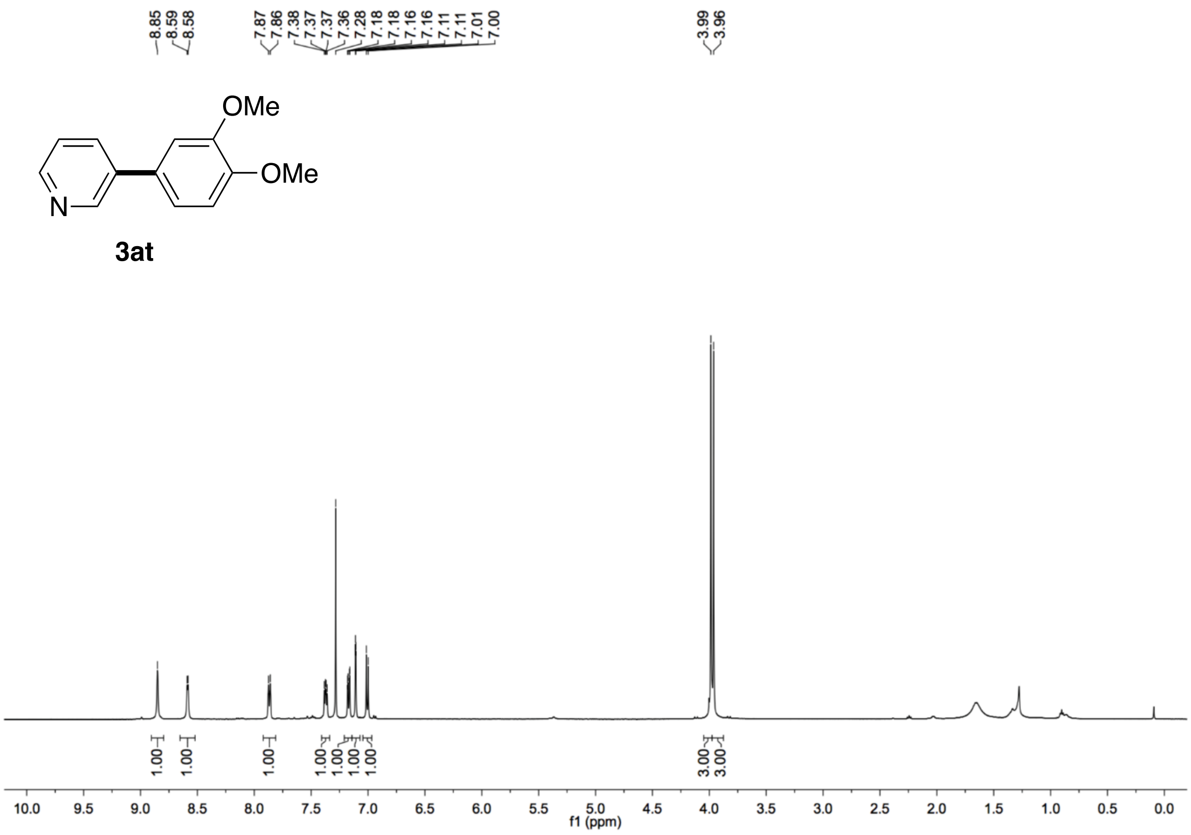

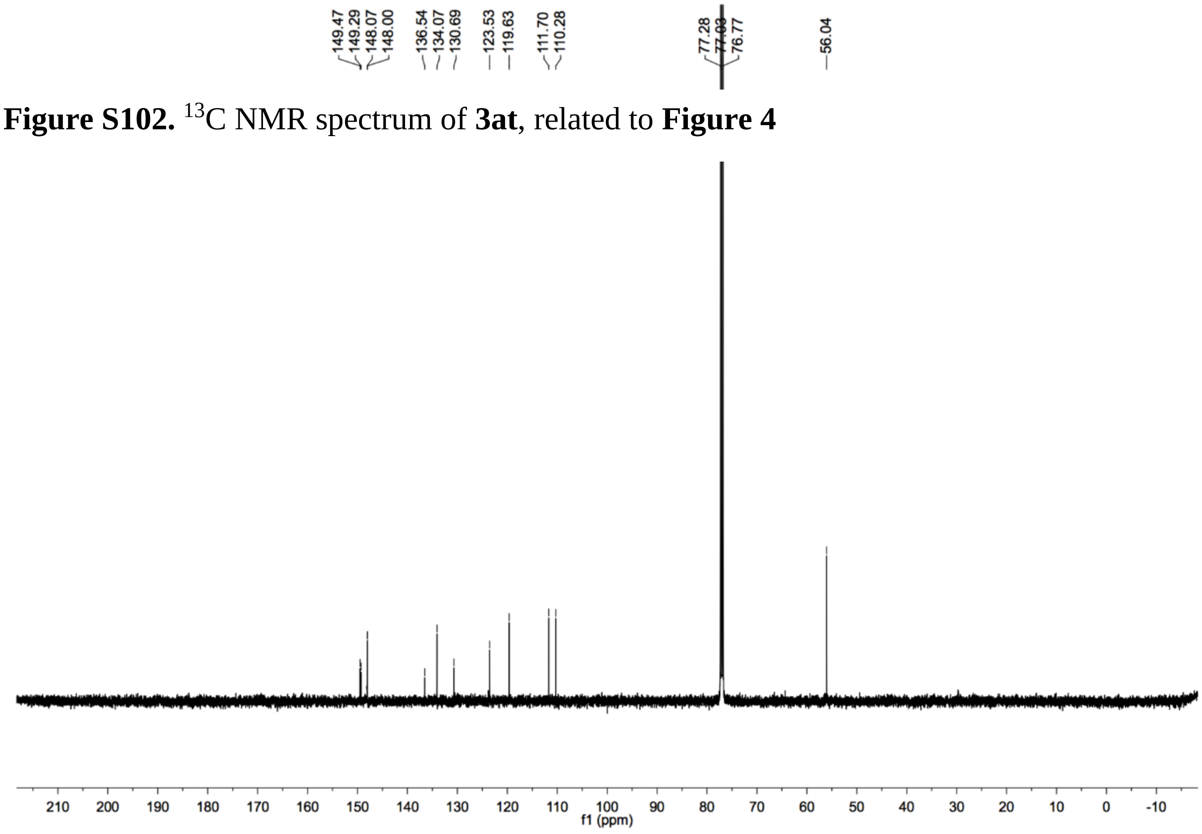

Figure S102. ^{13}C NMR spectrum of **3at**, related to **Figure 4**

Figure S103. ^{1}H NMR spectrum of **3au**, related to **Figure 4**

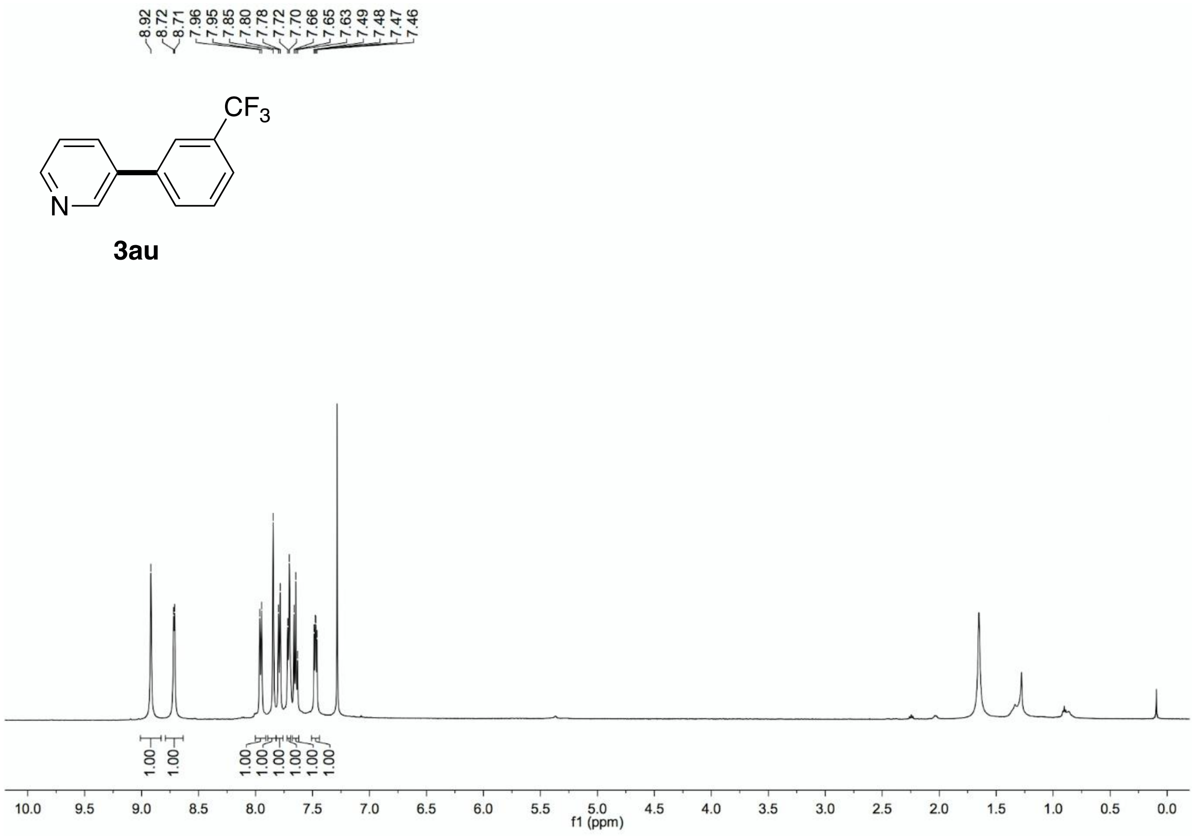

Figure S104. ^{13}C NMR spectrum of **3au**, related to **Figure 4**

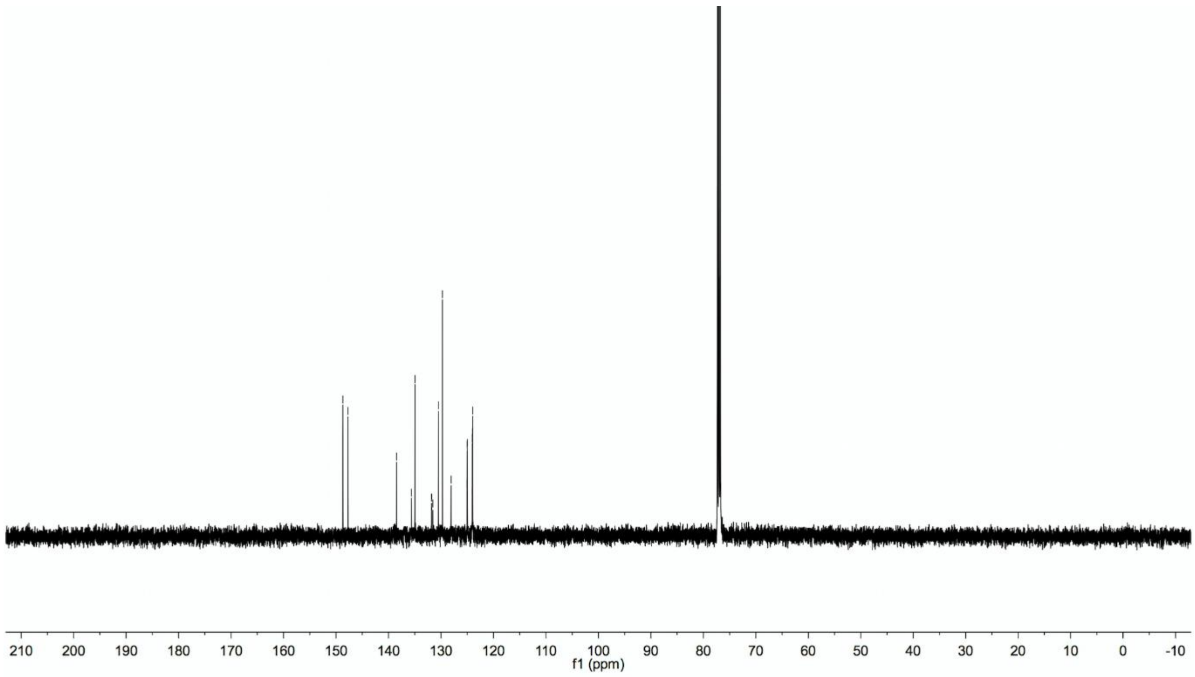

Figure S105. ^{19}F NMR spectrum of **3au**, related to **Figure 4**

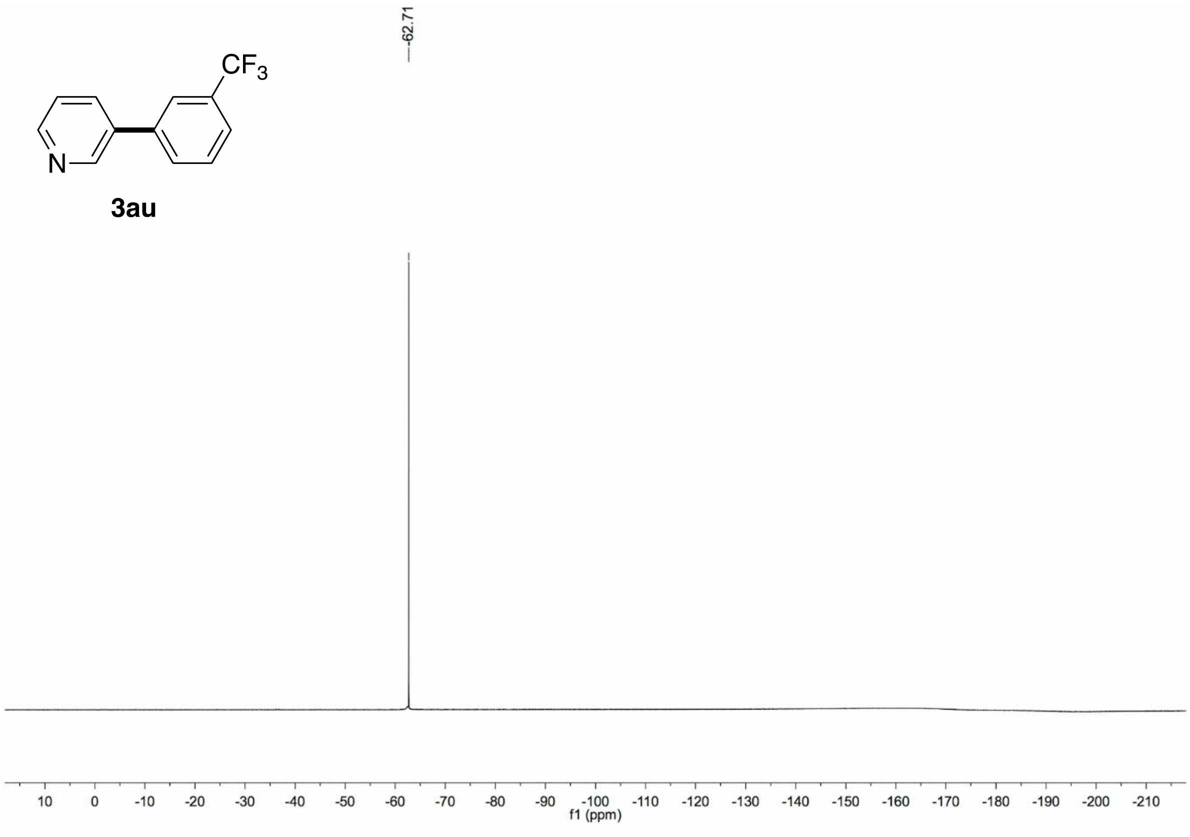

Figure S106. ^{1}H NMR spectrum of **3av**, related to **Figure 4**

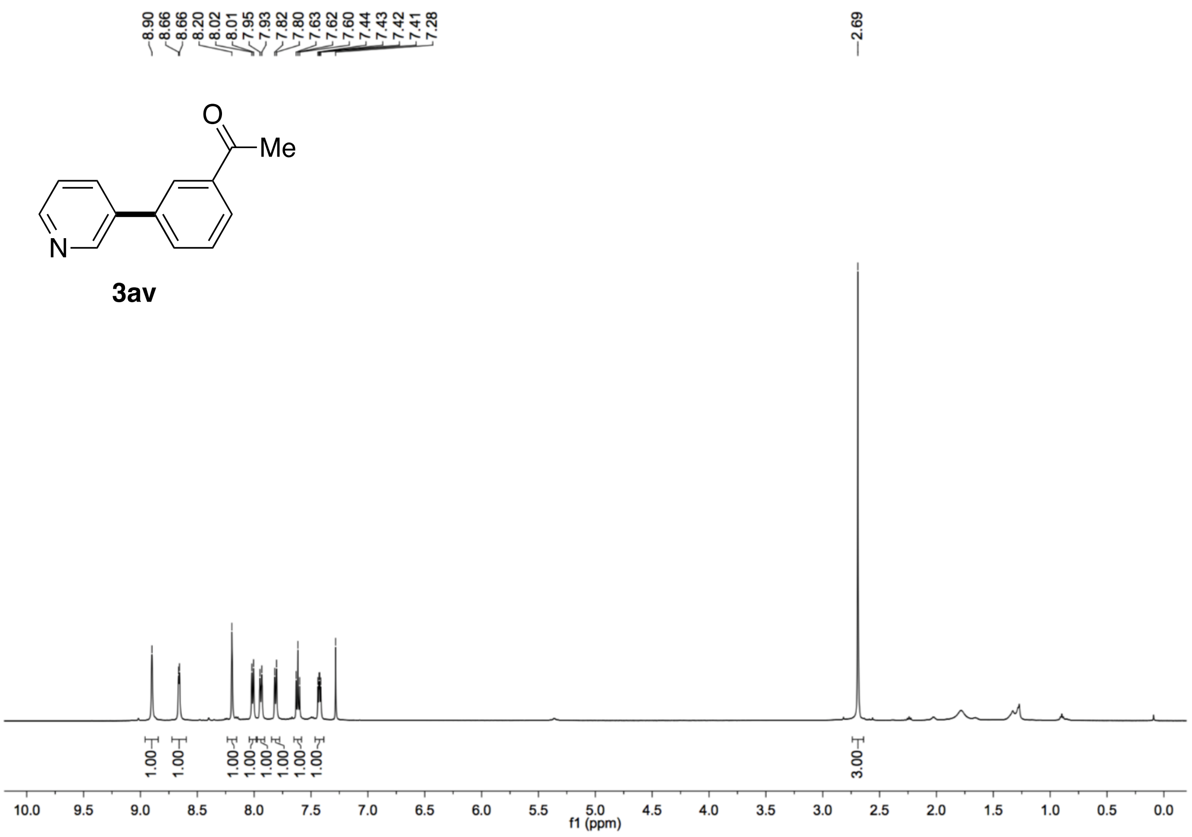

Figure S107. ^{13}C NMR spectrum of **3av**, related to **Figure 4**

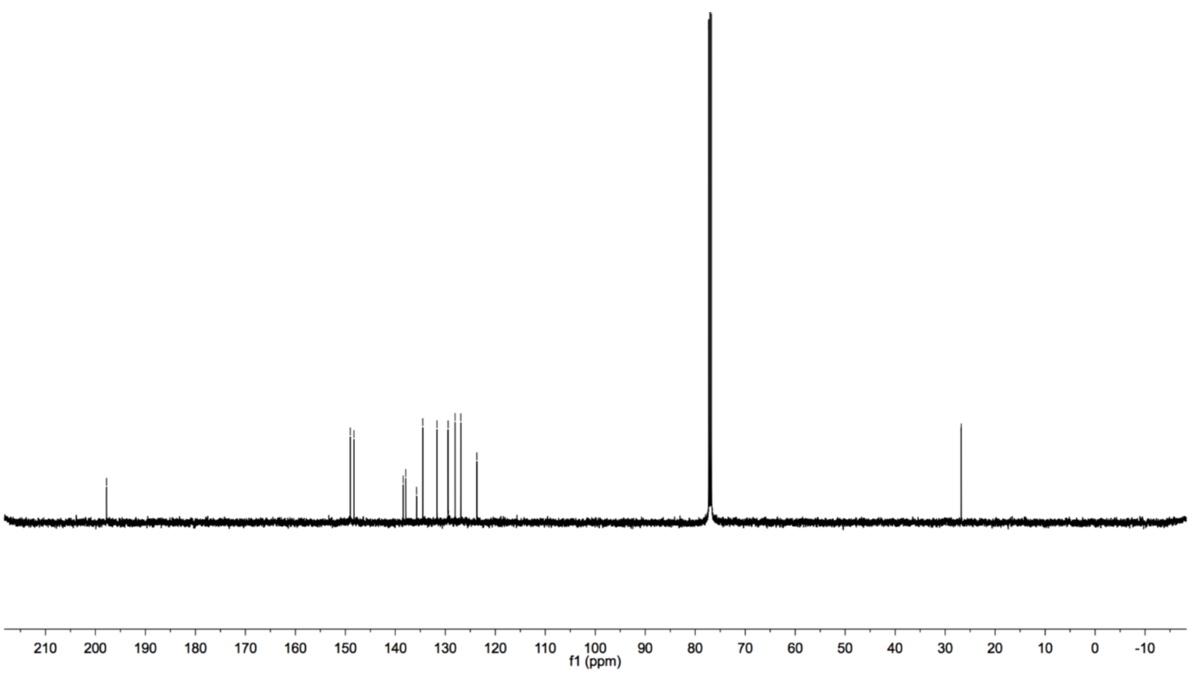

Figure S108. ^{1}H NMR spectrum of **3aw**, related to **Figure 4**

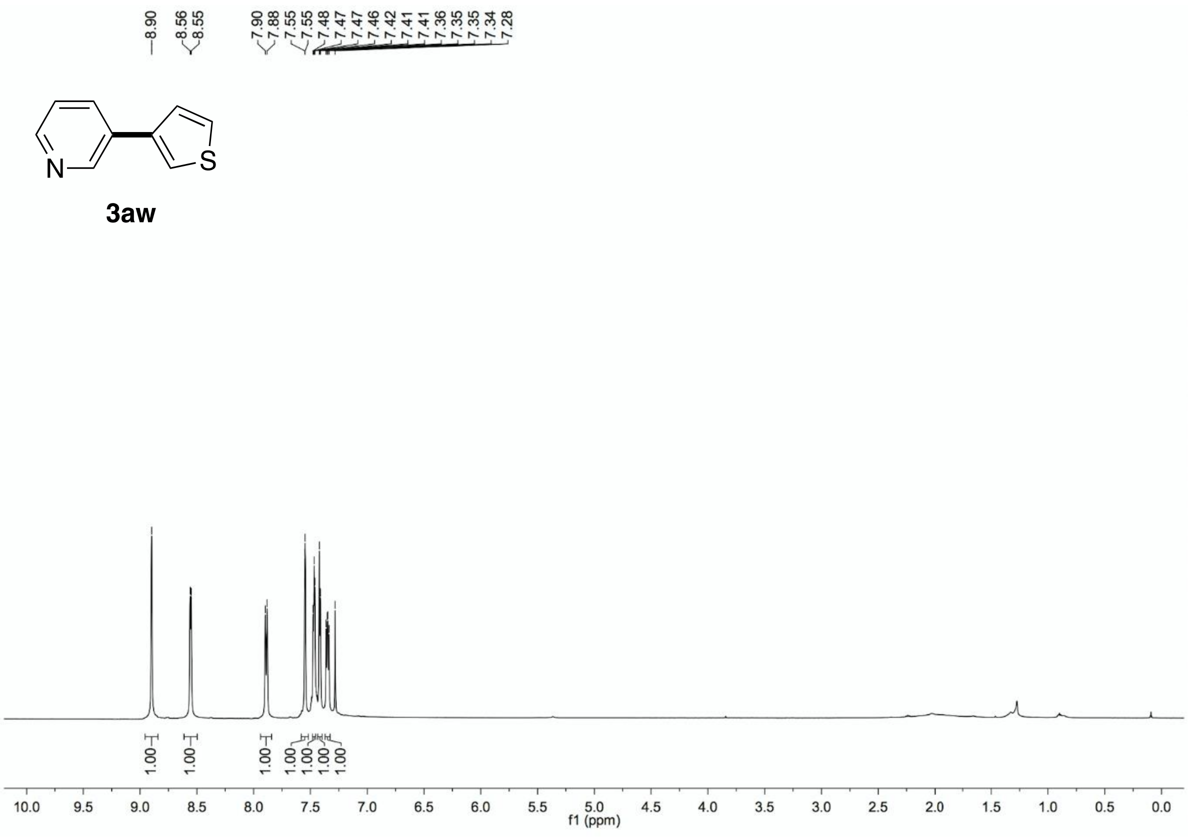

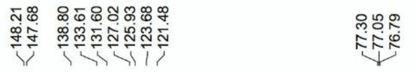

Figure S109. ^{13}C NMR spectrum of **3aw**, related to **Figure 4**

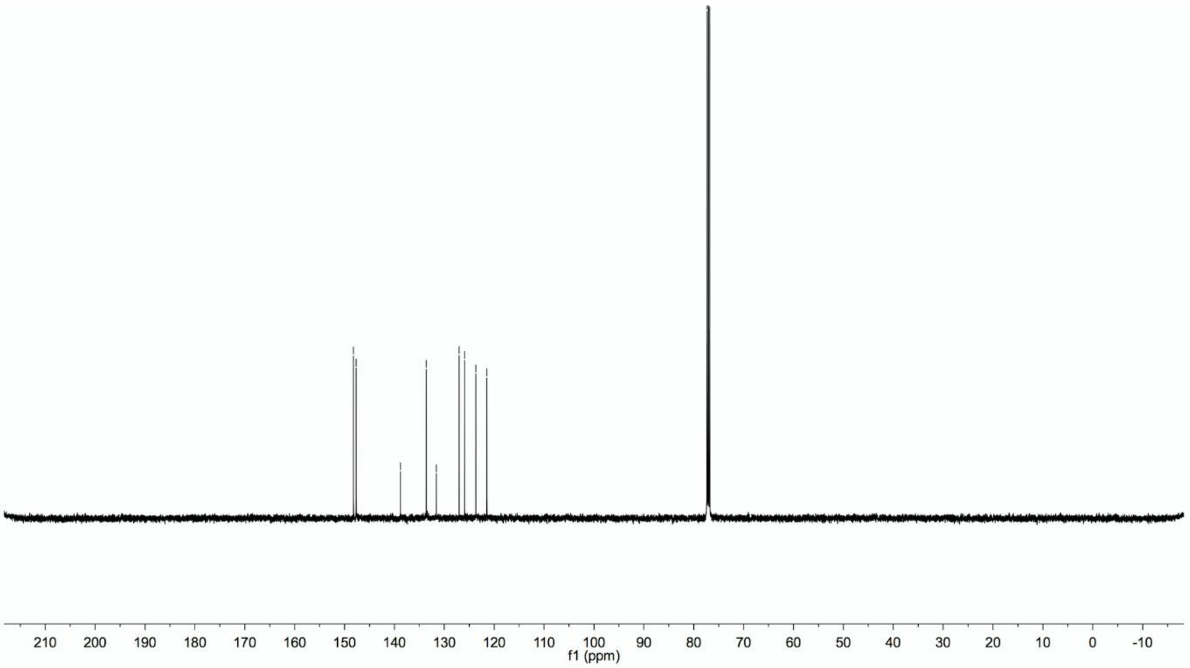

Figure S110. ^{1}H NMR spectrum of **3ax**, related to **Figure 4**

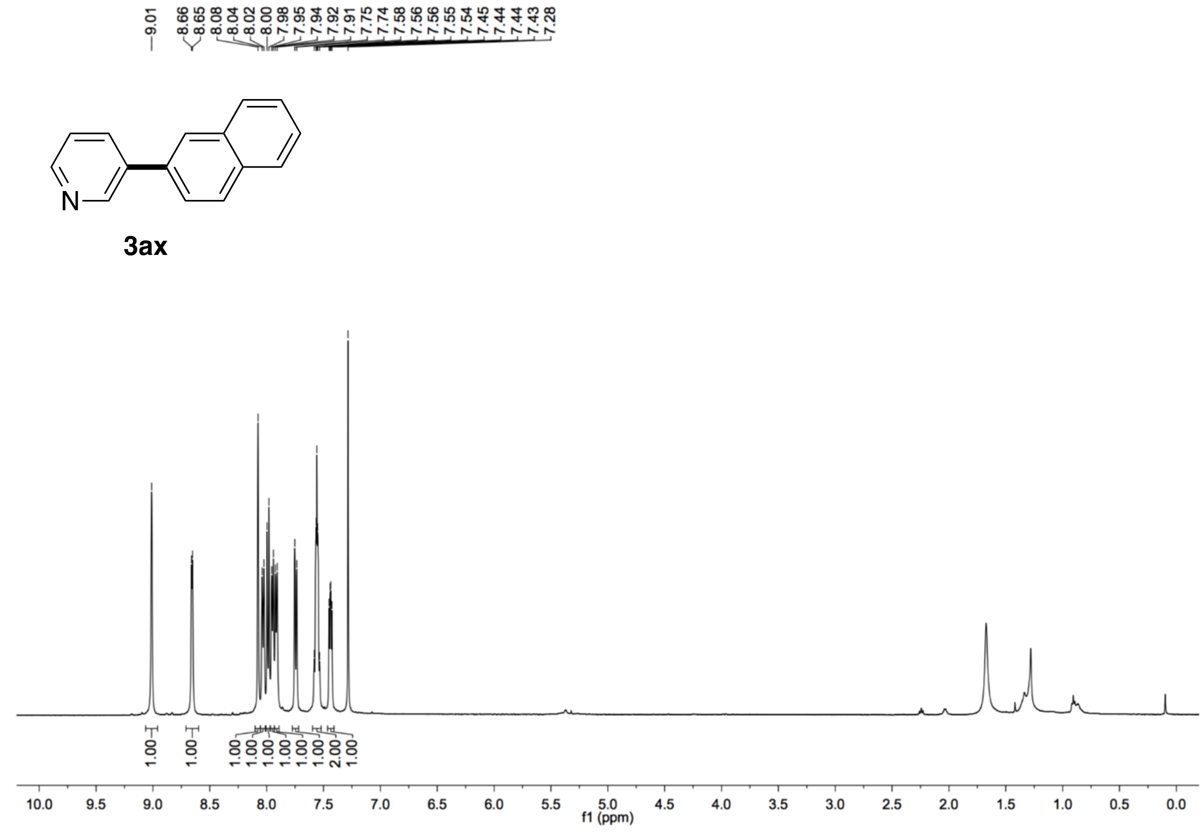

3ax

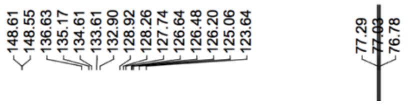

Figure S111. ^{13}C NMR spectrum of **3ax**, related to **Figure 4**

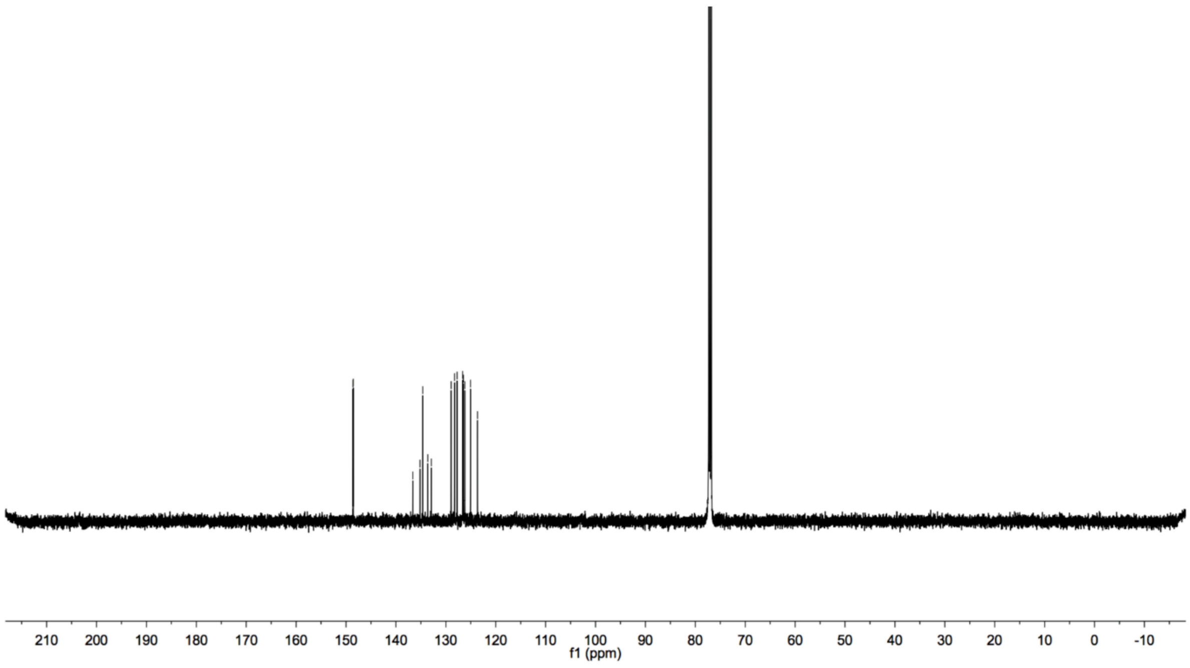

Figure S112. ^{1}H NMR spectrum of **3ay**, related to **Figure 4**

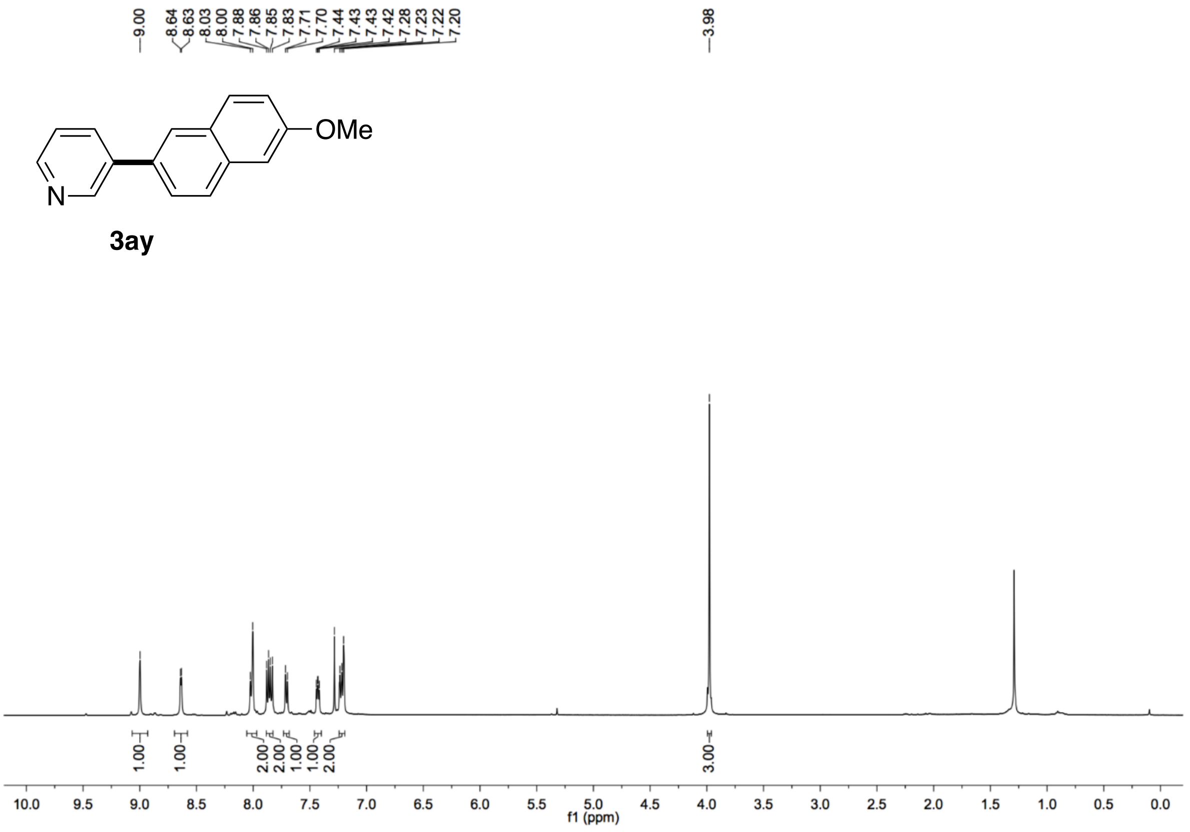

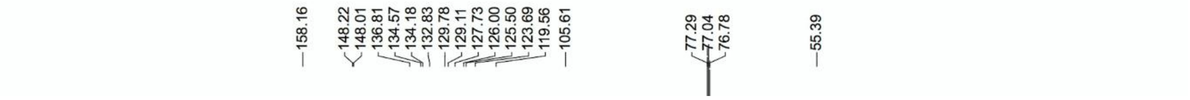

Figure S113. ^{13}C NMR spectrum of **3ay**, related to **Figure 4**

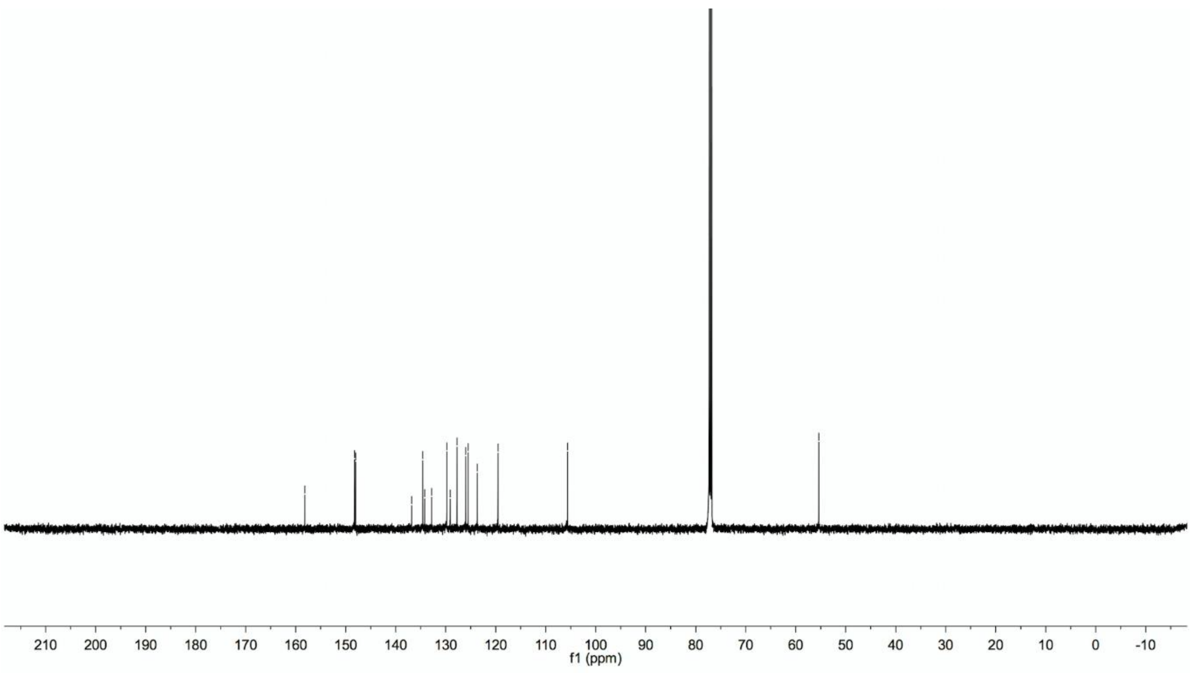

Figure S114. ^{1}H NMR spectrum of **3az**, related to **Figure 4**

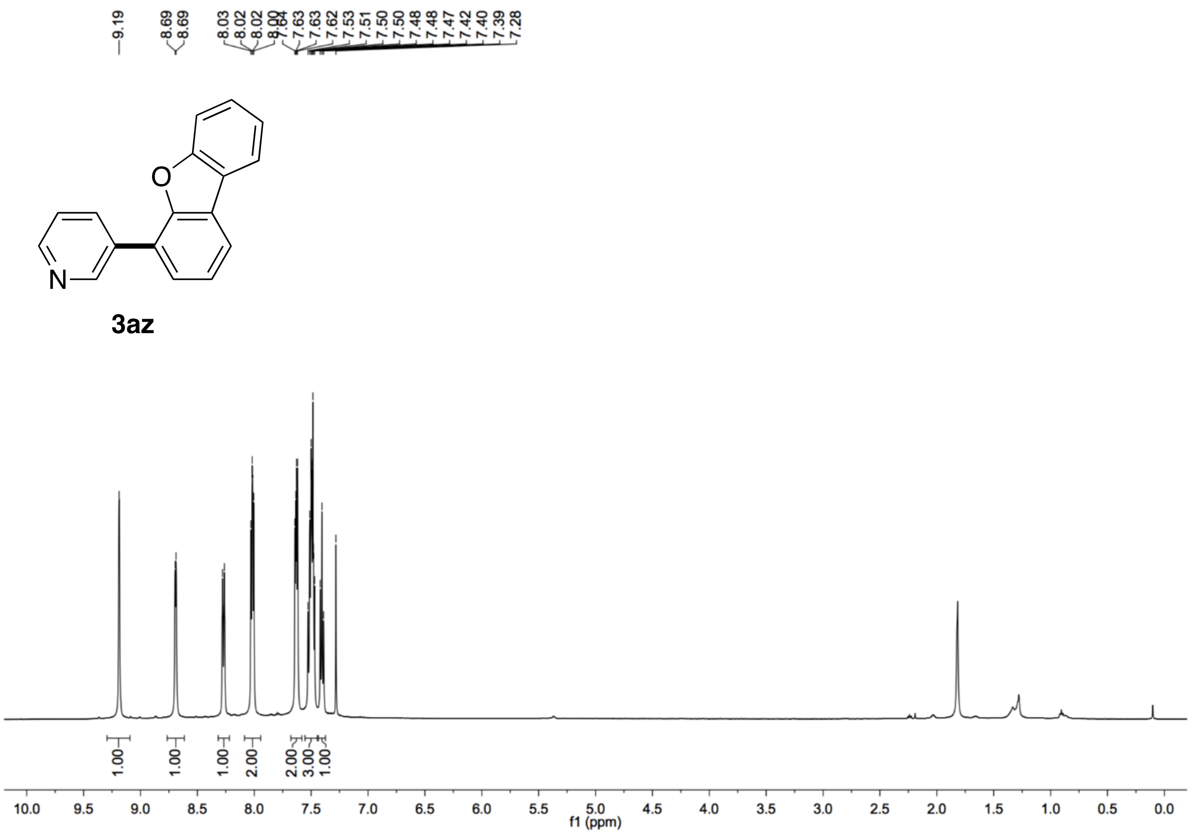

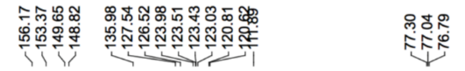

Figure S115. ^{13}C NMR spectrum of **3az**, related to **Figure 4**

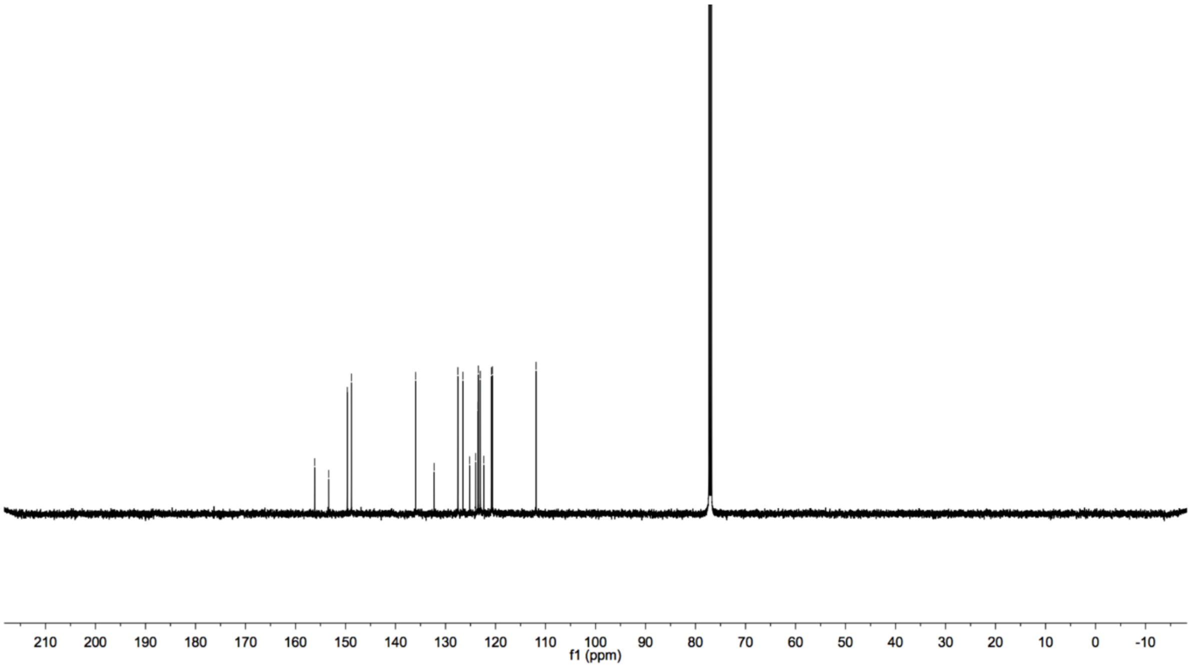

Figure S116. ^{1}H NMR spectrum of **3ba**, related to **Figure 4**

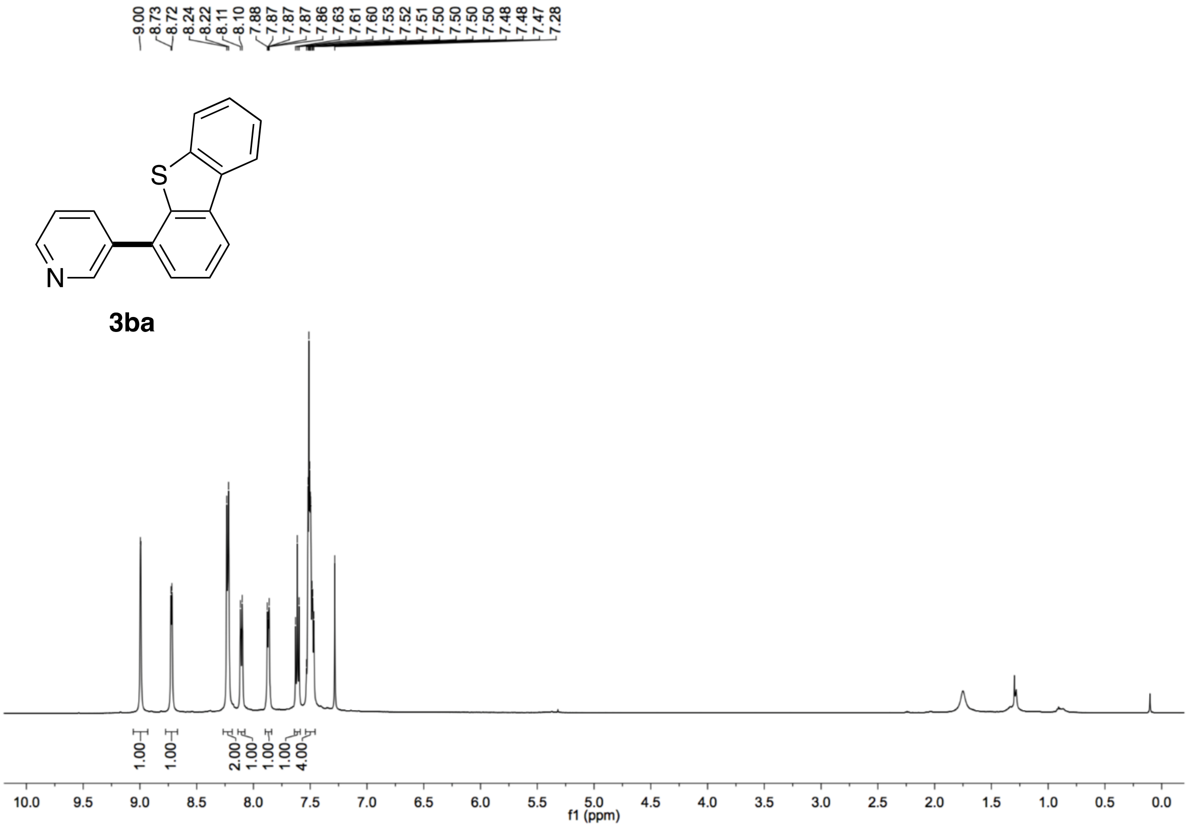

Figure S117. ^{13}C NMR spectrum of **3ba**, related to **Figure 4**

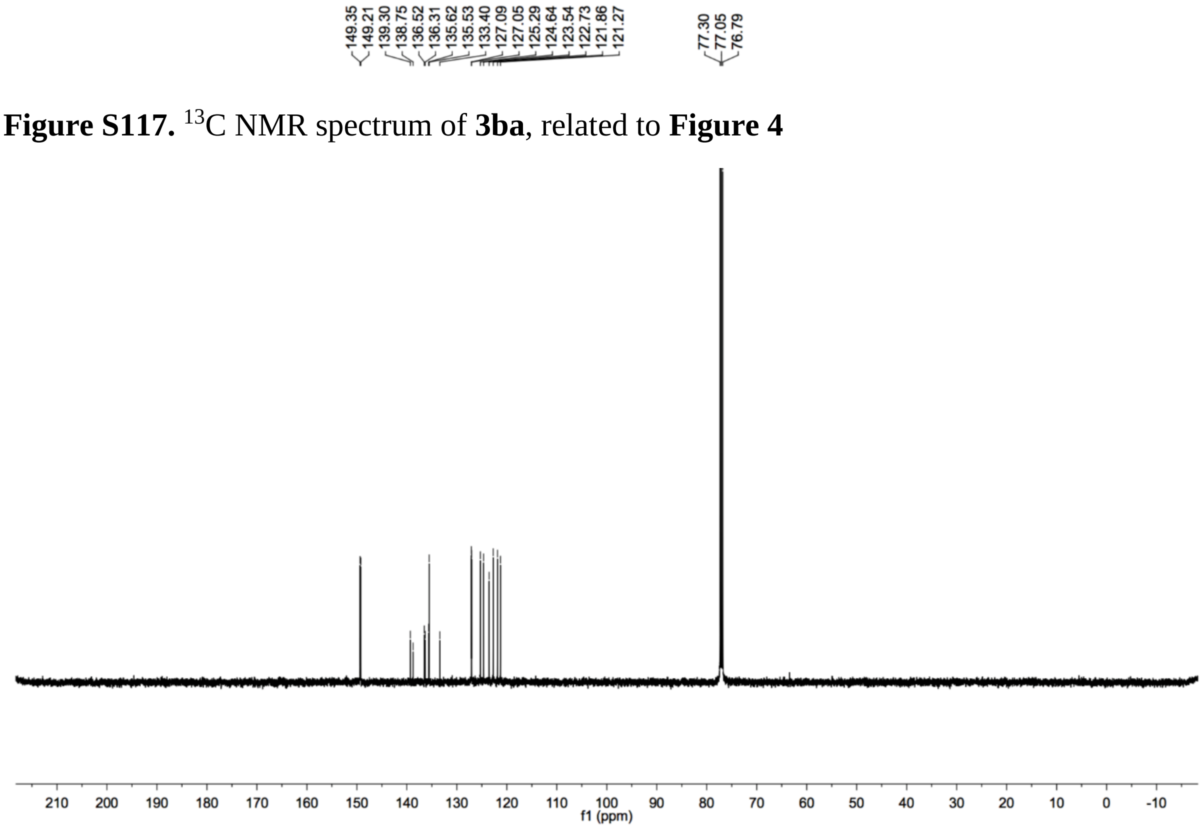

Figure S118. ^{1}H NMR spectrum of **3bb**, related to **Figure 4**

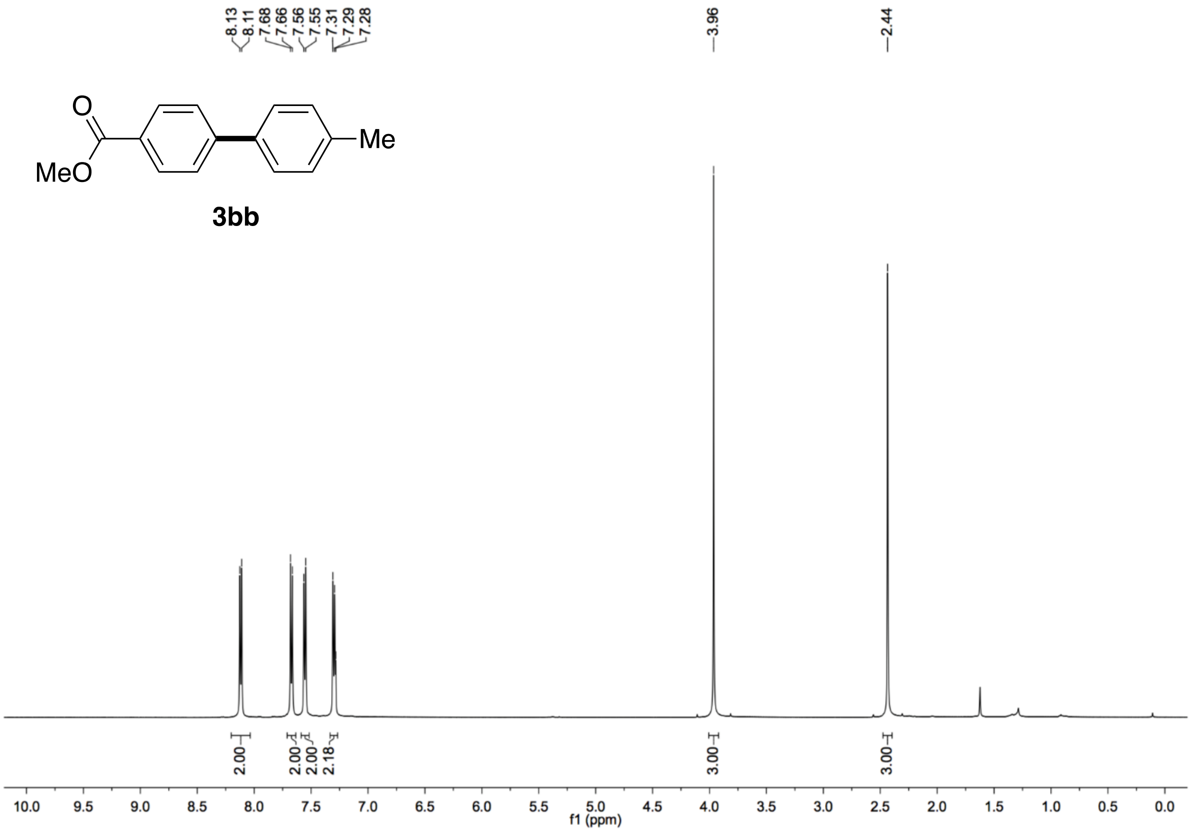

Figure S119. ^{13}C NMR spectrum of **3bb**, related to **Figure 4**

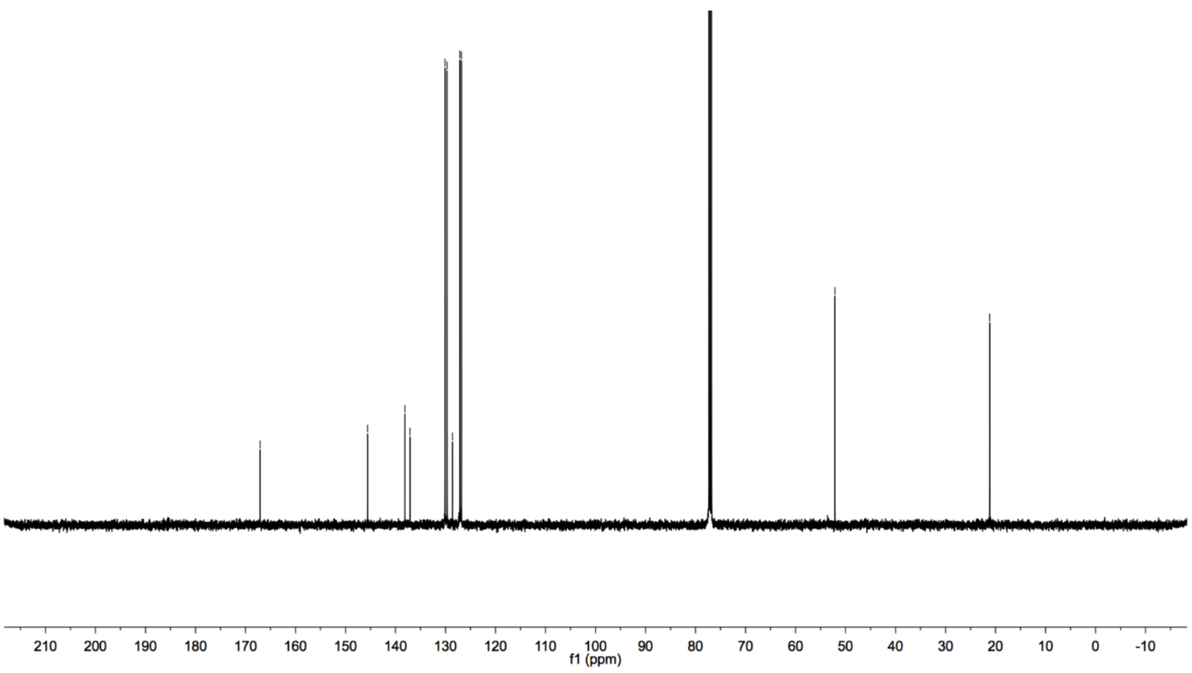

Figure S120. ^{1}H NMR spectrum of **3bc**, related to **Figure 4**

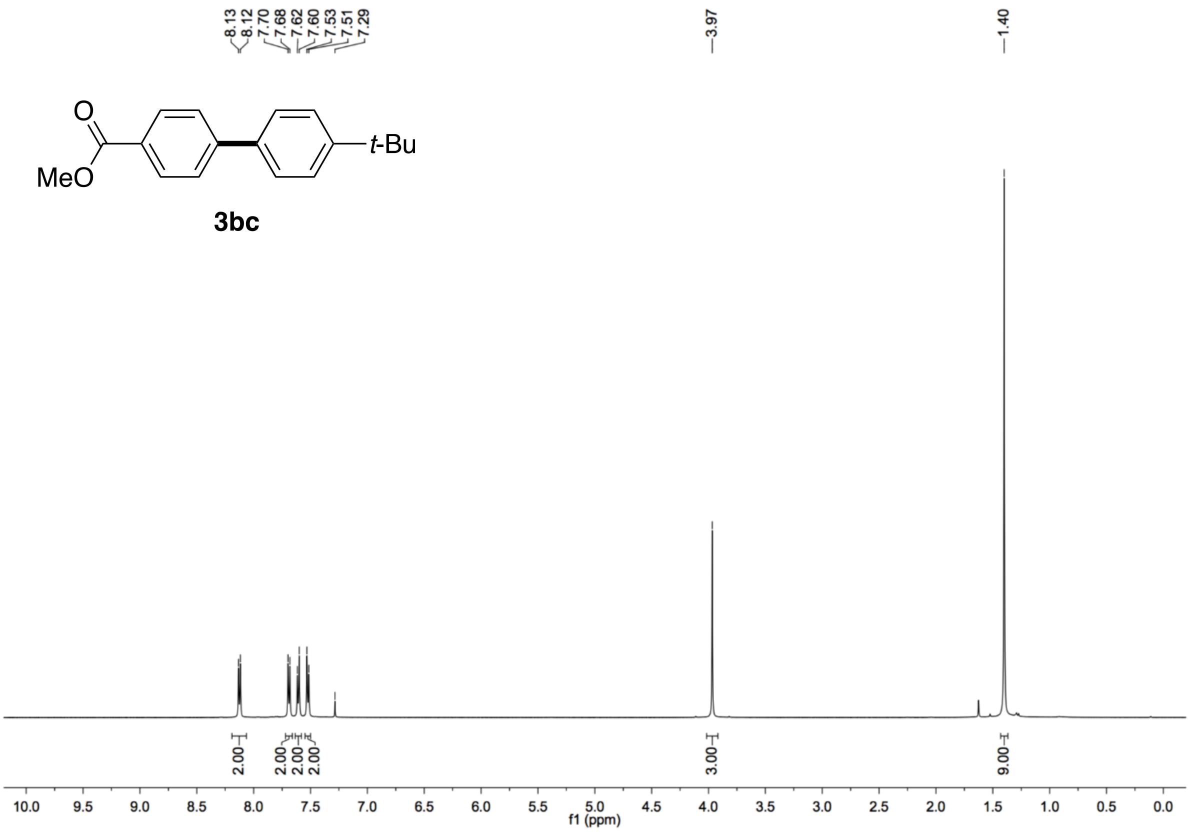

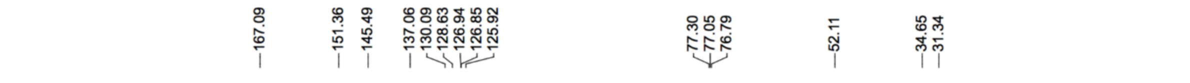

Figure S121. ^{13}C NMR spectrum of **3bc**, related to **Figure 4**

Figure S122. ^{1}H NMR spectrum of **3bd**, related to **Figure 4**

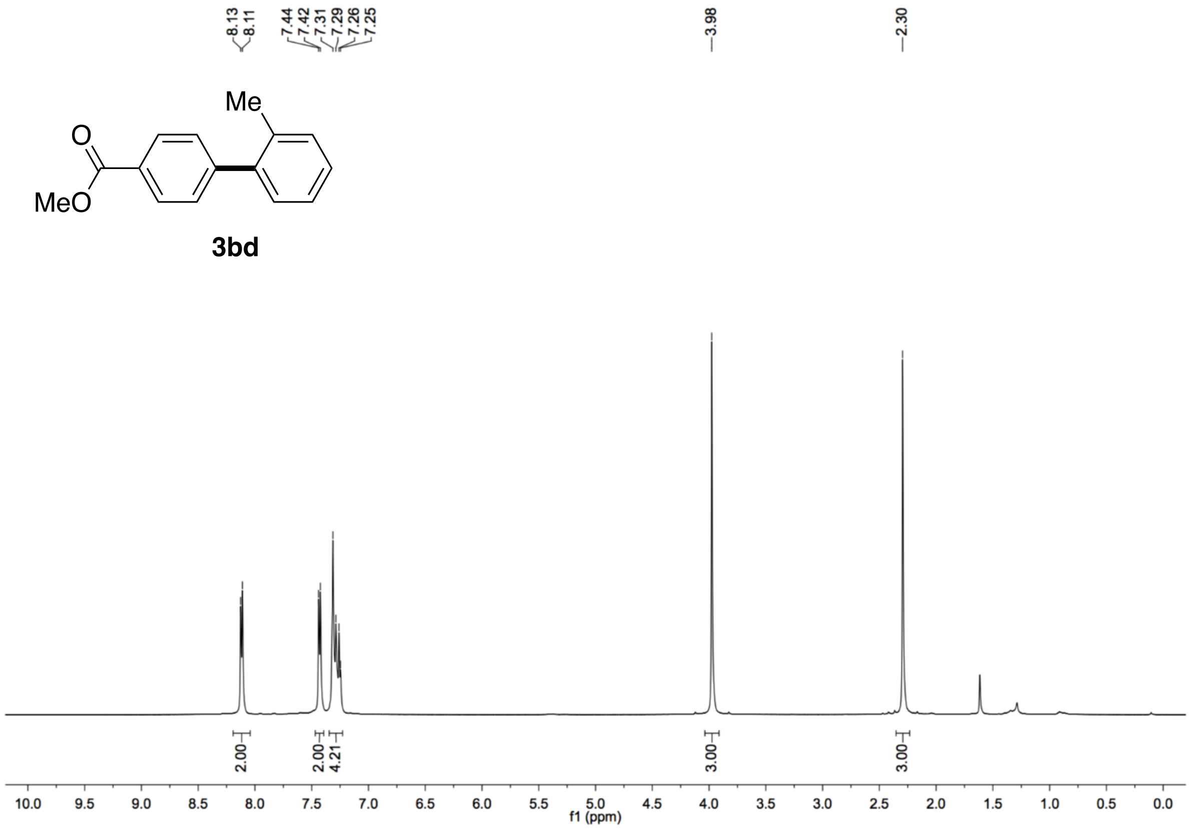

Figure S123. ^{13}C NMR spectrum of **3bd**, related to **Figure 4**

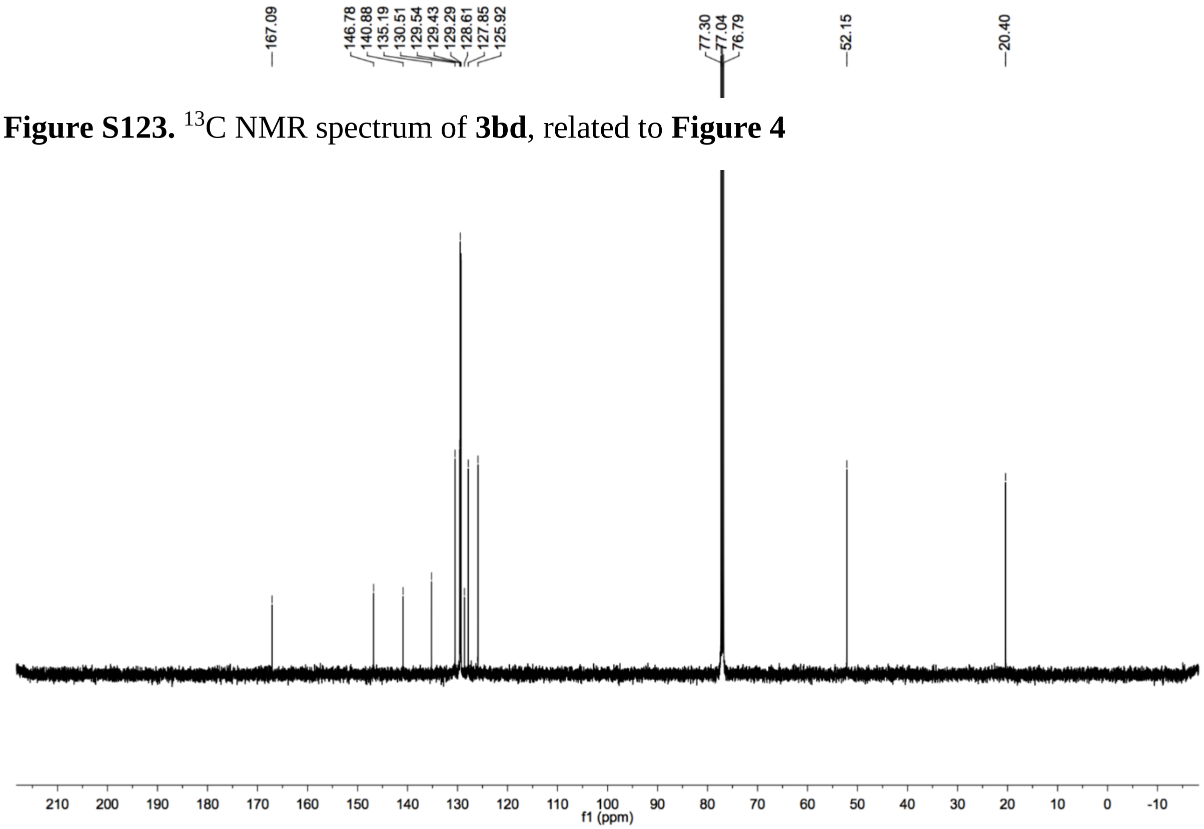

Figure S124. ^{1}H NMR spectrum of **3be**, related to **Figure 4**

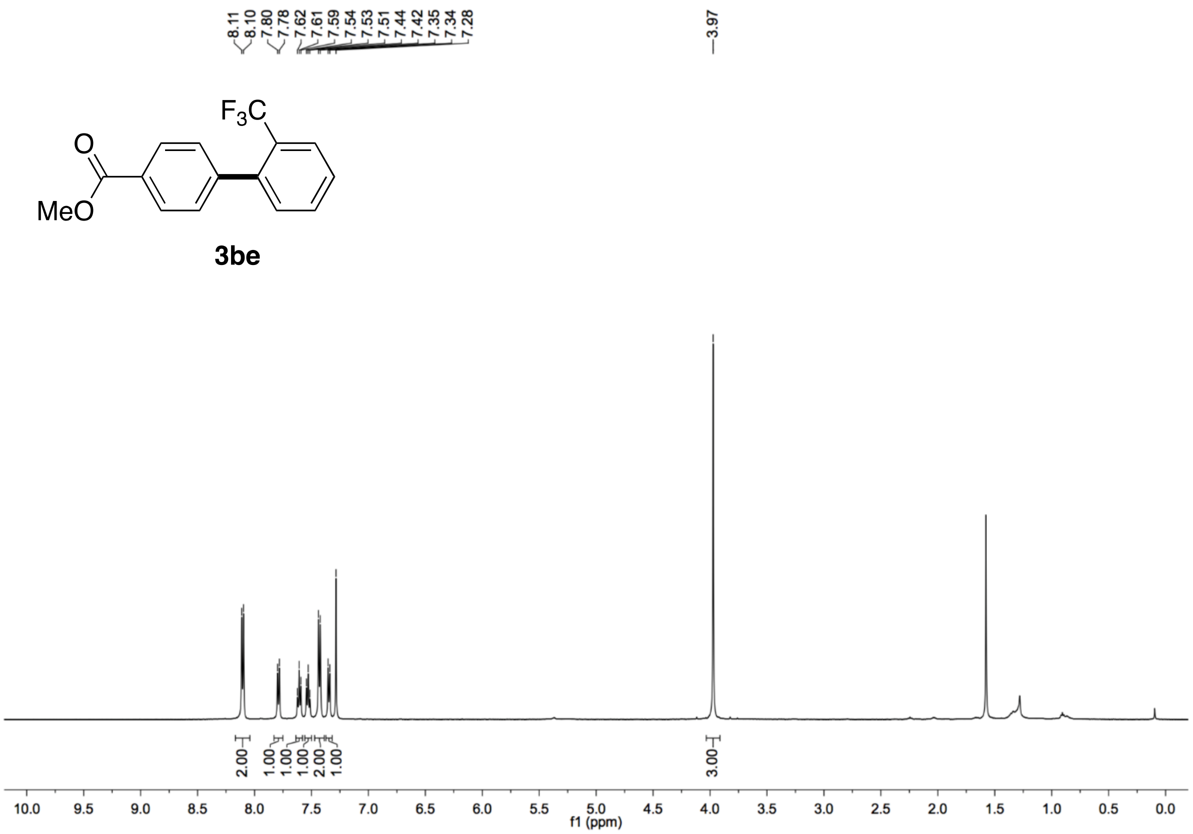

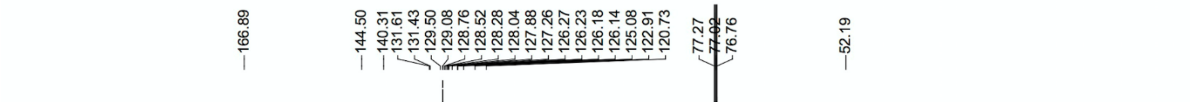

Figure S125. ^{13}C NMR spectrum of **3be**, related to **Figure 4**

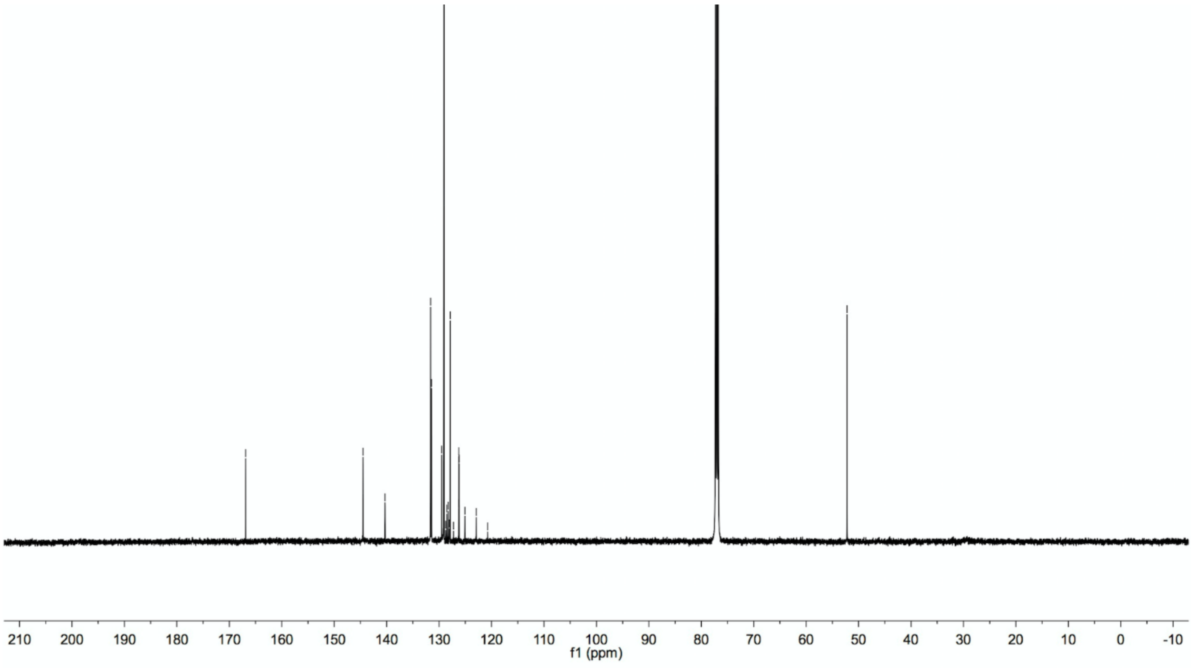

Figure S126. ^{1}H NMR spectrum of **3be**, related to **Figure 4**

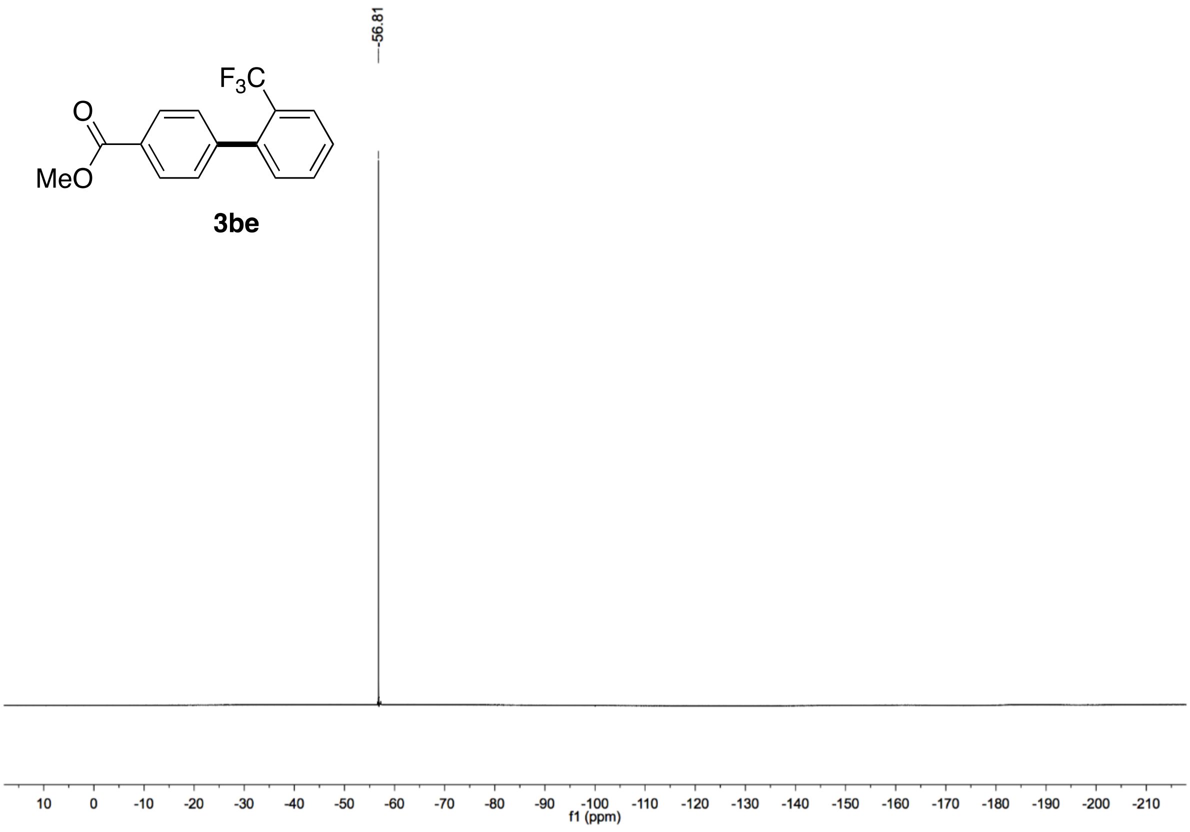

Figure S127. ^{1}H NMR spectrum of **3bf**, related to **Figure 4**

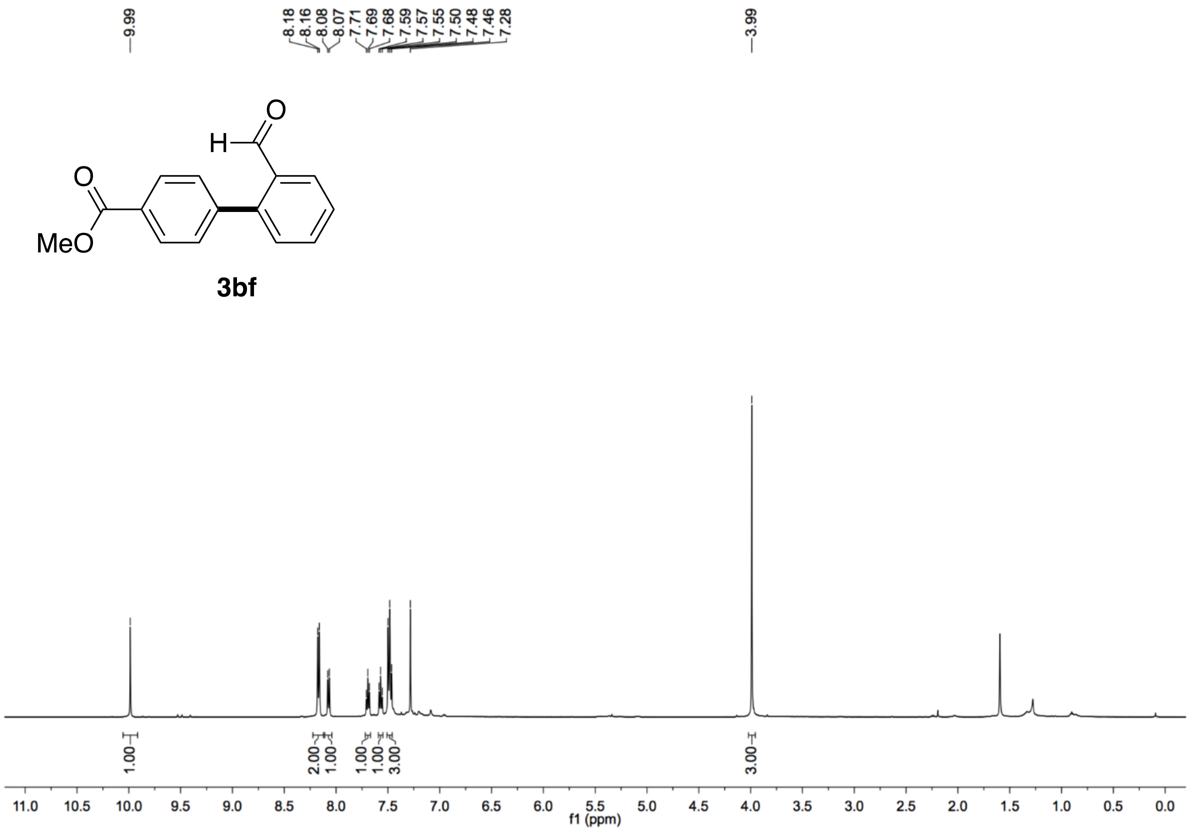

Figure S128. ^{13}C NMR spectrum of **3bf**, related to **Figure 4**

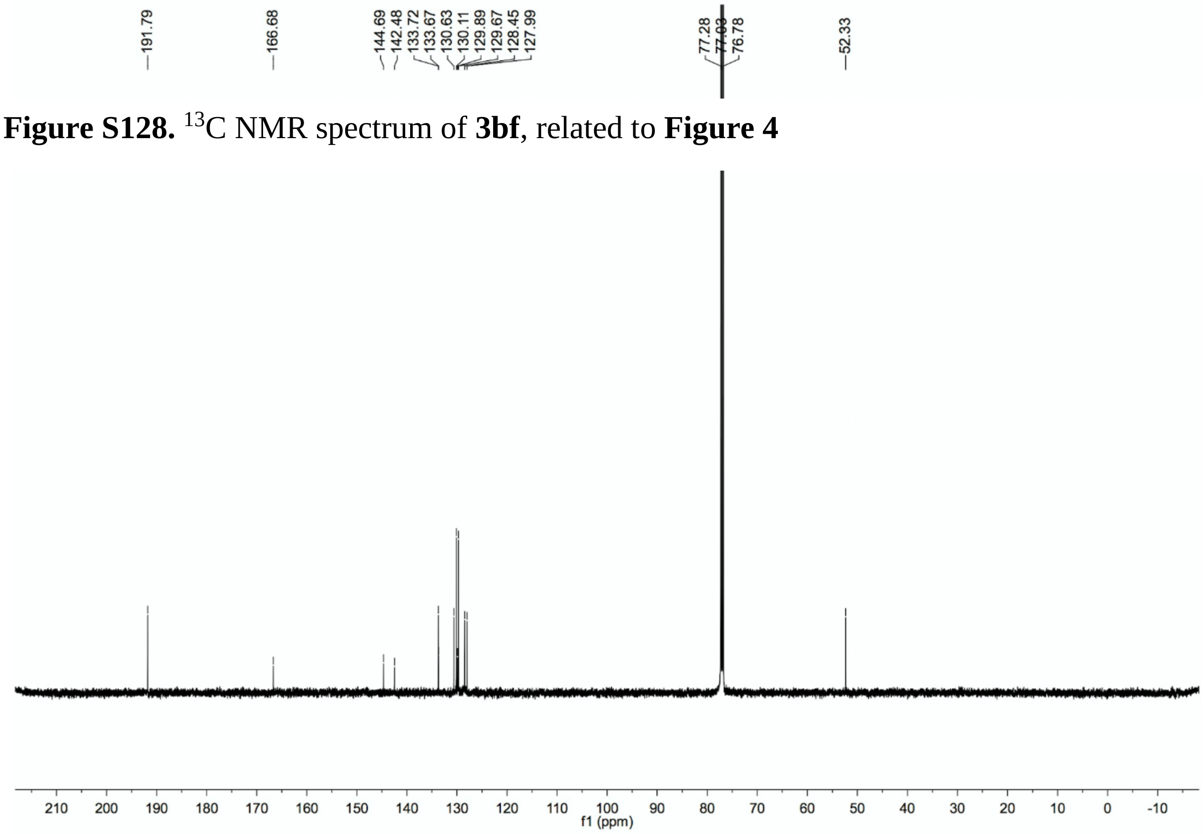

Figure S129. ^{1}H NMR spectrum of **3bg**, related to **Figure 4**

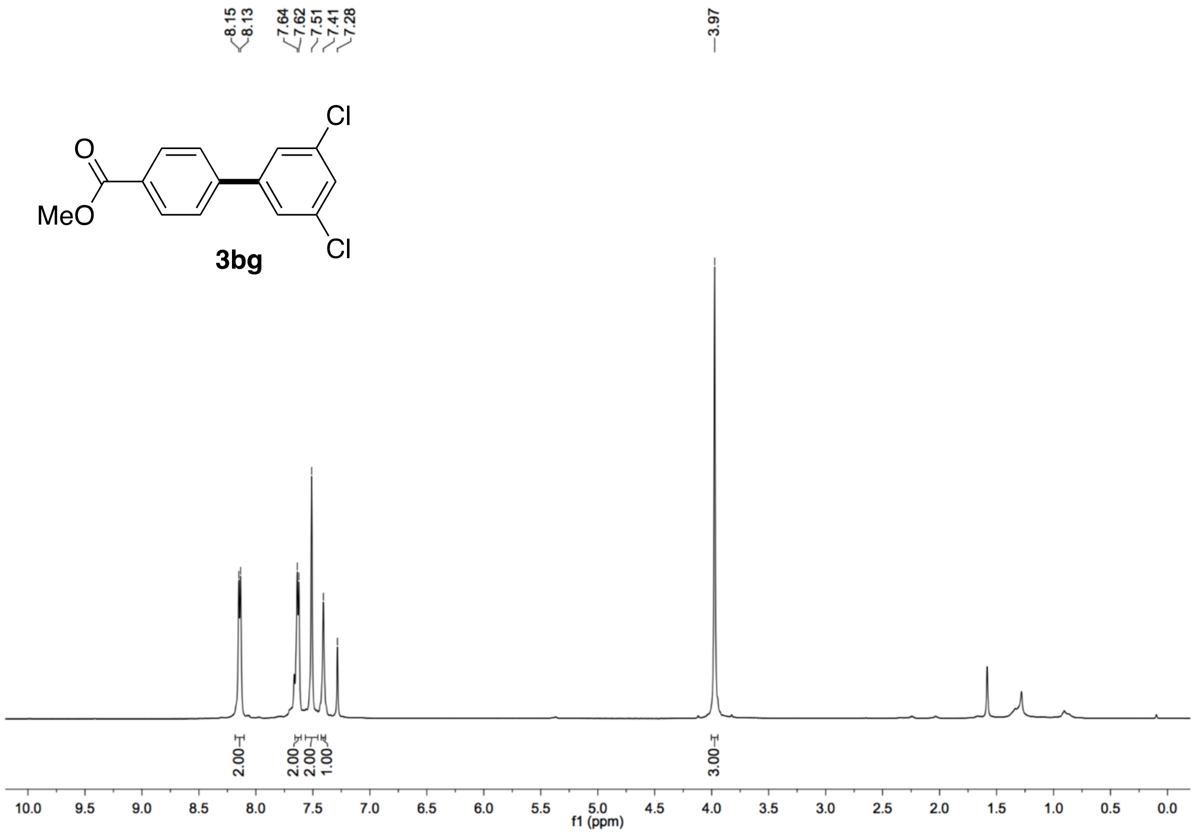

Figure S130. ^{13}C NMR spectrum of **3bg**, related to **Figure 4**

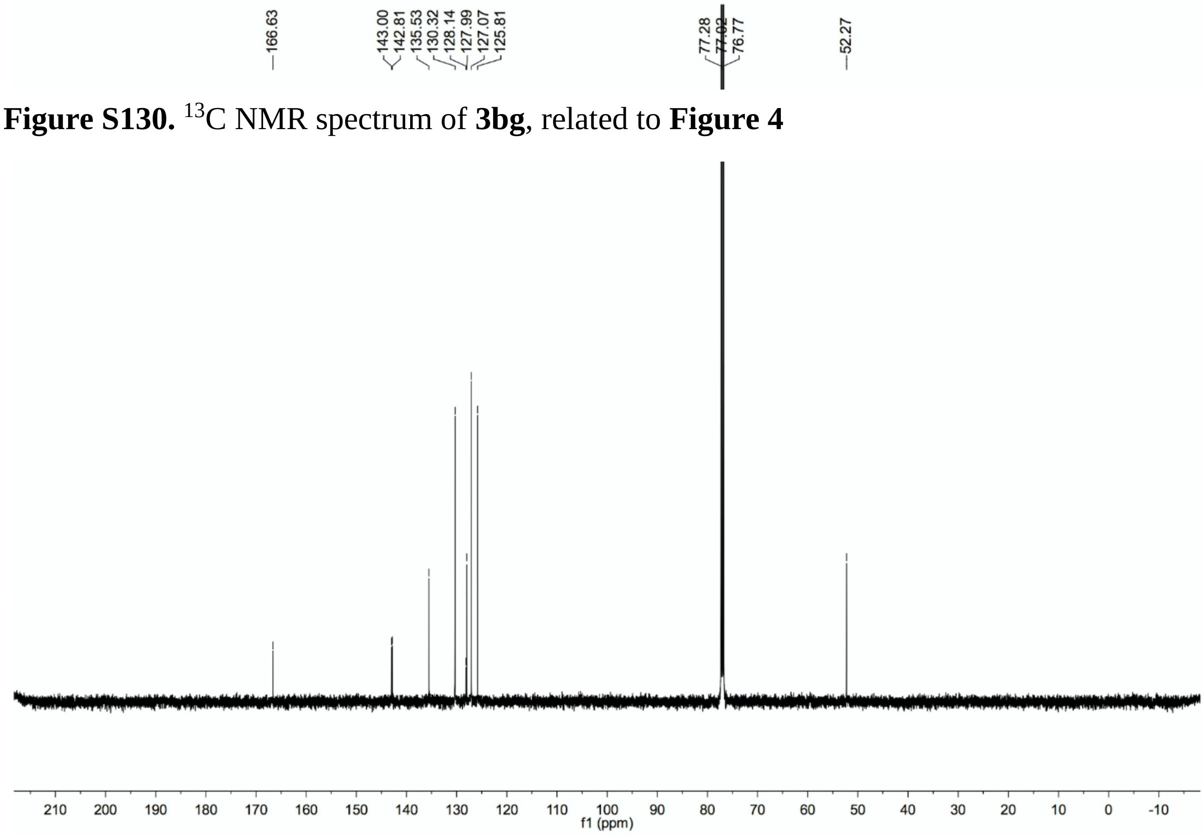

Figure S131. ^{1}H NMR spectrum of **3bh**, related to **Figure 4**

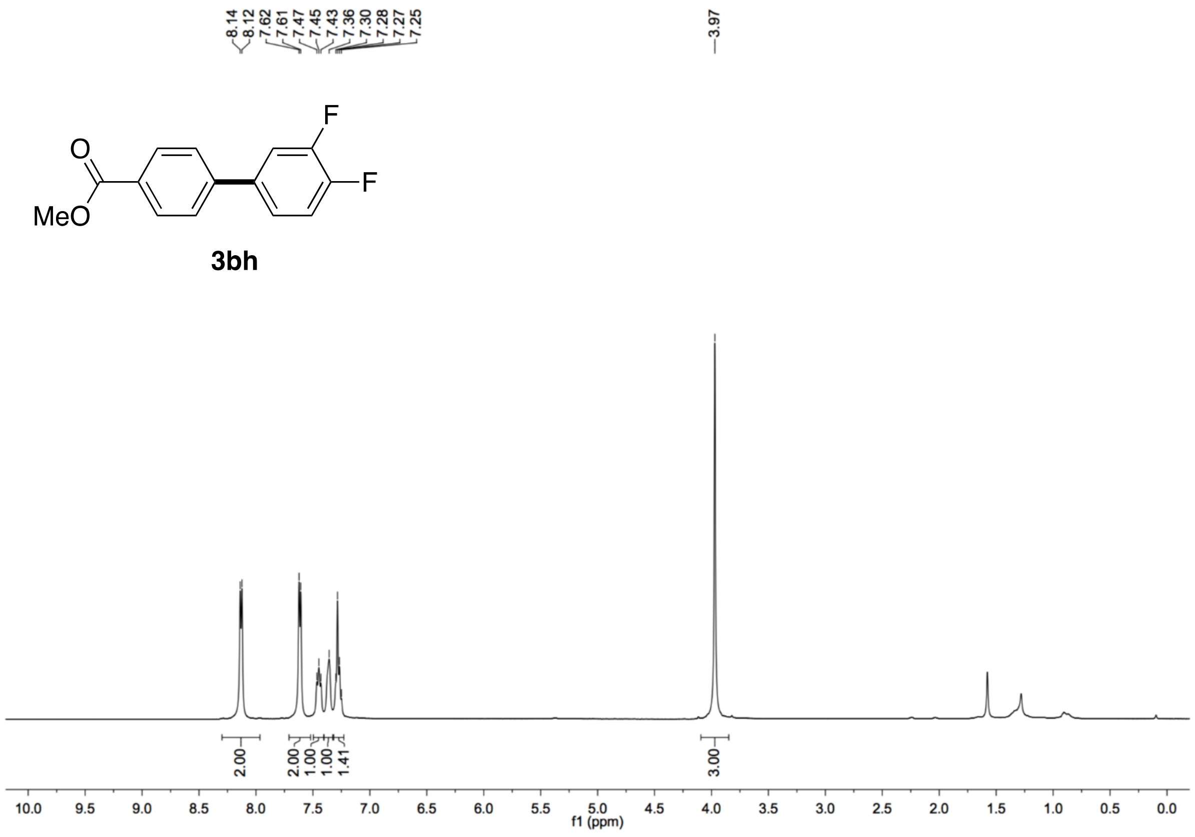

Figure S132. ^{13}C NMR spectrum of **3bh**, related to **Figure 4**

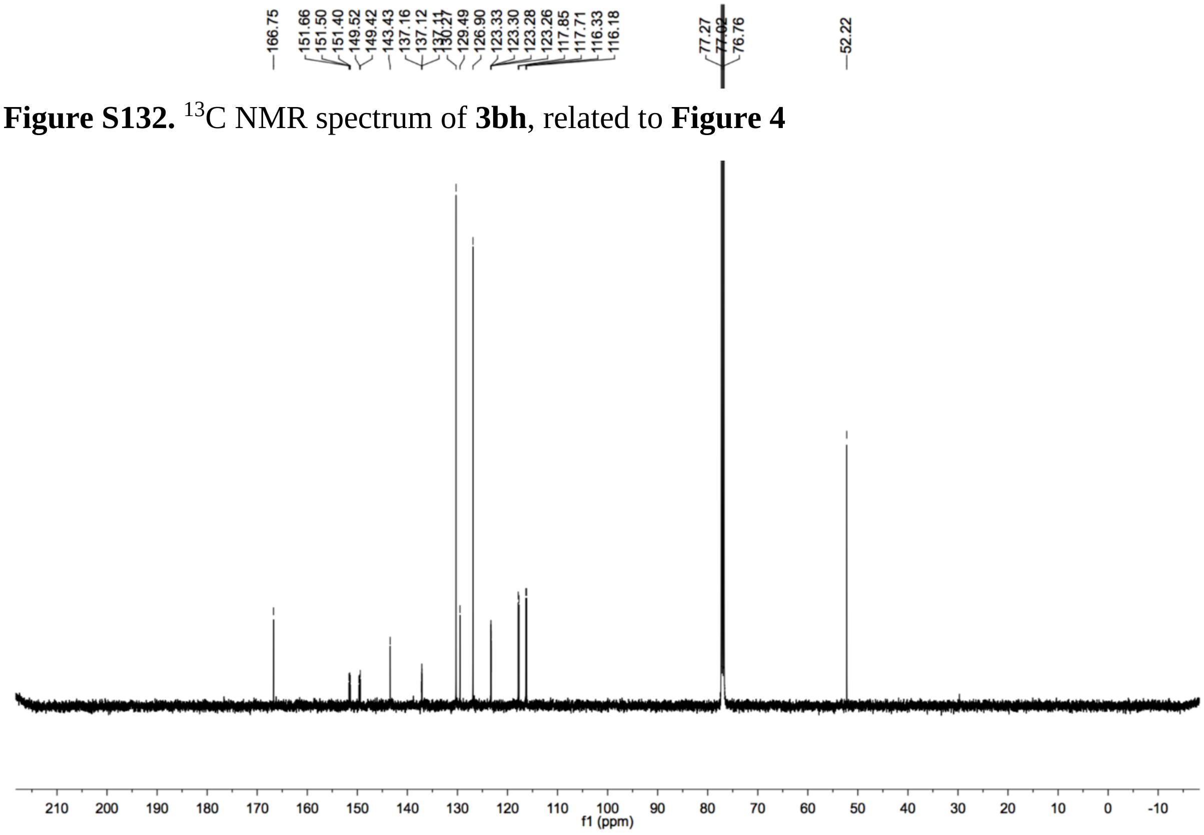

Figure S133. ^{19}F NMR spectrum of **3bh**, related to **Figure 4**

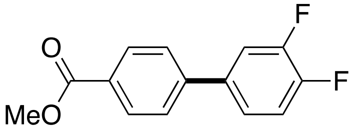

3bh

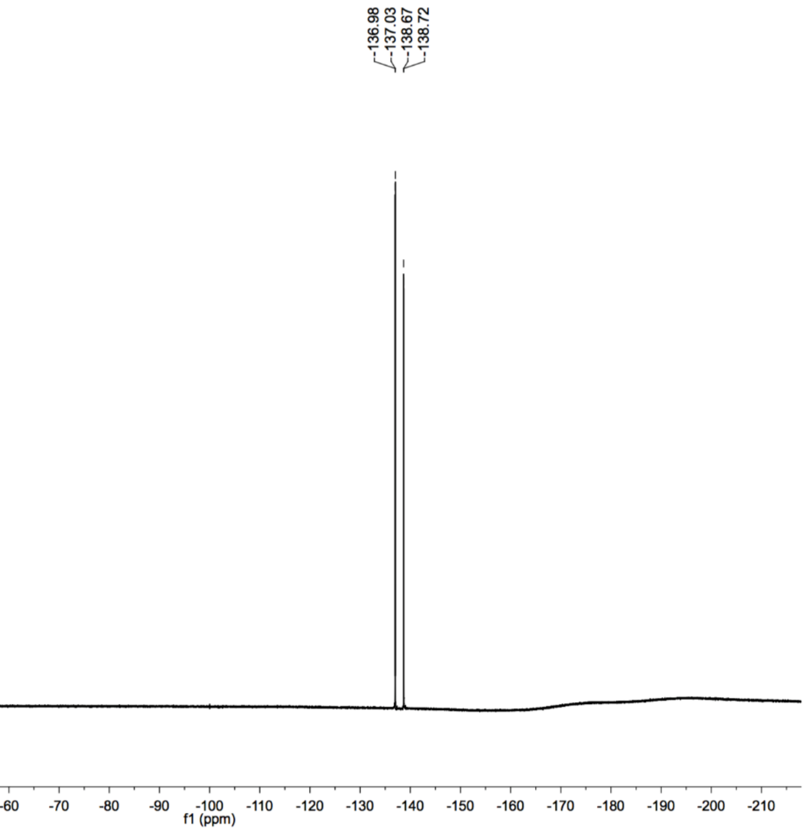

Figure S134. ^{1}H NMR spectrum of **3bi**, related to **Figure 4**

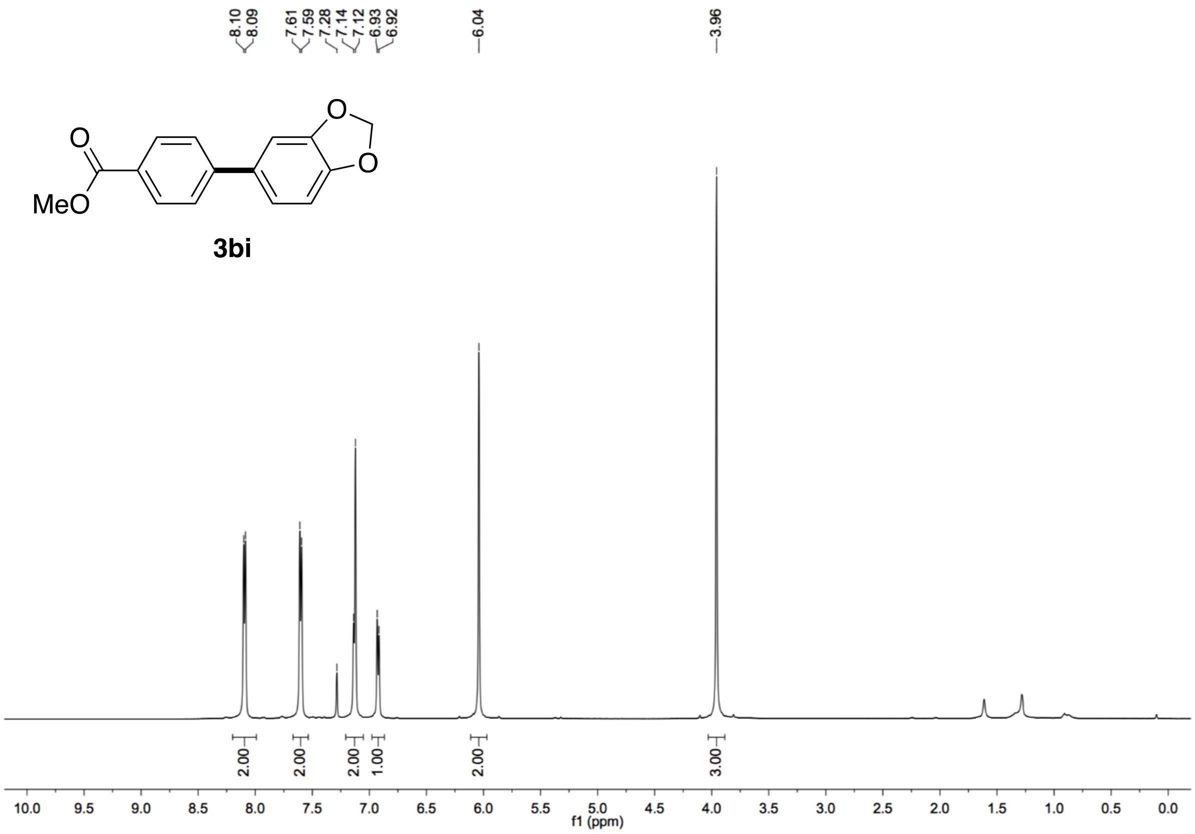

Figure S135. ^{13}C NMR spectrum of **3bi**, related to **Figure 4**

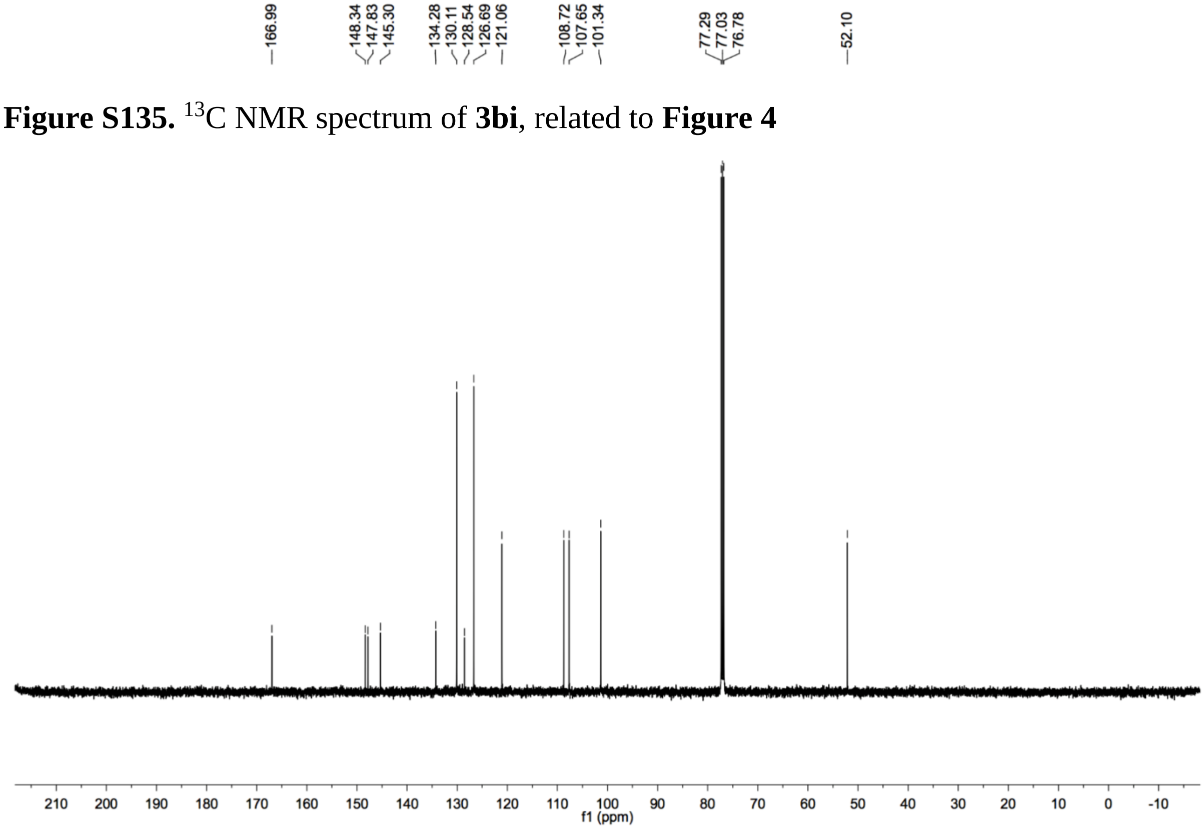

Figure S136. ^{1}H NMR spectrum of **3bj**, related to **Figure 4**

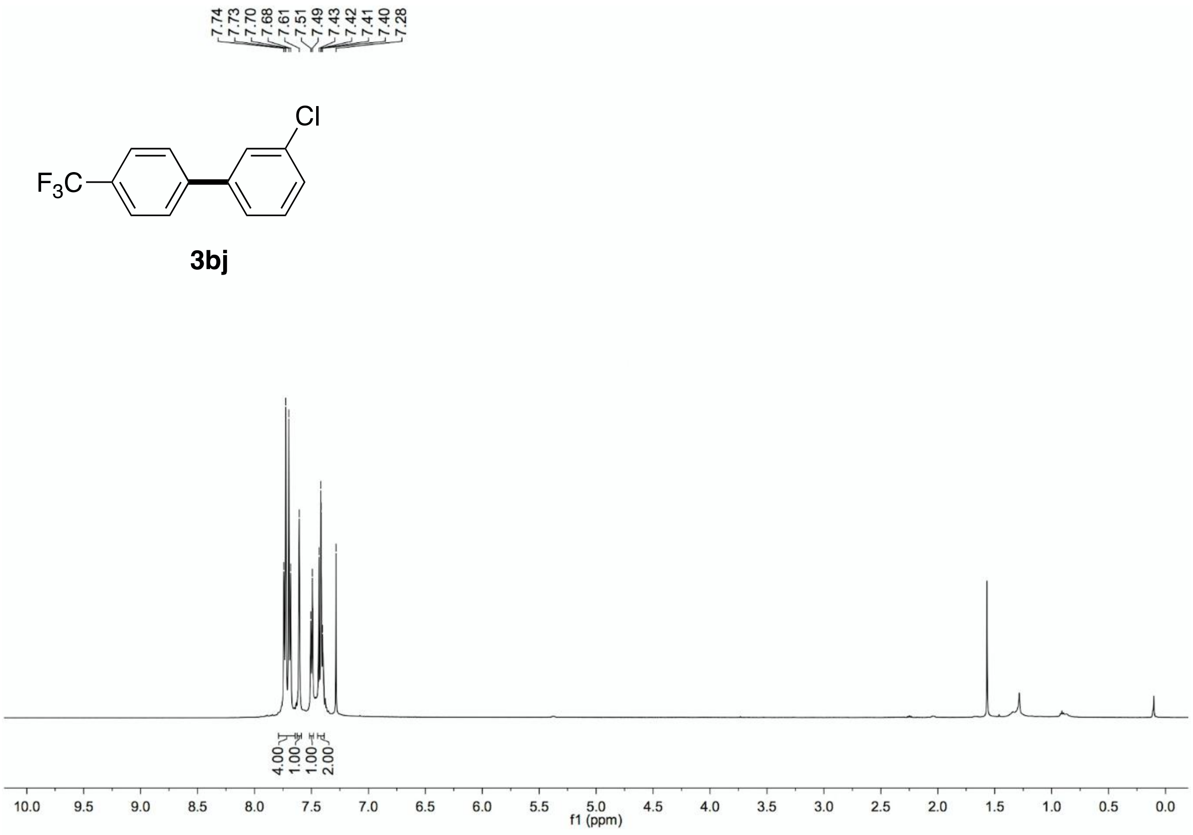

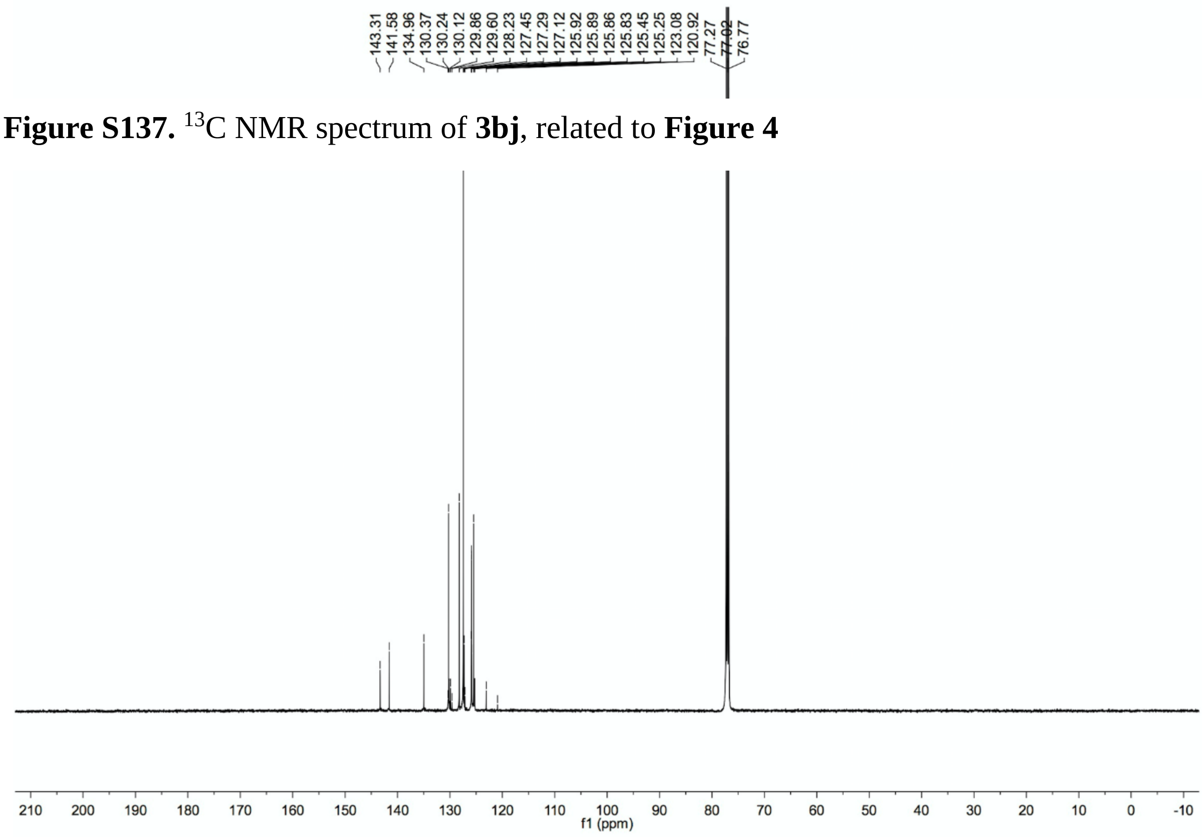

Figure S137. ^{13}C NMR spectrum of **3bj**, related to **Figure 4**

Figure S138. ^{1}H NMR spectrum of **3bj**, related to **Figure 4**

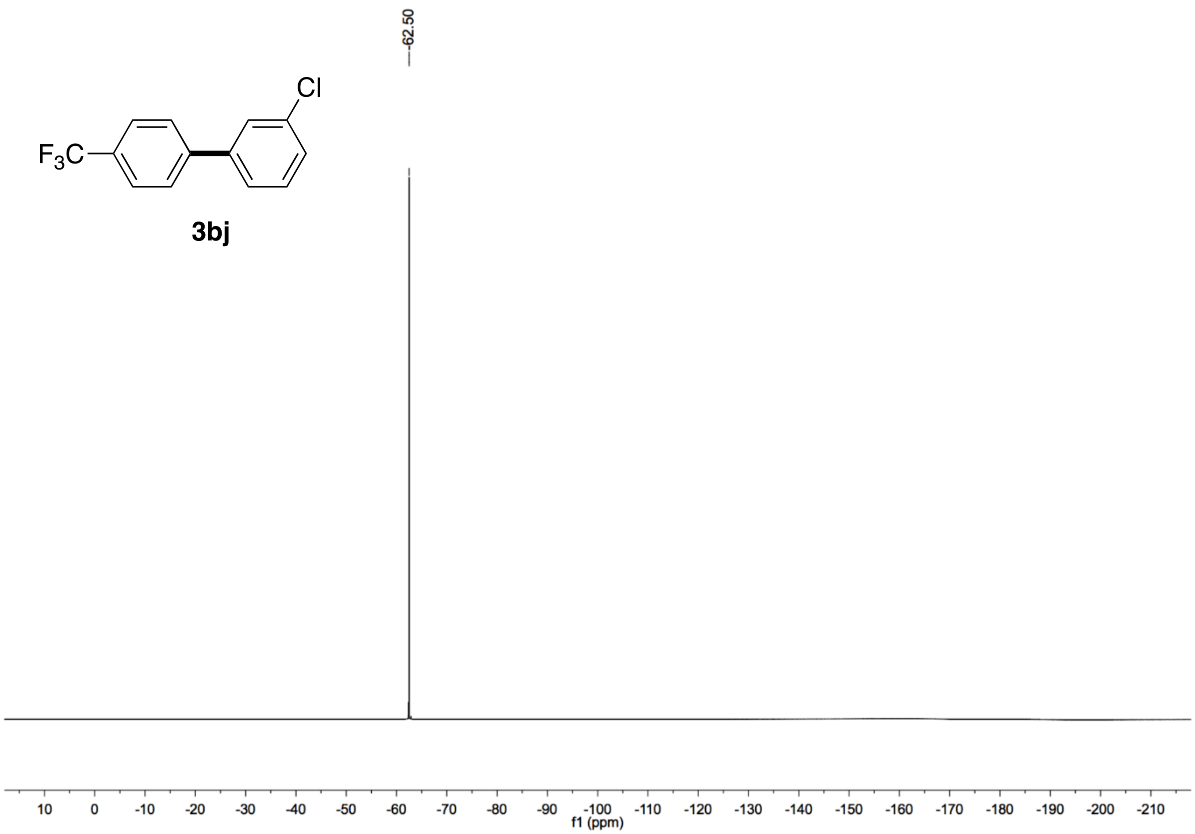

Figure S139. ^{1}H NMR spectrum of **3bk**, related to **Figure 4**

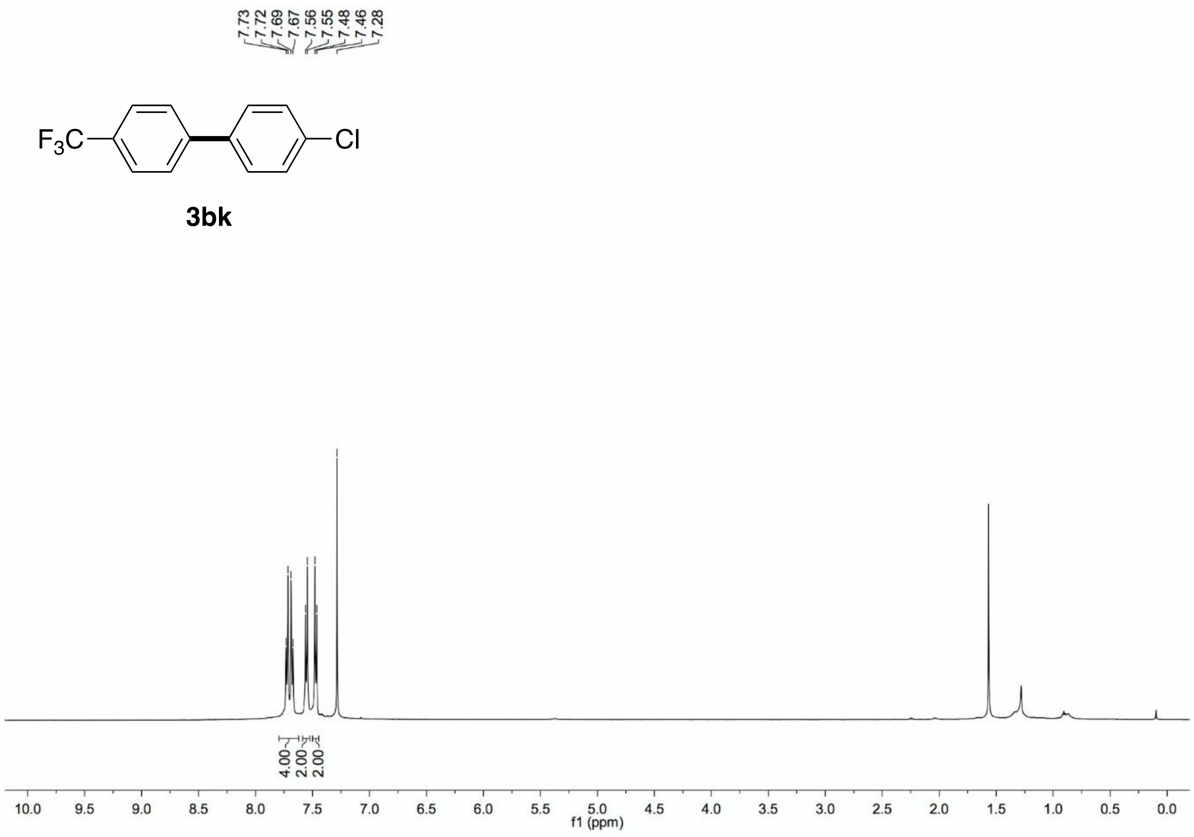

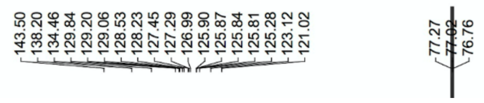

Figure S140. ^{13}C NMR spectrum of **3bk**, related to **Figure 4**

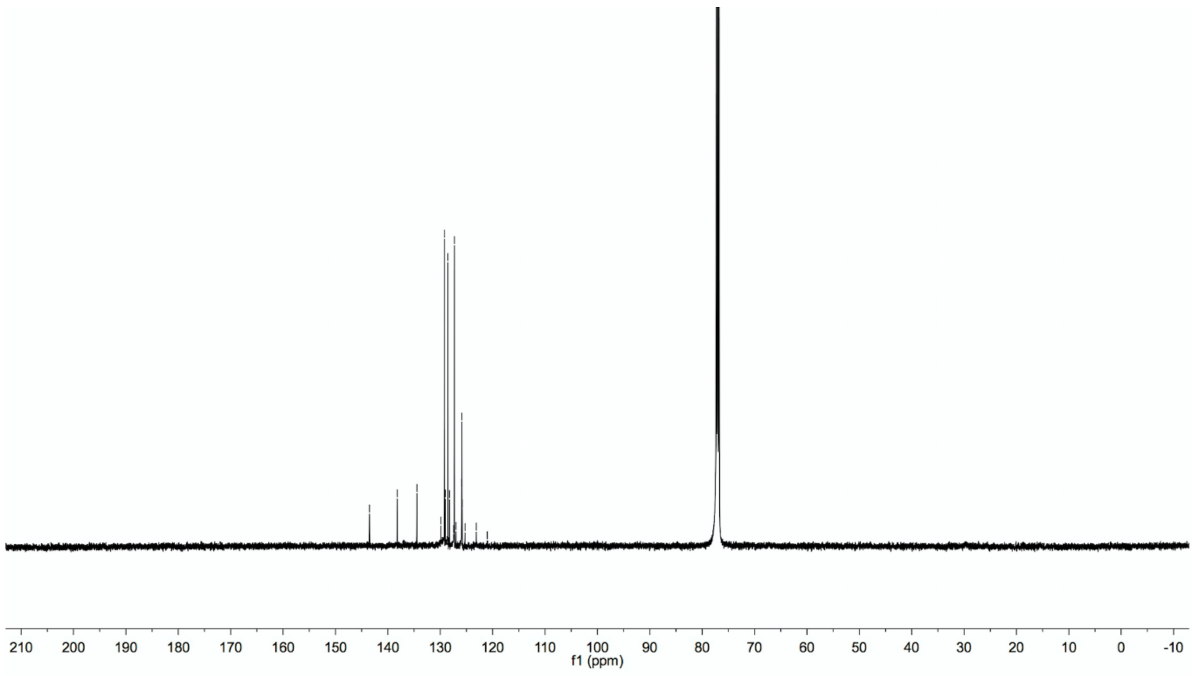

Figure S141. ^{1}H NMR spectrum of **3bk**, related to **Figure 4**

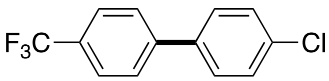

3bk

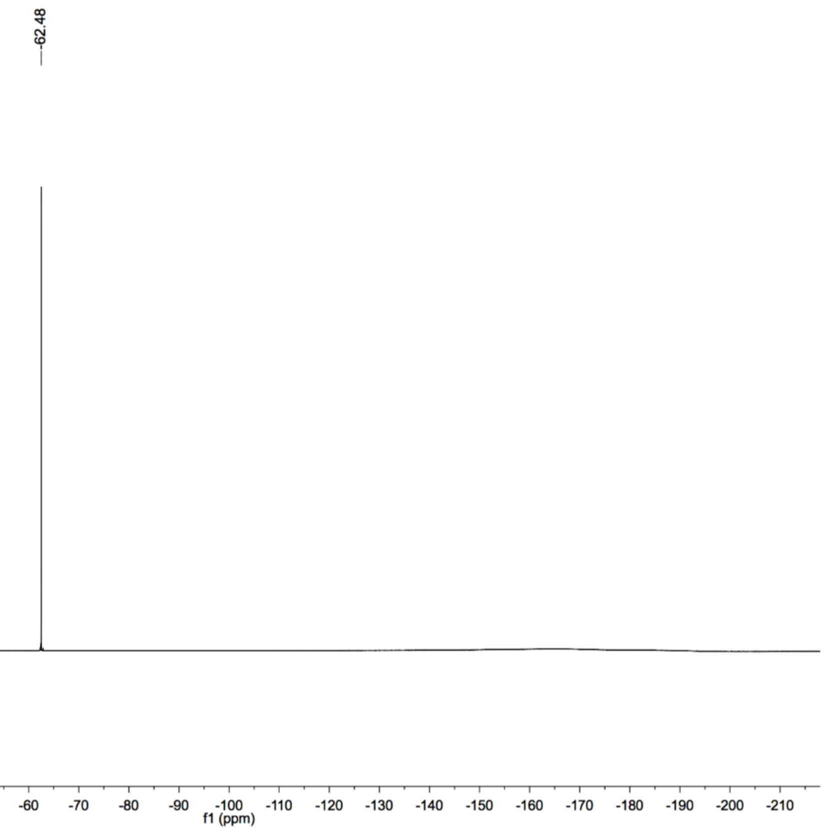

Figure S142. ^{1}H NMR spectrum of **3bl**, related to **Figure 4**

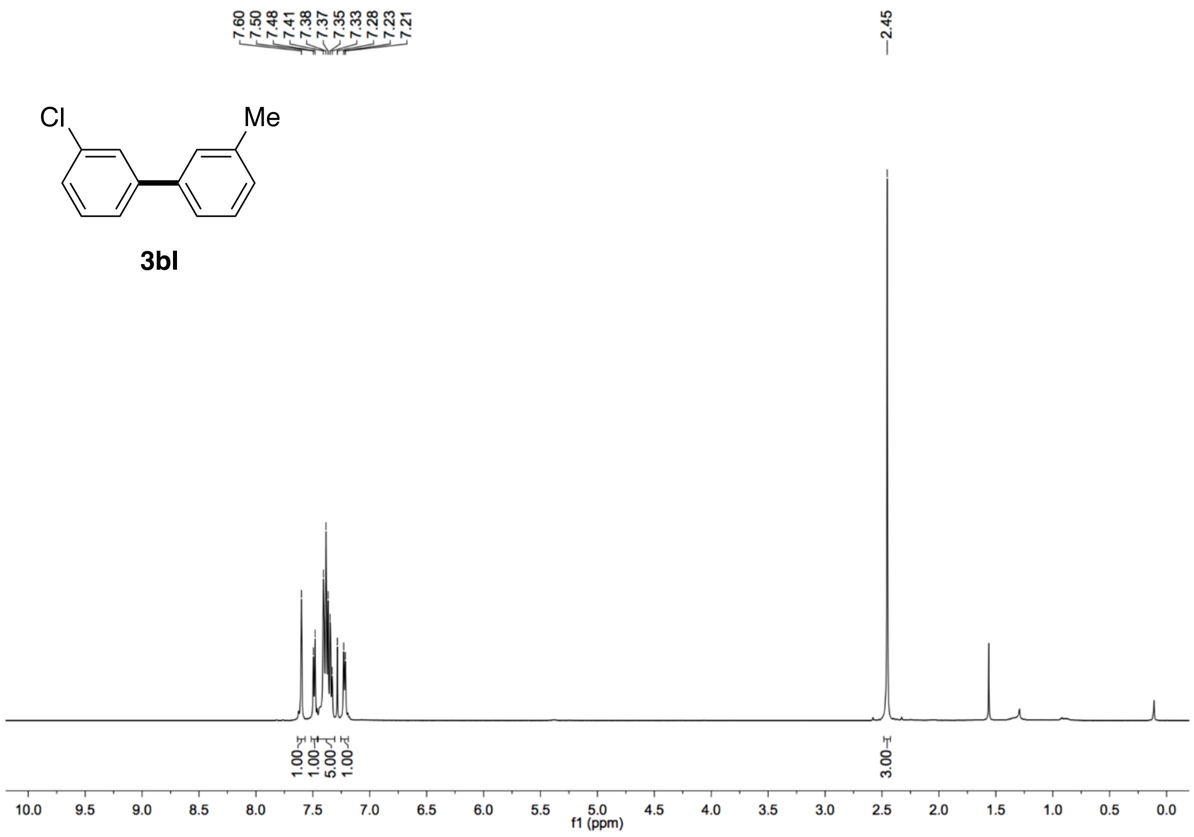

Figure S143. ^{13}C NMR spectrum of **3bl**, related to **Figure 4**

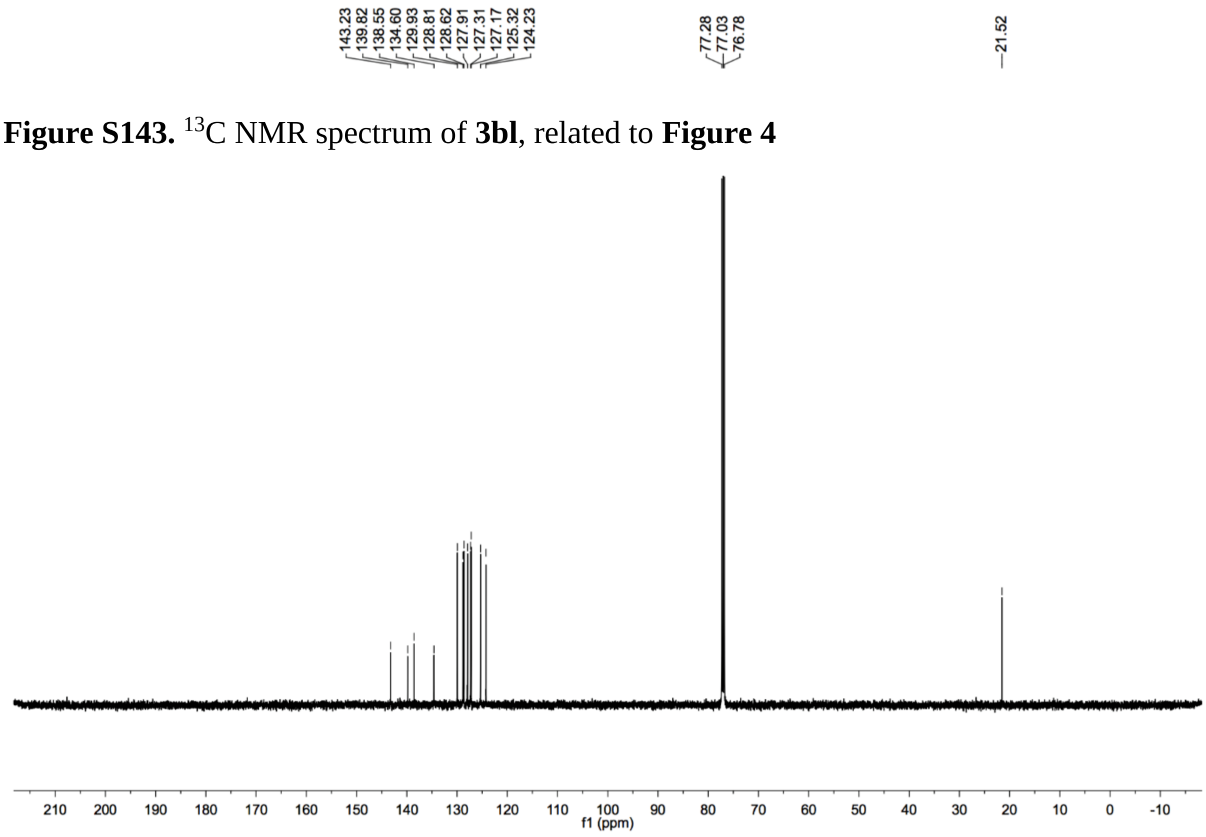

Figure S144. ^{1}H NMR spectrum of **3bm**, related to **Figure 4**

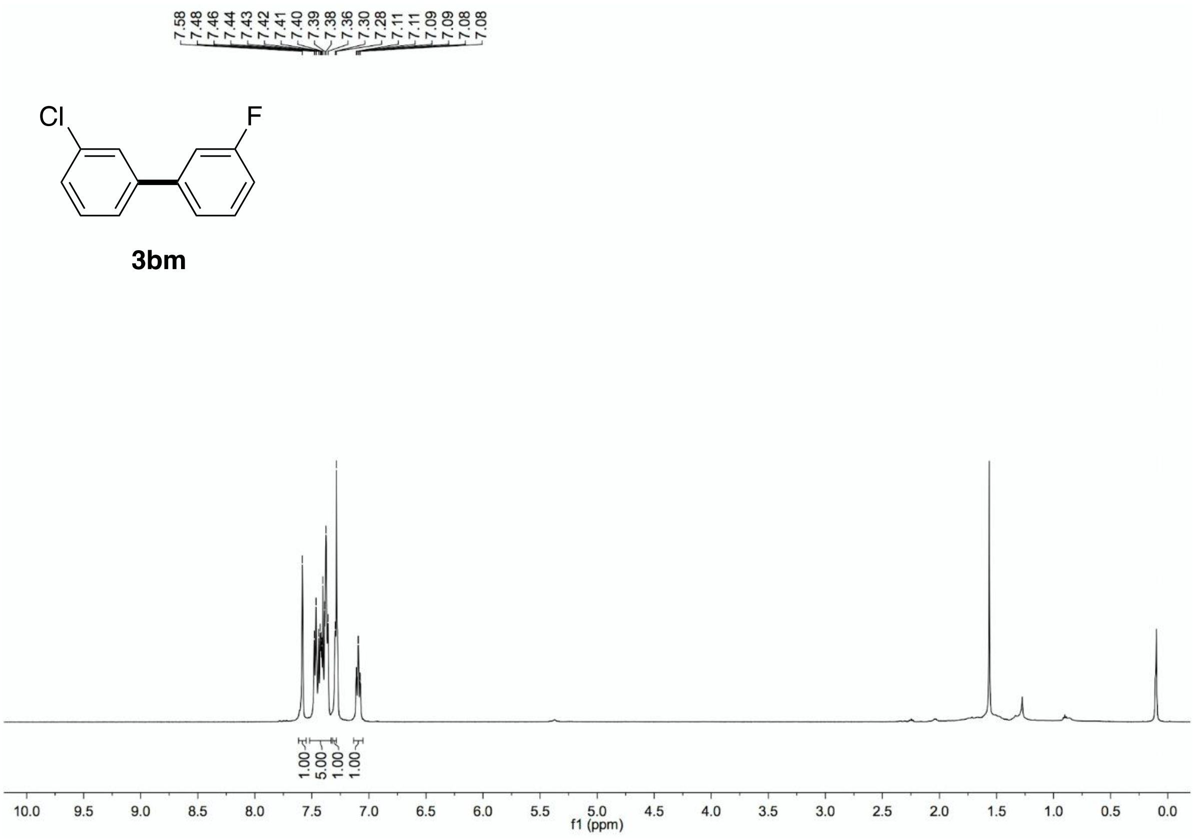

Figure S145. ^{13}C NMR spectrum of **3bm**, related to **Figure 4**

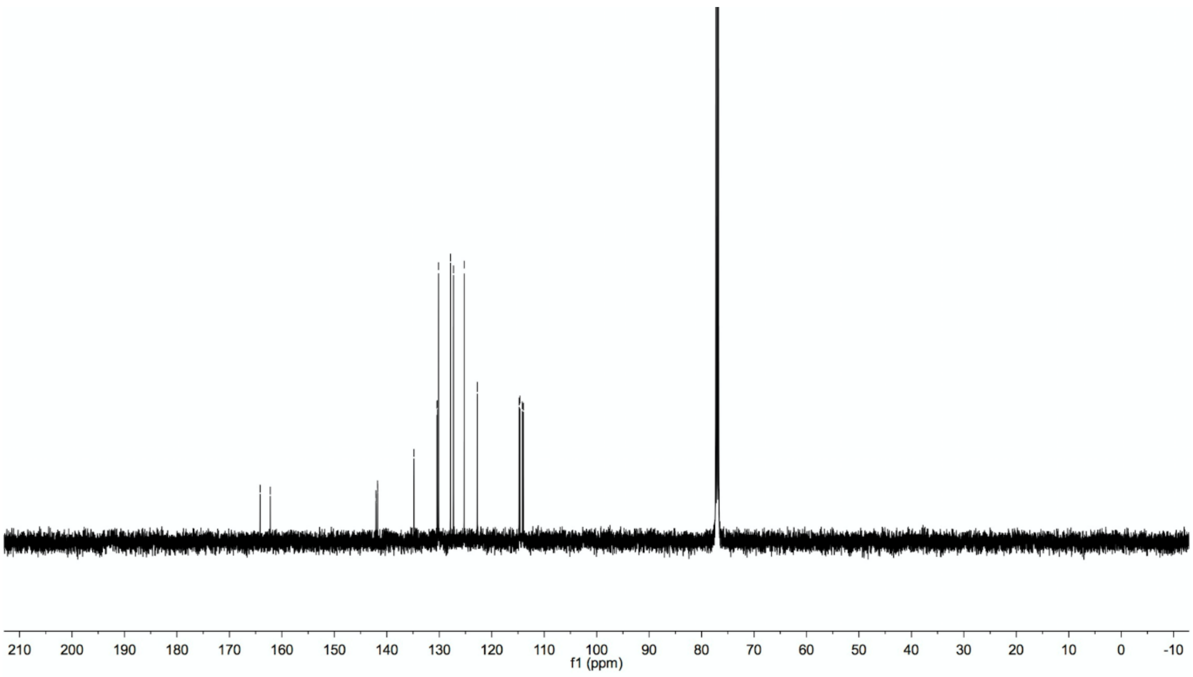

Figure S146. ^{19}F NMR spectrum of **3bm**, related to **Figure 4**

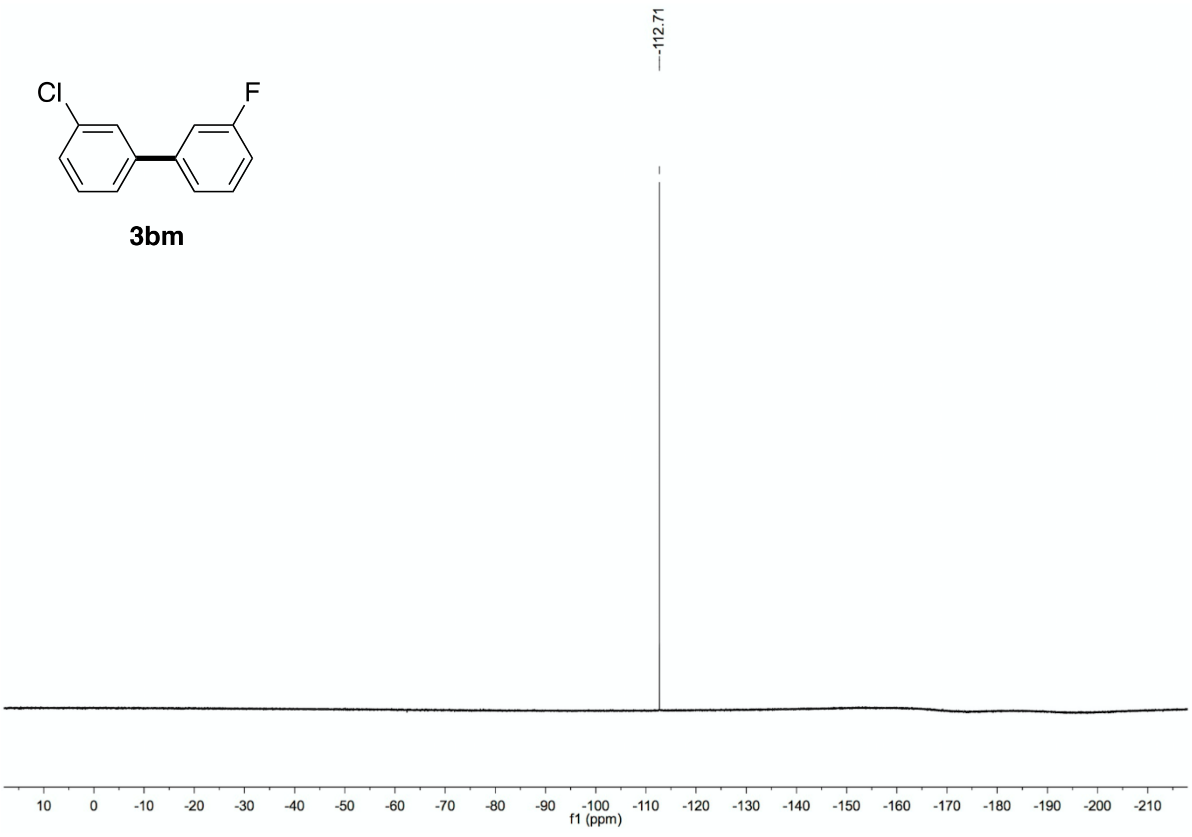

Figure S147. ^{1}H NMR spectrum of **3bn**, related to **Figure 4**

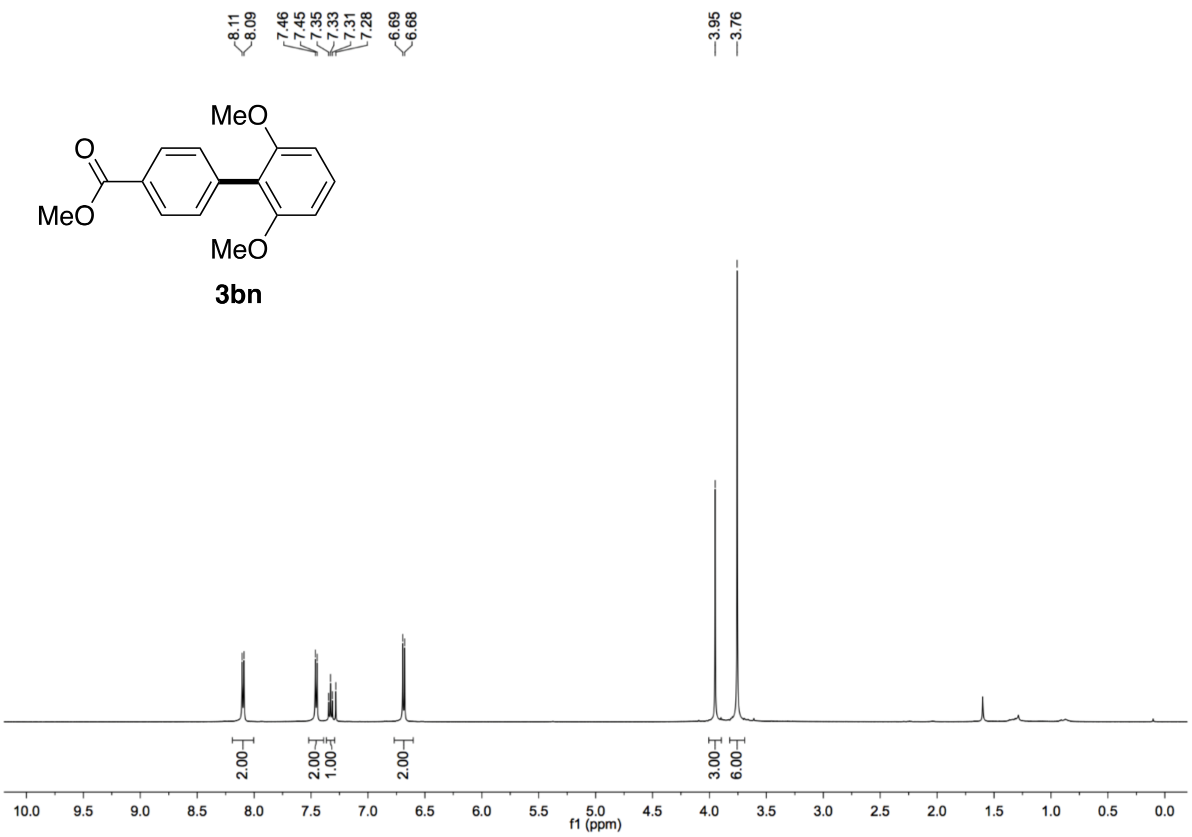

Figure S148. ^{13}C NMR spectrum of **3bn**, related to **Figure 4**

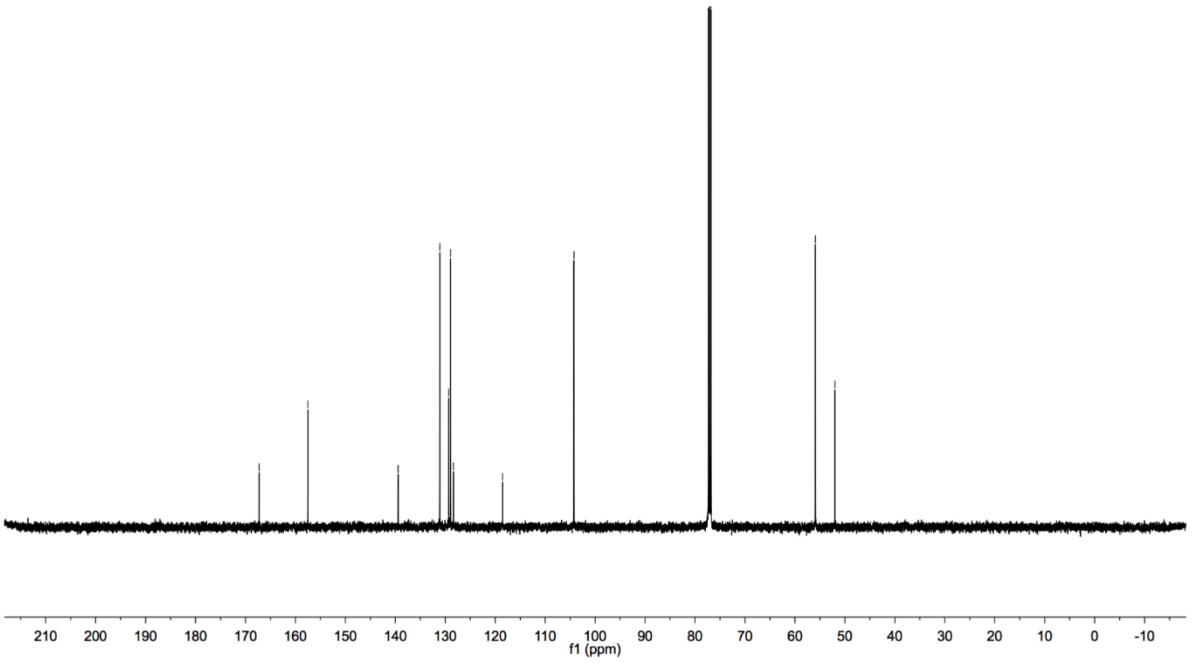

Figure S149. ^{1}H NMR spectrum of **3bo**, related to **Figure 4**

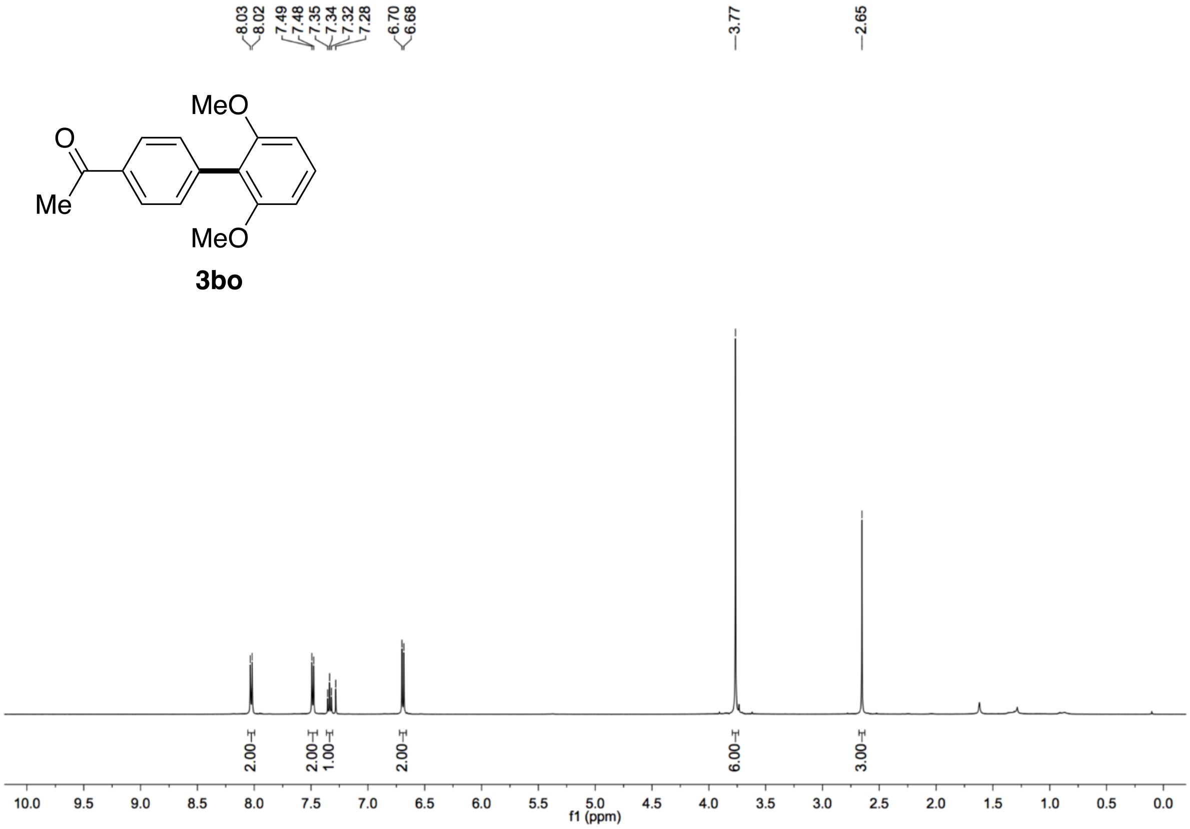

Figure S150. ^{13}C NMR spectrum of **3bo**, related to **Figure 4**

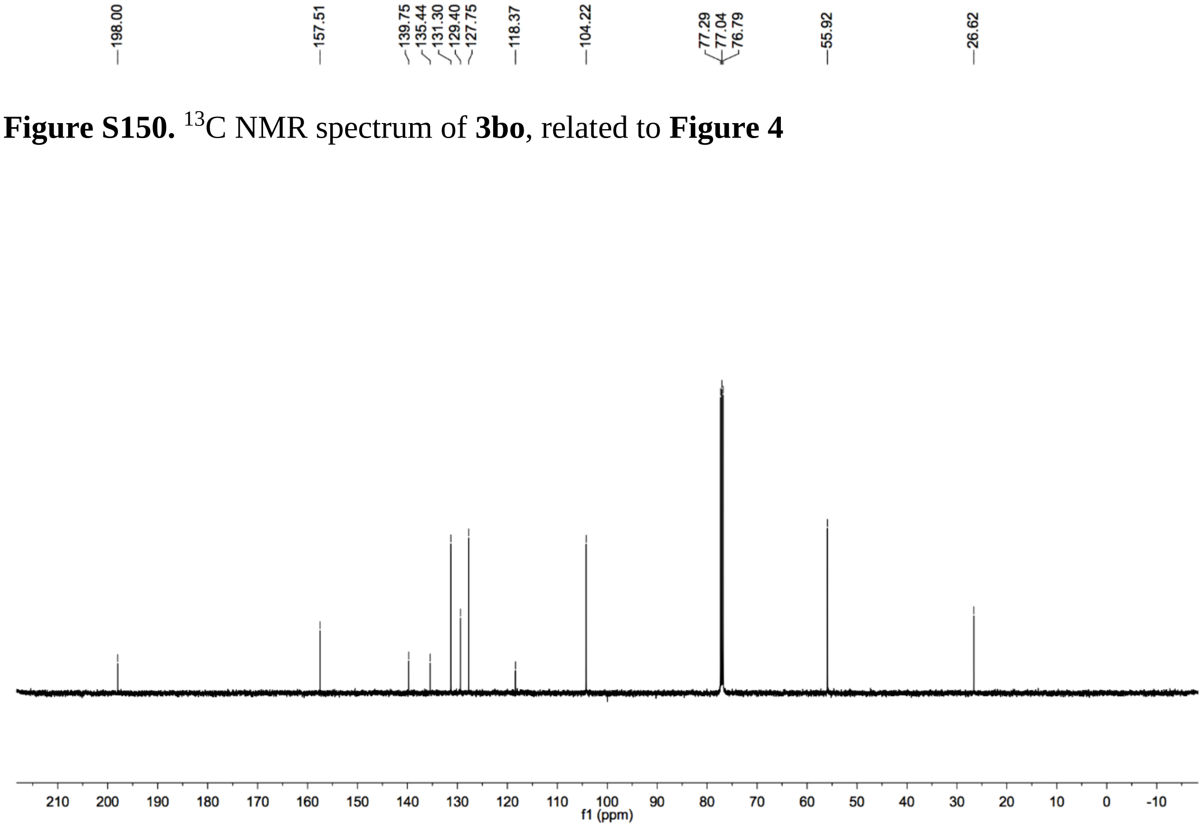

Figure S151. ^{1}H NMR spectrum of **3bp**, related to **Figure 4**

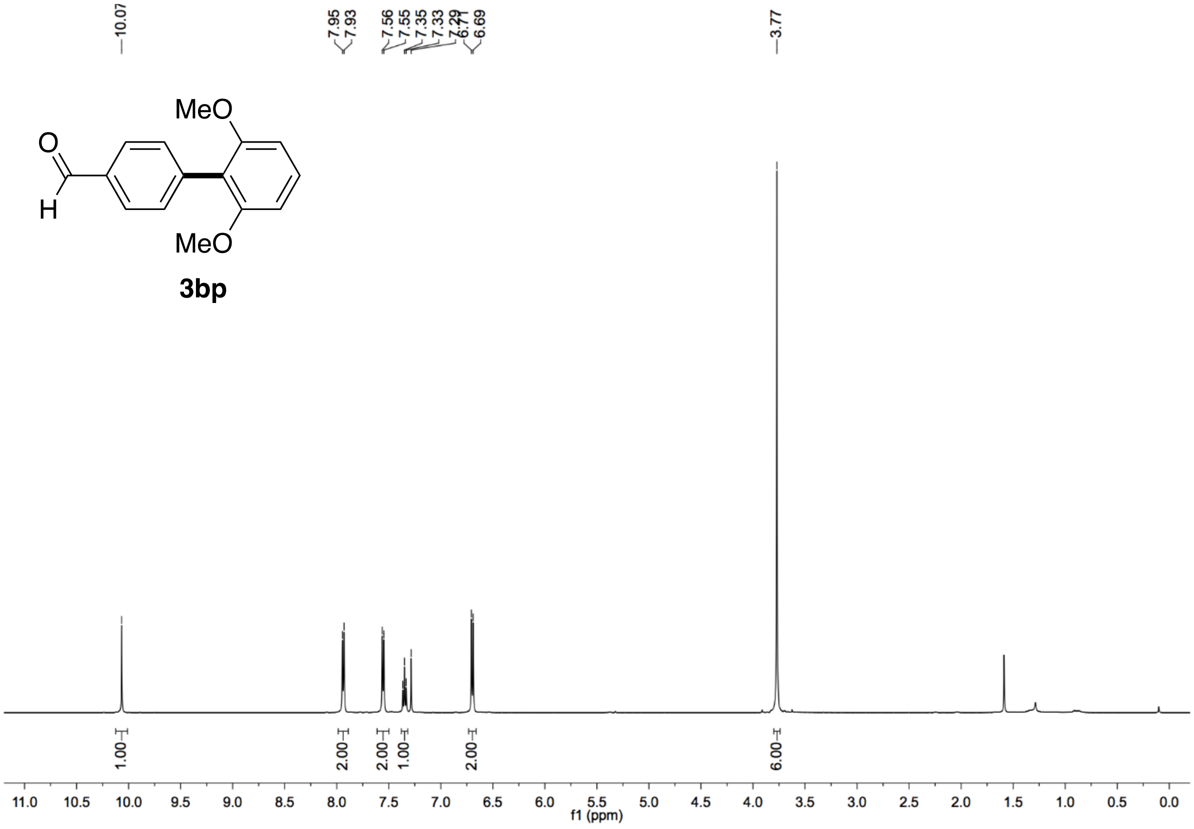

Figure S152. ^{13}C NMR spectrum of **3bp**, related to **Figure 4**

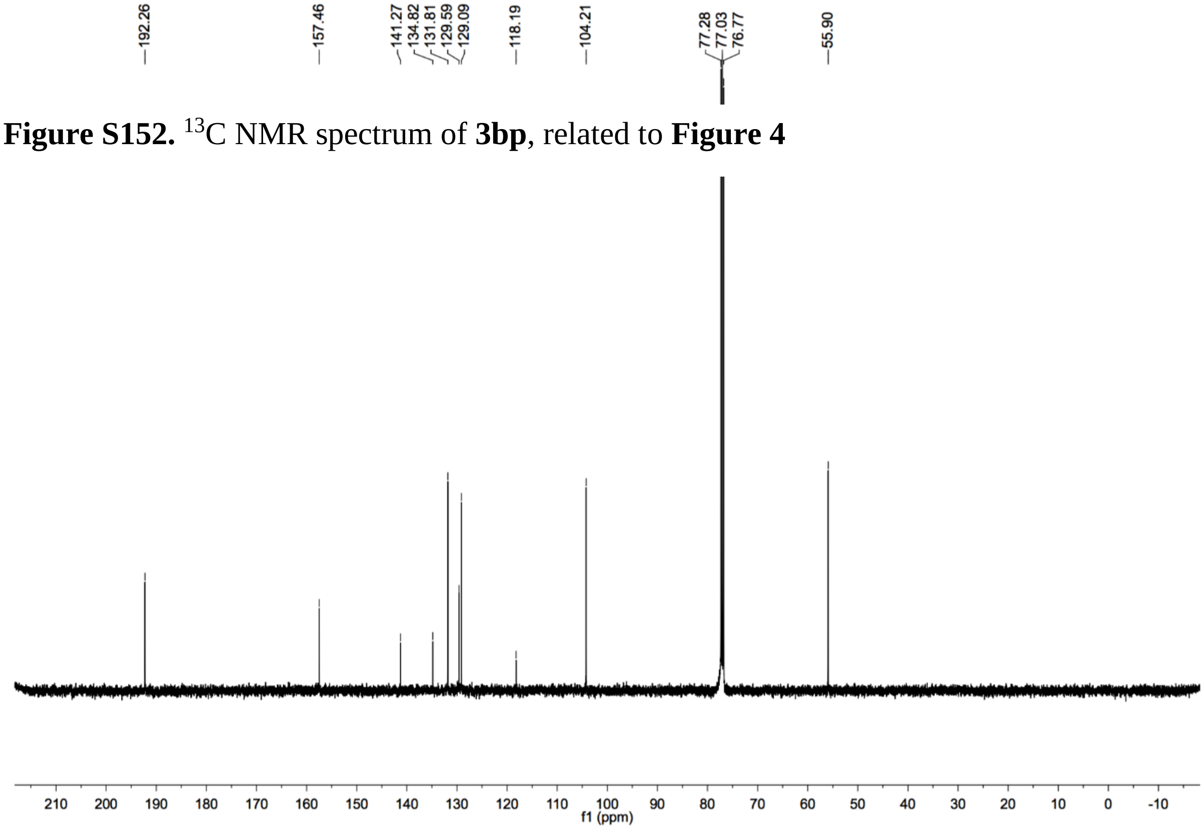

Figure S153. ^{1}H NMR spectrum of **3bq**, related to **Figure 4**

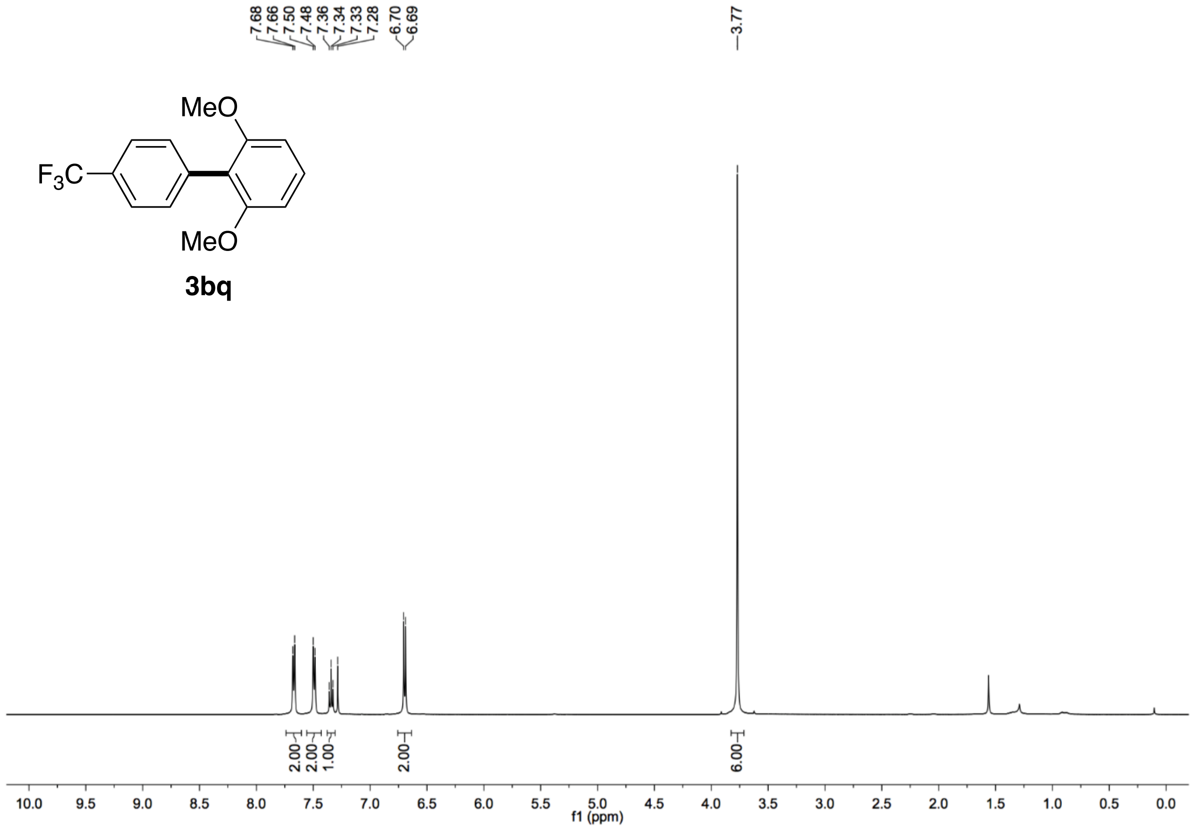

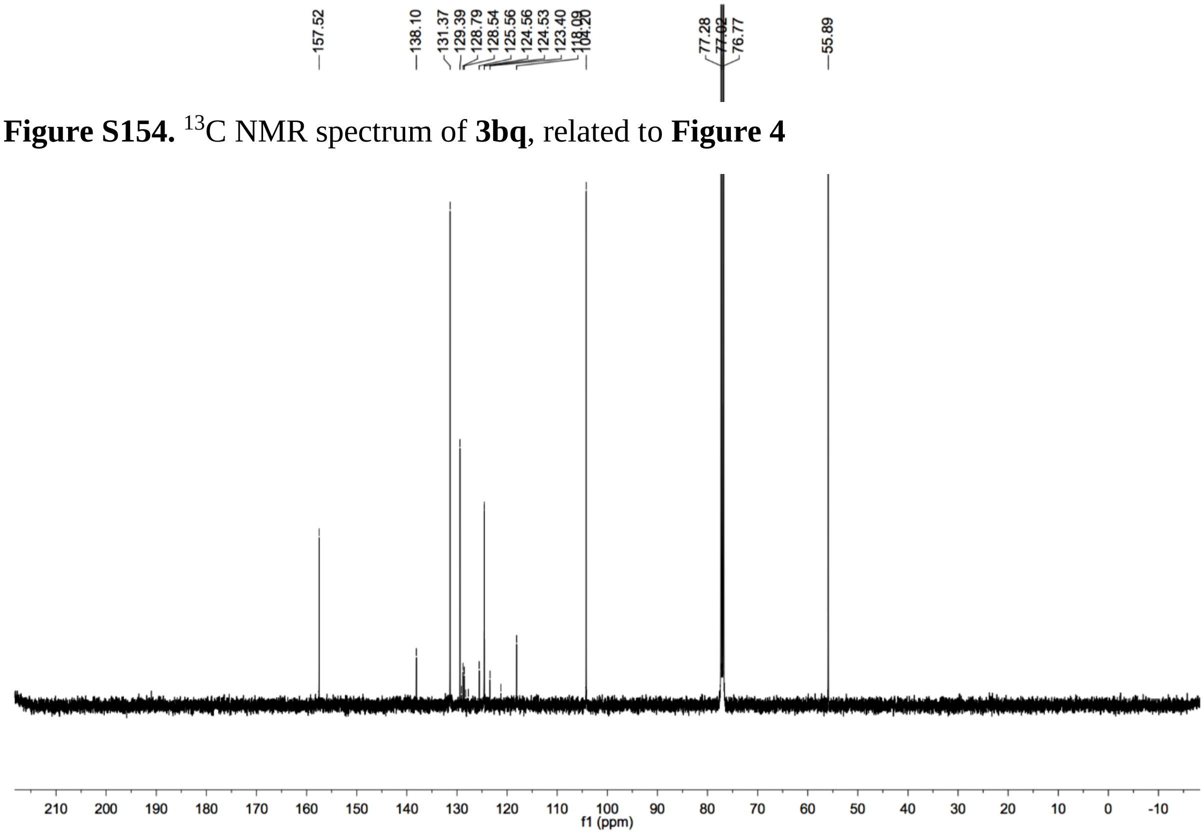

Figure S154. ^{13}C NMR spectrum of **3bq**, related to **Figure 4**

Figure S155. ^{19}F NMR spectrum of **3bq**, related to **Figure 4**

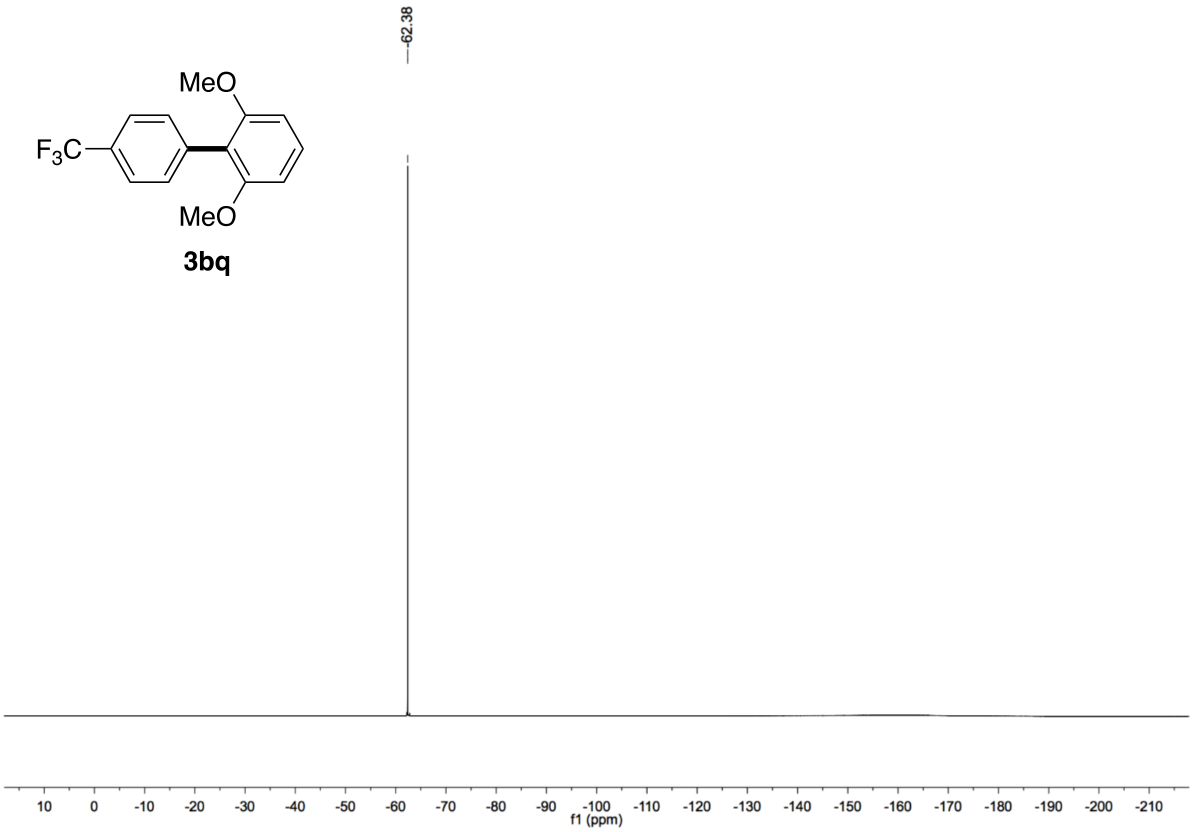

Figure S156. ^{1}H NMR spectrum of **3br**, related to **Figure 4**

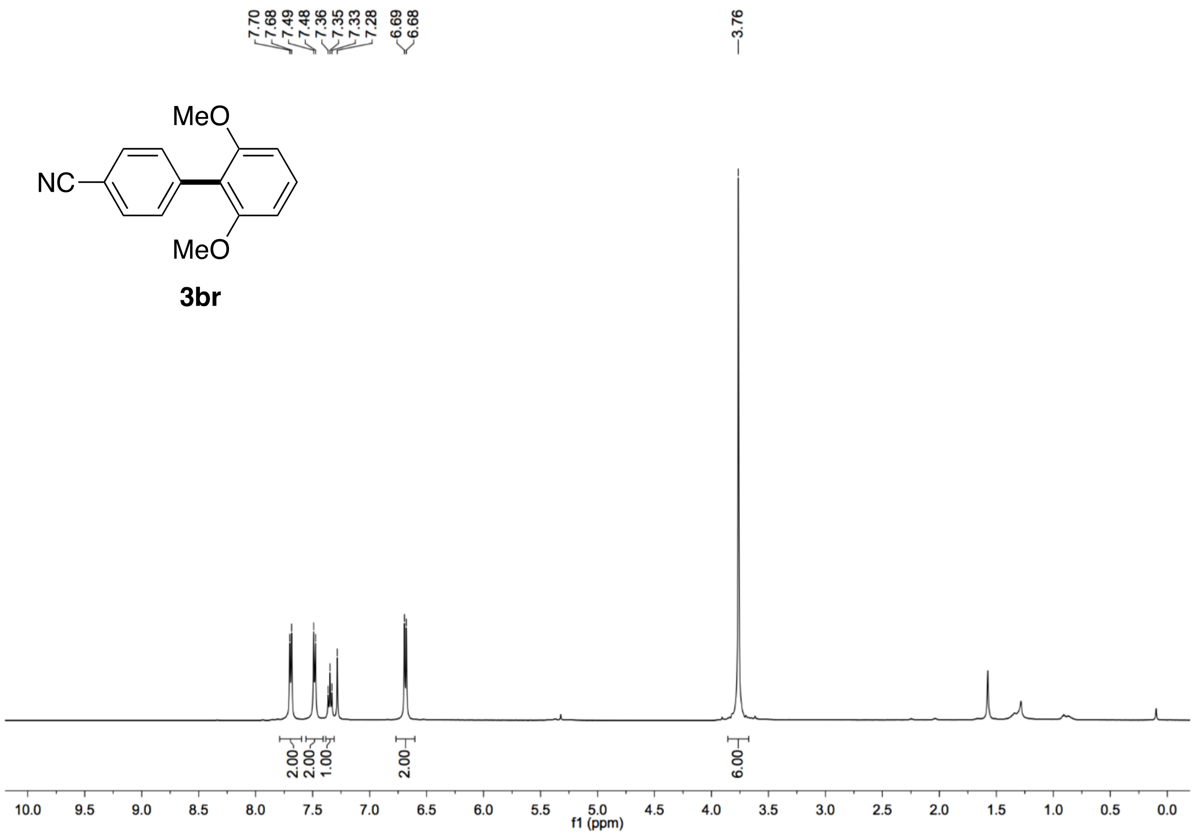

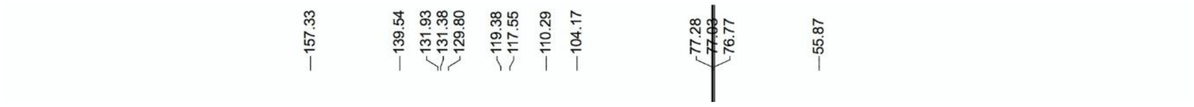

Figure S157. ^{13}C NMR spectrum of **3br**, related to **Figure 4**

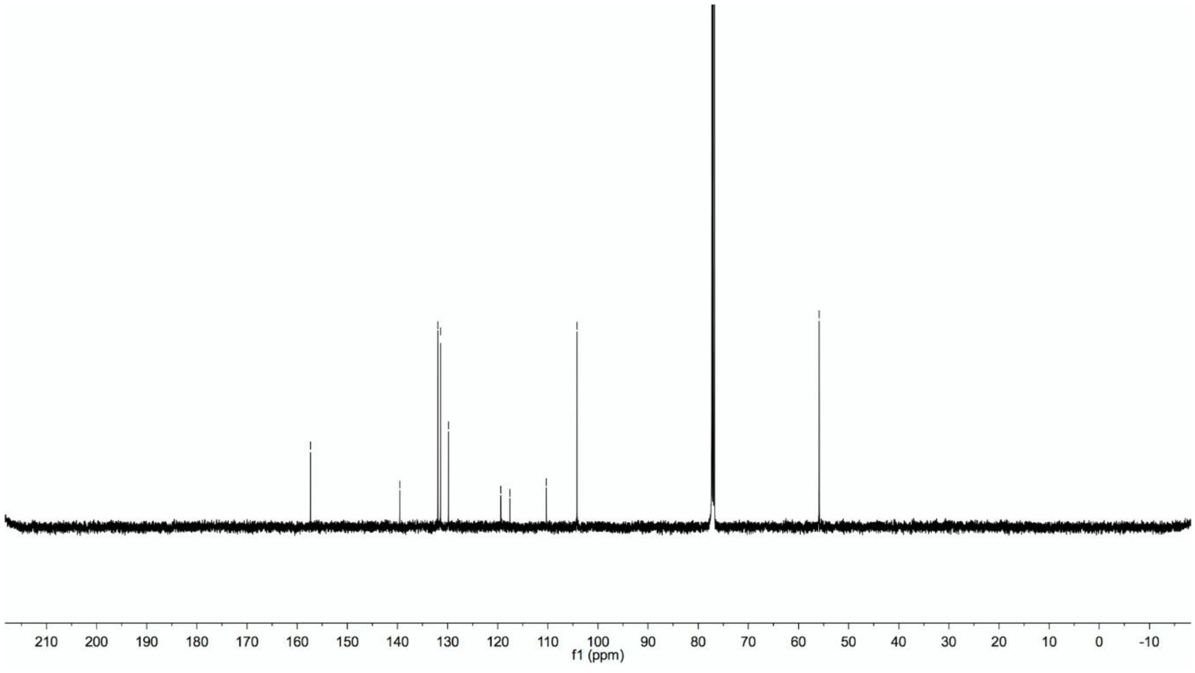

Figure S158. ^{1}H NMR spectrum of **3bs**, related to **Figure 4**

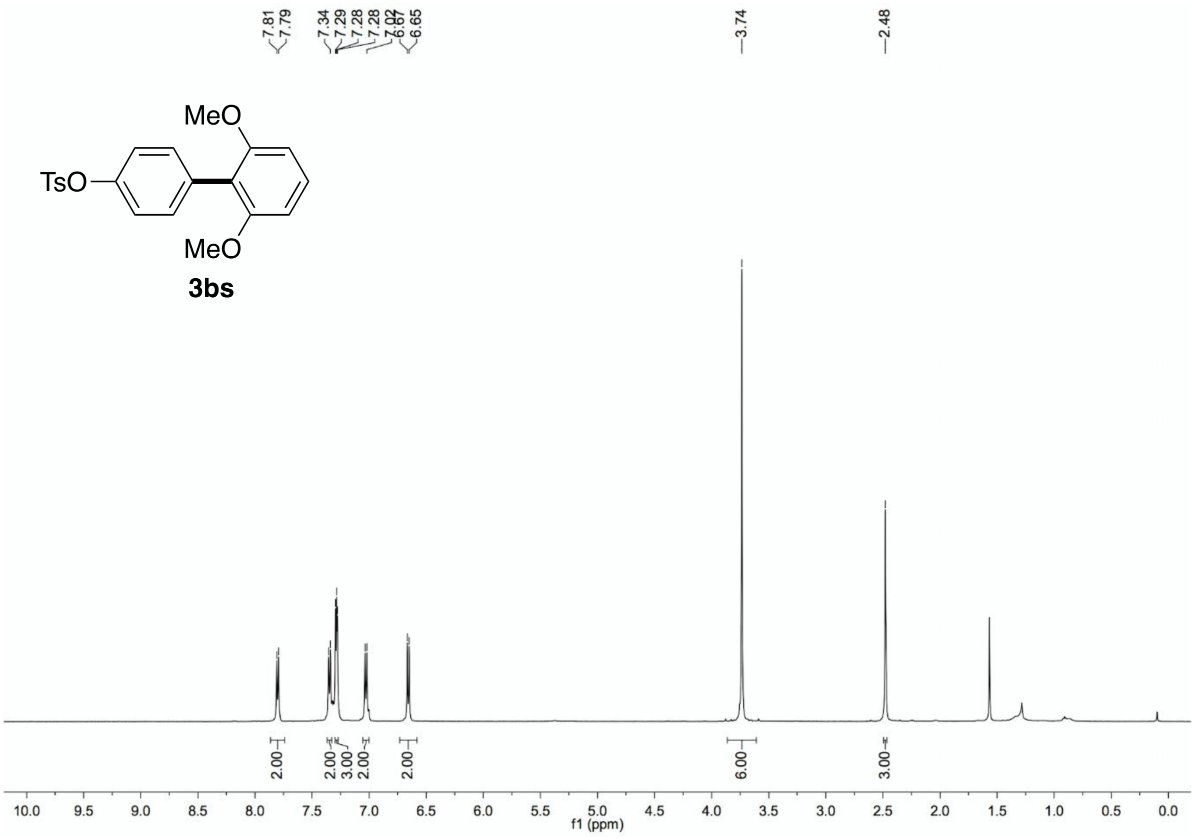

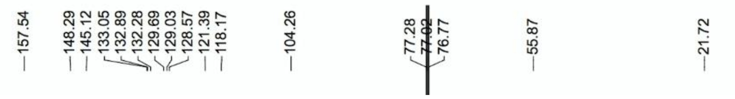

Figure S159. ^{13}C NMR spectrum of **3bs**, related to **Figure 4**

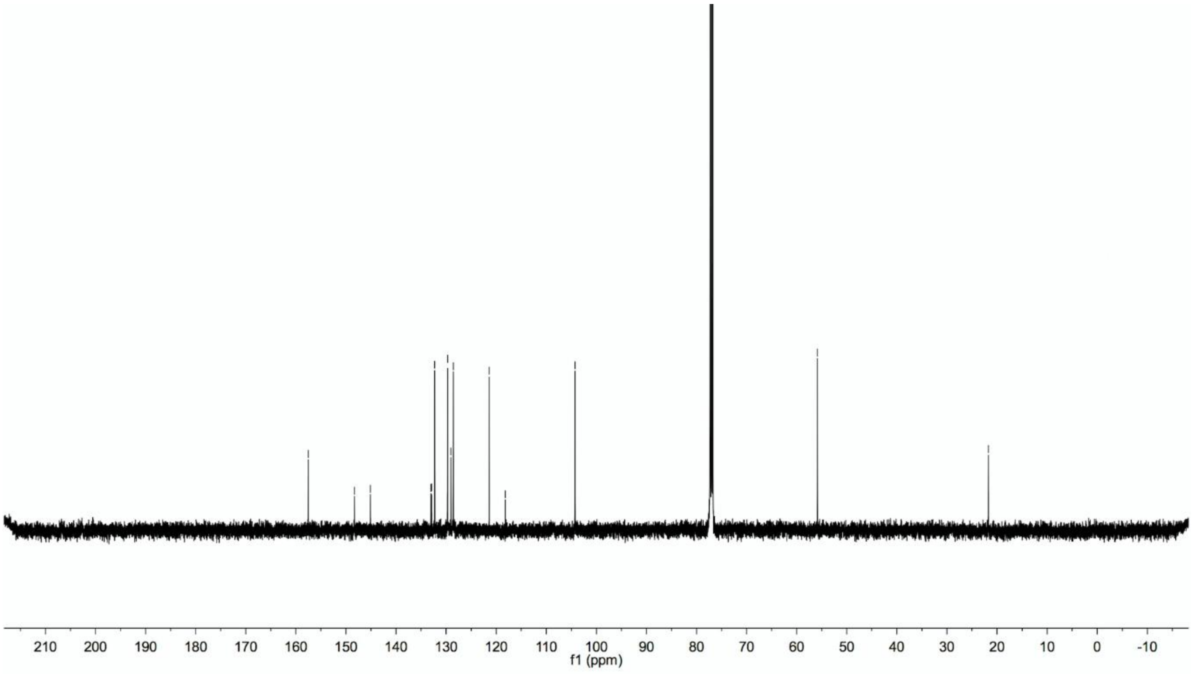

Figure S160. ^{1}H NMR spectrum of **3bt**, related to **Figure 4**

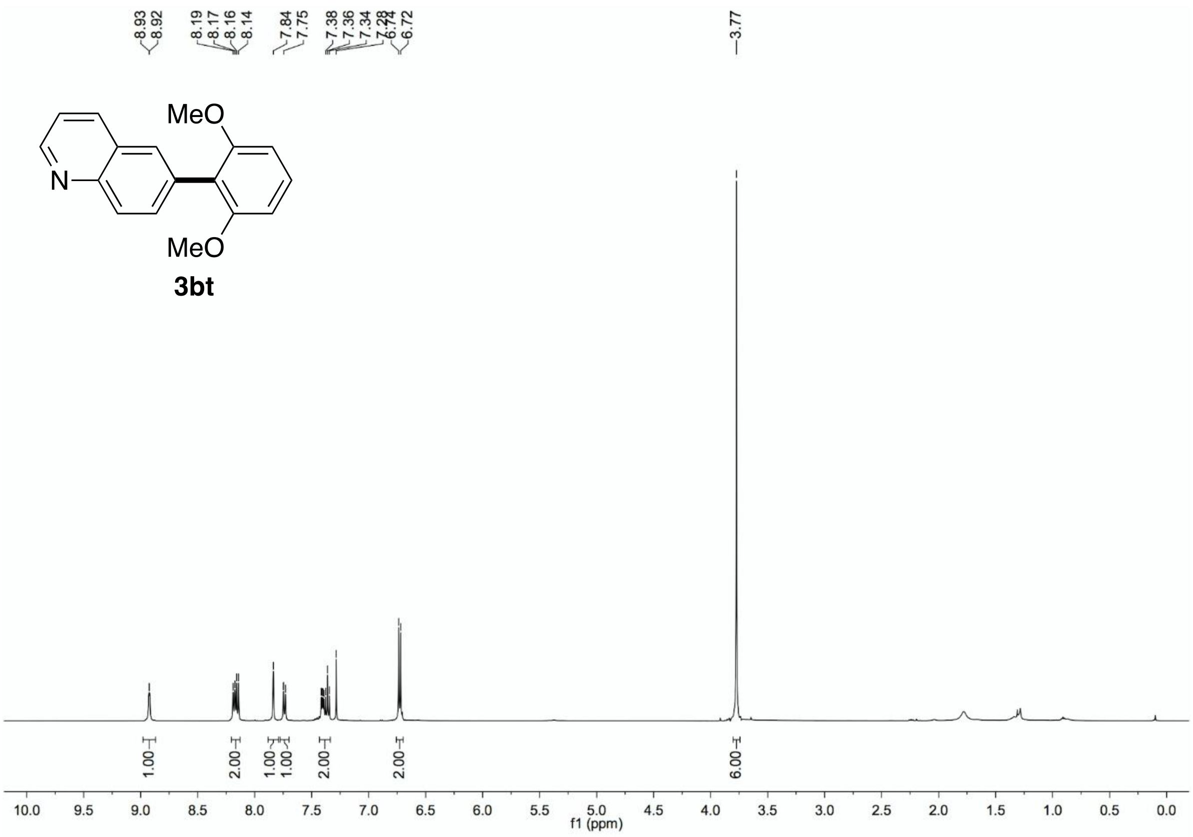

Figure S161. ^{13}C NMR spectrum of **3bt**, related to **Figure 4**

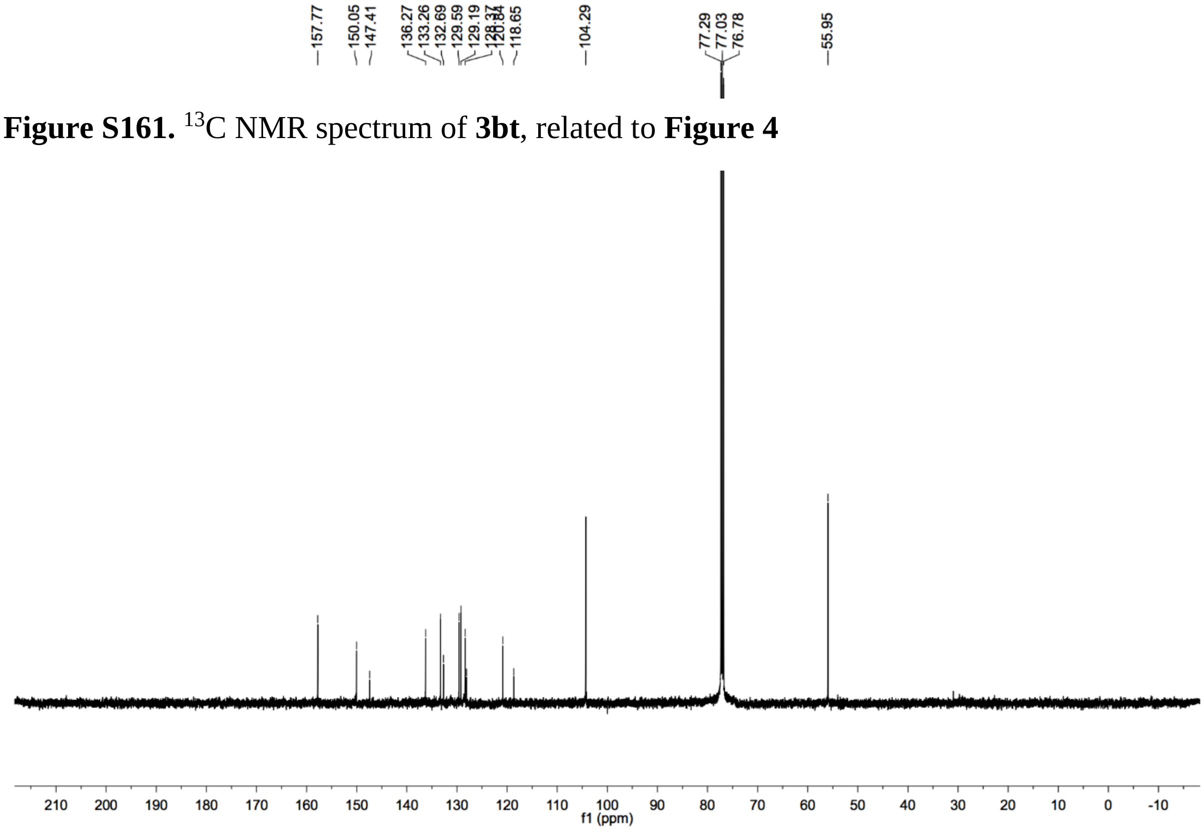

Figure S162. ^{1}H NMR spectrum of **3bu**, related to **Figure 4**

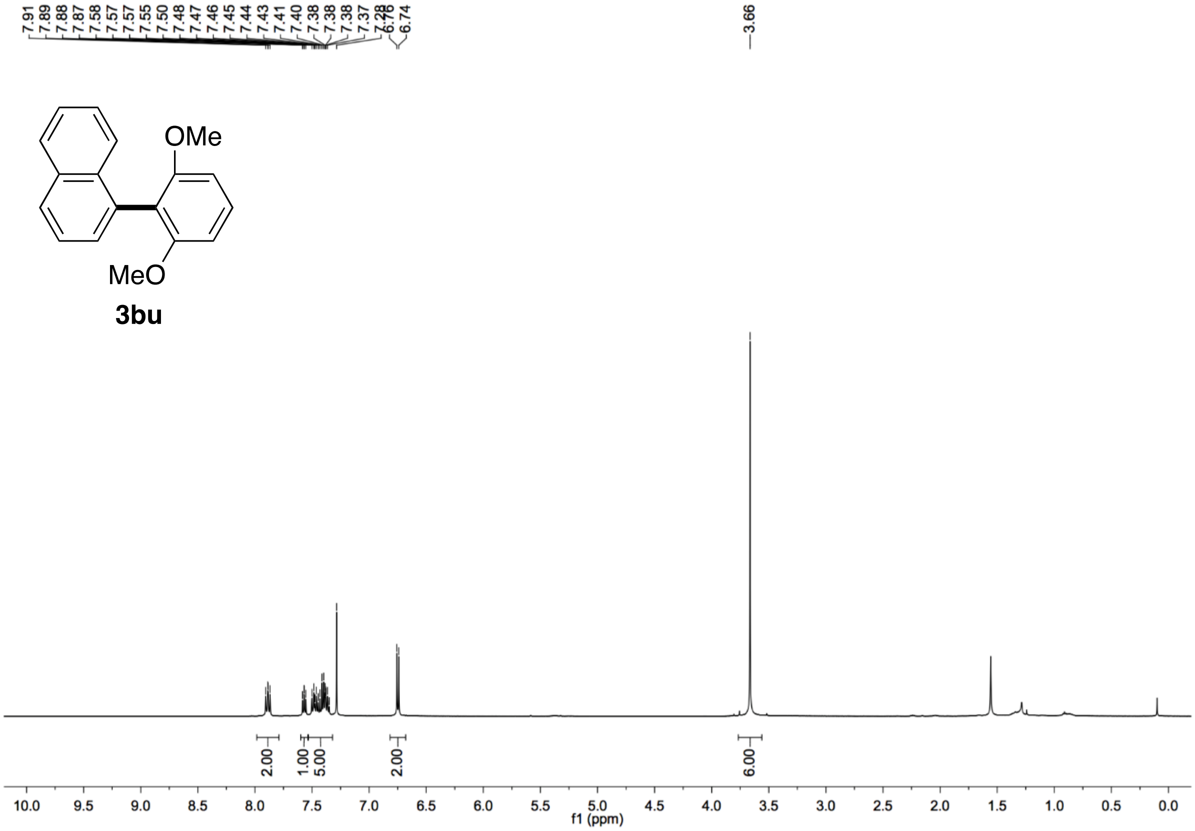

Figure S163. ^{13}C NMR spectrum of **3bu**, related to **Figure 4**

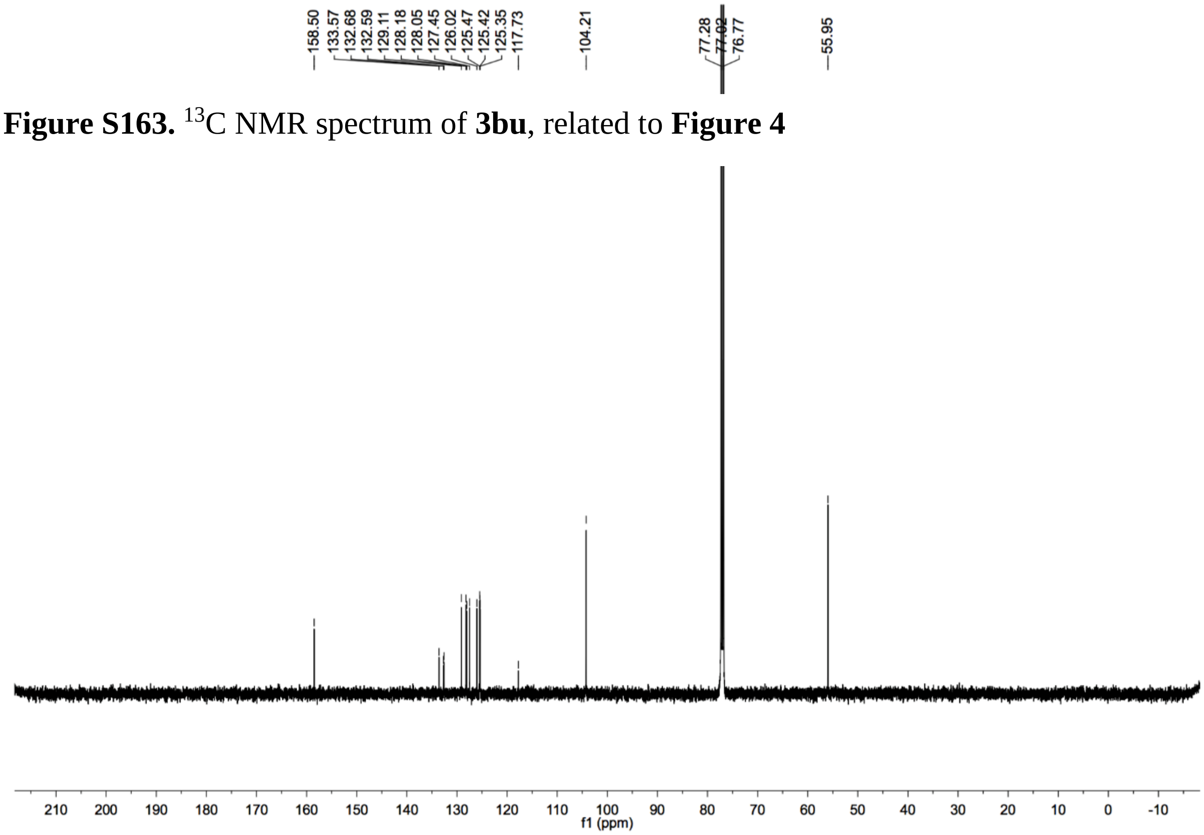

Figure S164. ^{1}H NMR spectrum of **3bv**, related to **Figure 4**

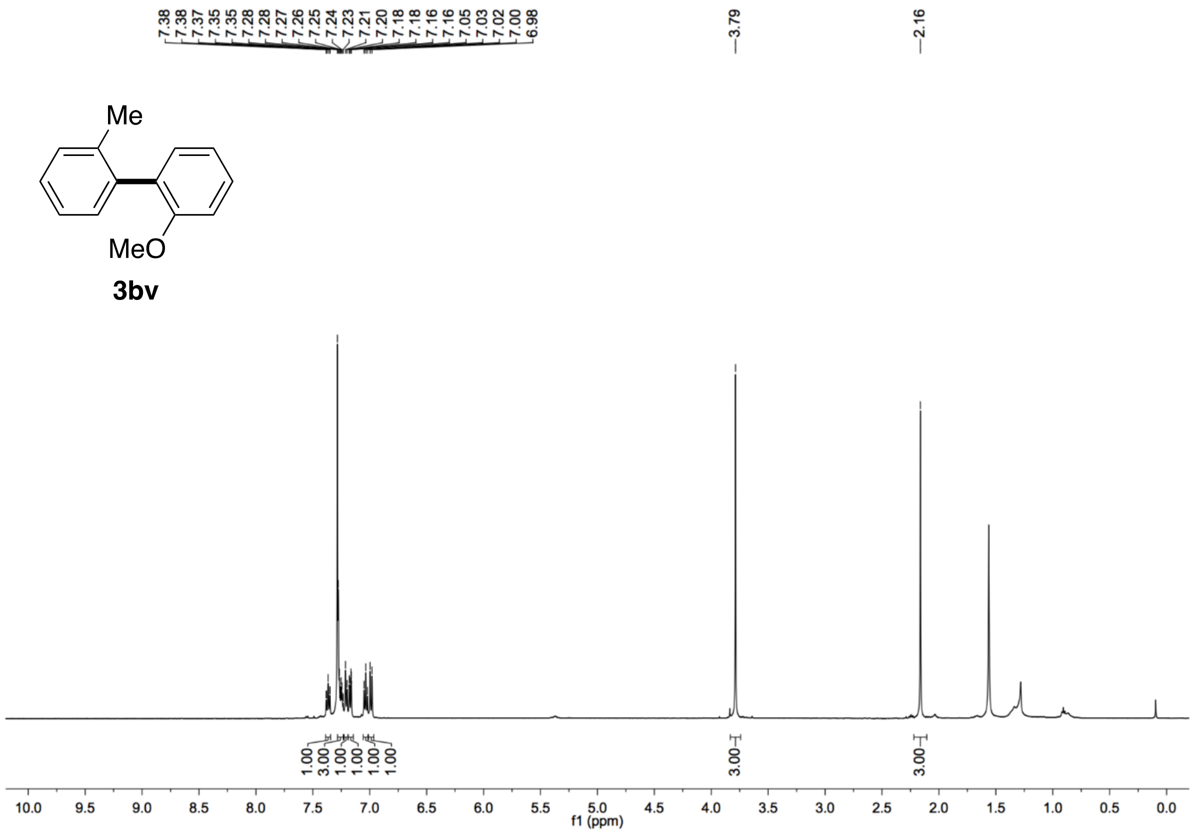

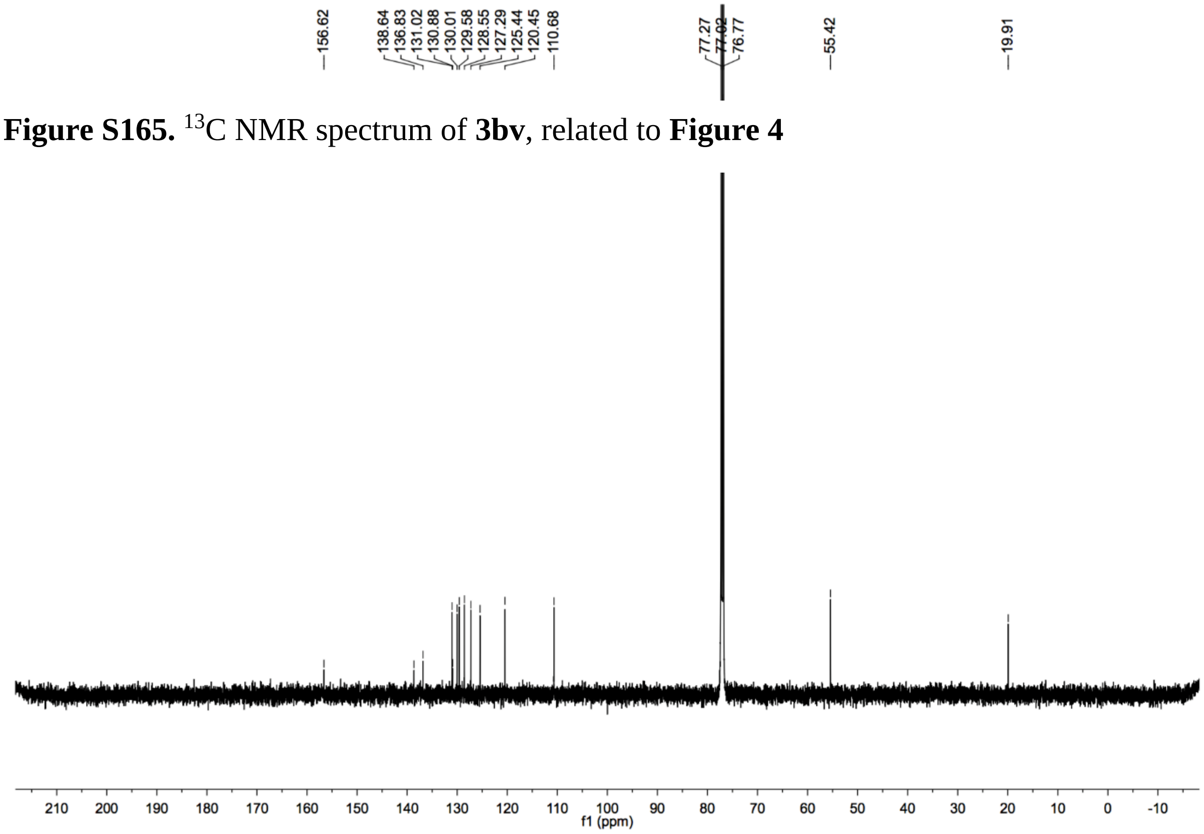

Figure S165. ^{13}C NMR spectrum of **3bv**, related to **Figure 4**

Figure S166. ^{1}H NMR spectrum of **3bw**, related to **Figure 4**

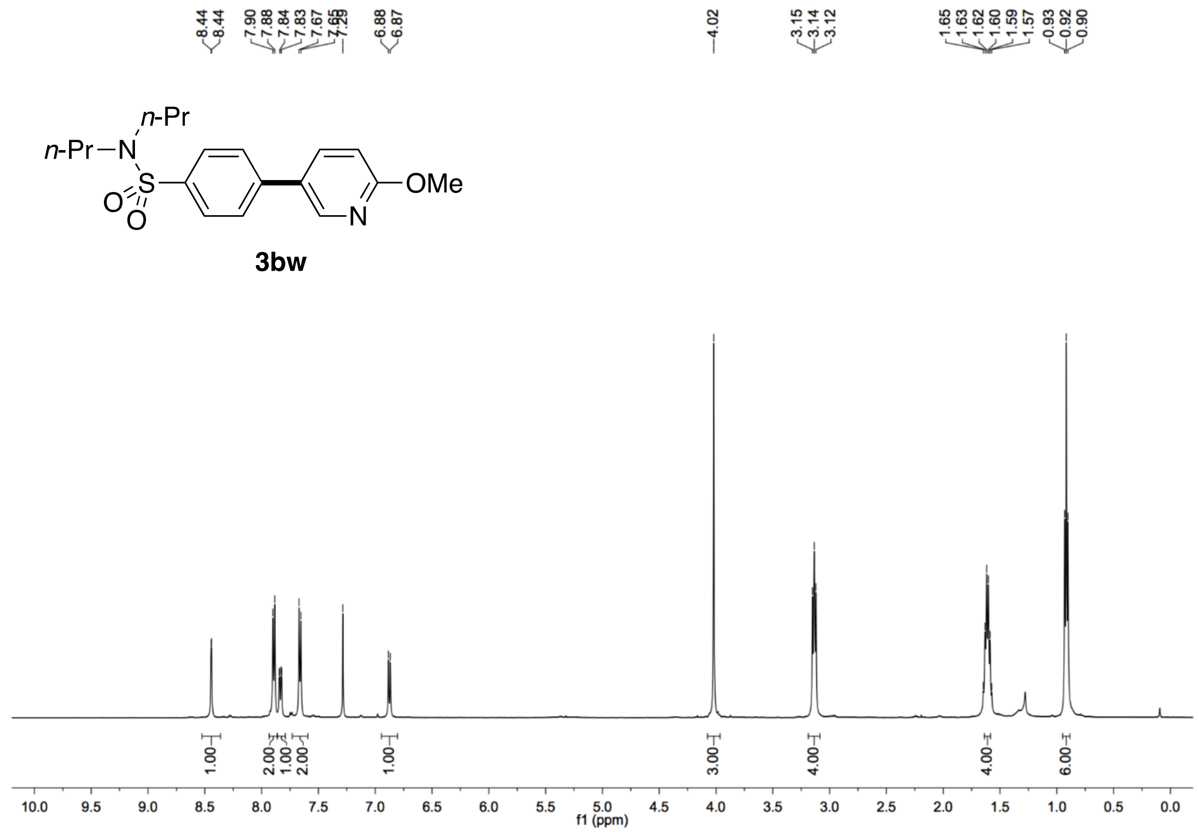

Figure S167. ^{13}C NMR spectrum of **3bw**, related to **Figure 4**

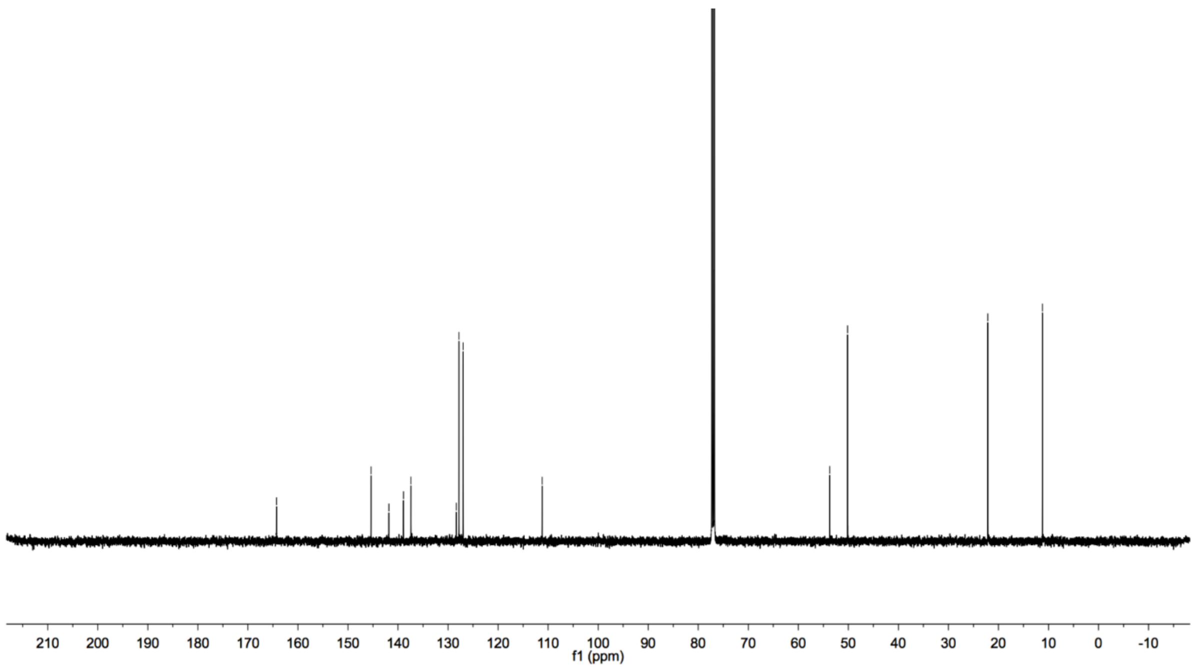

Figure S168. ^{1}H NMR spectrum of **3bx**, related to **Figure 4**

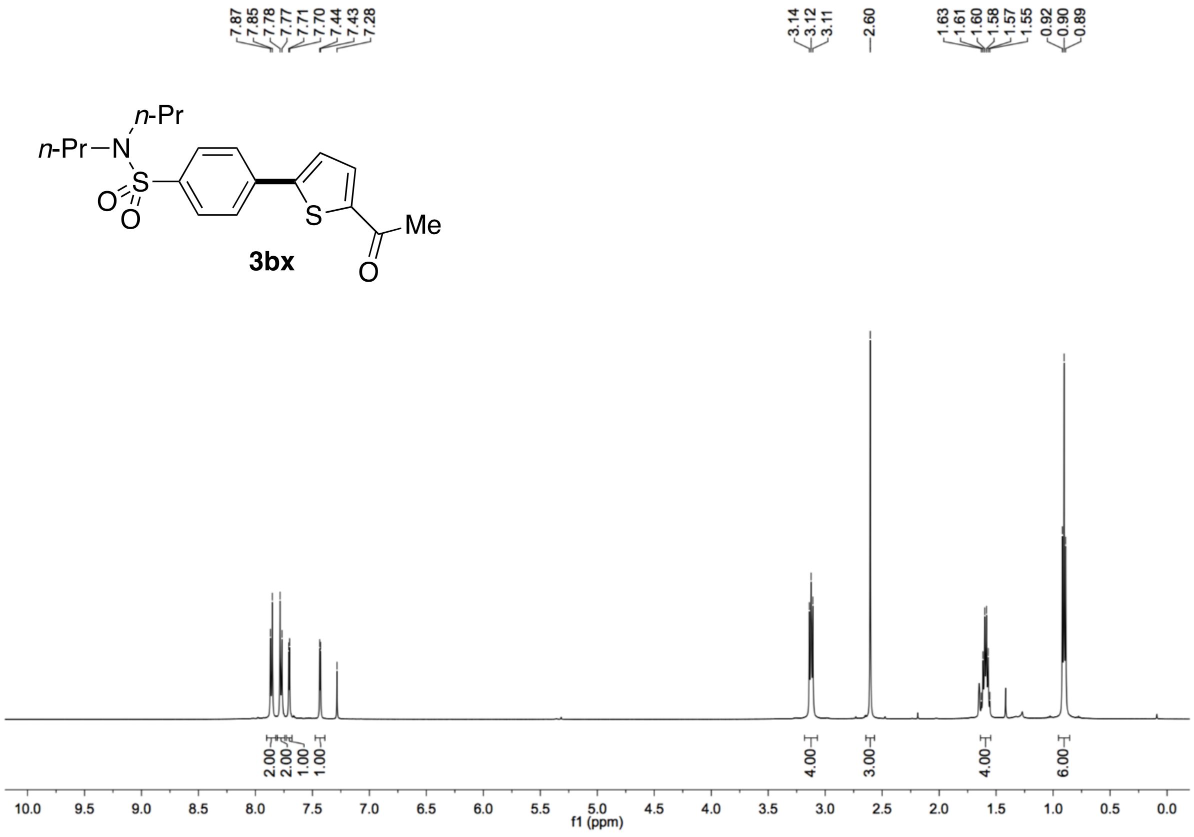

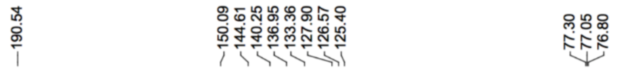

Figure S169. ^{13}C NMR spectrum of **3bx**, related to **Figure 4**

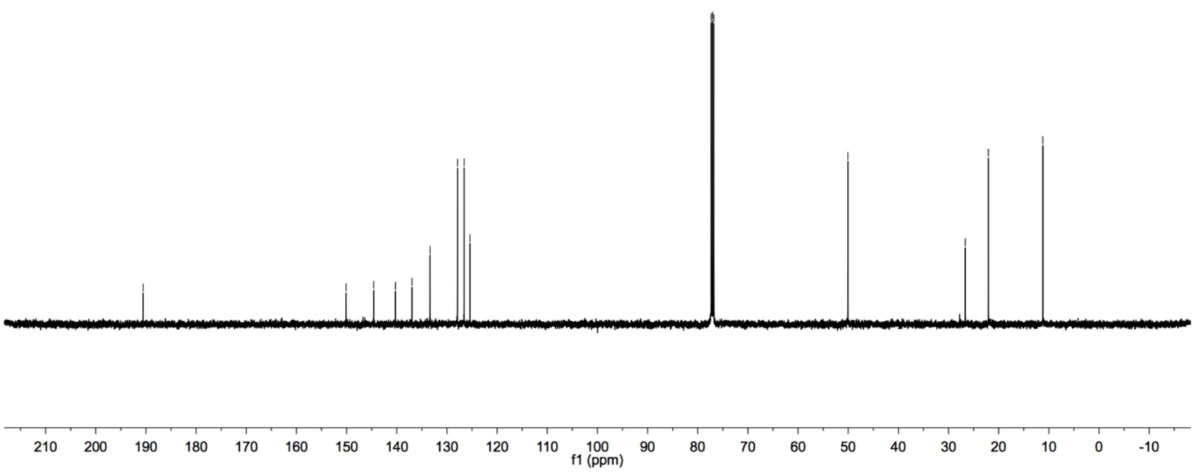

Figure S170. ^{1}H NMR spectrum of **3by**, related to **Figure 4**

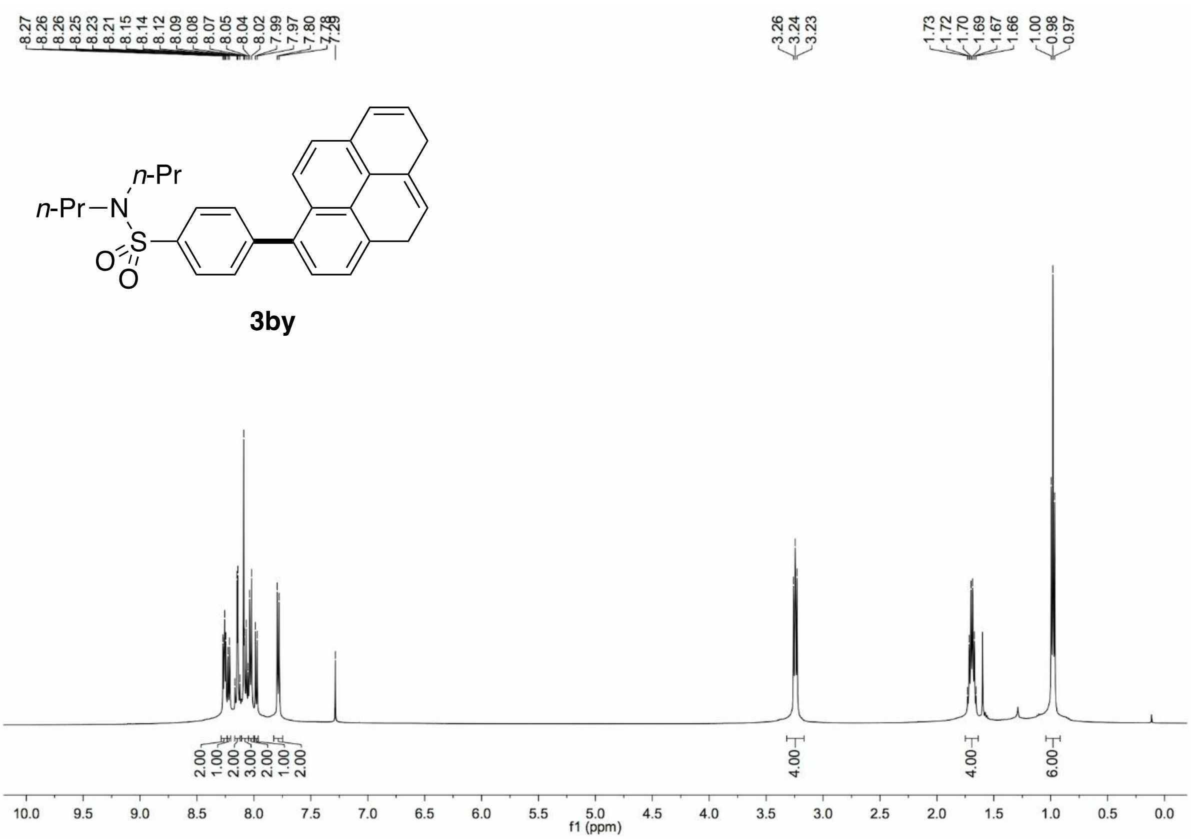

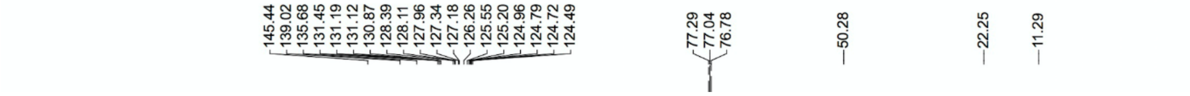

Figure S171. ^{13}C NMR spectrum of **3by**, related to **Figure 4**

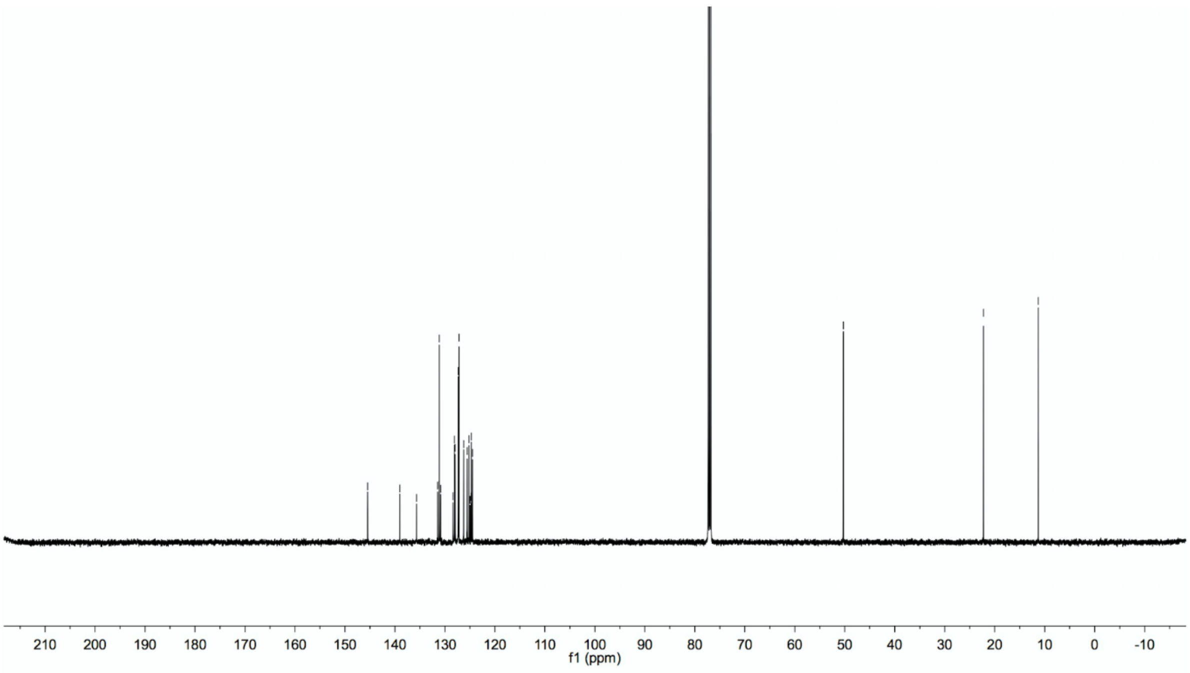

Figure S172. ^{1}H NMR spectrum of **3bz**, related to **Figure 4**

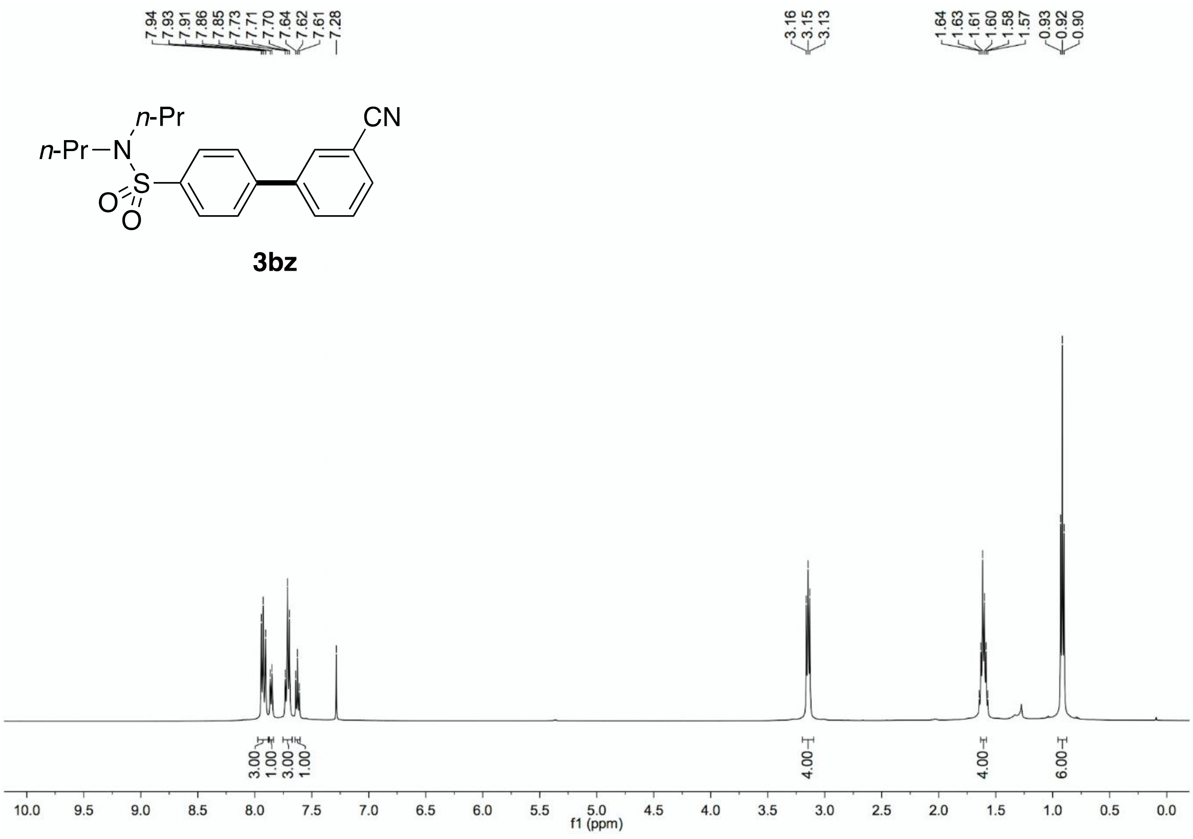

Figure S173. ^{13}C NMR spectrum of **3bz**, related to **Figure 4**

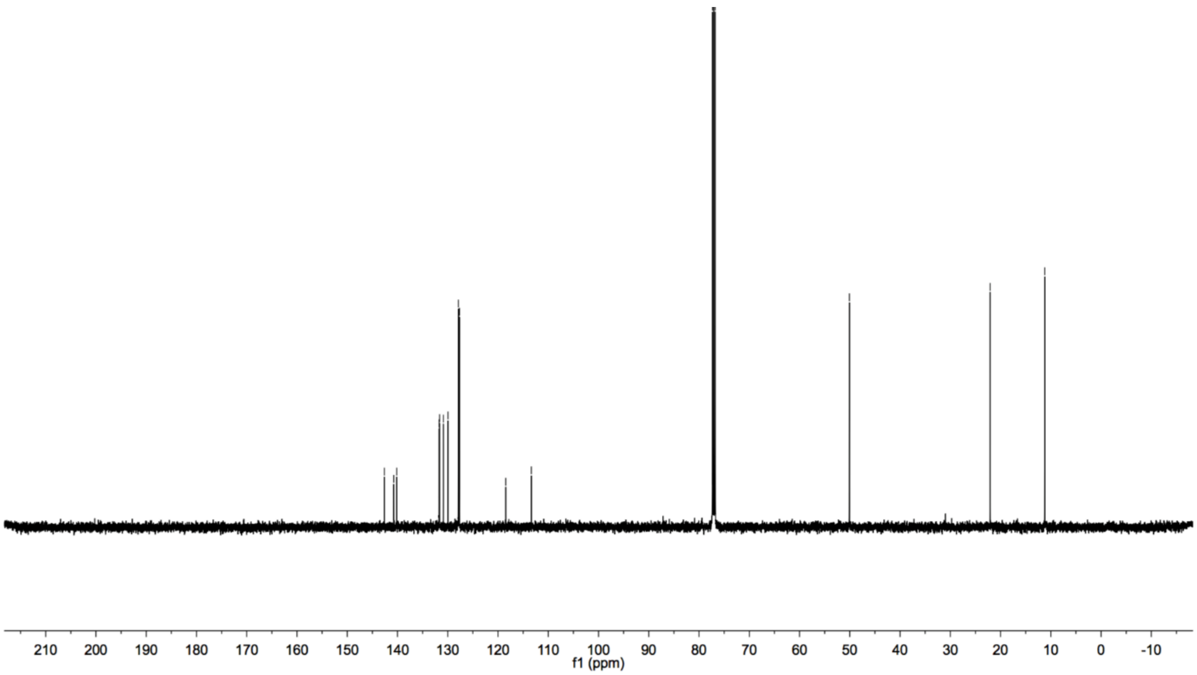

Figure S174. ^{1}H NMR spectrum of **3ca**, related to **Figure 4**

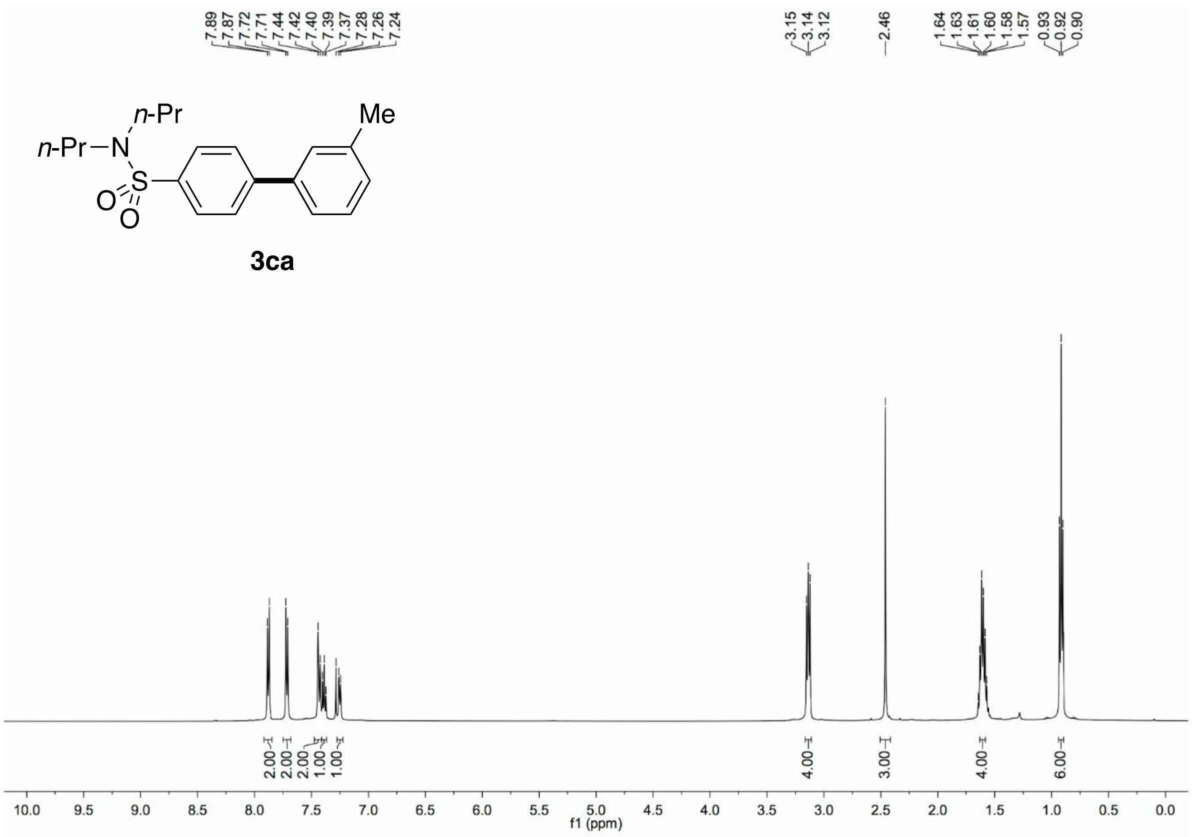

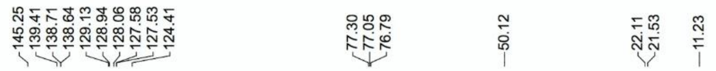

Figure S175. ^{13}C NMR spectrum of **3ca**, related to **Figure 4**

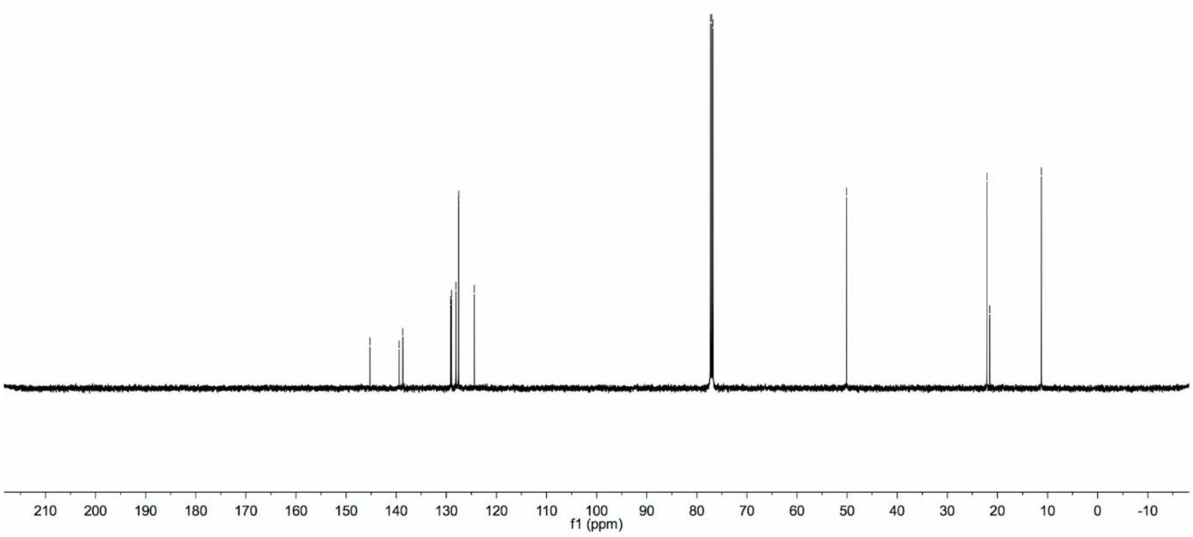

Figure S176. ^{1}H NMR spectrum of **3cb**, related to **Figure 4**

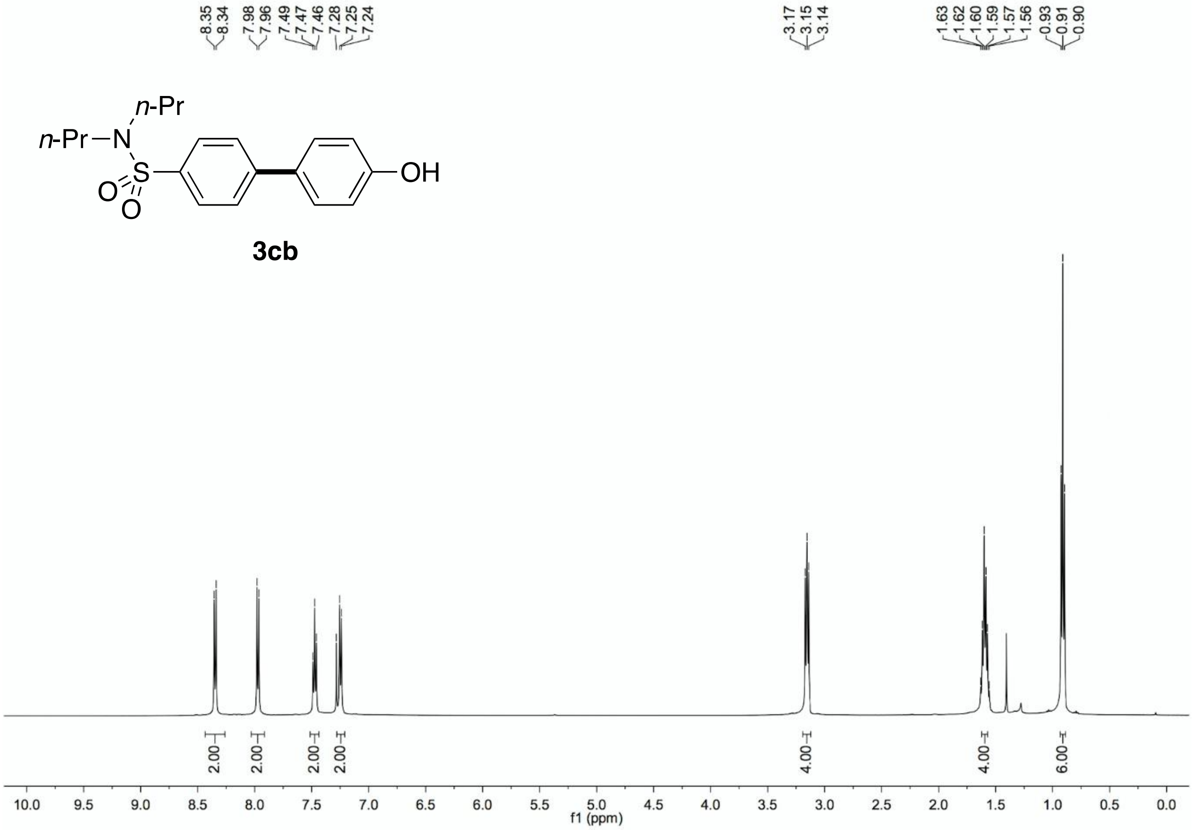

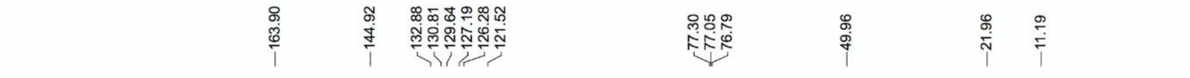

Figure S177. ^{13}C NMR spectrum of **3cb**, related to **Figure 4**

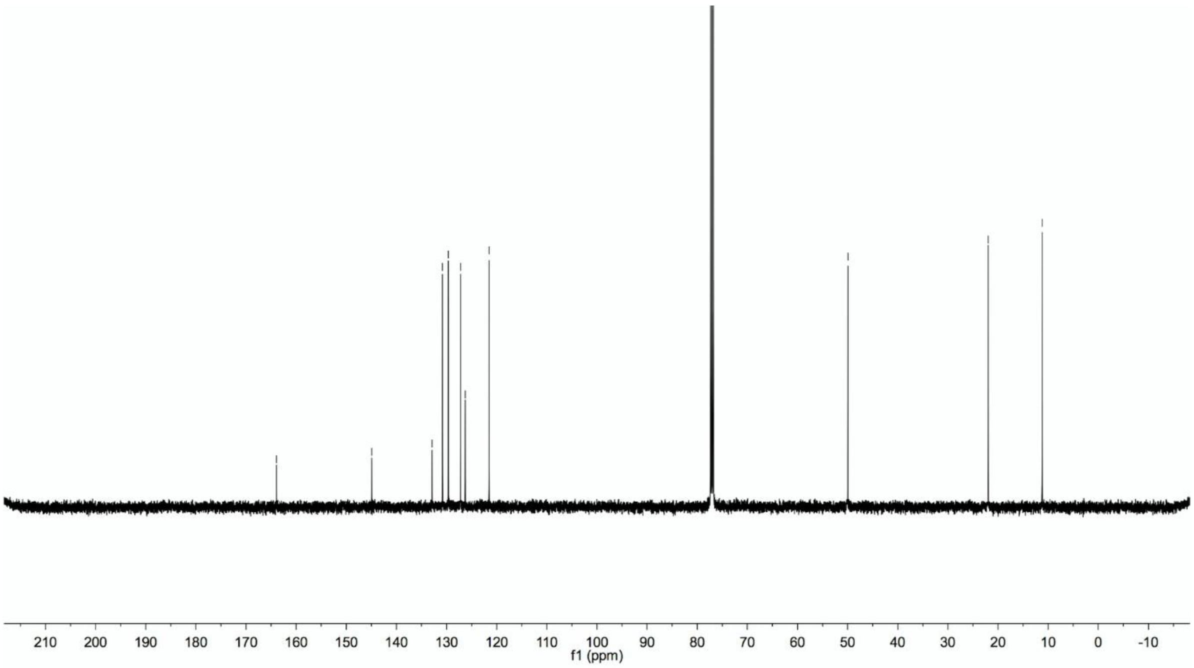

Figure S178. ^{1}H NMR spectrum of **3cc**, related to **Figure 4**

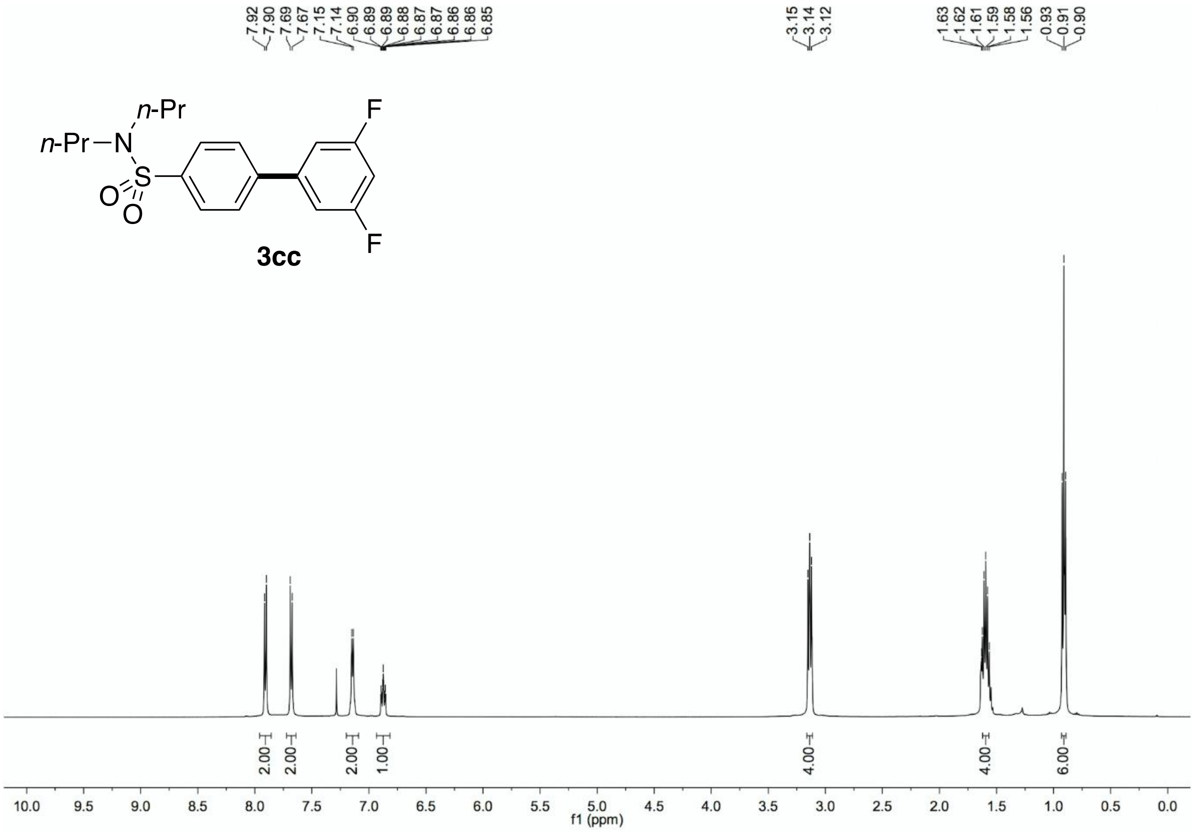

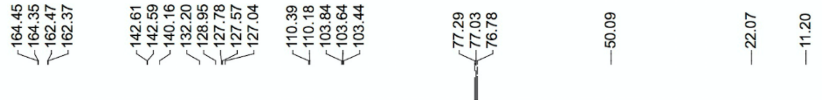

Figure S179. ^{13}C NMR spectrum of **3cc**, related to **Figure 4**

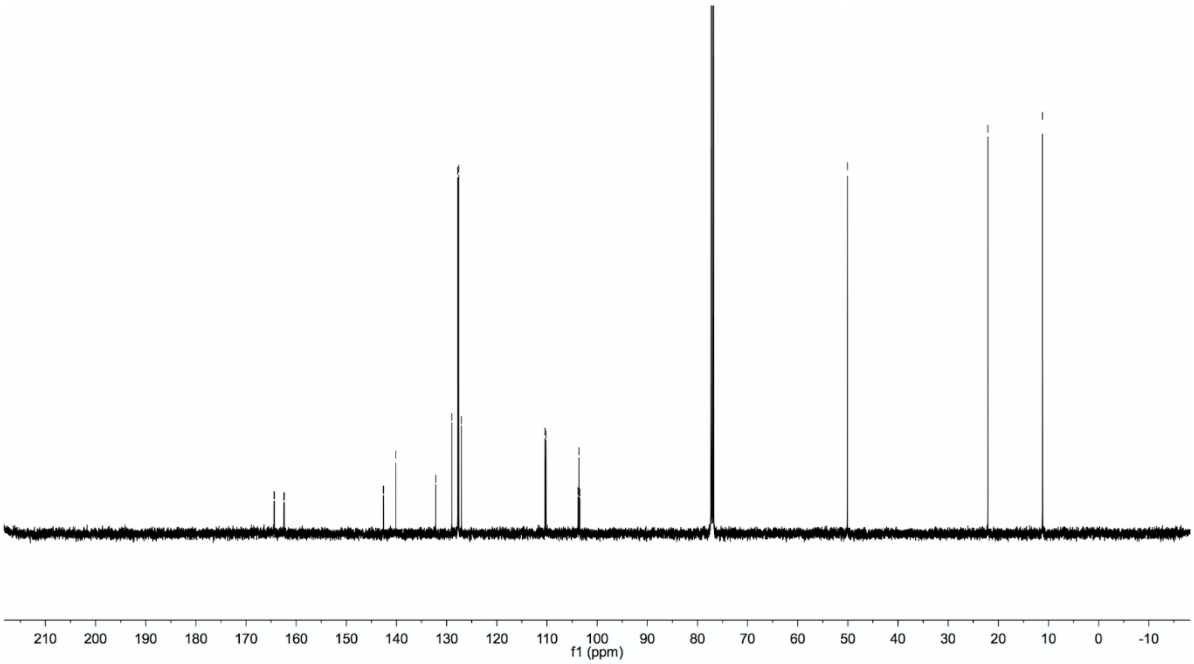

Figure S180. ^{19}F NMR spectrum of **3cc**, related to **Figure 4**

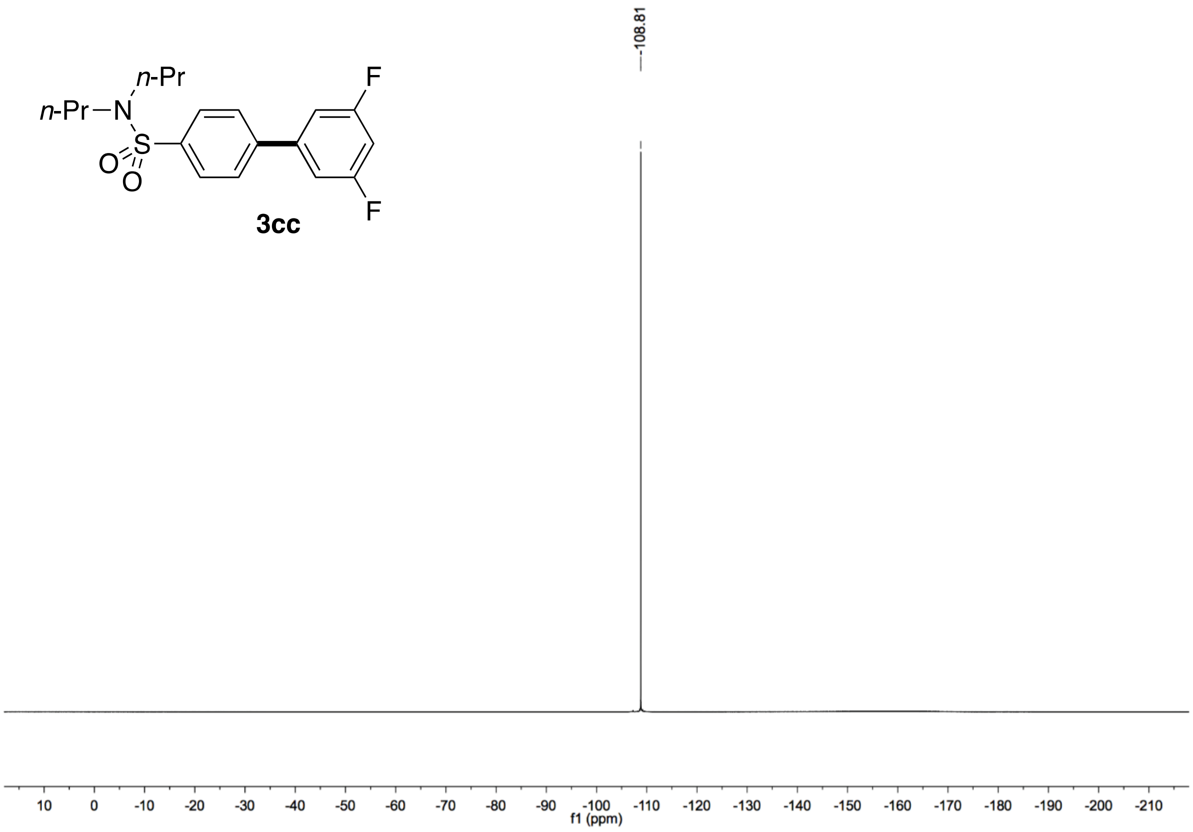

Figure S181. ^{1}H NMR spectrum of **3cd**, related to **Figure 4**

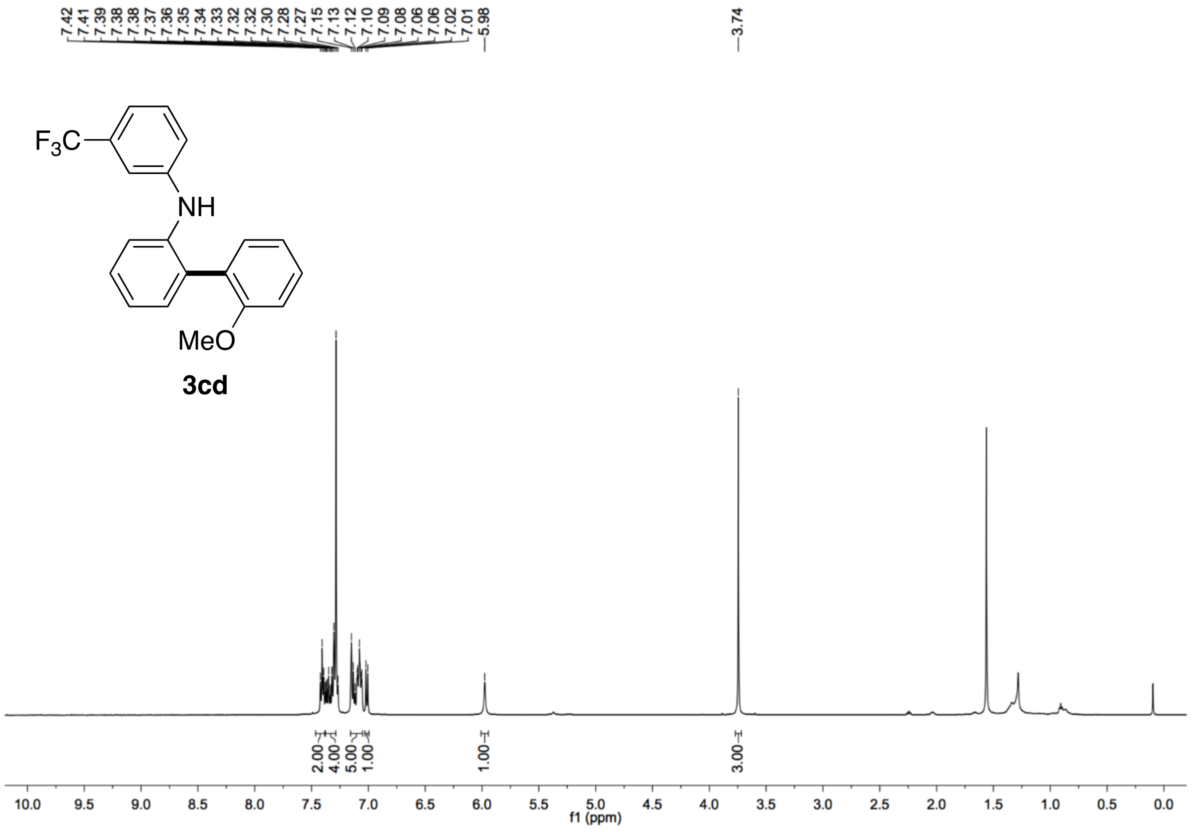

Figure S182. ^{13}C NMR spectrum of **3cd**, related to **Figure 4**

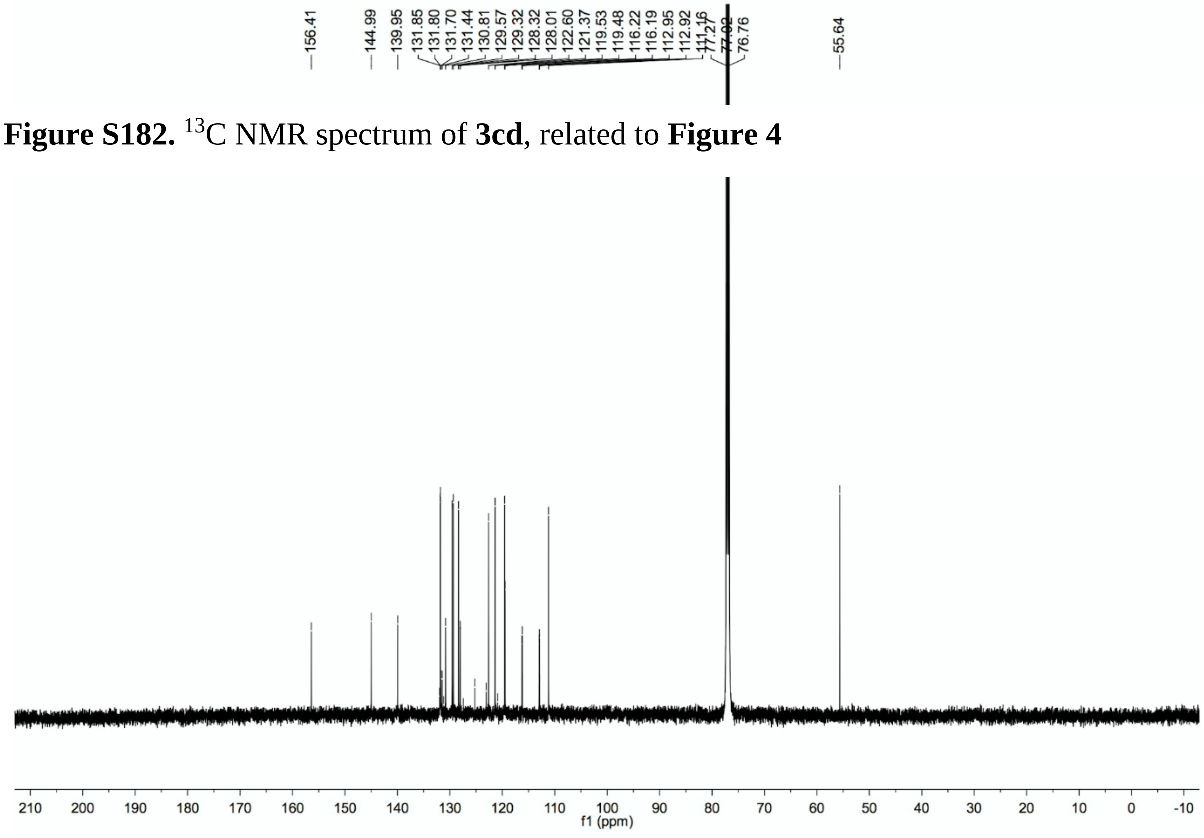

Figure S183. ^{19}F NMR spectrum of **3cd**, related to **Figure 4**

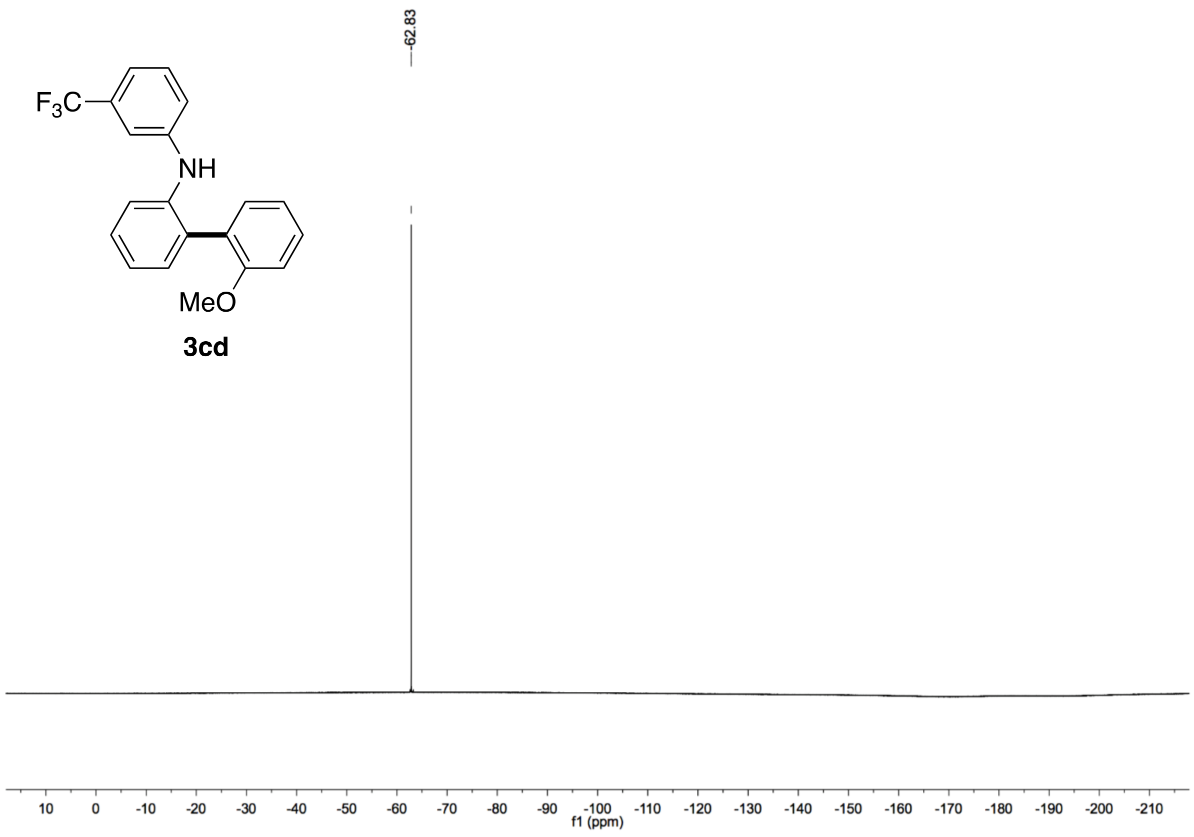

Figure S184. ^{1}H NMR spectrum of **3ce**, related to **Figure 4**

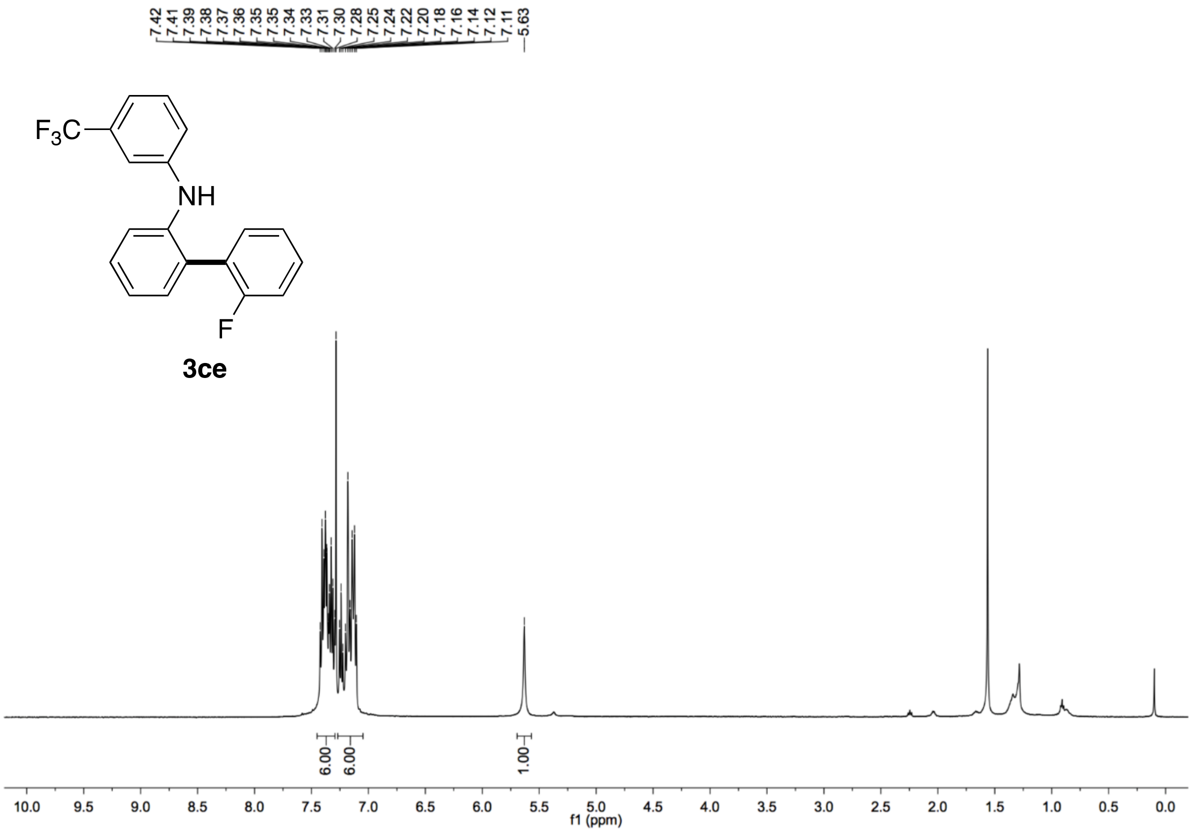

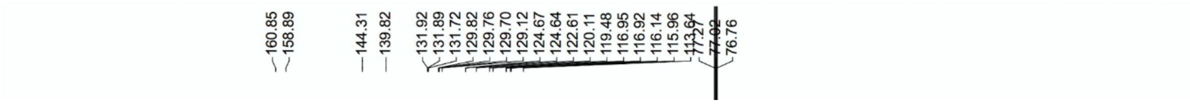

Figure S185. ^{13}C NMR spectrum of **3ce**, related to **Figure 4**

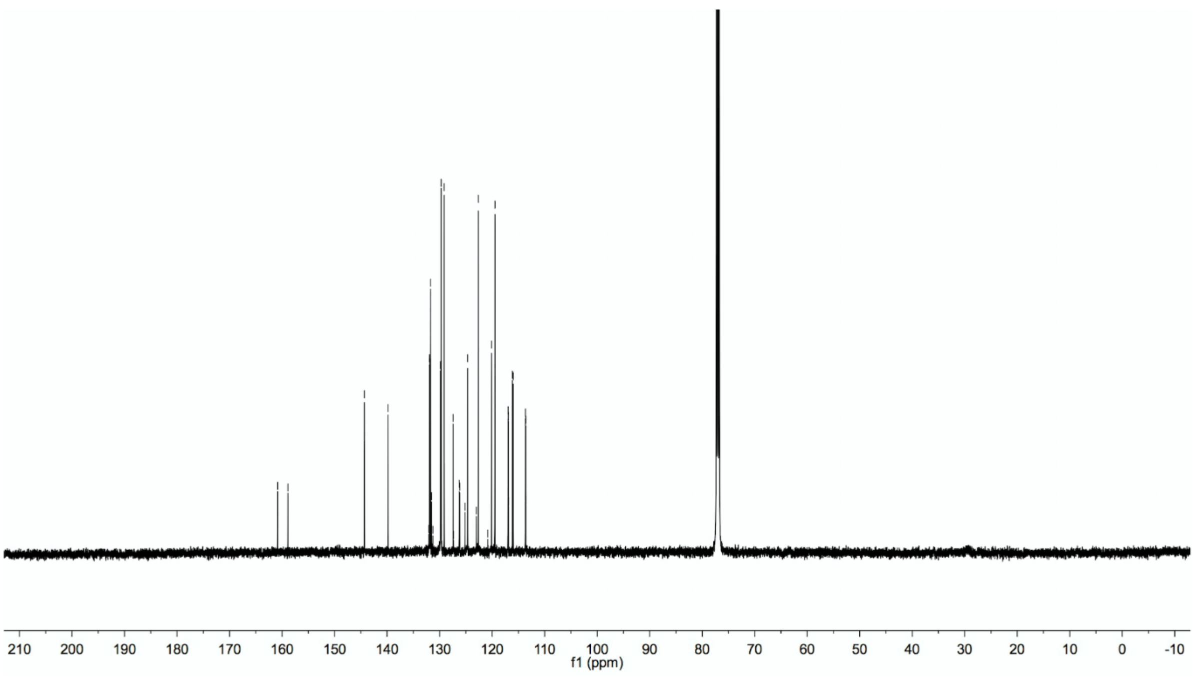

Figure S186. ^{19}F NMR spectrum of **3ce**, related to **Figure 4**

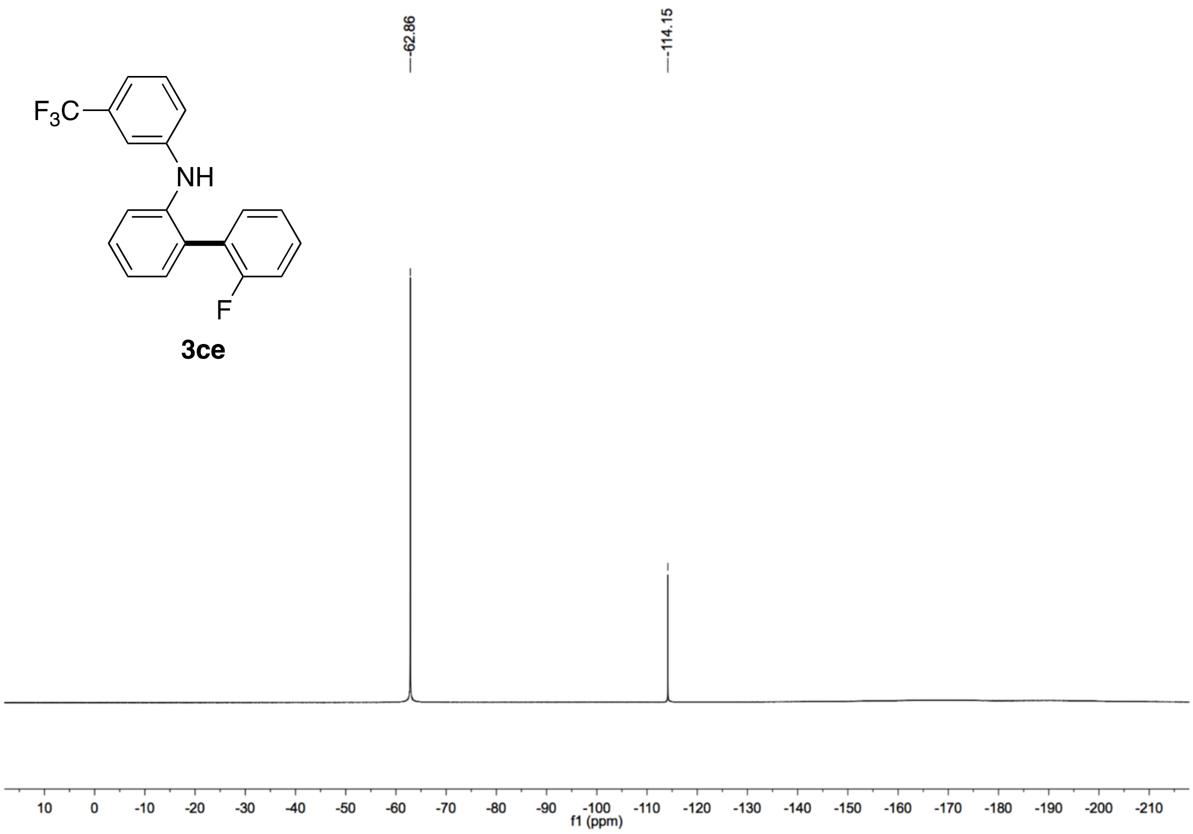

Supplemental Tables

Table S1. Table of energies, related to **Figure 2**.[a]

Structures	*ZPE*	*tcH*	*tcG*	*E*	*H*	*G*	Imaginary Frequency
4	0.720759	0.768242	0.631103	-2585.076110	-2584.307868	-2584.445007	
TS5	0.719659	0.766234	0.634892	-2585.056356	-2584.290122	-2584.421464	219.5i
6	0.721010	0.768049	0.634402	-2585.084588	-2584.316539	-2584.450186	
TS7	0.718489	0.765785	0.633776	-2585.045331	-2584.279546	-2584.411555	265.6i
8	0.718602	0.766500	0.633911	-2585.057358	-2584.290858	-2584.423447	
9	0.711880	0.756666	0.630785	-2471.763250	-2471.006584	-2471.132465	
10	0.837748	0.892049	0.737099	-2879.871451	-2878.979402	-2879.134352	
TS11	0.835418	0.889795	0.736457	-2879.856567	-2878.966772	-2879.120110	272.2i
12	0.836135	0.891635	0.733007	-2879.877414	-2878.985779	-2879.144407	
13	0.664032	0.705341	0.586658	-2356.942849	-2356.237508	-2356.356191	
TS14	0.662757	0.703609	0.585497	-2356.928693	-2356.225084	-2356.343196	316.6i
15	0.665036	0.706460	0.582992	-2356.982031	-2356.275571	-2356.399039	
16	0.182078	0.191889	0.147524	-463.048132	-462.856243	-462.900608	
TS17	0.845338	0.901703	0.742053	-2993.180885	-2992.279182	-2992.438832	274.8i
18	0.192113	0.203769	0.154571	-576.351077	-576.147308	-576.196506	
19	0.725325	0.764631	0.648919	-1865.903522	-1865.138891	-1865.254603	
20	0.725188	0.764422	0.650581	-1865.912683	-1865.148261	-1865.262102	
TS21	0.722477	0.761875	0.648158	-1865.879980	-1865.118105	-1865.231822	258.6i
TS22	0.850548	0.898210	0.767258	-2274.031585	-2273.133375	-2273.264327	275.7i
TS-S1	0.724397	0.762829	0.652398	-1865.890644	-1865.127815	-1865.238246	39.5i
S2	0.852963	0.900590	0.767260	-2274.046953	-2273.146363	-2273.279693	
S3	0.851908	0.900421	0.764789	-2274.053930	-2273.153509	-2273.289141	
S4	0.677439	0.713181	0.605797	-1751.077163	-1750.363982	-1750.471366	
TS-S5	0.677117	0.712121	0.607739	-1751.072247	-1750.360126	-1750.464508	230.5i
S6	0.679487	0.714702	0.609314	-1751.114793	-1750.400091	-1750.505479	
S7	0.722884	0.762930	0.647756	-1865.890254	-1865.127324	-1865.242498	
S8	0.715378	0.752583	0.642984	-1752.586757	-1751.834174	-1751.943773	
CO	0.005036	0.008341	-0.014102	-113.285648	-113.277307	-113.299750	
$PhB(OH)_2$	0.125189	0.134020	0.091304	-408.123464	-407.989444	-408.032160	
PhCOOPiv	0.237711	0.253481	0.194092	-691.139846	-690.886365	-690.945754	
$PivOB(OH)_2$	0.171429	0.184212	0.132914	-522.945261	-522.761049	-522.812347	

[a]Zero-point correction (*ZPE*), thermal correction to enthalpy (*TCH*), thermal correction to Gibbs free energy (*TCG*), energies (*E*), enthalpies (*H*), and Gibbs free energies (*G*) (in Hartree) of the structures calculated at the M06/6-311+G(d,p)-SDD-SMD(1,4-dioxane)//B3LYP/6-31G(d)-LANL2DZ level of theory.

Table S2. Selected optimization in decarbonylative Suzuki-Miyaura coupling of carboxylic acids, related to **Table 1**.[a]

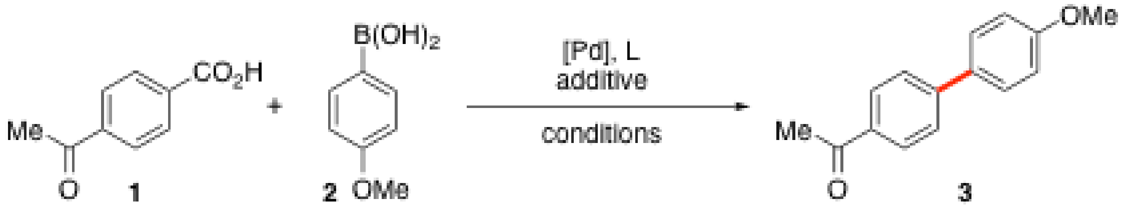

entry	[Pd]	ligand	base	additive	**1**:**2**:base:additive: piv_2O (equiv)	Yield[b] (**3**:**3'**) (%)
1	5% $Pd(OAc)_2$	10% dppb	--	--	1 : 2.0 : 2.0 : 0 : 2.0	14 : 3
2	5% $Pd(OAc)_2$	10% dppb	Et_3N	--	1 : 1.5 : 1.5 : 0 : 1.5	70 : 7
3	5% $Pd(OAc)_2$	10% dppb	Et_3N	--	1 : 2.0 : 2.0 : 0 : 2.0	67 : 8
4	5% $Pd(OAc)_2$	10% dppb	Et_3N	--	1 : 3.0 : 3.0 : 0 : 2.0	60 : 25
5	5% $Pd(OAc)_2$	10% dppb	Et_3N	--	1 : 4.0 : 4.0 : 0 : 2.0	58 : 11
6	5% $Pd(OAc)_2$	6% dppb	Et_3N	--	1 : 2.0 : 2.0 : 0 : 2.0	42 : 6
7	2.5% $Pd(OAc)_2$	5% dppb	Et_3N	--	1 : 2.0 : 2.0 : 0 : 2.0	76 : 3
8[c]	5% $Pd(OAc)_2$	10% dppb	Et_3N	--	1 : 2.0 : 2.0 : 0 : 2.0	49: 9
9	5% $Pd(OAc)_2$	10% dppb	Na_2CO_3	--	1 : 2.0 : 2.0 : 0 : 2.0	52 : 15
10	5% $Pd(OAc)_2$	10% dppb	K_2CO_3	--	1 : 2.0 : 2.0 : 0 : 2.0	51 : 23
11	5% $Pd(OAc)_2$	10% dppb	K_3PO_4	--	1 : 2.0 : 2.0 : 0 : 2.0	<2 : <2
12	5% $Pd(OAc)_2$	10% dppb	Et_3N	Na_2CO_3	1 : 2.0 : 2.0 : 2.0 : 2.0	59 : 12
13	5% $Pd(OAc)_2$	10% dppb	Et_3N	K_2CO_3	1 : 2.0 : 2.0 : 2.0 : 2.0	39 : 19
14	5% $Pd(OAc)_2$	10% dppb	Et_3N	K_3PO_4	1 : 2.0 : 2.0 : 2.0 : 2.0	3 : 5
15	5% $Pd(OAc)_2$	10% dppb	Et_3N	H_3BO_3	1 : 2.0 : 2.0 : 2.0 : 2.0	82 : 15
16	5% $Pd(OAc)_2$	10% dppb	Et_3N	$AdCO_2H$	1 : 2.0 : 2.0 : 2.0 : 2.0	<2 : 0
17	5% $Pd(OAc)_2$	10% dppb	Et_3N	PivOH	1 : 2.0 : 2.0 : 2.0 : 2.0	73 : 4
18	2.5% $Pd(OAc)_2$	5% dppb	Et_3N	PivOH	1 : 1.2 : 1.2 : 1.2 : 1.2	56 : <2
19	5% $Pd(OAc)_2$	10% dppb	Et_3N	H_2O	1 : 2.0 : 2.0 : 2.0 : 2.0	64 : 25
20	5% $Pd(OAc)_2$	10% dppb	Na_2CO_3	H_3BO_3	1 : 2.0 : 2.0 : 2.0 : 2.0	54 : 8
21	5% $Pd(OAc)_2$	10% dppb	K_2CO_3	H_3BO_3	1 : 2.0 : 2.0 : 2.0 : 2.0	59 : 9
22	5% $Pd(OAc)_2$	10% dppb	Et_3N	Na_2CO_3, H_3BO_3	1 : 2.0 : 2.0 : 2.0 : 2.0 :2.0	80 : 12
23	5% $Pd(OAc)_2$	10% dppb	Et_3N	K_2CO_3, H_3BO_3	1 : 2.0 : 2.0 : 2.0 : 2.0 :2.0	71 : 13
24	5% $Pd(OAc)_2$	10% dppb	DIPEA	H_3BO_3	1 : 2.0 : 2.0 : 2.0 : 2.0	69 : 14
25	5% $Pd(OAc)_2$	10% dppb	Bu_3N	H_3BO_3	1 : 2.0 : 2.0 : 2.0 : 2.0	60 : 4
26	5% $Pd(OAc)_2$	10% dppb	DMAP	H_3BO_3	1 : 2.0 : 2.0 : 2.0 : 2.0	43 : <2
27	5% $Pd(OAc)_2$	10% dppb	Pyridine	H_3BO_3	1 : 2.0 : 2.0 : 2.0 : 2.0	43 : 6
28	5% $Pd(OAc)_2$	10% dppb	NMM	H_3BO_3	1 : 2.0 : 2.0 : 2.0 : 2.0	53 : 13

29	5% $Pd(OAc)_2$	10% dppb	Et_2NH	H_3BO_3	1 : 2.0 : 2.0 : 2.0 : 2.0	21 : 3
30[d]	5% $Pd(OAc)_2$	10% dppb	Et_3N	H_3BO_3	1 : 2.0 : 2.0 : 2.0 : 2.0	44 : 9
31[e]	5% $Pd(OAc)_2$	10% dppb	Et_3N	H_3BO_3	1 : 2.0 : 2.0 : 2.0 : 2.0	80 : 7
32[f]	5% $Pd(OAc)_2$	10% dppb	Et_3N	H_3BO_3	1 : 2.0 : 2.0 : 2.0 : 2.0	50 : 14
33[g]	5% $Pd(OAc)_2$	10% dppb	Et_3N	H_3BO_3	1 : 2.0 : 2.0 : 2.0 : 2.0	73 : 12
34	5% $PdCl_2$	10% dppb	Et_3N	H_3BO_3	1 : 2.0 : 2.0 : 2.0 : 2.0	55 : 4
35	10% $Pd_2(dba)_3$	20% dppb	Et_3N	H_3BO_3	1 : 2.0 : 2.0 : 2.0 : 2.0	79 : 9
36	5% $Pd_2(dba)_3$	10% dppb	Et_3N	H_3BO_3	1 : 2.0 : 2.0 : 2.0 : 2.0	70 : 10
37	2.5% $Pd_2(dba)_3$	5% dppb	Et_3N	H_3BO_3	1 : 2.0 : 2.0 : 2.0 : 2.0	80 : 10
38	10% $Pd_2(dba)_3$	40% dppb	Et_3N	H_3BO_3	1 : 2.0 : 2.0 : 2.0 : 2.0	31 : 2
39	5% $Pd_2(dba)_3$	20% dppb	Et_3N	H_3BO_3	1 : 2.0 : 2.0 : 2.0 : 2.0	67 : 10
40	2.5% $Pd_2(dba)_3$	10% dppb	Et_3N	H_3BO_3	1 : 2.0 : 2.0 : 2.0 : 2.0	66 : 8
41	5% $Pd(dba)_2$	10% dppb	Et_3N	H_3BO_3	1 : 2.0 : 2.0 : 2.0 : 2.0	59 : 8
42	5% PEPPSI	10% dppb	Et_3N	H_3BO_3	1 : 2.0 : 2.0 : 2.0 : 2.0	0 : 0
43	5% Neolyst	10% dppb	Et_3N	H_3BO_3	1 : 2.0 : 2.0 : 2.0 : 2.0	<2 : 0
44	5% $Pd(OAc)_2$	10% dppm	Et_3N	H_3BO_3	1 : 2.0 : 2.0 : 2.0 : 2.0	9 : 2
45	5% $Pd(OAc)_2$	10% dppe	Et_3N	H_3BO_3	1 : 2.0 : 2.0 : 2.0 : 2.0	0 : 0
46	5% $Pd(OAc)_2$	10% dppp	Et_3N	H_3BO_3	1 : 2.0 : 2.0 : 2.0 : 2.0	5 : <2
47	5% $Pd(OAc)_2$	10% dpppe	Et_3N	H_3BO_3	1 : 2.0 : 2.0 : 2.0 : 2.0	44 : 21
48	5% $Pd(OAc)_2$	10% dpphex	Et_3N	H_3BO_3	1 : 2.0 : 2.0 : 2.0 : 2.0	18 : 33
49	5% $Pd(OAc)_2$	10% BINAP	Et_3N	H_3BO_3	1 : 2.0 : 2.0 : 2.0 : 2.0	27 : 27
50	5% $Pd(OAc)_2$	10% DPEPhos	Et_3N	H_3BO_3	1 : 2.0 : 2.0 : 2.0 : 2.0	47 : 14
51	5% $Pd(OAc)_2$	10% XantPhos	Et_3N	H_3BO_3	1 : 2.0 : 2.0 : 2.0 : 2.0	26 : 3
52	5% $Pd(OAc)_2$	10% dppf	Et_3N	H_3BO_3	1 : 2.0 : 2.0 : 2.0 : 2.0	41 : 8
53	5% $Pd(OAc)_2$	20% PCy_3HBF_4	Et_3N	H_3BO_3	1 : 2.0 : 2.0 : 2.0 : 2.0	15 : 68
54	5% $Pd(OAc)_2$	20% PCy_2Ph	Et_3N	H_3BO_3	1 : 2.0 : 2.0 : 2.0 : 2.0	13 : 87
55	5% $Pd(OAc)_2$	20% $PCyPh_2$	Et_3N	H_3BO_3	1 : 2.0 : 2.0 : 2.0 : 2.0	17 : 72
56	5% $Pd(OAc)_2$	20% PPh_3	Et_3N	H_3BO_3	1 : 2.0 : 2.0 : 2.0 : 2.0	24 : 61
57	5% $Pd(OAc)_2$	20% DavePhos	Et_3N	H_3BO_3	1 : 2.0 : 2.0 : 2.0 : 2.0	<2 : <2
58	5% $Pd(OAc)_2$	20% XPhos	Et_3N	H_3BO_3	1 : 2.0 : 2.0 : 2.0 : 2.0	<2 : <2
59	5% $Pd(OAc)_2$	20% SPhos	Et_3N	H_3BO_3	1 : 2.0 : 2.0 : 2.0 : 2.0	<2 : <2
60	5% $Pd(OAc)_2$	10% dppb	Et_3N	H_3BO_3	1 : 1.2 : 1.2 : 1.2 : 1.2	48 : 7
61	5% $Pd(OAc)_2$	10% dppb	Et_3N	H_3BO_3	1 : 1.5 : 1.5 : 1.5 : 1.5	79 : 6
62	5% $Pd(OAc)_2$	10% dppb	Et_3N	H_3BO_3	1 : 1.5 : 4.5 : 2.0 : 2.0	79 : 9
63	5% $Pd(OAc)_2$	10% dppb	Et_3N	H_3BO_3	1 : 2.0 : 1.5 : 1.5 : 1.5	82 : 8
64	5% $Pd(OAc)_2$	10% dppb	Et_3N	H_3BO_3	1 : 2.0 : 2.0 : 1.5 : 1.5	84 : 8
65	5% $Pd(OAc)_2$	10% dppb	Et_3N	H_3BO_3	1 : 2.0 : 2.0 : 1.0 : 2.0	82 : 15
66	5% $Pd(OAc)_2$	10% dppb	Et_3N	H_3BO_3	1 : 2.0 : 2.0 : 1.5 : 2.0	82 : 11
67	5% $Pd(OAc)_2$	10% dppb	Et_3N	H_3BO_3	1 : 2.0 : 2.0 : 4.0 : 2.0	77 : 14

68	5% $Pd(OAc)_2$	10% dppb	Et_3N	H_3BO_3	1 : 2.0 : 4.0 : 2.0 : 2.0	75 : 10
69	5% $Pd(OAc)_2$	10% dppb	Et_3N	H_3BO_3	1 : 2.0 : 4.0 : 4.0 : 2.0	82 : 10
70	5% $Pd(OAc)_2$	10% dppb	Et_3N	H_3BO_3	1 : 3.0 : 1.5 : 2.0 : 2.0	77 : 14
71	5% $Pd(OAc)_2$	10% dppb	Et_3N	H_3BO_3	1 : 3.0 : 4.5 : 2.0 : 2.0	63 : 12
72	5% $Pd(OAc)_2$	10% dppb	Et_3N	H_3BO_3	1 : 2.0 : 1.5 : 1.5 : 1.5	72 : 15
73[h]	5% $Pd(OAc)_2$	10% dppb	Et_3N	H_3BO_3	1 : 2.0 : 1.5 : 1.5 : 1.5	48 : 10
74[i]	5% $Pd(OAc)_2$	10% dppb	Et_3N	H_3BO_3	1 : 2.0 : 1.5 : 1.5 : 1.5	38 : 11

[a]Conditions: carboxylic acid (1.0 equiv), boronic acid (1.2-4.0 equiv), base (1.5-4.5 equiv), 1,4-dioxane (0.20 M), 160 °C, 15 h; [b]Determined by ^{1}H NMR and/or GC-MS; [c]140 °C; [d]Toluene; [e]0.10 M; [f]0.50 M; [g]100 mg MS; [h]120 °C; [i]100 °C. **3** = 1-(4'-methoxy-[1,1'-biphenyl]-4-yl)ethan-1-one; **3’** = 1-(4-(4-methoxybenzoyl)phenyl)ethan-1-one.

Transparent Methods

Computational Details

All density functional theory (DFT) calculations were performed with Gaussian 09 package (Frisch et al., 2010). Geometry optimizations of all the minima and transition states were carried out at the B3LYP (Becke et al., 1993; Lee et al., 1988) level of theory, with the LANL2DZ (Hay et al., 1985; Wadt et al., 1985; Hay et al., 1985) basis set for Pd and the 6-31G(d) basis set for the other atoms. Vibrational frequencies were computed at the same level of theory to evaluate its zero-point vibrational energy (ZPVE) and thermal corrections at 298 K, and to check whether each optimized structure is an energy minimum or a transition state. On the basis of the gas-phase optimized structures, the single point energies were calculated with the M06 functional developed by Truhlar and coworkers (Zhao et al., 2008; Zhao et al., 2008) and a mixed basis set of SDD (von Szentpaly et al., 1982; Dolg et al., 1987; Schwerdtfeger et al., 1898) for Pd and 6-311+G(d,p) for other atoms. Solvation energy corrections were calculated using the SMD model (Marenich et al., 2009) with 1,4-dioxane as the solvent. The 3D diagrams of computed species were generated using CYLView (Legault, 2009). In order to adjust the Gibbs free energies from 1 atm to 1 mol/L, a correction of $R\mathrm{Tln}(c_s/c_g)$ (about 1.9 kcal/mol) is added to energies of all species. c_s is the standard molar concentration in solution (1 mol/L), c_g is the standard molar concentration in gas phase (0.0446 mol/L), and R is the gas constant. Extensive conformational searches are conducted to ensure that the most stable conformers are located.

List of Known Compounds/General Methods

All starting materials reported in the manuscript have been previously described in literature or prepared by the method reported previously. All experiments involving palladium were performed using standard Schlenk techniques under argon atmosphere unless stated otherwise. All solvents were purchased at the highest commercial grade and used as received or after purification by passing through activated alumina columns or distillation from sodium/benzophenone under nitrogen. All solvents were deoxygenated prior to use. All other chemicals were purchased at the highest commercial grade and used as received. Reaction glassware was oven-dried at 140 °C for at least 24 h or flame-dried prior to use, allowed to cool under vacuum and purged with argon (three cycles). All products were identified using ^{1}H NMR analysis and comparison with authentic samples. GC and/or GC/MS analysis was used for volatile products. All yields refer to yields determined by ^{1}H NMR and/or GC or GC/MS using an internal standard (optimization) and isolated yields (preparative runs) unless stated otherwise. ^{1}H NMR and ^{13}C NMR spectra were recorded in $CDCl_3$ on Bruker spectrometers at 500 (^{1}H NMR) and 125 MHz (^{13}C NMR). All shifts are reported in parts per million (ppm) relative to residual $CHCl_3$ peak (7.27 and 77.2 ppm, ^{1}H NMR and ^{13}C NMR, respectively). All coupling constants (*J*) are reported in hertz (Hz). Abbreviations are: s, singlet; d, doublet; t, triplet; q, quartet; brs, broad singlet. GC-MS chromatography was performed using Agilent HP6890 GC System and Agilent 5973A inert XL EI/CI MSD using helium as the carrier gas at a flow rate of 1 mL/min and an initial oven temperature of 50 °C. The injector temperature was 250 °C. The detector temperature was 250 °C. For runs with the initial oven temperature of 50 °C, temperature was increased with a 10 °C/min ramp after 50 °C hold for 3 min to a final temperature of 220 °C, then hold at 220 °C for 15 min (splitless mode of injection, total run time of 22.0 min). High-resolution mass spectra (HRMS) were measured on a 7T Bruker Daltonics FT-MS instrument (for HRMS). Melting point was measured on MeltEMP (laboratory devices). All flash chromatography was performed using silica gel, 60 Å, 300 mesh. TLC analysis was carried out on glass plates coated with silica gel 60 F254, 0.2 mm thickness. The plates were visualized using a 254 nm ultraviolet lamp or aqueous potassium permanganate solutions. ^{1}H NMR and ^{13}C NMR data are given for all compounds. ^{1}H NMR, ^{13}C NMR and HRMS data are reported for all new compounds.

Experimental Procedures and Characterization Data

General Procedure for Decarbonylative Suzuki-Miyaura Coupling of Carboxylic Acids. An oven-dried vial equipped with a stir bar was charged with carboxylic acid (neat, 1.0 equiv), boronic acid (neat, typically, 2.0 equiv), $Pd(OAc)_2$ (typically, 5 mol%), ligand (typically, 10 mol%), triethylamine (typically, 1.5 equiv), boric acid (typically, 1.5 equiv) and trimethylacetic anhydride (typically, 1.5 equiv), placed under a positive pressure of argon, and subjected to three evacuation/backfilling cycles under high vacuum. Dioxane (0.20 M) was added with vigorous stirring at room temperature, the reaction mixture was placed in a preheated oil bath at 160 °C, and stirred for the indicated time at 160 °C.

Representative Procedure for Decarbonylative Suzuki-Miyaura Coupling of Carboxylic Acids. An oven-dried vial equipped with a stir bar was charged with 4-acetylbenzoic acid (neat, 32.9 mg, 0.20 mmol), phenylboronic acid (neat, 48.8 mg, 0.40 mmol, 2.0 equiv), $Pd(OAc)_2$ (2.3 mg, 5 mol%), 1,4-bis(diphenylphosphino)butane (8.6 mg, 10 mol%), triethylamine (30.4 mg, 0.30 mmol, 1.5 equiv), boric acid (18.6 mg, 0.30 mmol, 1.5 equiv) and trimethylacetic anhydride (55.9 mg, 0.30 mmol, 1.5 equiv), placed under a positive pressure of argon, and subjected to three evacuation/backfilling cycles under high vacuum. Dioxane (1.0 mL, 0.20 M) was added with vigorous stirring at room temperature, the reaction mixture was placed in a preheated oil bath at 160 °C, and stirred for 15 h at 160 °C. After the indicated time, the reaction mixture was cooled down to room temperature, diluted with CH_2Cl_2 (10 mL), filtered, and concentrated. Purification by chromatography on silica gel (ethyl acetate/hexane) afforded the title product. Yield 83% (32.6 mg). White solid. Characterization data are included in the section below.

Representative Procedure for Decarbonylative Suzuki-Miyaura Coupling of Carboxylic Acids. Gram Scale. An oven-dried vial equipped with a stir bar was charged with probenecid (*p*-(dipropylsulfamoyl)benzoic acid, neat, 1.00 g, 3.50 mmol), phenylboronic acid (neat, 0.855 g, 7.00 mmol, 2.0 equiv), $Pd(OAc)_2$ (39.3 mg, 5 mol%), 1,4-bis(diphenylphosphino)butane (149.3 mg, 10 mol%), triethylamine (0.531 g, 5.25 mmol, 1.5 equiv), boric acid (0.324 g, 5.25 mmol, 1.5 equiv) and trimethylacetic anhydride (0.978 g, 5.25 mmol, 1.5 equiv), placed under a positive pressure of argon, and subjected to three evacuation/backfilling cycles under high vacuum. Dioxane (17.5 mL, 0.20 M) was added with vigorous stirring at room temperature, the reaction

mixture was placed in a preheated oil bath at 160 °C, and stirred for 15 h at 160 °C. After the indicated time, the reaction mixture was cooled down to room temperature, diluted with CH_2Cl_2 (50 mL), filtered, and concentrated. Purification by chromatography on silica gel (ethyl acetate/hexane) afforded the title product. Yield 86% (0.958 g). White solid. Characterization data are included in the section below.

Mechanistic Studies

A series of synthetic studies were performed to gain insight into the reaction mechanism and investigate factors involved in controlling the Pd-catalyzed decarbonylative Suzuki-Miyaura coupling of carboxylic acids (Schemes S1-S6).

(1) To investigate whether benzoic pivalic anhydride was a possible reaction intermediate, benzoic pivalic anhydride **1S** was prepared and subjected to the reaction conditions (Scheme S1). Formation of product **3S** from **1S** was observed using Pd-catalysis, suggesting that **1S** could be a competent intermediate. Moreover, benzoic anhydride served as a competent intermediate under Pd-catalyzed conditions (45% yield, not shown).

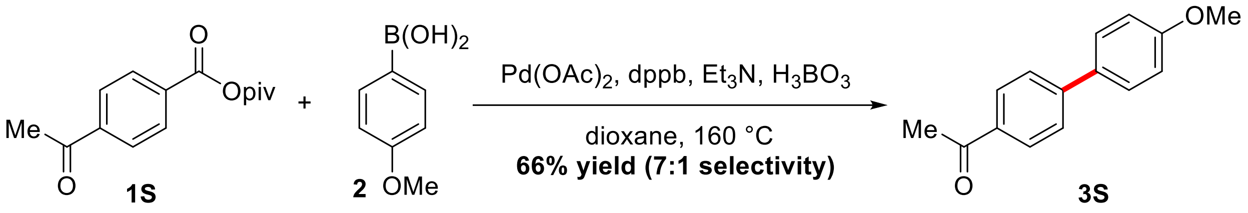

Scheme S1. Decarbonylative Suzuki-Miyaura of benzoic pivalic anhydride, related to Figure 1. *Conditions:* ***1S*** *(1.0 equiv), 4-MeO-C_6H_4-$B(OH)_2$ (2.0 equiv), $Pd(OAc)_2$ (5 mol%), dppb (10 mol%), Et_3N (2.0 equiv), H_3BO_3 (2.0 equiv), dioxane (0.20 M), 160 °C, 15 h.*

(2) To investigate electronic effect on the decarbonylative coupling, intermolecular competition experiments between differently substituted carboxylic acids were conducted (Scheme S2). The experiments revealed that electron-deficient arenes are inherently more reactive than their electron-rich counterparts, consistent with facility of metal insertion and decarbonylation.

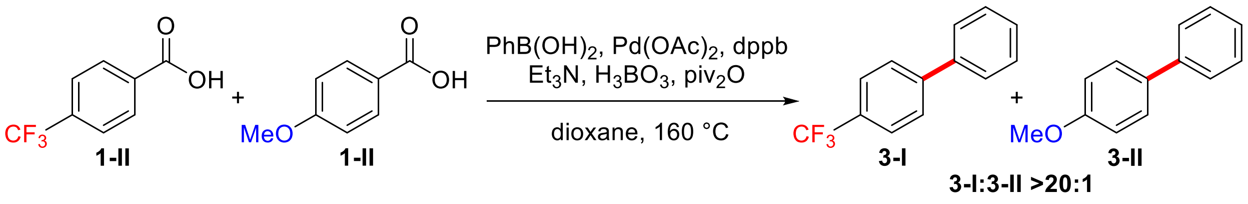

Scheme S2. Intermolecular competition experiments in decarbonylative Suzuki-Miyaura coupling of carboxylic acids, related to Figure 1. *Conditions:* ***1*** *(1.0 equiv each), $PhB(OH)_2$*

(0.5 equiv), Pd(OAc)$_2$ (5 mol%), dppb (10 mol%), Et$_3$N (1.5 equiv), H$_3$BO$_3$ (1.5 equiv), piv$_2$O (1.5 equiv), dioxane (0.20 M), 160 °C, 15 h.

(3) To investigate steric effect on the decarbonylative coupling, intermolecular competition experiments between differently substituted carboxylic acids were conducted (Scheme S3). The experiments revealed that sterically-hindered carboxylic acids react preferentially, consistent with decarbonylation favored by steric demand of acylpalladium complexes.

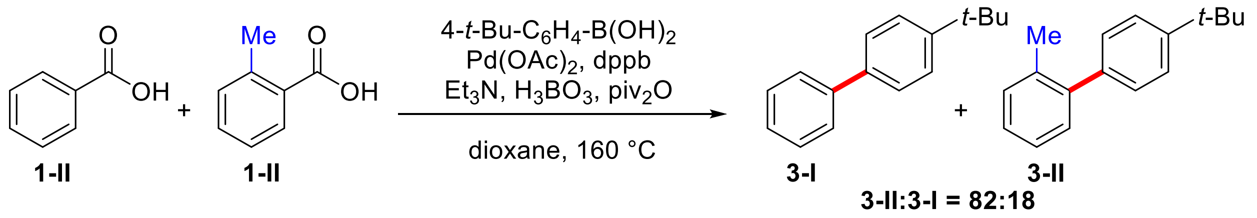

Scheme S3. Intermolecular competition experiments in decarbonylative Suzuki-Miyaura coupling of carboxylic acids, related to Figure 1. *Conditions:* ***1*** *(1.0 equiv each), 4-t-Bu-C$_6$H$_4$-B(OH)$_2$ (0.5 equiv), Pd(OAc)$_2$ (5 mol%), dppb (10 mol%), Et$_3$N (1.5 equiv), H$_3$BO$_3$ (1.5 equiv), piv$_2$O (1.5 equiv), dioxane (0.20 M), 160 °C, 15 h.*

(4) To investigate electronic effect on the boronic acid component on the decarbonylative coupling, intermolecular competition experiments between differently substituted boronic acids were conducted (Scheme S4). The experiments revealed that electron-rich boronic acids are inherently more reactive than their electron-deficient counterparts, consistent with facility of transmetallation.

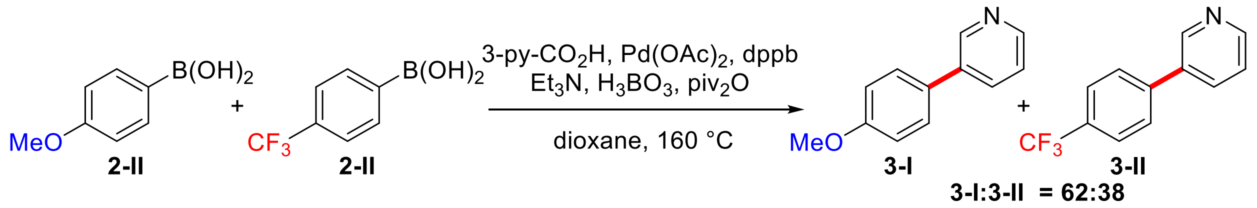

Scheme S4. Intermolecular competition experiments in decarbonylative Suzuki-Miyaura coupling of carboxylic acids, related to Figure 1. *Conditions:* ***2*** *(1.0 equiv each), 3-py-CO$_2$H (0.5 equiv), Pd(OAc)$_2$ (5 mol%), dppb (10 mol%), Et$_3$N (1.5 equiv), H$_3$BO$_3$ (1.5 equiv), piv$_2$O (1.5 equiv), dioxane (0.20 M), 160 °C, 15 h.*

(5) To investigate steric effect on the boronic acid component on the decarbonylative coupling, intermolecular competition experiments between differently substituted boronic acids were conducted (Scheme S5). The experiments revealed that electron-rich boronic acids are inherently more reactive than their electron-deficient counterparts, consistent with decarbonylation favored by steric demand of acylpalladium complexes.

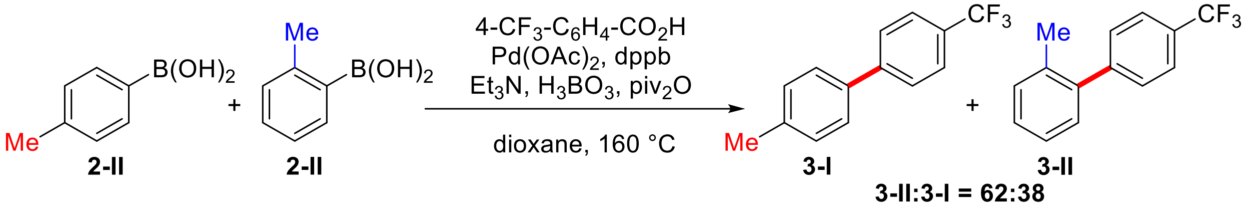

Scheme S5. Intermolecular competition experiments in decarbonylative Suzuki-Miyaura coupling of carboxylic acids, related to Figure 1. *Conditions:* ***2*** *(1.0 equiv each), 4-*CF_3*-*C_6H_4*-*CO_2H *(0.5 equiv), Pd*$(OAc)_2$ *(5 mol%), dppb (10 mol%),* Et_3N *(1.5 equiv),* H_3BO_3 *(1.5 equiv), piv*$_2$*O (1.5 equiv), dioxane (0.20 M), 160 °C, 15 h.*

(6) To investigate the effect of low catalytic loading on the decarbonylative coupling, the cross-coupling was conducted at 0.25 mol% of Pd$(OAc)_2$ (Scheme S6). Formation of product **3b** from **1b** was observed without a noticeable decrease in yield, consistent with the high efficiency of this cross-coupling. The high catalytic efficiency is characteristic to Pd catalysis and bodes well for the future development of protocols in the redox-neutral decarbonylative cross-coupling manifold of carboxylic acids.

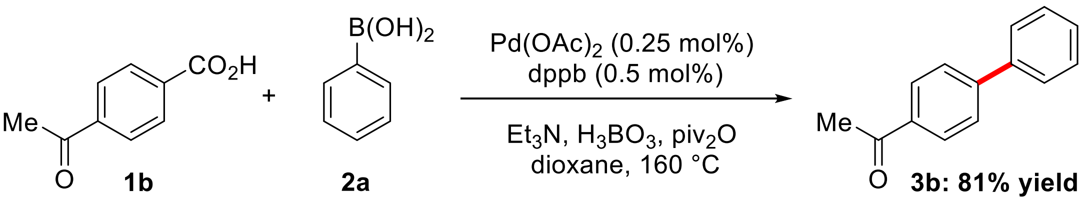

Scheme S6. Decarbonylative Suzuki-Miyaura coupling of carboxylic acids at low catalyst loading, related to Figure 1. *Conditions:* ***1*** *(1.0 equiv), PhB*$(OH)_2$ *(2.0 equiv), Pd*$(OAc)_2$ *(0.25 mol%), dppb (0.5 mol%),* Et_3N *(1.5 equiv),* H_3BO_3 *(1.5 equiv), piv*$_2$*O (1.5 equiv), dioxane, 160 °C, 15 h.*

Characterization Data of Cross-Coupling Products

4-(Methoxycarbonyl)benzoic acid and phenylboronic acid (3a, Figure 3, Entry 1)

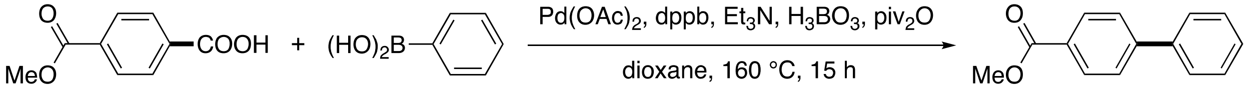

1a **2a** **3a**

According to the general procedure, the reaction of 4-(methoxycarbonyl)benzoic acid (0.20 mmol), phenylboronic acid (2.0 equiv), $Pd(OAc)_2$ (5 mol%), 1,4-bis(diphenylphosphino)butane (10 mol%), triethylamine (1.5 equiv), H_3BO_3 (1.5 equiv) and trimethylacetic anhydride (1.5 equiv) in 1,4-dioxane (0.20 M) for 15 h at 160 °C, afforded after work-up and chromatography the title compound in 92% yield (39.1 mg). White solid. **^{1}H NMR (500 MHz, $CDCl_3$)** δ 8.15-8.13 (d, *J* = 8.3 Hz, 2 H), 7.70-7.68 (d, *J* = 8.3 Hz, 2 H), 7.66-7.65 (d, *J* = 7.6 Hz, 2 H), 7.51-7.48 (t, *J* = 7.4 Hz, 2 H), 7.44-7.41 (t, *J* = 7.4 Hz, 1 H), 3.97 (s, 3 H). **^{13}C NMR (125 MHz, $CDCl_3$)** δ 167.02, 145.65, 140.02, 130.13, 128.95, 128.92, 128.17, 127.30, 127.07, 52.15. The spectral data matched those reported in the literature (Liu et al., 2018).

4-Acetylbenzoic acid and phenylboronic acid (3b, Figure 3, Entry 2)

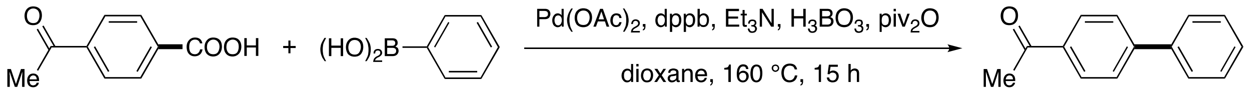

1b **2a** **3b**

According to the general procedure, the reaction of 4-acetylbenzoic acid (0.20 mmol), phenylboronic acid (2.0 equiv), $Pd(OAc)_2$ (5 mol%), 1,4-bis(diphenylphosphino)butane (10 mol%), triethylamine (1.5 equiv), H_3BO_3 (1.5 equiv) and trimethylacetic anhydride (1.5 equiv) in 1,4-dioxane (0.20 M) for 15 h at 160 °C, afforded after work-up and chromatography the title compound in 83% yield (32.6 mg). White solid. **^{1}H NMR (500 MHz, $CDCl_3$)** δ 8.07-8.05 (d, *J* = 8.2 Hz, 2 H), 7.72-7.71 (d, *J* = 8.3 Hz, 2 H), 7.66-7.65 (d, *J* = 7.8 Hz, 2 H), 7.52-7.49 (t, *J* = 7.4 Hz, 2 H), 7.44-7.42 (t, *J* = 7.4 Hz, 1 H), 2.67 (s, 3 H). **^{13}C NMR (125 MHz, $CDCl_3$)** δ 197.79, 145.81, 139.90, 135.87, 128.98, 128.94, 128.26, 127.30, 127.25, 26.69. The spectral data matched those reported in the literature (Shi et al., 2016).

4-Formylbenzoic acid and phenylboronic acid (3c, Figure 3, Entry 3)

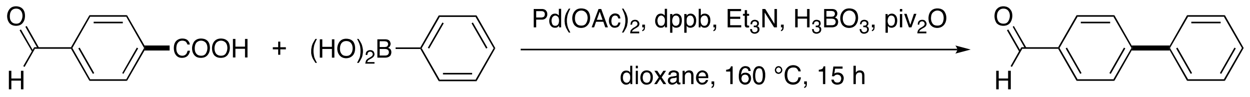

1c **2a** **3c**

According to the general procedure, the reaction of 4-formylbenzoic acid (0.20 mmol), phenylboronic acid (2.0 equiv), $Pd(OAc)_2$ (5 mol%), 1,4-bis(diphenylphosphino)butane (10 mol%), triethylamine (1.5 equiv), H_3BO_3 (1.5 equiv) and trimethylacetic anhydride (1.5 equiv) in 1,4-dioxane (0.20 M) for 15 h at 160 °C, afforded after work-up and chromatography the title compound in 82% yield (29.9 mg). White solid. **^{1}H NMR (500 MHz, $CDCl_3$)** δ 10.09 (s, 1 H), 7.99-7.98 (d, *J* = 8.2 Hz, 2 H), 7.79-7.78 (d, *J* = 8.1 Hz, 2 H), 7.67-7.66 (d, *J* = 7.4 Hz, 2 H), 7.53-7.50 (t, *J* = 7.4 Hz, 2 H), 7.46-7.43 (t, *J* = 7.2 Hz, 1 H). **^{13}C NMR (125 MHz, $CDCl_3$)** δ 191.98, 147.24, 139.75, 135.22, 130.30, 129.04, 128.50, 127.72, 127.39. The spectral data matched those reported in the literature (Kadam et al., 2018).

4-(Trifluoromethyl)benzoic acid and phenylboronic acid (3d, Figure 3, Entry 4)

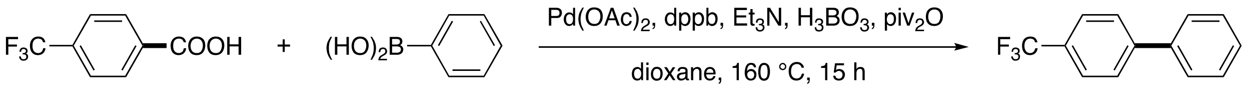

1d **2a** **3d**

According to the general procedure, the reaction of 4-(trifluoromethyl)benzoic acid (0.20 mmol), phenylboronic acid (2.0 equiv), $Pd(OAc)_2$ (5 mol%), 1,4-bis(diphenylphosphino)butane (10 mol%), triethylamine (1.5 equiv), H_3BO_3 (1.5 equiv) and trimethylacetic anhydride (1.5 equiv) in 1,4-dioxane (0.20 M) for 15 h at 160 °C, afforded after work-up and chromatography the title compound in 91% yield (40.5 mg). White solid. **^{1}H NMR (500 MHz, $CDCl_3$)** δ 7.73 (s, 4 H), 7.64-7.63 (d, *J* = 7.6 Hz, 2 H), 7.52-7.49 (t, *J* = 7.5 Hz, 2 H), 7.46-7.43 (t, *J* = 7.4 Hz, 1 H). **^{13}C NMR (125 MHz, $CDCl_3$)** δ 144.75, 139.79, 129.36 (q, J^F = 32.3 Hz), 129.01, 128.21, 127.44, 127.30, 125.73 (q, J^F = 3.7 Hz), 124.34 (q, J^F = 270.2 Hz). **^{19}F NMR (471 MHz, $CDCl_3$)** δ -62.39. The spectral data matched those reported in the literature (Shi et al., 2016).

4-(Tosyloxy)benzoic acid and phenylboronic acid (3e, Figure 3, Entry 5)

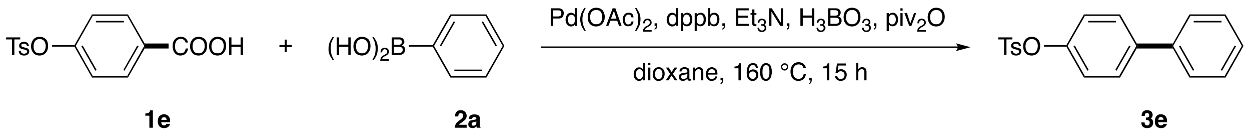

According to the general procedure, the reaction of 4-(tosyloxy)benzoic acid (0.20 mmol), phenylboronic acid (2.0 equiv), $Pd(OAc)_2$ (5 mol%), 1,4-bis(diphenylphosphino)butane (10 mol%), triethylamine (1.5 equiv), H_3BO_3 (1.5 equiv) and trimethylacetic anhydride (1.5 equiv) in 1,4-dioxane (0.20 M) for 15 h at 160 °C, afforded after work-up and chromatography the title compound in 94% yield (61.0 mg). White solid. **^{1}H NMR (500 MHz, $CDCl_3$)** δ 7.79-7.76 (t, *J* = 6.9 Hz, 4 H), 7.55-7.50 (m, 3 H), 7.47-7.44 (t, *J* = 7.5 Hz, 1 H), 7.39-7.34 (m, 3 H), 7.15-7.13 (d, *J* = 8.4 Hz, 1 H), 7.08-7.06 (d, *J* = 8.4 Hz, 1 H), 2.48 (s, 3 H). **^{13}C NMR (125 MHz, $CDCl_3$)** δ 149.02, 140.18, 137.13, 132.74, 131.73, 129.96, 129.79, 128.87, 128.58, 128.25, 127.08, 122.67, 21.74. The spectral data matched those reported in the literature (Lv et al., 2018).

4-Cyanobenzoic acid and phenylboronic acid (3f, Figure 3, Entry 6)

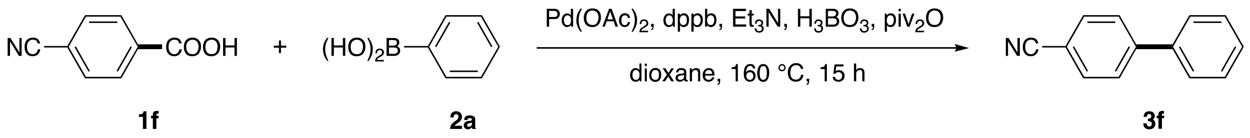

According to the general procedure, the reaction of 4-cyanobenzoic acid (0.20 mmol), phenylboronic acid (2.0 equiv), $Pd(OAc)_2$ (5 mol%), 1,4-bis(diphenylphosphino)butane (10 mol%), triethylamine (1.5 equiv), H_3BO_3 (1.5 equiv) and trimethylacetic anhydride (1.5 equiv) in 1,4-dioxane (0.20 M) for 15 h at 160 °C, afforded after work-up and chromatography the title compound in 62% yield (22.3 mg). White solid. **^{1}H NMR (500 MHz, $CDCl_3$)** δ 7.76-7.70 (m, 4 H), 7.62-7.61 (d, *J* = 7.4 Hz, 2 H), 7.53-7.50 (t, *J* = 7.3 Hz, 2 H), 7.47-7.44 (t, *J* = 7.2 Hz, 1 H). **^{13}C NMR (125 MHz, $CDCl_3$)** δ 145.70, 139.20, 132.62, 129.13, 128.68, 127.76, 127.25, 118.97, 110.94. The spectral data matched those reported in the literature (Gan et al., 2018).

2-Methylbenzoic acid and 4-methoxyphenylboronic acid (3g, Figure 3, Entry 7)

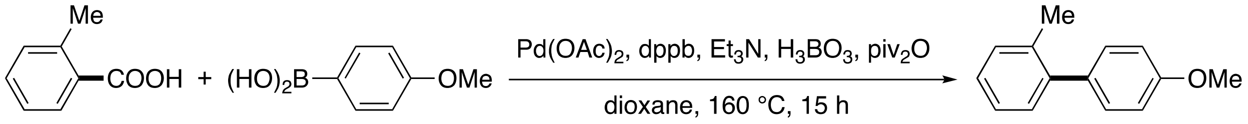

1g **2b** **3g**

According to the general procedure, the reaction of 2-methylbenzoic acid (0.20 mmol), 4-methoxyphenylboronic acid (2.0 equiv), $Pd(OAc)_2$ (5 mol%), 1,4-bis(diphenylphosphino)butane (10 mol%), triethylamine (1.5 equiv), H_3BO_3 (1.5 equiv) and trimethylacetic anhydride (1.5 equiv) in 1,4-dioxane (0.20 M) for 15 h at 160 °C, afforded after work-up and chromatography the title compound in 80% yield (31.8 mg). White solid. **^{1}H NMR (500 MHz, $CDCl_3$)** δ 7.30-7.26 (m, 6 H), 7.00-6.98 (d, J = 8.6 Hz, 2 H), 3.89 (s, 3 H), 2.31 (s, 3 H). **^{13}C NMR (125 MHz, $CDCl_3$)** δ 158.52, 141.56, 135.51, 134.39, 130.31, 130.27, 129.92, 126.99, 125.77, 113.50, 55.30, 20.56. The spectral data matched those reported in the literature (Simpson et al., 2018).

2-(Methylthio)benzoic acid and 4-methoxyphenylboronic acid (3h, Figure 3, Entry 8)

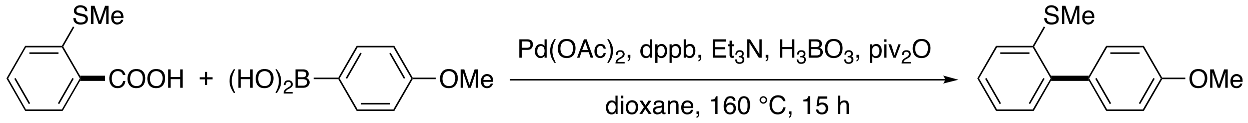

1h **2b** **3h**

According to the general procedure, the reaction of 2-(methylthio)benzoic acid (0.20 mmol), 4-methoxyphenylboronic acid (2.0 equiv), $Pd(OAc)_2$ (5 mol%), 1,4-bis(diphenylphosphino)butane (10 mol%), triethylamine (1.5 equiv), H_3BO_3 (1.5 equiv) and trimethylacetic anhydride (1.5 equiv) in 1,4-dioxane (0.20 M) for 15 h at 160 °C, afforded after work-up and chromatography the title compound in 97% yield (44.7 mg). White solid. **^{1}H NMR (500 MHz, $CDCl_3$)** δ 7.39-7.33 (m, 3 H), 7.30-7.29 (d, J = 9.5 Hz, 1 H), 7.24-7.20 (m, 2 H), 7.01-6.99 (d, J = 8.6 Hz, 2 H), 3.89 (s, 3 H), 2.40 (s, 3 H). **^{13}C NMR (125 MHz, $CDCl_3$)** δ 159.06, 140.56, 137.30, 132.93, 130.47, 130.07, 127.69, 125.09, 124.69, 113.54, 55.28, 16.00. The spectral data matched those reported in the literature (Sugahara et al., 2014).

2-Methoxybenzoic acid and phenylboronic acid (3i, Figure 3, Entry 9)

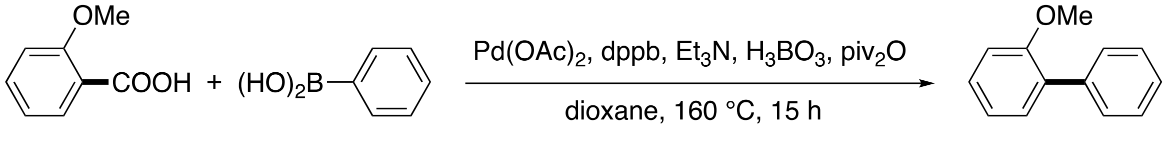

According to the general procedure, the reaction of 2-methoxybenzoic acid (0.20 mmol), phenylboronic acid (2.0 equiv), $Pd(OAc)_2$ (5 mol%), 1,4-bis(diphenylphosphino)butane (10 mol%), triethylamine (1.5 equiv), H_3BO_3 (1.5 equiv) and trimethylacetic anhydride (1.5 equiv) in 1,4-dioxane (0.20 M) for 15 h at 160 °C, afforded after work-up and chromatography the title compound in 55% yield (20.3 mg). White solid. **^{1}H NMR (500 MHz, $CDCl_3$)** δ 7.58-7.57 (d, *J* = 7.6 Hz, 2 H), 7.47-7.44 (t, *J* = 7.5 Hz, 2 H), 7.38-7.35 (t, *J* = 7.1 Hz, 3 H), 7.09-7.06 (t, *J* = 7.5 Hz, 1 H), 7.04-7.02 (d, *J* = 8.6 Hz, 1 H), 3.85 (s, 3 H). **^{13}C NMR (125 MHz, $CDCl_3$)** δ 156.49, 138.57, 130.92, 130.75, 129.57, 128.64, 128.01, 126.94, 120.85, 111.25, 55.58. The spectral data matched those reported in the literature (Simpson et al., 2018).

1-Naphthoic acid and phenylboronic acid (3j, Figure 3, Entry 10)

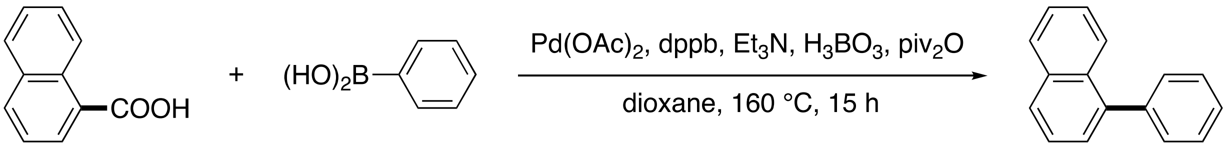

According to the general procedure, the reaction of 1-naphthoic acid (0.20 mmol), phenylboronic acid (2.0 equiv), $Pd(OAc)_2$ (5 mol%), 1,4-bis(diphenylphosphino)butane (10 mol%), triethylamine (1.5 equiv), H_3BO_3 (1.5 equiv) and trimethylacetic anhydride (1.5 equiv) in 1,4-dioxane (0.20 M) for 15 h at 160 °C, afforded after work-up and chromatography the title compound in 77% yield (31.5 mg). White solid. **^{1}H NMR (500 MHz, $CDCl_3$)** δ 7.95-7.94 (d, *J* = 8.6 Hz, 2 H), 7.91-7.89 (d, *J* = 8.2 Hz, 1 H), 7.58-7.51 (m, 6 H), 7.48-7.45 (m, 3 H). **^{13}C NMR (125 MHz, $CDCl_3$)** δ 140.79, 140.29, 133.82, 131.64, 130.10, 128.28, 127.65, 127.26, 126.95, 126.05, 126.04, 125.79, 125.40. The spectral data matched those reported in the literature (Shi et al., 2016).

2-Naphthoic acid and 4-methoxyphenylboronic acid (3k, Figure 3, Entry 11)

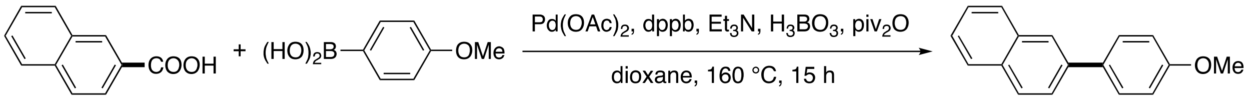

1k **2b** **3k**

According to the general procedure, the reaction of 2-naphthoic acid (0.20 mmol), 4-methoxyphenylboronic acid (2.0 equiv), $Pd(OAc)_2$ (5 mol%), 1,4-bis(diphenylphosphino)butane (10 mol%), triethylamine (1.5 equiv), H_3BO_3 (1.5 equiv) and trimethylacetic anhydride (1.5 equiv) in 1,4-dioxane (0.20 M) for 15 h at 160 °C, afforded after work-up and chromatography the title compound in 60% yield (28.2 mg). White solid. **^{1}H NMR (500 MHz, $CDCl_3$)** δ 8.02 (s, 1 H), 7.93-7.87 (m, 3 H), 7.76-7.74 (dd, *J* = 8.5 Hz, 1 H), 7.70-7.69 (d, *J* = 8.7 Hz, 2 H), 7.54-7.48 (m, 2 H), 7.07-7.05 (d, *J* = 8.7 Hz, 2 H), 3.91 (s, 3 H). **^{13}C NMR (125 MHz, $CDCl_3$)** δ 159.27, 138.17, 133.77, 133.66, 132.33, 128.45, 128.36, 128.07, 127.64, 126.25, 125.66, 125.46, 125.05, 114.34, 55.41. The spectral data matched those reported in the literature (Muto et al., 2015).

Quinoline-6-carboxylic acid and phenylboronic acid (3l, Figure 3, Entry 12)

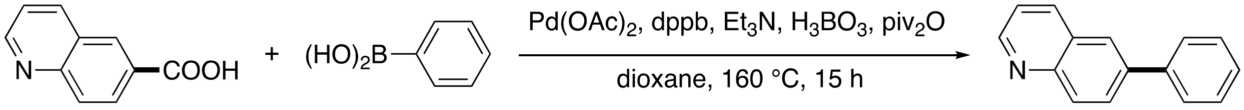

1l **2a** **3l**

According to the general procedure, the reaction of quinoline-6-carboxylic acid (0.20 mmol), phenylboronic acid (2.0 equiv), $Pd(OAc)_2$ (5 mol%), 1,4-bis(diphenylphosphino)butane (10 mol%), triethylamine (1.5 equiv), H_3BO_3 (1.5 equiv) and trimethylacetic anhydride (1.5 equiv) in 1,4-dioxane (0.20 M) for 15 h at 160 °C, afforded after work-up and chromatography the title compound in 76% yield (31.2 mg). White solid. **^{1}H NMR (500 MHz, $CDCl_3$)** δ 8.95-8.95 (d, *J* = 2.7 Hz, 1 H), 8.25-8.20 (m, 2 H), 8.03-8.01 (m, 2 H), 7.76-7.74 (d, *J* = 7.5 Hz, 2 H), 7.55-7.52 (t, *J* = 7.5 Hz, 2 H), 7.47-7.42 (m, 2 H). **^{13}C NMR (125 MHz, $CDCl_3$)** δ 150.40, 147.70, 140.35, 139.37, 136.28, 129.92, 129.27, 128.99, 128.49, 127.78, 127.49, 125.51, 121.50. The spectral data matched those reported in the literature (Okura et al., 2018).

Nicotinic acid and phenylboronic acid (3m, Figure 3, Entry 13)

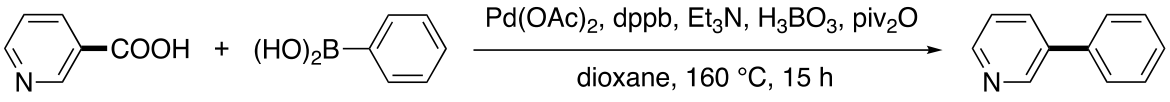

1m **2a** **3m**

According to the general procedure, the reaction of nicotinic acid (0.20 mmol), phenylboronic acid (2.0 equiv), $Pd(OAc)_2$ (5 mol%), 1,4-bis(diphenylphosphino)butane (10 mol%), triethylamine (1.5 equiv), H_3BO_3 (1.5 equiv) and trimethylacetic anhydride (1.5 equiv) in 1,4-dioxane (0.20 M) for 15 h at 160 °C, afforded after work-up and chromatography the title compound in 92% yield (28.6 mg). White solid. **^{1}H NMR (500 MHz, $CDCl_3$)** δ 8.88 (s, 1 H), 8.63-8.62 (d, *J* = 4.5 Hz, 1 H), 7.91-7.90 (d, *J* = 7.9 Hz, 1 H), 7.62-7.61 (d, *J* = 7.5 Hz, 2 H), 7.53-7.50 (t, *J* = 7.5 Hz, 2 H), 7.45-7.42 (t, *J* = 7.4 Hz, 1 H), 7.41-7.38 (m, 1 H). **^{13}C NMR (125 MHz, $CDCl_3$)** δ 148.49, 148.37, 137.87, 136.67, 134.39, 129.10, 128.12, 127.18, 123.56. The spectral data matched those reported in the literature (Muto et al., 2015).

Picolinic acid and 4-methoxyphenylboronic acid (3n, Figure 3, Entry 14)

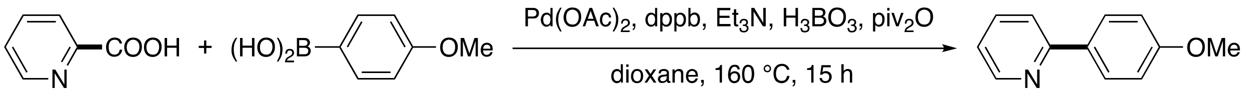

1n **2b** **3n**

According to the general procedure, the reaction of picolinic acid (0.20 mmol), 4-methoxyphenylboronic acid (2.0 equiv), $Pd(OAc)_2$ (5 mol%), 1,4-bis(diphenylphosphino)butane (10 mol%), triethylamine (1.5 equiv), H_3BO_3 (1.5 equiv) and trimethylacetic anhydride (1.5 equiv) in 1,4-dioxane (0.20 M) for 15 h at 160 °C, afforded after work-up and chromatography the title compound in 75% yield (27.8 mg). White solid. **^{1}H NMR (500 MHz, $CDCl_3$)** δ 8.68-8.67 (d, *J* = 4.5 Hz, 1 H), 7.99-7.97 (d, *J* = 8.7 Hz, 2 H), 7.75-7.72 (t, *J* = 7.8 Hz, 1 H), 7.70-7.68 (m, 1 H), 7.21-7.18 (t, *J* = 6.0 Hz, 1 H), 7.03-7.02 (d, *J* = 8.7 Hz, 2 H), 3.89 (s, 3 H). **^{13}C NMR (125 MHz, $CDCl_3$)** δ 160.47, 157.15, 149.56, 136.68, 132.06, 128.18, 121.42, 119.83, 114.13, 55.37. The spectral data matched those reported in the literature (Muto et al., 2015).

4-(Dibenzo[*b,d*]furan-4-yl)benzoic acid and 4-methoxyphenylboronic acid (3o, Figure 3, Entry 15)

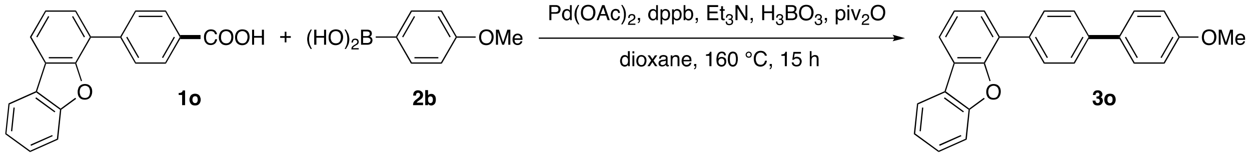

According to the general procedure, the reaction of 4-(dibenzo[*b,d*]furan-4-yl)benzoic acid (0.20 mmol), 4-methoxyphenylboronic acid (2.0 equiv), $Pd(OAc)_2$ (5 mol%), 1,4-bis(diphenylphosphino)butane (10 mol%), triethylamine (1.5 equiv), H_3BO_3 (1.5 equiv) and trimethylacetic anhydride (1.5 equiv) in 1,4-dioxane (0.20 M) for 15 h at 160 °C, afforded after work-up and chromatography the title compound in 78% yield (54.7 mg). *New compound.* White solid. **Mp** = 189-191 °C. **^{1}H NMR (500 MHz, $CDCl_3$)** δ 8.04-8.00 (t, *J* = 7.9 Hz, 3 H), 7.98-7.97 (d, *J* = 7.5 Hz, 1 H), 7.77-7.75 (d, *J* = 8.2 Hz, 2 H), 7.69-7.64 (m, 4 H), 7.52-7.46 (m, 2 H), 7.41-7.38 (t, *J* = 7.4 Hz, 1 H), 7.06-7.04 (d, *J* = 8.6 Hz, 2 H), 3.90 (s, 3 H). **^{13}C NMR (125 MHz, $CDCl_3$)** δ 159.31, 156.22, 153.44, 140.25, 134.77, 133.35, 129.14, 128.16, 127.25, 126.96, 126.69, 125.56, 124.97, 124.24, 123.26, 122.80, 120.70, 119.63, 114.32, 111.89, 55.39. **HRMS** calcd for $C_{25}H_{18}O_2Na$ (M^+ + Na) 350.1301, found 350.1314.

4-(Dibenzo[*b,d*]thiophen-4-yl)benzoic acid and 4-methoxyphenylboronic acid (3p, Figure 3, Entry 16)

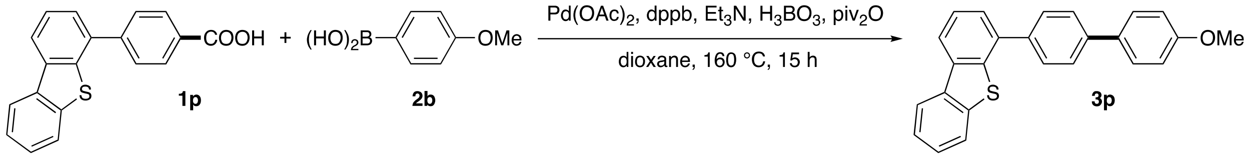

According to the general procedure, the reaction of 4-(dibenzo[*b,d*]thiophen-4-yl)benzoic acid (0.20 mmol), 4-methoxyphenylboronic acid (2.0 equiv), $Pd(OAc)_2$ (5 mol%), 1,4-bis(diphenylphosphino)butane (10 mol%), triethylamine (1.5 equiv), H_3BO_3 (1.5 equiv) and trimethylacetic anhydride (1.5 equiv) in 1,4-dioxane (0.20 M) for 15 h at 160 °C, afforded after work-up and chromatography the title compound in 73% yield (53.5 mg). *New compound.* White solid. **Mp** = 198-200 °C. **^{1}H NMR (500 MHz, $CDCl_3$)** δ 8.24-8.19 (m, 2 H), 7.89-7.87 (m, 1 H), 7.84-7.83 (d, *J* = 8.1 Hz, 2 H), 7.75-7.73 (d, *J* = 8.2 Hz, 2 H), 7.66-7.65 (d, *J* = 8.7 Hz, 2 H), 7.62-7.59 (t, *J* = 7.5 Hz, 1 H), 7.57-7.55 (m, 1 H), 7.52-7.48 (m, 2 H), 7.06-7.04 (d, *J* = 8.6 Hz, 2 H), 3.91 (s, 3 H). **^{13}C NMR (125 MHz, $CDCl_3$)** δ 159.38, 140.48, 139.63, 138.93, 138.59,

136.73, 136.30, 135.84, 133.22, 128.63, 128.16, 127.05, 126.83, 126.81, 125.14, 124.39, 122.63, 121.75, 120.43, 114.34, 55.38. **HRMS** calcd for $C_{25}H_{18}OSNa$ (M^+ + Na) 366.1073, found 366.1065.

4'-(Trifluoromethyl)-[1,1'-biphenyl]-4-carboxylic acid and phenylboronic acid (3q, Figure 3, Entry 17)

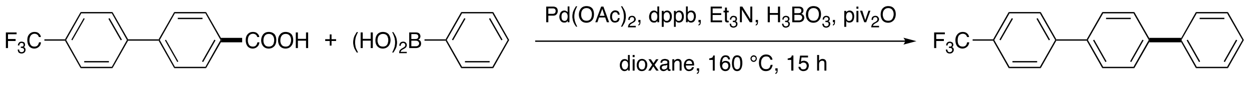

1q 2a 3q

According to the general procedure, the reaction of 4'-(trifluoromethyl)-[1,1'-biphenyl]-4-carboxylic acid (0.20 mmol), phenylboronic acid (2.0 equiv), $Pd(OAc)_2$ (5 mol%), 1,4-bis(diphenylphosphino)butane (10 mol%), triethylamine (1.5 equiv), H_3BO_3 (1.5 equiv) and trimethylacetic anhydride (1.5 equiv) in 1,4-dioxane (0.20 M) for 15 h at 160 °C, afforded after work-up and chromatography the title compound in 78% yield (46.6 mg). White solid. **^{1}H NMR (500 MHz, CDCl$_3$)** δ 7.78-7.70 (m, 8 H), 7.68-7.66 (d, J = 7.8 Hz, 2 H), 7.51-7.48 (t, J = 7.5 Hz, 2 H), 7.42-7.39 (t, J = 7.4 Hz, 1 H). **^{13}C NMR (125 MHz, CDCl$_3$)** δ 144.23, 141.12, 140.41, 138.60, 129.05 (q, J^F = 56.8 Hz), 128.90, 127.72, 127.65, 127.61, 127.51 (q, J^F = 220.7 Hz), 127.29, 127.09, 125.78 (q, J^F = 4.4 Hz). **^{19}F NMR (471 MHz, CDCl$_3$)** δ -62.40. The spectral data matched those reported in the literature (Nakamura et al., 2015).

(*E*)-2-Styrylbenzoic acid and 4-methoxyphenylboronic acid (3r, Figure 3, Entry 18)

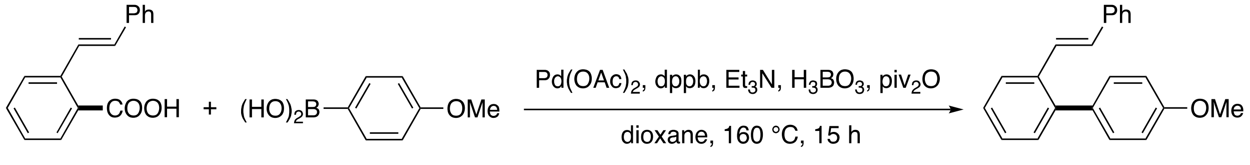

1r 2b 3r

According to the general procedure, the reaction of (*E*)-2-styrylbenzoic acid (0.20 mmol), 4-methoxyphenylboronic acid (2.0 equiv), $Pd(OAc)_2$ (5 mol%), 1,4-bis(diphenylphosphino)butane (10 mol%), triethylamine (1.5 equiv), H_3BO_3 (1.5 equiv) and trimethylacetic anhydride (1.5 equiv) in 1,4-dioxane (0.20 M) for 15 h at 160 °C, afforded after work-up and chromatography the title compound in 82% yield (47.0 mg). White solid. **^{1}H NMR (500 MHz, CDCl$_3$)** δ 7.78-7.76 (d, J = 7.4 Hz, 1 H), 7.43-7.33 (m, 9 H), 7.27-7.24 (t, J = 7.4 Hz, 1 H), 7.19-7.16 (d, J = 16.3 Hz, 1 H), 7.09-7.05 (d, J = 16.3 Hz, 1 H), 7.02-7.00 (d, J = 8.6 Hz, 2 H), 3.91 (s, 3 H). **^{13}C**

NMR (125 MHz, CDCl3) δ 158.85, 140.82, 137.70, 135.48, 133.29, 131.02, 130.29, 129.27, 128.65, 128.07, 127.55, 127.45, 127.25, 126.55, 125.91, 113.59, 55.34. The spectral data matched those reported in the literature (Veld et al., 1978).

[1,1'-Biphenyl]-2-carboxylic acid and 4-methoxyphenylboronic acid (3s, Figure 3, Entry 19)

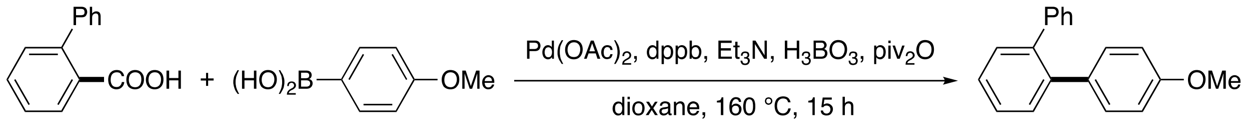

According to the general procedure, the reaction of [1,1'-biphenyl]-2-carboxylic acid (0.20 mmol), 4-methoxyphenylboronic acid (2.0 equiv), $Pd(OAc)_2$ (5 mol%), 1,4-bis(diphenylphosphino)butane (10 mol%), triethylamine (1.5 equiv), H_3BO_3 (1.5 equiv) and trimethylacetic anhydride (1.5 equiv) in 1,4-dioxane (0.20 M) for 15 h at 160 °C, afforded after work-up and chromatography the title compound in 56% yield (29.2 mg). White solid. **^{1}H NMR (500 MHz, CDCl3)** δ 7.45-7.43 (m, 4 H), 7.27-7.21 (m, 3 H), 7.19-7.18 (m, 2 H), 7.09-7.08 (d, *J* = 8.6 Hz, 2 H), 6.79-6.78 (d, *J* = 8.6 Hz, 2 H), 3.80 (s, 3 H). **^{13}C NMR (125 MHz, CDCl3)** δ 158.30, 141.74, 140.49, 140.16, 133.91, 130.94, 130.64, 130.55, 129.89, 127.91, 127.48, 127.14, 126.38, 113.35, 55.18. The spectral data matched those reported in the literature (Yadav et al., 2017).

Benzoic acid and 4-methoxyphenylboronic acid (3t, Figure 3, Entry 20)

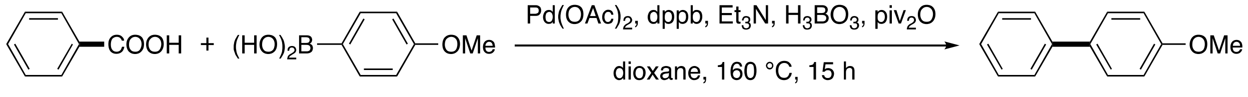

According to the general procedure, the reaction of benzoic acid (0.20 mmol), 4-methoxyphenylboronic acid (2.0 equiv), $Pd(OAc)_2$ (5 mol%), 1,4-bis(diphenylphosphino)butane (10 mol%), triethylamine (1.5 equiv), H_3BO_3 (1.5 equiv) and trimethylacetic anhydride (1.5 equiv) in 1,4-dioxane (0.20 M) for 15 h at 160 °C, afforded after work-up and chromatography the title compound in 51% yield (18.8 mg). White solid. **^{1}H NMR (500 MHz, CDCl3)** δ 7.59-7.55 (m, 4 H), 7.46-7.43 (t, *J* = 7.6 Hz, 2 H), 7.34-7.31 (t, *J* = 7.4 Hz, 1 H), 7.02-7.00 (d, *J* = 8.7 Hz, 2 H), 3.88 (s, 3 H). **^{13}C NMR (125 MHz, CDCl3)** δ 159.15, 140.85, 133.80, 128.73, 128.17,

126.76, 126.67, 114.21, 55.37. The spectral data matched those reported in the literature (Song et al., 2018).

4-Chlorobenzoic acid and 4-methoxyphenylboronic acid (3u, Figure 3, Entry 21)

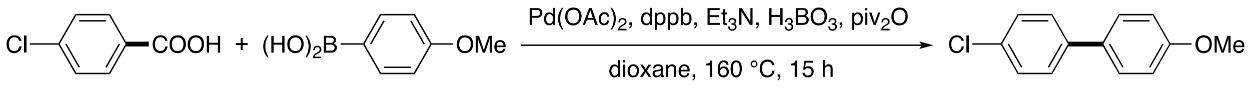

1u **2b** **3u**

According to the general procedure, the reaction of 4-chlorobenzoic acid (0.20 mmol), 4-methoxyphenylboronic acid (2.0 equiv), $Pd(OAc)_2$ (5 mol%), 1,4-bis(diphenylphosphino)butane (10 mol%), triethylamine (1.5 equiv), H_3BO_3 (1.5 equiv) and trimethylacetic anhydride (1.5 equiv) in 1,4-dioxane (0.20 M) for 15 h at 160 °C, afforded after work-up and chromatography the title compound in 59% yield (25.8 mg). White solid. **^{1}H NMR (500 MHz, $CDCl_3$)** δ 7.52-7.49 (t, *J* = 8.3 Hz, 4 H), 7.41-7.39 (d, *J* = 8.5 Hz, 2 H), 7.01-6.99 (d, *J* = 8.7 Hz, 2 H), 3.88 (s, 3 H). **^{13}C NMR (125 MHz, $CDCl_3$)** δ 159.38, 139.29, 132.69, 132.52, 128.85, 128.03, 127.95, 114.33, 55.39. The spectral data matched those reported in the literature (Keaveney et al., 2018).

3-(Methoxycarbonyl)benzoic acid and phenylboronic acid (3v, Figure 3, Entry 22)

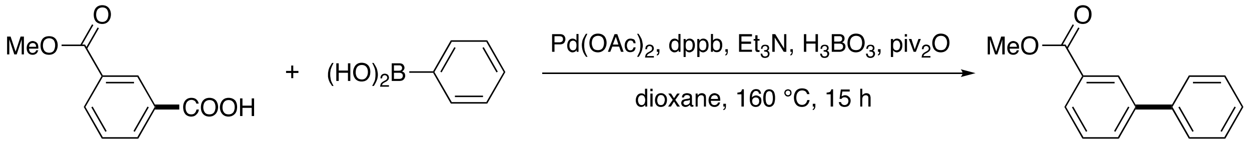

1v **2a** **3v**

According to the general procedure, the reaction of 3-(methoxycarbonyl)benzoic acid (0.20 mmol), phenylboronic acid (2.0 equiv), $Pd(OAc)_2$ (5 mol%), 1,4-bis(diphenylphosphino)butane (10 mol%), triethylamine (1.5 equiv), H_3BO_3 (1.5 equiv) and trimethylacetic anhydride (1.5 equiv) in 1,4-dioxane (0.20 M) for 15 h at 160 °C, afforded after work-up and chromatography the title compound in 52% yield (22.1 mg). White solid. **^{1}H NMR (500 MHz, $CDCl_3$)** δ 8.31 (s, 1 H), 8.06-8.04 (d, *J* = 7.8 Hz, 1 H), 7.82-7.81 (d, *J* = 7.7 Hz, 1 H), 7.66-7.65 (d, *J* = 7.5 Hz, 2 H), 7.56-7.53 (t, *J* = 7.7 Hz, 1 H), 7.51-7.48 (t, *J* = 7.7 Hz, 2 H), 7.42-7.39 (t, *J* = 7.4 Hz, 1 H), 3.98 (s, 3 H). **^{13}C NMR (125 MHz, $CDCl_3$)** δ 167.09, 141.50, 140.14, 131.56, 130.71, 128.91, 128.88, 128.37, 128.30, 127.77, 127.19, 52.23. The spectral data matched those reported in the literature (Yadav et al., 2017).

3-Acetylbenzoic acid and phenylboronic acid (3w, Figure 3, Entry 23)

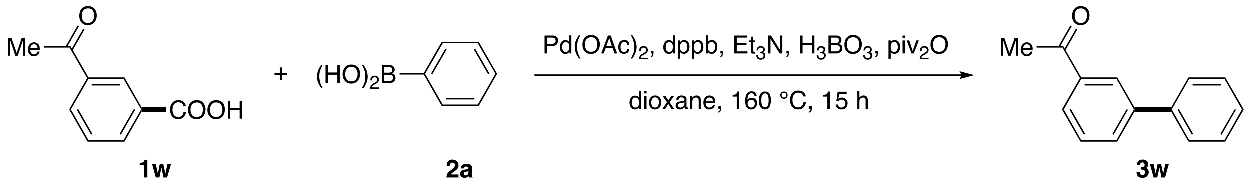

According to the general procedure, the reaction of 3-acetylbenzoic acid (0.20 mmol), phenylboronic acid (2.0 equiv), $Pd(OAc)_2$ (5 mol%), 1,4-bis(diphenylphosphino)butane (10 mol%), triethylamine (1.5 equiv), H_3BO_3 (1.5 equiv) and trimethylacetic anhydride (1.5 equiv) in 1,4-dioxane (0.20 M) for 15 h at 160 °C, afforded after work-up and chromatography the title compound in 68% yield (26.7 mg). White solid. **^{1}H NMR (500 MHz, $CDCl_3$)** δ 8.21 (s, 1 H), 7.97-7.96 (d, J = 7.7 Hz, 1 H), 7.83-7.81 (d, J = 7.6 Hz, 1 H), 7.66-7.64 (d, J = 7.3 Hz, 2 H), 7.58-7.55 (t, J = 7.7 Hz, 1 H), 7.51-7.48 (t, J = 7.4 Hz, 2 H), 7.43-7.40 (t, J = 7.4 Hz, 1 H), 2.69 (s, 3 H). **^{13}C NMR (125 MHz, $CDCl_3$)** δ 198.12, 141.75, 140.21, 137.66, 131.77, 129.07, 128.95, 127.84, 127.22, 127.21, 126.99, 26.79. The spectral data matched those reported in the literature (Liu et al., 2018).

3-Chlorobenzoic acid and phenylboronic acid (3x, Figure 3, Entry 24)

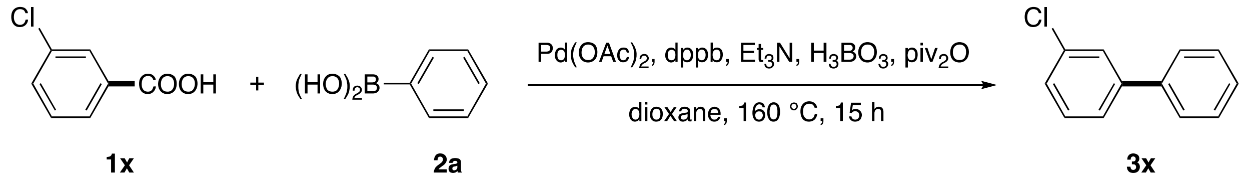

According to the general procedure, the reaction of 3-chlorobenzoic acid (0.20 mmol), phenylboronic acid (2.0 equiv), $Pd(OAc)_2$ (5 mol%), 1,4-bis(diphenylphosphino)butane (10 mol%), triethylamine (1.5 equiv), H_3BO_3 (1.5 equiv) and trimethylacetic anhydride (1.5 equiv) in 1,4-dioxane (0.20 M) for 15 h at 160 °C, afforded after work-up and chromatography the title compound in 55% yield (20.8 mg). White solid. **^{1}H NMR (500 MHz, $CDCl_3$)** δ 7.60-7.58 (m, 3 H), 7.50-7.46 (m, 3 H), 7.41-7.38 (m, 2 H), 7.35-7.34 (m, 1 H). **^{13}C NMR (125 MHz, $CDCl_3$)** δ 143.09, 139.83, 134.66, 130.00, 128.91, 127.88, 127.32, 127.27, 127.13, 125.32. The spectral data matched those reported in the literature (Keaveney et al., 2018).

4-(Trifluoromethoxy)benzoic acid and 4-methoxyphenylboronic acid (3y, Figure 3, Entry 25)

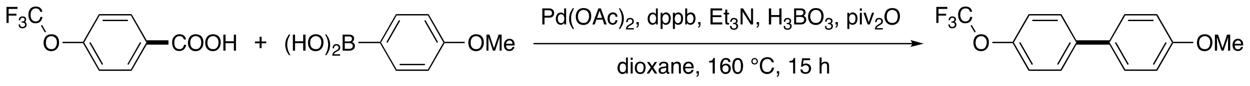

1y **2b** **3y**

According to the general procedure, the reaction of 4-(trifluoromethoxy)benzoic acid (0.20 mmol), 4-methoxyphenylboronic acid (2.0 equiv), $Pd(OAc)_2$ (5 mol%), 1,4-bis(diphenylphosphino)butane (10 mol%), triethylamine (1.5 equiv), H_3BO_3 (1.5 equiv) and trimethylacetic anhydride (1.5 equiv) in 1,4-dioxane (0.20 M) for 15 h at 160 °C, afforded after work-up and chromatography the title compound in 95% yield (51.0 mg). White solid. **^{1}H NMR (500 MHz, $CDCl_3$)** δ 7.58-7.57 (d, *J* = 8.6 Hz, 2 H), 7.53-7.51 (d, *J* = 8.6 Hz, 2 H), 7.29-7.28 (d, *J* = 8.1 Hz, 2 H), 7.02-7.00 (d, *J* = 8.6 Hz, 2 H), 3.88 (s, 3 H). **^{13}C NMR (125 MHz, $CDCl_3$)** δ 159.44, 148.20, 139.64, 132.38, 128.17, 127.97, 121.25, 120.56 (q, J^F = 255.3 Hz), 114.34, 55.37. **^{19}F NMR (471 MHz, $CDCl_3$)** δ -57.83. The spectral data matched those reported in the literature (Liu et al., 2013).

4-(Benzoyloxy)benzoic acid and 4-methoxyphenylboronic acid (3z, Figure 3, Entry 26)

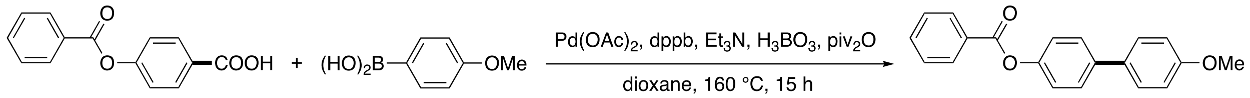

1z **2b** **3z**

According to the general procedure, the reaction of 4-(benzoyloxy)benzoic acid (0.20 mmol), 4-methoxyphenylboronic acid (2.0 equiv), $Pd(OAc)_2$ (5 mol%), 1,4-bis(diphenylphosphino)butane (10 mol%), triethylamine (1.5 equiv), H_3BO_3 (1.5 equiv) and trimethylacetic anhydride (1.5 equiv) in 1,4-dioxane (0.20 M) for 15 h at 160 °C, afforded after work-up and chromatography the title compound in 71% yield (43.3 mg). White solid. **^{1}H NMR (500 MHz, $CDCl_3$)** δ 8.26-8.25 (d, *J* = 7.3 Hz, 2 H), 7.69-7.66 (t, *J* = 7.5 Hz, 1 H), 7.63-7.61 (d, *J* = 8.6 Hz, 2 H), 7.57-7.54 (m, 4 H), 7.30-7.28 (d, *J* = 8.6 Hz, 2 H), 7.02-7.01 (d, *J* = 8.7 Hz, 2 H), 3.89 (s, 3 H). **^{13}C NMR (125 MHz, $CDCl_3$)** δ 165.31, 159.22, 149.92, 138.71, 133.63, 133.00, 130.96, 130.22, 128.60, 128.19, 127.80, 121.93, 114.27, 55.38. The compound has been previously reported (van Alpen et al., 1931).

Probenecid and phenylboronic acid (3aa, Figure 3, Entry 27)

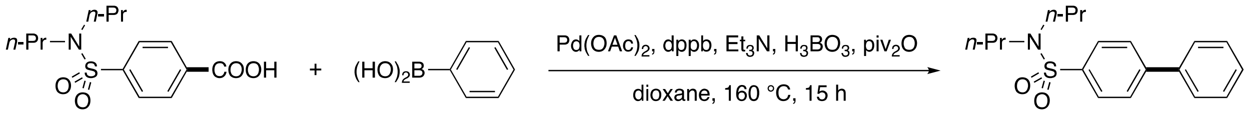

1aa **2a** **3aa**

According to the general procedure, the reaction of probenecid (0.20 mmol), phenylboronic acid (2.0 equiv), $Pd(OAc)_2$ (5 mol%), 1,4-bis(diphenylphosphino)butane (10 mol%), triethylamine (1.5 equiv), H_3BO_3 (1.5 equiv) and trimethylacetic anhydride (1.5 equiv) in 1,4-dioxane (0.20 M) for 15 h at 160 °C, afforded after work-up and chromatography the title compound in 90% yield (57.2 mg). *New compound.* White solid. **Mp** = 50-51 °C. **^{1}H NMR (500 MHz, $CDCl_3$)** δ 7.90-7.88 (d, J = 8.4 Hz, 2 H), 7.73-7.71 (d, J = 8.4 Hz, 2 H), 7.64-7.62 (d, J = 7.2 Hz, 2 H), 7.51-7.48 (t, J = 7.4 Hz, 2 H), 7.44-7.41 (t, J = 7.3 Hz, 1 H), 3.16-3.13 (t, J = 7.6 Hz, 4 H), 1.65-1.57 (m, 4 H), 0.93-0.90 (t, J = 7.4 Hz, 6 H). **^{13}C NMR (125 MHz, $CDCl_3$)** δ 145.09, 139.38, 138.77, 129.05, 128.41, 127.59, 127.58, 127.29, 50.16, 22.13, 11.24. **HRMS** calcd for $C_{18}H_{23}NO_2SNa$ (M^+ + Na) 340.1347, found 340.1383.

Flufenamic acid and 4-methoxyphenylboronic acid (3ab, Figure 3, Entry 28)

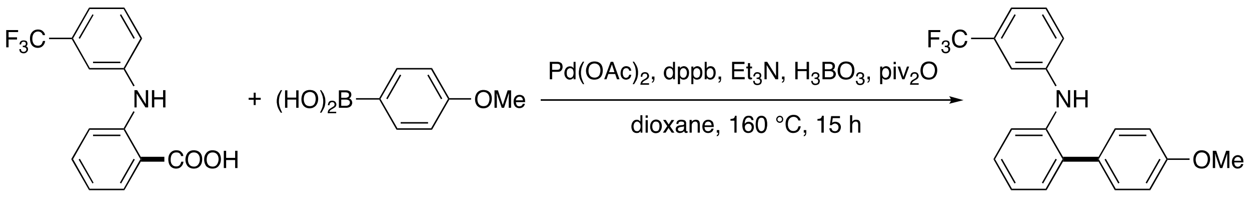

1ab **2b** **3ab**

According to the general procedure, the reaction of flufenamic acid (0.20 mmol), 4-methoxyphenylboronic acid (2.0 equiv), $Pd(OAc)_2$ (5 mol%), 1,4-bis(diphenylphosphino)butane (10 mol%), triethylamine (1.5 equiv), H_3BO_3 (1.5 equiv) and trimethylacetic anhydride (1.5 equiv) in 1,4-dioxane (0.20 M) for 15 h at 160 °C, afforded after work-up and chromatography the title compound in 60% yield (41.2 mg). *New compound.* White solid. **Mp** = 144-145 °C. **^{1}H NMR (500 MHz, $CDCl_3$)** δ 7.40-7.29 (m, 6 H), 7.24 (s, 1 H), 7.18-7.08 (m, 3 H), 7.00-6.98 (d, J = 8.7 Hz, 2 H), 5.69 (s, 1 H), 3.87 (s, 3 H). **^{13}C NMR (125 MHz, $CDCl_3$)** δ 159.17, 144.38, 138.93, 132.53, 131.77 (q, J^F = 31.8 Hz), 131.13, 130.38, 129.81, 128.07, 124.10 (q, J^F = 270.8 Hz), 122.45, 119.94, 119.07, 118.75, 116.91 (q, J^F = 3.8 Hz), 114.38, 113.42 (q, J^F = 3.9 Hz),

55.34. **^{19}F NMR (471 MHz, CDCl$_3$)** δ -62.85. **HRMS** calcd for $C_{20}H_{16}ONF_3Na$ (M^+ + Na) 343.1179, found 343.1165.

4-((*N*-(2,4-Difluorophenyl)-2-(3-(trifluoromethyl)phenoxy)nicotinamido)methyl)benzoic acid and 4-methoxyphenylboronic acid (3ac, Figure 3, Entry 29)

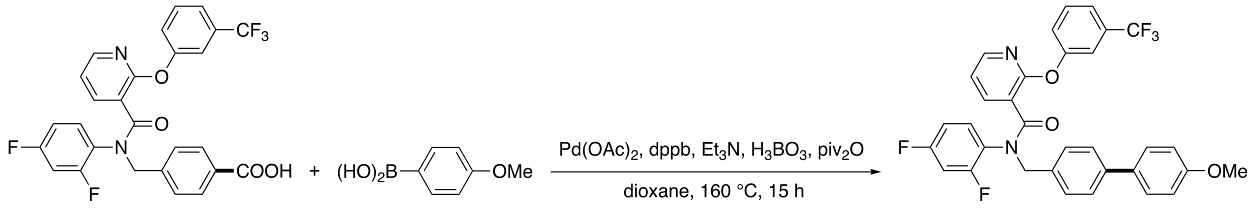

1ac 2b 3ac

According to the general procedure, the reaction of 4-((*N*-(2,4-difluorophenyl)-2-(3-(trifluoromethyl)phenoxy)nicotinamido)methyl)benzoic acid (0.20 mmol), 4-methoxyphenylboronic acid (2.0 equiv), Pd(OAc)$_2$ (5 mol%), 1,4-bis(diphenylphosphino)butane (10 mol%), triethylamine (1.5 equiv), H_3BO_3 (1.5 equiv) and trimethylacetic anhydride (1.5 equiv) in 1,4-dioxane (0.20 M) for 15 h at 160 °C, afforded after work-up and chromatography the title compound in 66% yield (78.0 mg). *New compound.* White solid. **Mp** = 300-302 °C. **^{1}H NMR (500 MHz, CDCl$_3$)** δ 8.04 (s, 1 H), 7.87-7.85 (d, *J* = 7.1 Hz, 1 H), 7.53-7.48 (m, 4 H), 7.37-7.30 (m, 4 H), 7.17-7.16 (m, 2 H), 7.05-6.99 (m, 4 H), 6.72-6.68 (t, *J* = 9.0 Hz, 1 H), 6.66-6.63 (t, *J* = 7.7 Hz, 1 H), 5.55-5.52 (d, *J* = 14.3 Hz, 1 H), 4.72-4.70 (d, *J* = 14.4 Hz, 1 H), 3.81 (s, 3 H). **^{13}C NMR (125 MHz, CDCl$_3$)** δ 167.15, 160.02 (q, J^F = 268.0 Hz), 159.92 (q, J^F = 269.5 Hz), 157.42, 156.45, 153.22, 148.44, 138.78, 138.04, 134.75, 131.91 (q, J^F = 32.6 Hz), 131.39 (d, J^F = 9.5 Hz), 130.79, 130.07, 130.01, 129.70, 128.75, 128.57, 128.19, 124.65, 121.61 (q, J^F = 3.8 Hz), 121.25, 120.88, 118.78, 118.37 (q, J^F = 3.8 Hz), 111.33, 104.85 (q, J^F = 24.2 Hz), 55.54, 52.32. **^{19}F NMR (471 MHz, CDCl$_3$)** δ -62.69, -107.80, -113.93. **HRMS** calcd for $C_{33}H_{23}N_2O_3F_5Na$ (M^+ + Na) 613.1521, found 613.1527.

4-((((*S*)-2,5,7,8-Tetramethyl-2-((4*R*,8*R*)-4,8,12-trimethyltridecyl)chroman-6-yl)oxy)methyl) benzoic acid and 4-methoxyphenylboronic acid (3ad, Figure 3, Entry 30)

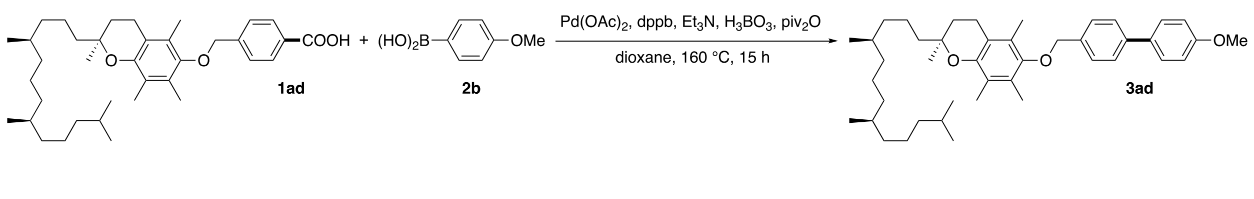

1ad 2b 3ad

According to the general procedure, the reaction of 4-((((*S*)-2,5,7,8-tetramethyl-2-((4*R*,8*R*)-4,8,12-trimethyltridecyl)chroman-6-yl)oxy)methyl)benzoic acid (0.20 mmol), 4-methoxyphenylboronic acid (2.0 equiv), $Pd(OAc)_2$ (5 mol%), 1,4-bis(diphenylphosphino)butane (10 mol%), triethylamine (1.5 equiv), H_3BO_3 (1.5 equiv) and trimethylacetic anhydride (1.5 equiv) in 1,4-dioxane (0.20 M) for 15 h at 160 °C, afforded after work-up and chromatography the title compound in 61% yield (76.5 mg). *New compound.* White solid. **Mp** = 145-147 °C. **^{1}H NMR (500 MHz, $CDCl_3$)** δ 7.62-7.57 (m, 6 H), 7.03-7.01 (d, *J* = 8.6 Hz, 2 H), 4.75 (s, 2 H), 3.89 (s, 3 H), 2.64-2.61 (t, *J* = 6.6 Hz, 2 H), 2.27 (s, 3 H), 2.22 (s, 3 H), 2.14 (s, 3 H), 1.89-1.77 (m, 2 H), 1.55-1.53 (m, 3 H), 1.45-1.38 (m, 3 H), 1.35-1.28 (m, 12 H), 1.18-1.14 (m, 3 H), 1.13-1.09 (m, 3 H), 0.90-0.87 (m, 12 H). **^{13}C NMR (125 MHz, $CDCl_3$)** δ 159.20, 148.17, 140.39, 136.47, 133.56, 128.16, 127.96, 127.75, 126.82, 125.98, 122.96, 117.62, 114.25, 114.18, 74.85, 74.49, 55.38, 40.11, 39.39, 37.48, 37.44, 37.41, 37.31, 32.82, 32.73, 31.35, 28.00, 24.83, 24.46, 23.92, 22.73, 22.64, 20.71, 19.77, 19.70, 12.91, 12.05, 11.84. **HRMS** calcd for $C_{43}H_{62}O_3Na$ (M^+ + Na) 649.4591, found 649.4616.

4-Methyl-[1,1'-biphenyl]-2-carboxylic acid and 4-methoxyphenylboronic acid (3ae, Figure 3, Entry 31)

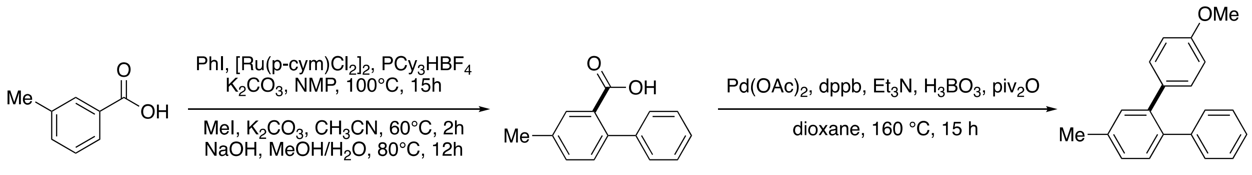

1ae 1af 3ae

According to the general procedure (Huang et al., 2016), *m*-toluic acid (1.0 mmol) was reacted with iodobenzene (1.5 equiv), $[Ru(p\text{-cym})Cl_2]_2$ (4 mol%), tricyclohexylphosphine tetrafluoroborate (8 mol%) and K_2CO_3 (1.0 equiv) in *N*-methyl-2-pyrrolidone (0.20 M) for 15 h at 100 °C. The reaction mixture was charged with iodomethane (3.0 equiv), K_2CO_3 (2.5 equiv) and acetonitrile (0.50 M) and heated for 2 h at 60 °C. Work-up and chromatography afforded methyl 4-methyl-[1,1'-biphenyl]-2-carboxylate in 93% yield (210.5 mg). A 20 mL vial was charged with methyl 4-methyl-[1,1'-biphenyl]-2-carboxylate (0.93 mmol), NaOH (10.0 equiv), methanol (1 mL) and water (10 mL) and heated for 12 h at 80 °C. The organic solvent was removed and the reaction mixture was acidified with HCl (6.0 *N*, 5 mL). The precipitate was filtered and washed with water (3 × 10 mL) to afford the product in 90% yield (177.7 mg).

White solid. The reaction of 4-methyl-[1,1'-biphenyl]-2-carboxylic acid (0.20 mmol), 4-methoxyphenyl boronic acid (2.0 equiv), $Pd(OAc)_2$ (5 mol%), 1,4-bis(diphenylphosphino)butane (10 mol%), triethylamine (1.5 equiv), H_3BO_3 (1.5 equiv) and trimethylacetic anhydride (1.5 equiv) in 1,4-dioxane (0.20 M) for 15 h at 160 °C, afforded after work-up and chromatography the title compound in 52% yield (28.6 mg). White solid. **^{1}H NMR (500 MHz, $CDCl_3$)** δ 7.39-7.37 (d, J = 10.6 Hz, 1 H), 7.34-7.32 (d, J = 7.7 Hz, 1 H), 7.25-7.22 (t, J = 7.3 Hz, 4 H), 7.16-7.15 (d, J = 6.7 Hz, 2 H), 7.08-7.07 (d, J = 8.4 Hz, 2 H), 6.78-6.77 (d, J = 8.3 Hz, 2 H), 3.80 (s, 3 H), 2.45 (s, 3 H). **^{13}C NMR (125 MHz, $CDCl_3$)** δ 158.27, 141.68, 139.98, 137.69, 137.15, 134.03, 131.30, 130.89, 130.58, 129.90, 128.16, 127.85, 126.16, 113.32, 55.17, 21.10. The spectral data matched those reported in the literature (Campo et al., 2007).

3'-Methoxy-[1,1'-biphenyl]-3-carboxylic acid and 2-methoxyphenylboronic acid (3af, Figure 3, Entry 32)

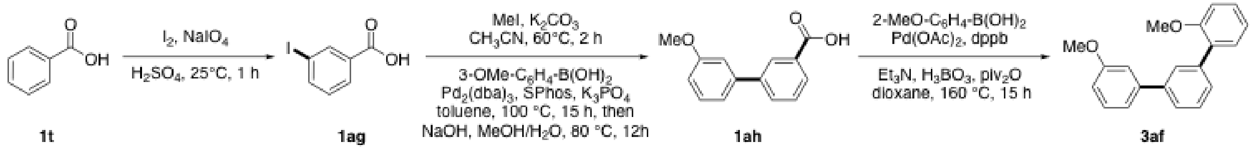

According to the general procedure (Kraszkiewicz et al., 2006), iodine (3.0 mmol) and $NaIO_4$ (1.0 mmol) were dropwise added to stirred 95% H_2SO_4 (15.0 mL). Stirring was continued for 30 min at 25 °C to give a dark brown iodinating solution containing ca. 7.0 mmol (1.17 equiv) of the I^+ intermediate. Benzoic acid (6.0 mmol) was added in one portion to the iodinating solution containing the I^+ intermediate (1.17 equiv) and the resulting solution was stirred for 1 h at 25 °C. Then the reaction mixture was slowly poured into stirred ice water (50 g). The crude solid product was filtered and washed with water (3 × 10 mL) to afforded 3-iodobenzoic acid in 72% yield (1.072 g). White solid. According to the general procedure (Huang et al., 2016), 3-iodobenzoic acid (4.32 mmol), was charged with iodomethane (3.0 equiv), K_2CO_3 (2.5 equiv) and acetonitrile (10.0 mL) and heated for 2 h at 60 °C. Work-up and chromatography afforded methyl 3-iodobenzoate in 91% yield (1.031 g). According to the general procedure (Barder et al., 2005), the reaction of 3-iodobenzoate (1.0 mmol), (3-methoxyphenyl)boronic acid (1.5 equiv), $Pd_2(dba)_3$ (1 mol%), SPhos (4 mol%) and K_3PO_4 (2.0 equiv) in toluene (0.25 M) for 15 h at 100 °C, afforded after work-up and chromatography methyl 3'-methoxy-[1,1'-biphenyl]-3-carboxylate in 98% yield (237.4 mg). White solid. A 20 mL vial was charged with methyl 3'-

methoxy-[1,1'-biphenyl]-3-carboxylate (0.98 mmol), NaOH (10.0 equiv) in methanol (1 mL) and water (10 mL) and heated for 12 h at 80 °C. The organic solvent was removed and the reaction mixture was acidified with HCl (6.0 *N*, 5 mL). The precipitate was filtered and washed with water (3 × 10 mL) to afford the product in 89% yield (199.1 mg). White solid. The reaction of 3'-methoxy-[1,1'-biphenyl]-3-carboxylic acid (0.20 mmol), 2-methoxyphenylboronic acid (2.0 equiv), $Pd(OAc)_2$ (5 mol%), 1,4-bis(diphenylphosphino)butane (10 mol%), triethylamine (1.5 equiv), H_3BO_3 (1.5 equiv) and trimethylacetic anhydride (1.5 equiv) in 1,4-dioxane (0.20 M) for 15 h at 160 °C, afforded after work-up and chromatography the title compound in 67% yield (38.9 mg). New compound. Colorless oil. **^{1}H NMR (500 MHz, $CDCl_3$)** δ 7.78 (s, 1 H), 7.59-7.55 (t, *J* = 8.1 Hz, 2 H), 7.52-7.49 (t, *J* = 7.5 Hz, 1 H), 7.42-7.36 (m, 3 H), 7.27-7.25 (d, *J* = 7.5 Hz, 1 H), 7.20 (s, 1 H), 7.10-7.07 (t, *J* = 7.3 Hz, 1 H), 7.05-7.04 (d, *J* = 8.1 Hz, 1 H), 6.94-6.93 (d, *J* = 7.8 Hz, 1 H), 3.90 (s, 3 H), 3.86 (s, 3 H). **^{13}C NMR (125 MHz, $CDCl_3$)** δ 159.94, 156.53, 142.96, 140.89, 139.00, 130.94, 130.59, 129.71, 128.78, 128.70, 128.53, 128.36, 125.85, 120.88, 119.83, 112.98, 112.72, 111.26, 55.61, 55.32. **HRMS** calcd for $C_{20}H_{18}O_2Na$ (M^+ + Na) 313.1199, found 313.1202.

Quinoline-6-carboxylic acid and 2-naphthylboronic acid (3ag, Figure 3, Entry 33)

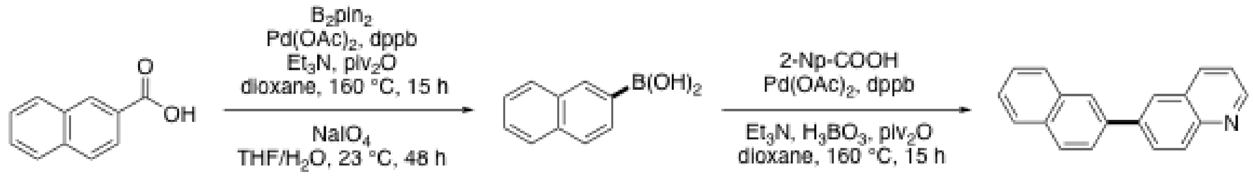

1k 2ax 3ag

According to the general procedure (Liu et al., 2018), the reaction of 2-naphthoic acid (1.0 mmol), bis(pinacolato)diboron (3.0 equiv), $Pd(OAc)_2$ (5 mol%), 1,4-bis(diphenylphosphino)butane (10 mol%), triethylamine (3.0 equiv) and trimethylacetic anhydride (3.0 equiv) in 1,4-dioxane (0.20 M) for 15 h at 160 °C, afforded after work-up and chromatography 4,4,5,5-tetramethyl-2-(naphthalen-2-yl)-1,3,2-dioxaborolane in 76% yield (193.2 mg). White solid. According to the general procedure (Crawford et al., 2012), the reaction of 4,4,5,5-tetramethyl-2-(naphthalen-2-yl)-1,3,2-dioxaborolane (0.76 mmol), $NaIO_4$ (3.0 equiv) in THF/H_2O (2.8 mL/0.7 mL) for 48 h at room temperature, afforded after work-up 2-naphthylboronic acid in 86% yield (112.4 mg). The reaction of 2-naphthylboronic acid (0.20 mmol), quinoline-6-carboxylic acid (1.0 equiv), $Pd(OAc)_2$ (5 mol%), 1,4-

bis(diphenylphosphino)butane (10 mol%), triethylamine (1.5 equiv), H_3BO_3 (1.5 equiv) and trimethylacetic anhydride (1.5 equiv) in 1,4-dioxane (0.20 M) for 15 h at 160 °C, afforded after work-up and chromatography the title compound in 72% yield (36.8 mg). *New compound.* White solid. **Mp** = 144-146 °C. **^{1}H NMR (500 MHz, CDCl$_3$)** δ 8.97 (s, 1 H), 8.28-8.24 (t, *J* = 8.8 Hz, 2 H), 8.20 (s, 1 H), 8.16 (s, 2 H), 8.01-7.96 (m, 2 H), 7.93-7.89 (m, 2 H), 7.57-7.54 (m, 2 H), 7.49-7.47 (m, 1 H). **^{13}C NMR (125 MHz, CDCl$_3$)** δ 150.46, 147.77, 139.25, 137.63, 136.28, 133.71, 132.83, 130.03, 129.41, 128.72, 128.54, 128.30, 127.72, 126.54, 126.39, 126.29, 125.80, 125.55, 121.56. **HRMS** calcd for $C_{19}H_{13}NNa$ (M^+ + Na) 278.0940, found 278.0961.

[1,1’-Biphenyl]-4-carboxylic acid and 2-methoxyphenylboronic acid (3ah, Figure 3, Entry 34)

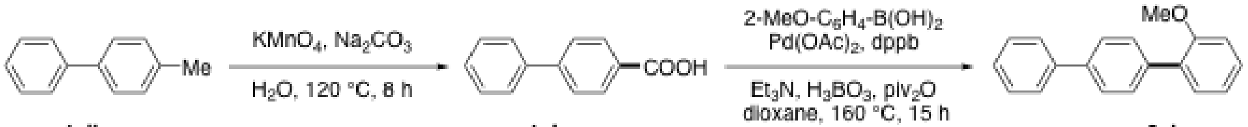

According to the general procedure (Ni et al., 2016), methyl 4-methyl-1,1’-biphenyl (2.0 mmol) was reacted with $KMnO_4$ (2.5 equiv), Na_2CO_3 (1.0 equiv) in H_2O (0.2 M) for 8 h at 120 °C. The reaction mixture was filtered through a pad of celite, and the filtrate was acidified with HCl (6.0 *N*, 5 mL). The precipitate was filtered and washed with water (3 × 10 mL) to afford [1,1’-biphenyl]-4-carboxylic acid in 87% yield (344.9 mg). White solid. The reaction of [1,1’-biphenyl]-4-carboxylic acid (0.20 mmol), 2-methoxyphenylboronic acid (2.0 equiv), $Pd(OAc)_2$ (5 mol%), 1,4-bis(diphenylphosphino)butane (10 mol%), triethylamine (1.5 equiv), H_3BO_3 (1.5 equiv) and trimethylacetic anhydride (1.5 equiv) in 1,4-dioxane (0.20 M) for 15 h at 160 °C, afforded after work-up and chromatography the title compound in 92% yield (47.9 mg). White solid. **^{1}H NMR (500 MHz, CDCl$_3$)** δ 7.70-7.66 (m, 6 H), 7.51-7.48 (t, *J* = 7.2 Hz, 2 H), 7.43-7.37 (m, 3 H), 7.11-7.08 (t, *J* = 7.3 Hz, 1 H), 7.06-7.04 (d, *J* = 8.2 Hz, 1 H), 3.88 (s, 3 H). **^{13}C NMR (125 MHz, CDCl$_3$)** δ 156.58, 141.07, 139.78, 137.57, 130.85, 130.27, 129.95, 128.78, 128.73, 127.21, 127.16, 126.81, 120.93, 111.29, 55.61. The spectral data matched those reported in the literature (Miguez et al., 2007).

Nicotinic acid and 4-acetylphenylboronic acid (3ai, Figure 4, Entry 35)

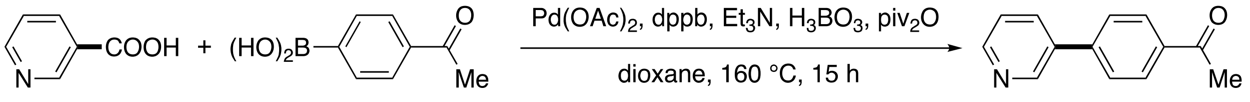

1m **2c** **3ai**

According to the general procedure, the reaction of nicotinic acid (0.20 mmol), 4-acetylphenylboronic acid (2.0 equiv), $Pd(OAc)_2$ (5 mol%), 1,4-bis(diphenylphosphino)butane (10 mol%), triethylamine (1.5 equiv), H_3BO_3 (1.5 equiv) and trimethylacetic anhydride (1.5 equiv) in 1,4-dioxane (0.20 M) for 15 h at 160 °C, afforded after work-up and chromatography the title compound in 86% yield (34.0 mg). White solid. **^{1}H NMR (500 MHz, $CDCl_3$)** δ 8.91 (s, 1 H), 8.68-8.67 (d, *J* = 4.0 Hz, 1 H), 8.11-8.09 (d, *J* = 8.2 Hz, 2 H), 7.95-7.94 (d, *J* = 7.9 Hz, 1 H), 7.72-7.71 (d, *J* = 8.3 Hz, 2 H), 7.45-7.42 (m, 1 H), 2.68 (s, 3 H). **^{13}C NMR (125 MHz, $CDCl_3$)** δ 197.59, 149.30, 148.32, 142.35, 136.56, 135.50, 134.56, 129.16, 127.32, 123.73, 26.72. The spectral data matched those reported in the literature (Adak et al., 2011).

Nicotinic acid and 4-(methoxycarbonyl)phenylboronic acid (3aj, Figure 4, Entry 36)

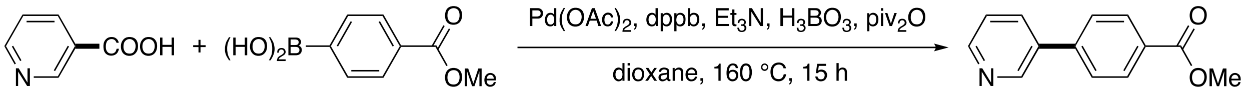

1m **2d** **3aj**

According to the general procedure, the reaction of nicotinic acid (0.20 mmol), 4-(methoxycarbonyl)phenylboronic acid (2.0 equiv), $Pd(OAc)_2$ (5 mol%), 1,4-bis(diphenylphosphino)butane (10 mol%), triethylamine (1.5 equiv), H_3BO_3 (1.5 equiv) and trimethylacetic anhydride (1.5 equiv) in 1,4-dioxane (0.20 M) for 15 h at 160 °C, afforded after work-up and chromatography the title compound in 98% yield (41.8 mg). White solid. **^{1}H NMR (500 MHz, $CDCl_3$)** δ 8.90 (s, 1 H), 8.66-8.65 (d, *J* = 3.8 Hz, 1 H), 8.17-8.15 (d, *J* = 8.3 Hz, 2 H), 7.93-7.92 (d, *J* = 7.9 Hz, 1 H), 7.68-7.66 (d, *J* = 8.3 Hz, 2 H), 7.42-7.40 (m, 1 H), 3.96 (s, 3 H). **^{13}C NMR (125 MHz, $CDCl_3$)** δ 166.73, 149.26, 148.35, 142.22, 135.56, 134.52, 130.37, 129.76, 127.10, 123.68, 52.26. The spectral data matched those reported in the literature (Muto et al., 2015).

Nicotinic acid and 4-formylphenylboronic acid (3ak, Figure 4, Entry 37)

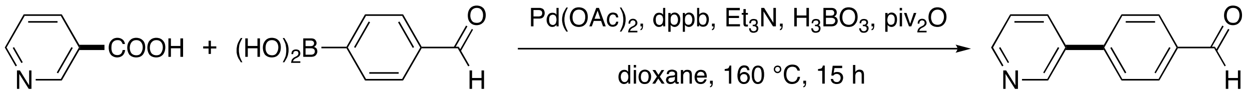

1m **2e** **3ak**

According to the general procedure, the reaction of nicotinic acid (0.20 mmol), 4-formylphenylboronic acid (2.0 equiv), $Pd(OAc)_2$ (5 mol%), 1,4-bis(diphenylphosphino)butane (10 mol%), triethylamine (1.5 equiv), H_3BO_3 (1.5 equiv) and trimethylacetic anhydride (1.5 equiv) in 1,4-dioxane (0.20 M) for 15 h at 160 °C, afforded after work-up and chromatography the title compound in 72% yield (26.4 mg). White solid. **^{1}H NMR (500 MHz, $CDCl_3$)** δ 10.11 (s, 1 H), 8.93 (s, 1 H), 8.70-8.69 (d, *J* = 4.0 Hz, 1 H), 8.04-8.02 (d, *J* = 8.1 Hz, 2 H), 7.97-7.95 (d, *J* = 7.9 Hz, 1 H), 7.80-7.78 (d, *J* = 8.1 Hz, 2 H), 7.46-7.44 (m, 1 H). **^{13}C NMR (125 MHz, $CDCl_3$)** δ 191.73, 149.56, 148.40, 143.77, 135.83, 135.36, 134.63, 130.49, 127.79, 123.76. The spectral data matched those reported in the literature (Rao et al., 2018).

Nicotinic acid and 4-(trifluoromethyl)phenylboronic acid (3al, Figure 4, Entry 38)

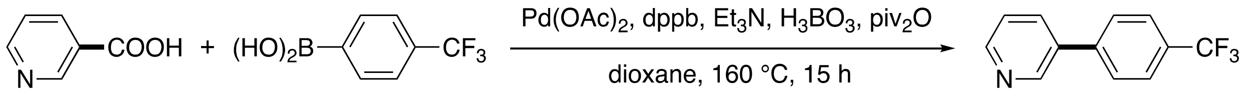

1m **2f** **3al**

According to the general procedure, the reaction of nicotinic acid (0.20 mmol), 4-(trifluoromethyl)phenylboronic acid (2.0 equiv), $Pd(OAc)_2$ (5 mol%), 1,4-bis(diphenylphosphino)butane (10 mol%), triethylamine (1.5 equiv), H_3BO_3 (1.5 equiv) and trimethylacetic anhydride (1.5 equiv) in 1,4-dioxane (0.20 M) for 15 h at 160 °C, afforded after work-up and chromatography the title compound in 82% yield (36.6 mg). White solid. **^{1}H NMR (500 MHz, $CDCl_3$)** δ 8.88 (s, 1 H), 8.68-8.67 (d, *J* = 3.8 Hz, 1 H), 7.92-7.91 (d, *J* = 7.9 Hz, 1 H), 7.77-7.71 (m, 4 H), 7.44-7.42 (m, 1 H). **^{13}C NMR (125 MHz, $CDCl_3$)** δ 149.39, 148.36, 141.41, 135.32, 134.54, 130.26 (q, J^F = 32.6 Hz), 127.52, 126.07 (q, J^F = 3.6 Hz), 124.10 (q, J^F = 270.4 Hz), 123.72. **^{19}F NMR (471 MHz, $CDCl_3$)** δ -62.58. The spectral data matched those reported in the literature (Muto et al., 2015).

Nicotinic acid and 4-cyanophenylboronic acid (3am, Figure 4, Entry 39)

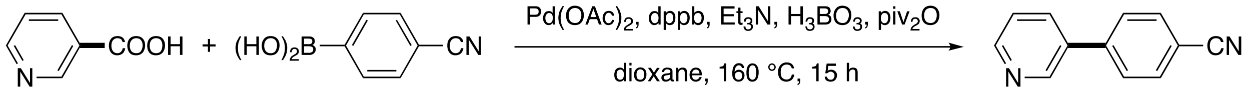

1m **2g** **3am**

According to the general procedure, the reaction of nicotinic acid (0.20 mmol), 4-cyanophenylboronic acid (2.0 equiv), $Pd(OAc)_2$ (5 mol%), 1,4-bis(diphenylphosphino)butane (10 mol%), triethylamine (1.5 equiv), H_3BO_3 (1.5 equiv) and trimethylacetic anhydride (1.5 equiv) in 1,4-dioxane (0.20 M) for 15 h at 160 °C, afforded after work-up and chromatography the title compound in 70% yield (25.3 mg). White solid. **^{1}H NMR (500 MHz, $CDCl_3$)** δ 8.88 (s, 1 H), 8.70-8.69 (d, *J* = 4.0 Hz, 1 H), 7.92-7.91 (d, *J* = 7.9 Hz, 1 H), 7.81-7.79 (d, *J* = 8.4 Hz, 2 H), 7.72-7.71 (d, *J* = 8.4 Hz, 2 H), 7.46-7.44 (m, 1 H). **^{13}C NMR (125 MHz, $CDCl_3$)** δ 149.73, 148.22, 142.32, 134.85, 134.56, 132.91, 127.82, 123.85, 118.56, 111.99. The spectral data matched those reported in the literature (Wang et al., 2015).

Nicotinic acid and 4-methoxyphenylboronic acid (3an, Figure 4, Entry 40)

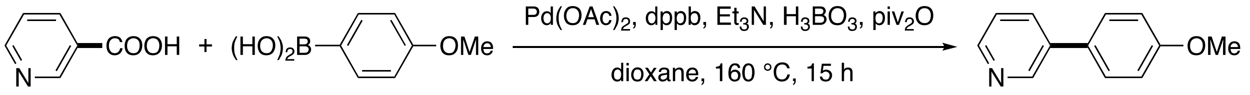

1m **2h** **3an**

According to the general procedure, the reaction of nicotinic acid (0.20 mmol), 4-methoxyphenylboronic acid (2.0 equiv), $Pd(OAc)_2$ (5 mol%), 1,4-bis(diphenylphosphino)butane (10 mol%), triethylamine (1.5 equiv), H_3BO_3 (1.5 equiv) and trimethylacetic anhydride (1.5 equiv) in 1,4-dioxane (0.20 M) for 15 h at 160 °C, afforded after work-up and chromatography the title compound in 75% yield (27.8 mg). White solid. **^{1}H NMR (500 MHz, $CDCl_3$)** δ 8.84 (s, 1 H), 8.57-8.56 (d, *J* = 4.6 Hz, 1 H), 7.86-7.84 (m, 1 H), 7.55-7.53 (d, *J* = 8.6 Hz, 2 H), 7.37-7.34 (m, 1 H), 7.04-7.03 (d, *J* = 8.7 Hz, 2 H), 3.88 (s, 3 H). **^{13}C NMR (125 MHz, $CDCl_3$)** δ 159.78, 148.00, 147.88, 136.27, 133.88, 130.27, 128.24, 123.52, 114.57, 55.40. The spectral data matched those reported in the literature (Muto et al., 2015).

Nicotinic acid and 4-fluorophenylboronic acid (3ao, Figure 4, Entry 41)

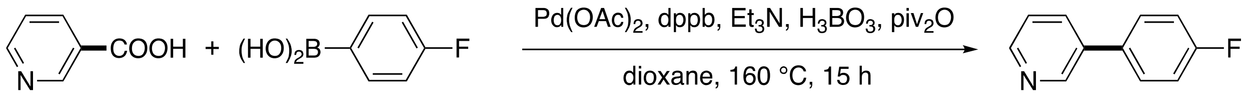

1m **2i** **3ao**

According to the general procedure, the reaction of nicotinic acid (0.20 mmol), 4-fluorophenylboronic acid (2.0 equiv), $Pd(OAc)_2$ (5 mol%), 1,4-bis(diphenylphosphino)butane (10 mol%), triethylamine (1.5 equiv), H_3BO_3 (1.5 equiv) and trimethylacetic anhydride (1.5 equiv) in 1,4-dioxane (0.20 M) for 15 h at 160 °C, afforded after work-up and chromatography the title compound in 76% yield (26.4 mg). White solid. **^{1}H NMR (500 MHz, $CDCl_3$)** δ 8.83 (s, 1 H), 8.62-8.61 (d, J = 3.8 Hz, 1 H), 7.86-7.84 (d, J = 7.9 Hz, 1 H), 7.58-7.55 (m, 2 H), 7.40-7.37 (m, 1 H), 7.21-7.18 (t, J = 8.6 Hz, 2 H). **^{13}C NMR (125 MHz, $CDCl_3$)** δ 162.95 (d, J^F = 246.3 Hz), 148.48, 148.14, 135.78, 134.25, 133.98, 128.86 (d, J^F = 8.1 Hz), 123.61, 116.10 (d, J^F = 21.5 Hz). **^{19}F NMR (471 MHz, $CDCl_3$)** δ -114.19. The spectral data matched those reported in the literature (Muto et al., 2015).

Nicotinic acid and 2-fluorophenylboronic acid (3ap, Figure 4, Entry 42)

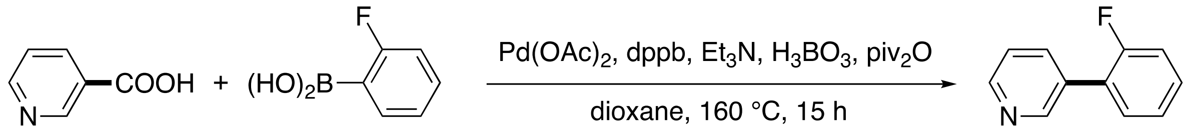

1m **2j** **3ap**

According to the general procedure, the reaction of nicotinic acid (0.20 mmol), 2-fluorophenylboronic acid (2.0 equiv), $Pd(OAc)_2$ (5 mol%), 1,4-bis(diphenylphosphino)butane (10 mol%), triethylamine (1.5 equiv), H_3BO_3 (1.5 equiv) and trimethylacetic anhydride (1.5 equiv) in 1,4-dioxane (0.20 M) for 15 h at 160 °C, afforded after work-up and chromatography the title compound in 90% yield (31.2 mg). White solid. **^{1}H NMR (500 MHz, $CDCl_3$)** δ 8.82 (s, 1 H), 8.64-8.64 (d, J = 3.9 Hz, 1 H), 7.91-7.90 (d, J = 7.8 Hz, 1 H), 7.48-7.39 (m, 3 H), 7.30-7.27 (t, J = 6.0 Hz, 1 H), 7.24-7.20 (t, J = 9.0 Hz, 1 H). **^{13}C NMR (125 MHz, $CDCl_3$)** δ 159.87 (d, J^F = 247.1 Hz), 149.65 (d, J^F = 3.1 Hz), 148.77, 136.37 (d, J^F = 3.3 Hz), 134.10 (d, J^F = 8.5 Hz), 131.68, 130.51 (d, J^F = 3.2 Hz), 129.99 (d, J^F = 8.2 Hz), 124.71 (d, J^F = 3.7 Hz), 123.29, 116.32 (d, J^F = 22.2 Hz). **^{19}F NMR (471 MHz, $CDCl_3$)** δ -117.94. The spectral data matched those reported in the literature (Roesner et al., 2016).

Nicotinic acid and 2,4-difluorophenylboronic acid (3aq, Figure 4, Entry 43)

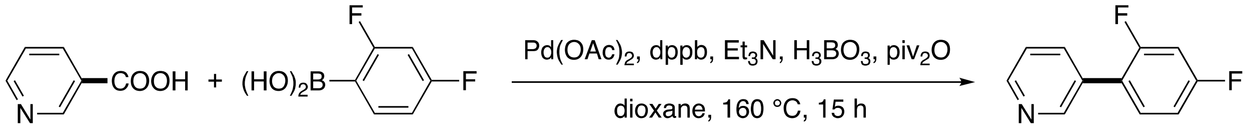

1m **2k** **3aq**

According to the general procedure, the reaction of nicotinic acid (0.20 mmol), 2,4-difluorophenylboronic acid (2.0 equiv), $Pd(OAc)_2$ (5 mol%), 1,4-bis(diphenylphosphino)butane (10 mol%), triethylamine (1.5 equiv), H_3BO_3 (1.5 equiv) and trimethylacetic anhydride (1.5 equiv) in 1,4-dioxane (0.20 M) for 15 h at 160 °C, afforded after work-up and chromatography the title compound in 80% yield (30.6 mg). *New compound.* White solid. **Mp** = 54-56 °C. **^{1}H NMR (500 MHz, $CDCl_3$)** δ 8.77 (s, 1 H), 8.64-8.64 (d, J = 3.8 Hz, 1 H), 7.86-7.84 (dd, J = 7.8 Hz, 1 H), 7.46-7.39 (m, 2 H), 7.04-6.96 (m, 2 H). **^{13}C NMR (125 MHz, $CDCl_3$)** δ 162.88 (dd, J^F = 249.0 Hz), 159.95 (dd, J^F = 249.7 Hz), 149.52 (d, J^F = 2.9 Hz), 148.89, 136.24 (d, J^F = 3.2 Hz), 131.28 (dd, J^F = 9.5 Hz), 130.89, 123.35, 121.93 (dd, J^F = 13.9 Hz), 112.04 (dd, J^F = 21.2 Hz), 104.71 (dd, J^F = 25.5 Hz). **^{19}F NMR (471 MHz, $CDCl_3$)** δ -109.66, -113.37. **HRMS** calcd for $C_{11}H_7NF_2Na$ (M^+ + Na) 214.0439, found 214.0431.

Nicotinic acid and 2-methoxyphenylboronic acid (3ar, Figure 4, Entry 44)

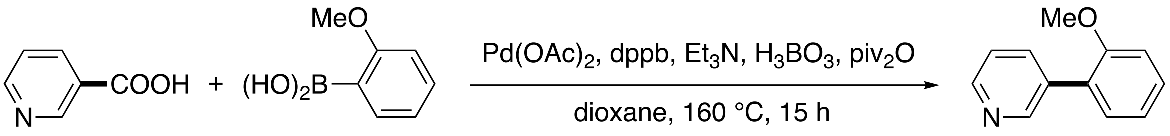

1m **2l** **3ar**

According to the general procedure, the reaction of nicotinic acid (0.20 mmol), 2-methoxyphenylboronic acid (2.0 equiv), $Pd(OAc)_2$ (5 mol%), 1,4-bis(diphenylphosphino)butane (10 mol%), triethylamine (1.5 equiv), H_3BO_3 (1.5 equiv) and trimethylacetic anhydride (1.5 equiv) in 1,4-dioxane (0.20 M) for 15 h at 160 °C, afforded after work-up and chromatography the title compound in 77% yield (28.6 mg). White solid. **^{1}H NMR (500 MHz, $CDCl_3$)** δ 8.81 (s, 1 H), 8.60-8.59 (d, J = 3.9 Hz, 1 H), 7.91-7.90 (d, J = 7.9 Hz, 1 H), 7.42-7.34 (m, 3 H), 7.10-7.07 (t, J = 7.5 Hz, 1 H), 7.04-7.03 (d, J = 8.3 Hz, 1 H), 3.85 (s, 3 H). **^{13}C NMR (125 MHz, $CDCl_3$)** δ 156.57, 150.20, 147.81, 137.14, 134.42, 130.67, 129.66, 126.87, 123.04, 121.08, 111.30, 55.53. The spectral data matched those reported in the literature (Zhang et al., 2017).

Nicotinic acid and 3-methoxyphenylboronic acid (3as, Figure 4, Entry 45)

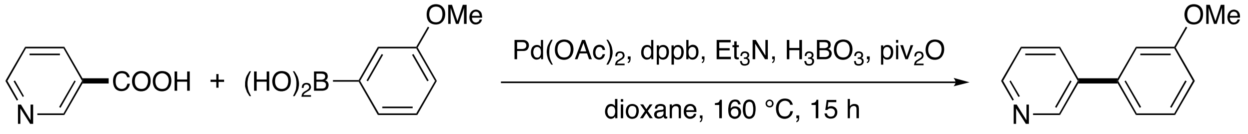

1m **2m** **3as**

According to the general procedure, the reaction of nicotinic acid (0.20 mmol), 3-methoxyphenylboronic acid (2.0 equiv), $Pd(OAc)_2$ (5 mol%), 1,4-bis(diphenylphosphino)butane (10 mol%), triethylamine (1.5 equiv), H_3BO_3 (1.5 equiv) and trimethylacetic anhydride (1.5 equiv) in 1,4-dioxane (0.20 M) for 15 h at 160 °C, afforded after work-up and chromatography the title compound in 86% yield (31.9 mg). White solid. **^{1}H NMR (500 MHz, $CDCl_3$)** δ 8.87 (s, 1 H), 8.62-8.61 (d, *J* = 4.2 Hz, 1 H), 7.90-7.88 (d, *J* = 7.9 Hz, 1 H), 7.44-7.37 (m, 2 H), 7.20-7.18 (d, *J* = 7.4 Hz, 1 H), 7.13 (s, 1 H), 6.99-6.96 (m, 1 H), 3.90 (s, 3 H). **^{13}C NMR (125 MHz, $CDCl_3$)** δ 160.15, 148.60, 148.35, 139.32, 136.56, 134.45, 130.16, 123.55, 119.62, 113.43, 112.98, 55.38. The spectral data matched those reported in the literature (Muto et al., 2015).

Nicotinic acid and 3,4-dimethoxyphenylboronic acid (3at, Figure 4, Entry 46)

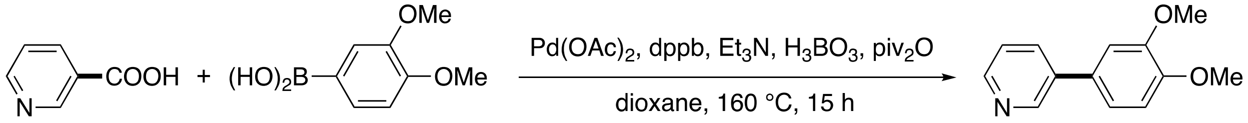

1m **2n** **3at**

According to the general procedure, the reaction of nicotinic acid (0.20 mmol), 3,4-dimethoxyphenylboronic acid (2.0 equiv), $Pd(OAc)_2$ (5 mol%), 1,4-bis(diphenylphosphino)butane (10 mol%), triethylamine (1.5 equiv), H_3BO_3 (1.5 equiv) and trimethylacetic anhydride (1.5 equiv) in 1,4-dioxane (0.20 M) for 15 h at 160 °C, afforded after work-up and chromatography the title compound in 84% yield (36.2 mg). White solid. **^{1}H NMR (500 MHz, $CDCl_3$)** δ 8.85 (s, 1 H), 8.59-8.58 (d, *J* = 4.0 Hz, 1 H), 7.87-7.86 (d, *J* = 7.9 Hz, 1 H), 7.38-7.36 (m, 1 H), 7.18-7.16 (m, 1 H), 7.11-7.11 (d, *J* = 1.8 Hz, 1 H), 7.01-7.00 (d, *J* = 8.3 Hz, 1 H), 3.99 (s, 3 H), 3.96 (s, 3 H). **^{13}C NMR (125 MHz, $CDCl_3$)** δ 149.47, 149.29, 148.07, 148.00, 136.54, 134.07, 130.69, 123.53, 119.63, 111.70, 110.28, 56.04. The spectral data matched those reported in the literature (Muto et al., 2015).

Nicotinic acid and 3-(trifluoromethyl)phenylboronic acid (3au, Figure 4, Entry 47)

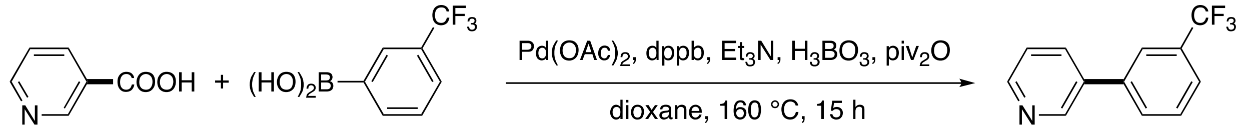

1m **2o** **3au**

According to the general procedure, the reaction of nicotinic acid (0.20 mmol), 3-(trifluoromethyl)phenylboronic acid (2.0 equiv), $Pd(OAc)_2$ (5 mol%), 1,4-bis(diphenylphosphino)butane (10 mol%), triethylamine (1.5 equiv), H_3BO_3 (1.5 equiv) and trimethylacetic anhydride (1.5 equiv) in 1,4-dioxane (0.20 M) for 15 h at 160 °C, afforded after work-up and chromatography the title compound in 87% yield (38.9 mg). White solid. **^{1}H NMR (500 MHz, $CDCl_3$)** δ 8.92 (s, 1 H), 8.72-8.71 (d, *J* = 4.1 Hz, 1 H), 7.96-7.95 (d, *J* = 7.9 Hz, 1 H), 7.85 (s, 1 H), 7.80-7.78 (d, *J* = 7.7 Hz, 1 H), 7.72-7.70 (d, *J* = 7.5 Hz, 1 H), 7.66-7.63 (t, *J* = 7.8 Hz, 1 H), 7.49-7.46 (m, 1 H). **^{13}C NMR (125 MHz, $CDCl_3$)** δ 148.69, 147.73, 138.47, 135.63, 134.96, 131.79, 131.53, 130.47, 129.71, 128.08, 124.99 (q, J^F = 3.4 Hz), 124.02 (q, J^F = 3.7 Hz). **^{19}F NMR (471 MHz, $CDCl_3$)** δ -62.71. The spectral data matched those reported in the literature (Barder et al., 2005).

Nicotinic acid and 3-acetylphenylboronic acid (3av, Figure 4, Entry 48)

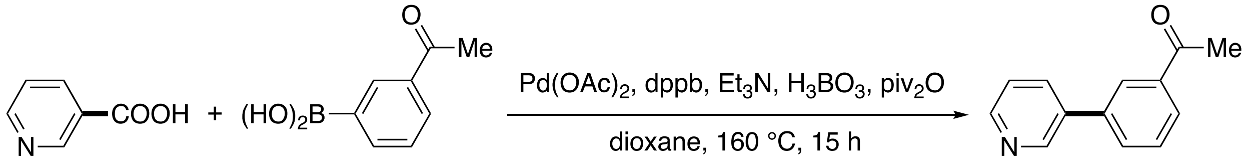

1m **2p** **3av**

According to the general procedure, the reaction of nicotinic acid (0.20 mmol), 3-acetylphenylboronic acid (2.0 equiv), $Pd(OAc)_2$ (5 mol%), 1,4-bis(diphenylphosphino)butane (10 mol%), triethylamine (1.5 equiv), H_3BO_3 (1.5 equiv) and trimethylacetic anhydride (1.5 equiv) in 1,4-dioxane (0.20 M) for 15 h at 160 °C, afforded after work-up and chromatography the title compound in 98% yield (38.7 mg). White solid. **^{1}H NMR (500 MHz, $CDCl_3$)** δ 8.90 (s, 1 H), 8.66-8.66 (d, *J* = 3.9 Hz, 1 H), 8.20 (s, 1 H), 8.02-8.01 (d, *J* = 7.7 Hz, 1 H), 7.95-7.93 (d, *J* = 7.9 Hz, 1 H), 7.82-7.80 (d, *J* = 7.7 Hz, 1 H), 7.63-7.60 (t, *J* = 7.8 Hz, 1 H), 7.44-7.41 (m, 1 H), 2.69 (s, 3 H). **^{13}C NMR (125 MHz, $CDCl_3$)** δ 197.78, 149.02, 148.29, 138.43, 137.90, 135.76,

134.52, 131.67, 129.44, 128.05, 126.92, 123.69, 26.77. The spectral data matched those reported in the literature (Kuriyama et al., 2013).

Nicotinic acid and thiophen-3-ylboronic acid (3aw, Figure 4, Entry 49)

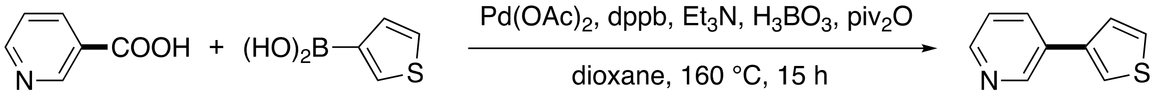

1m **2q** **3aw**

According to the general procedure, the reaction of nicotinic acid (0.20 mmol), thiophen-3-ylboronic acid (2.0 equiv), $Pd(OAc)_2$ (5 mol%), 1,4-bis(diphenylphosphino)butane (10 mol%), triethylamine (1.5 equiv), H_3BO_3 (1.5 equiv) and trimethylacetic anhydride (1.5 equiv) in 1,4-dioxane (0.20 M) for 15 h at 160 °C, afforded after work-up and chromatography the title compound in 80% yield (25.8 mg). White solid. **^{1}H NMR (500 MHz, $CDCl_3$)** δ 8.90 (s, 1 H), 8.56-8.55 (d, *J* = 4.6 Hz, 1 H), 7.90-7.88 (d, *J* = 7.9 Hz, 1 H), 7.55-7.55 (d, *J* = 1.4 Hz, 1 H), 7.48-7.46 (m, 1 H), 7.42-7.41 (m, 1 H), 7.36-7.34 (m, 1 H). **^{13}C NMR (125 MHz, $CDCl_3$)** δ 148.21, 147.68, 138.80, 133.61, 131.60, 127.02, 125.93, 123.68, 121.48. The spectral data matched those reported in the literature (Muto et al., 2015).

Nicotinic acid and naphthalen-2-ylboronic acid (3ax, Figure 4, Entry 50)

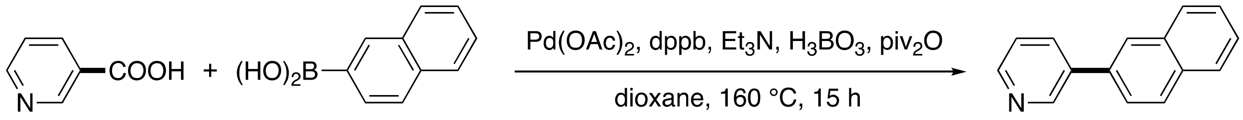

1m **2r** **3ax**

According to the general procedure, the reaction of nicotinic acid (0.20 mmol), naphthalen-2-ylboronic acid (2.0 equiv), $Pd(OAc)_2$ (5 mol%), 1,4-bis(diphenylphosphino)butane (10 mol%), triethylamine (1.5 equiv), H_3BO_3 (1.5 equiv) and trimethylacetic anhydride (1.5 equiv) in 1,4-dioxane (0.20 M) for 15 h at 160 °C, afforded after work-up and chromatography the title compound in 86% yield (35.3 mg). White solid. **^{1}H NMR (500 MHz, $CDCl_3$)** δ 9.01 (s, 1 H), 8.66-8.65 (d, *J* = 4.1 Hz, 1 H), 8.08 (s, 1 H), 8.04-8.02 (d, *J* = 7.9 Hz, 1 H), 8.00-7.98 (d, *J* = 8.5 Hz, 1 H), 7.95-7.94 (d, *J* = 7.1 Hz, 1 H), 7.92-7.91 (d, *J* = 8.2 Hz, 1 H), 7.75-7.74 (d, *J* = 8.5 Hz, 1 H), 7.58-7.54 (m, 2 H), 7.45-7.43 (m, 1 H). **^{13}C NMR (125 MHz, $CDCl_3$)** δ 148.61, 148.55, 136.63, 135.17, 134.61, 133.61, 132.90, 128.92, 128.26, 127.74, 126.64, 126.48, 126.20, 125.06, 123.64. The spectral data matched those reported in the literature (Muto et al., 2015).

Nicotinic acid and 6-methoxynaphthalen-2-ylboronic acid (3ay, Figure 4, Entry 51)

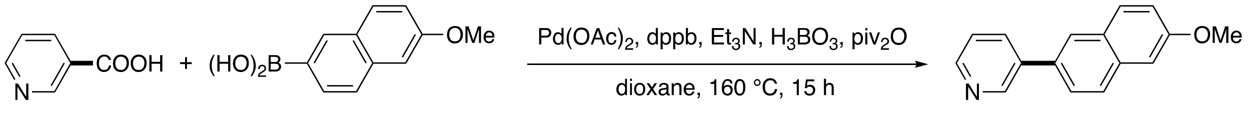

According to the general procedure, the reaction of nicotinic acid (0.20 mmol), 6-methoxynaphthalen-2-ylboronic acid (2.0 equiv), $Pd(OAc)_2$ (5 mol%), 1,4-bis(diphenylphosphino)butane (10 mol%), triethylamine (1.5 equiv), H_3BO_3 (1.5 equiv) and trimethylacetic anhydride (1.5 equiv) in 1,4-dioxane (0.20 M) for 15 h at 160 °C, afforded after work-up and chromatography the title compound in 98% yield (46.2 mg). White solid. **^{1}H NMR (500 MHz, $CDCl_3$)** δ 9.00 (s, 1 H), 8.64-8.63 (d, *J* = 4.0 Hz, 1 H), 8.03-8.00 (m, 2 H), 7.88-7.83 (m, 2 H), 7.71-7.70 (d, *J* = 8.4 Hz, 1 H), 7.44-7.42 (m, 1 H), 7.23-7.20 (m, 2 H), 3.98 (s, 3 H). **^{13}C NMR (125 MHz, $CDCl_3$)** δ 158.16, 148.22, 148.01, 136.81, 134.57, 134.18, 132.83, 129.78, 129.11, 127.73, 126.00, 125.50, 123.69, 119.56, 105.61, 55.39. The spectral data matched those reported in the literature (Lucas et al., 2008).

Nicotinic acid and dibenzo[*b*,*d*]furan-4-ylboronic acid (3az, Figure 4, Entry 52)

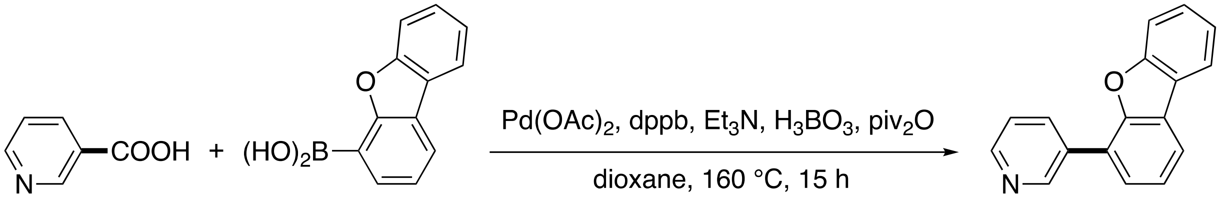

According to the general procedure, the reaction of nicotinic acid (0.20 mmol), dibenzo[*b*,*d*]furan-4-ylboronic acid (2.0 equiv), $Pd(OAc)_2$ (5 mol%), 1,4-bis(diphenylphosphino)butane (10 mol%), triethylamine (1.5 equiv), H_3BO_3 (1.5 equiv) and trimethylacetic anhydride (1.5 equiv) in 1,4-dioxane (0.20 M) for 15 h at 160 °C, afforded after work-up and chromatography the title compound in 69% yield (33.9 mg). White solid. **^{1}H NMR (500 MHz, $CDCl_3$)** δ 9.19 (s, 1 H), 8.69-8.69 (d, *J* = 3.8 Hz, 1 H), 8.28-8.26 (d, *J* = 7.9 Hz, 1 H), 8.03-8.00 (m, 2 H), 7.64-7.62 (m, 2 H), 7.53-7.47 (m, 3 H), 7.42-7.39 (t, *J* = 7.6 Hz, 1 H). **^{13}C NMR (125 MHz, $CDCl_3$)** δ 156.17, 153.37, 149.65, 148.82, 135.98, 132.26, 127.54, 126.52, 125.19, 123.98, 123.51, 123.43, 123.03, 122.36, 120.81, 120.62, 111.89. The spectral data matched those reported in the literature (Ramakrishna et al., 2017).

Nicotinic acid and dibenzo[*b,d*]thiophen-4-ylboronic acid (3ba, Figure 4, Entry 53)

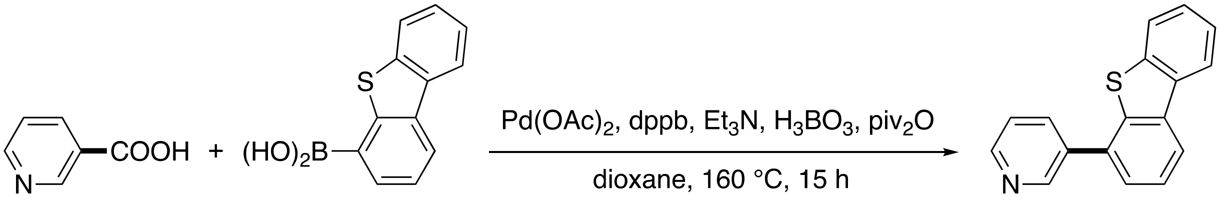

1m **2u** **3ba**

According to the general procedure, the reaction of nicotinic acid (0.20 mmol), dibenzo[*b,d*]thiophen-4-ylboronic acid (2.0 equiv), $Pd(OAc)_2$ (5 mol%), 1,4-bis(diphenylphosphino)butane (10 mol%), triethylamine (1.5 equiv), H_3BO_3 (1.5 equiv) and trimethylacetic anhydride (1.5 equiv) in 1,4-dioxane (0.20 M) for 15 h at 160 °C, afforded after work-up and chromatography the title compound in 65% yield (34.0 mg). <u>*New compound.*</u> White solid. **<u>Mp</u>** = 128-129 °C. **<u>^{1}H NMR (500 MHz, $CDCl_3$)</u>** δ 9.00 (s, 1 H), 8.73-8.72 (d, *J* = 3.7 Hz, 1 H), 8.24-8.22 (d, *J* = 7.8 Hz, 2 H), 8.11-8.10 (d, *J* = 7.8 Hz, 1 H), 7.88-7.86 (m, 1 H), 7.63-7.60 (t, *J* = 7.5 Hz, 1 H), 7.53-7.47 (m, 4 H). **<u>^{13}C NMR (125 MHz, $CDCl_3$)</u>** δ 149.35, 149.21, 139.30, 138.75, 136.52, 136.31, 135.62, 135.53, 133.40, 127.09, 127.05, 125.29, 124.64, 123.54, 122.73, 121.86, 121.27. **<u>HRMS</u>** calcd for $C_{17}H_{11}NS$ (M^+) 261.0607, found 261.0596.

4-(Methoxycarbonyl)benzoic acid and *p*-tolylboronic acid (3bb, Figure 4, Entry 54)

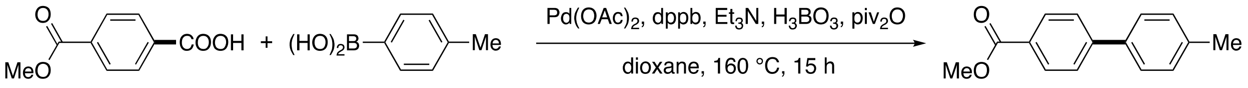

1a **2v** **3bb**

According to the general procedure, the reaction of 4-(methoxycarbonyl)benzoic acid (0.20 mmol), *p*-tolylboronic acid (2.0 equiv), $Pd(OAc)_2$ (5 mol%), 1,4-bis(diphenylphosphino)butane (10 mol%), triethylamine (1.5 equiv), H_3BO_3 (1.5 equiv) and trimethylacetic anhydride (1.5 equiv) in 1,4-dioxane (0.20 M) for 15 h at 160 °C, afforded after work-up and chromatography the title compound in 91% yield (41.2 mg). White solid. **<u>^{1}H NMR (500 MHz, $CDCl_3$)</u>** δ 8.13-8.11 (d, *J* = 8.3 Hz, 2 H), 7.68-7.66 (d, *J* = 8.3 Hz, 2 H), 7.56-7.55 (d, *J* = 8.0 Hz, 2 H), 7.31-7.29 (d, *J* = 7.9 Hz, 2 H), 3.96 (s, 3 H), 2.44 (s, 3 H). **<u>^{13}C NMR (125 MHz, $CDCl_3$)</u>** δ 167.08, 145.59, 138.13, 137.10, 130.10, 129.67, 128.60, 127.12, 126.81, 52.11, 21.18. The spectral data matched those reported in the literature (Kloss et al., 2018).

4-(Methoxycarbonyl)benzoic acid and 4-(*tert*-butyl)phenylboronic acid (3bc, Figure 4, Entry 55)

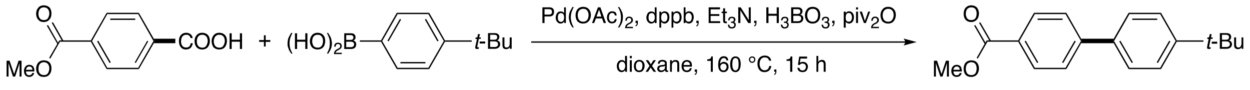

1a **2w** **3bc**

According to the general procedure, the reaction of 4-(methoxycarbonyl)benzoic acid (0.20 mmol), 4-(*tert*-butyl)phenylboronic acid (2.0 equiv), $Pd(OAc)_2$ (5 mol%), 1,4-bis(diphenylphosphino)butane (10 mol%), triethylamine (1.5 equiv), H_3BO_3 (1.5 equiv) and trimethylacetic anhydride (1.5 equiv) in 1,4-dioxane (0.20 M) for 15 h at 160 °C, afforded after work-up and chromatography the title compound in 82% yield (44.1 mg). White solid. **^{1}H NMR (500 MHz, $CDCl_3$)** δ 8.13-8.12 (d, *J* = 8.3 Hz, 2 H), 7.70-7.68 (d, *J* = 8.3 Hz, 2 H), 7.62-7.60 (d, *J* = 8.3 Hz, 2 H), 7.53-7.51 (d, *J* = 8.4 Hz, 2 H), 3.97 (s, 3 H), 1.40 (s, 9 H). **^{13}C NMR (125 MHz, $CDCl_3$)** δ 167.09, 151.36, 145.49, 137.06, 130.09, 128.63, 126.94, 126.85, 125.92, 52.11, 34.65, 31.34. The spectral data matched those reported in the literature (Miao et al., 2017).

4-(Methoxycarbonyl)benzoic acid and *o*-tolylboronic acid (3bd, Figure 4, Entry 56)

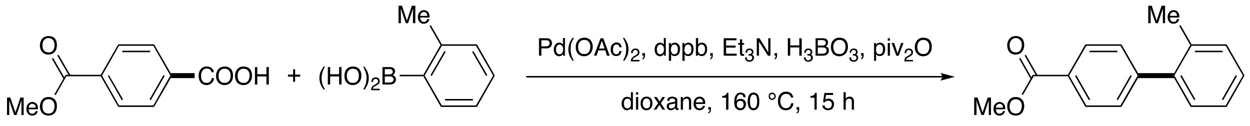

1a **2x** **3bd**

According to the general procedure, the reaction of 4-(methoxycarbonyl)benzoic acid (0.20 mmol), *o*-tolylboronic acid (2.0 equiv), $Pd(OAc)_2$ (5 mol%), 1,4-bis(diphenylphosphino)butane (10 mol%), triethylamine (1.5 equiv), H_3BO_3 (1.5 equiv) and trimethylacetic anhydride (1.5 equiv) in 1,4-dioxane (0.20 M) for 15 h at 160 °C, afforded after work-up and chromatography the title compound in 76% yield (34.4 mg). White solid. **^{1}H NMR (500 MHz, $CDCl_3$)** δ 8.13-8.11 (d, *J* = 8.0 Hz, 2 H), 7.44-7.42 (d, *J* = 7.9 Hz, 2 H), 7.31-7.25 (m, 4 H), 3.98 (s, 3 H), 2.30 (s, 3 H). **^{13}C NMR (125 MHz, $CDCl_3$)** δ 167.09, 146.78, 140.88, 135.19, 130.51, 129.54, 129.43, 129.29, 128.61, 127.85, 125.92, 52.15, 20.40. The spectral data matched those reported in the literature (Luan et al., 2017).

4-(Methoxycarbonyl)benzoic acid and 2-(trifluoromethyl)phenylboronic acid (3be, Figure 4, Entry 57)

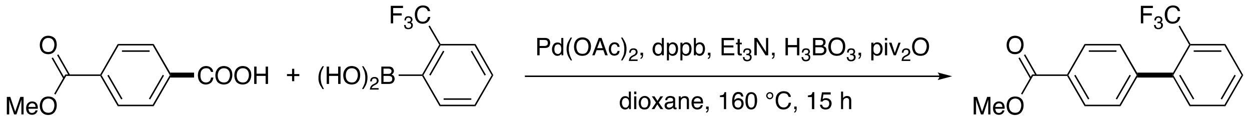

1a **2y** **3be**

According to the general procedure, the reaction of 4-(methoxycarbonyl)benzoic acid (0.20 mmol), 2-(trifluoromethyl)phenylboronic acid (2.0 equiv), $Pd(OAc)_2$ (5 mol%), 1,4-bis(diphenylphosphino)butane (10 mol%), triethylamine (1.5 equiv), H_3BO_3 (1.5 equiv) and trimethylacetic anhydride (1.5 equiv) in 1,4-dioxane (0.20 M) for 15 h at 160 °C, afforded after work-up and chromatography the title compound in 85% yield (47.7 mg). White solid. **^{1}H NMR (500 MHz, $CDCl_3$)** δ 8.11-8.10 (d, J = 8.1 Hz, 2 H), 7.80-7.78 (d, J = 7.8 Hz, 1 H), 7.62-7.59 (t, J = 7.4 Hz, 1 H), 7.54-7.51 (t, J = 7.7 Hz, 1 H), 7.44-7.42 (d, J = 8.0 Hz, 2 H), 7.35-7.34 (d, J = 7.5 Hz, 1 H), 3.97 (s, 3 H). **^{13}C NMR (125 MHz, $CDCl_3$)** δ 166.89, 144.50, 140.31, 131.61, 131.43, 129.50, 129.08, 128.40 (d, J^F = 30.1 Hz), 127.88, 126.21 (d, J^F = 5.2 Hz), 124.00 (d, J^F = 272.3 Hz), 52.19. **^{19}F NMR (471 MHz, $CDCl_3$)** δ -56.81. The spectral data matched those reported in the literature (Tang et al., 2015).

4-(Methoxycarbonyl)benzoic acid and 2-formylphenylboronic acid (3bf, Figure 4, Entry 58)

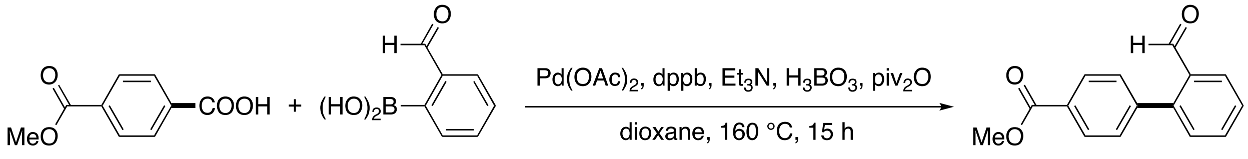

1a **2z** **3bf**

According to the general procedure, the reaction of 4-(methoxycarbonyl)benzoic acid (0.20 mmol), 2-formylphenylboronic acid (2.0 equiv), $Pd(OAc)_2$ (5 mol%), 1,4-bis(diphenylphosphino)butane (10 mol%), triethylamine (1.5 equiv), H_3BO_3 (1.5 equiv) and trimethylacetic anhydride (1.5 equiv) in 1,4-dioxane (0.20 M) for 15 h at 160 °C, afforded after work-up and chromatography the title compound in 73% yield (35.1 mg). White solid. **^{1}H NMR (500 MHz, $CDCl_3$)** δ 9.99 (s, 1 H), 8.18-8.16 (d, J = 8.2 Hz, 2 H), 8.08-8.07 (d, J = 7.7 Hz, 1 H), 7.71-7.68 (t, J = 7.1 Hz, 1 H), 7.59-7.55 (t, J = 7.6 Hz, 1 H), 7.50-7.46 (t, J = 8.4 Hz, 3 H), 3.99 (s, 3 H). **^{13}C NMR (125 MHz, $CDCl_3$)** δ 191.79, 166.68, 144.69, 142.48, 133.72, 133.67,

130.63, 130.11, 129.89, 129.67, 128.45, 127.99, 52.33. The spectral data matched those reported in the literature (Zhao et al., 2007).

4-(Methoxycarbonyl)benzoic acid and 3,5-dichlorophenylboronic acid (3bg, Figure 4, Entry 59)

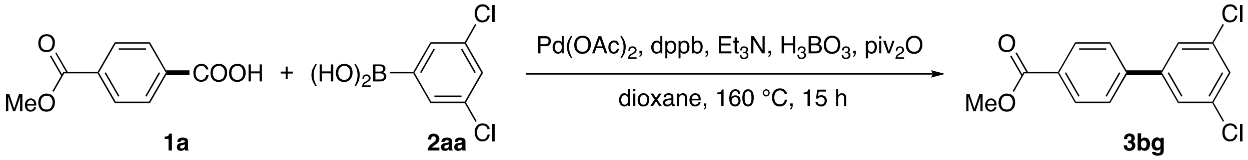

According to the general procedure, the reaction of 4-(methoxycarbonyl)benzoic acid (0.20 mmol), 3,5-dichlorophenylboronic acid (2.0 equiv), $Pd(OAc)_2$ (5 mol%), 1,4-bis(diphenylphosphino)butane (10 mol%), triethylamine (1.5 equiv), H_3BO_3 (1.5 equiv) and trimethylacetic anhydride (1.5 equiv) in 1,4-dioxane (0.20 M) for 15 h at 160 °C, afforded after work-up and chromatography the title compound in 62% yield (34.9 mg). White solid. **^{1}H NMR (500 MHz, $CDCl_3$)** δ 8.15-8.13 (d, *J* = 7.5 Hz, 2 H), 7.64-7.62 (d, *J* = 7.2 Hz, 2 H), 7.51 (s, 2 H), 7.41 (s, 1 H), 3.97 (s, 3 H). **^{13}C NMR (125 MHz, $CDCl_3$)** δ 166.63, 143.00, 142.81, 135.53, 130.32, 128.14, 127.99, 127.07, 125.81, 52.27. The spectral data matched those reported in the literature (Ishiyama et al., 2003).

4-(Methoxycarbonyl)benzoic acid and 3,4-difluorophenylboronic acid (3bh, Figure 4, Entry 60)

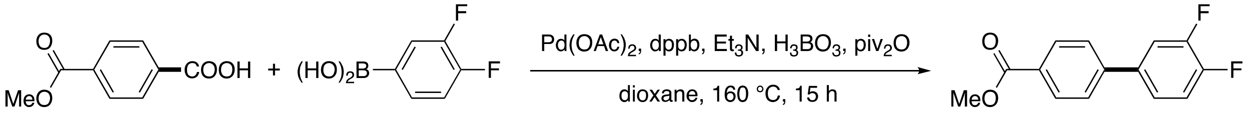

According to the general procedure, the reaction of 4-(methoxycarbonyl)benzoic acid (0.20 mmol), 3,4-difluorophenylboronic acid (2.0 equiv), $Pd(OAc)_2$ (5 mol%), 1,4-bis(diphenylphosphino)butane (10 mol%), triethylamine (1.5 equiv), H_3BO_3 (1.5 equiv) and trimethylacetic anhydride (1.5 equiv) in 1,4-dioxane (0.20 M) for 15 h at 160 °C, afforded after work-up and chromatography the title compound in 83% yield (41.2 mg). White solid. **^{1}H NMR (500 MHz, $CDCl_3$)** δ 8.14-8.12 (d, *J* = 7.5 Hz, 2 H), 7.62-7.61 (d, *J* = 7.4 Hz, 2 H), 7.47-7.43 (t, *J* = 8.6 Hz, 1 H), 7.36 (s, 1 H), 7.30-7.25 (m, 1 H), 3.97 (s, 3 H). **^{13}C NMR (125 MHz, $CDCl_3$)** δ 166.75, 151.53 (dd, J^F = 19.1 Hz), 149.55 (dd, J^F = 20.4 Hz), 143.43, 137.12 (dd, J^F = 5.6 Hz),

130.27, 129.49, 126.90, 123.29 (dd, J^F = 6.2 Hz), 117.78 (d, J^F = 17.3 Hz), 116.26 (d, J^F = 17.8 Hz), 52.22. **^{19}F NMR (471 MHz, $CDCl_3$)** δ -137.01, -138.70. The spectral data matched those reported in the literature (Tang et al., 2015).

4-(Methoxycarbonyl)benzoic acid and benzo[*d*][*1,3*]dioxol-5-ylboronic acid (3bi, Figure 4, Entry 61)

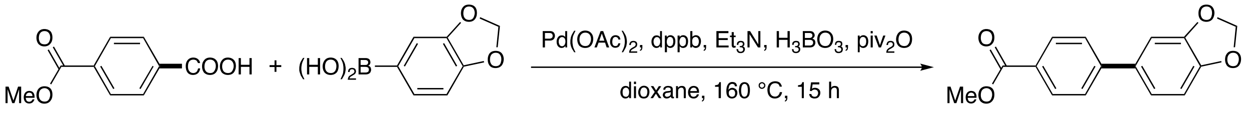

1a 2ac 3bi

According to the general procedure, the reaction of 4-(methoxycarbonyl)benzoic acid (0.20 mmol), benzo[*d*][*1,3*]dioxol-5-ylboronic acid (2.0 equiv), $Pd(OAc)_2$ (5 mol%), 1,4-bis(diphenylphosphino)butane (10 mol%), triethylamine (1.5 equiv), H_3BO_3 (1.5 equiv) and trimethylacetic anhydride (1.5 equiv) in 1,4-dioxane (0.20 M) for 15 h at 160 °C, afforded after work-up and chromatography the title compound in 90% yield (46.2 mg). White solid. **^{1}H NMR (500 MHz, $CDCl_3$)** δ 8.10-8.09 (d, *J* = 7.6 Hz, 2 H), 7.61-7.59 (d, *J* = 7.5 Hz, 2 H), 7.14-7.12 (m, 2 H), 6.93-6.92 (d, *J* = 7.7 Hz, 1 H), 6.04 (s, 2 H), 3.96 (s, 3 H). **^{13}C NMR (125 MHz, $CDCl_3$)** δ 166.99, 148.34, 147.83, 145.30, 134.28, 130.11, 128.54, 126.69, 121.06, 108.72, 107.65, 101.34, 52.10. The spectral data matched those reported in the literature (Wu et al., 2013).

4-(Trifluoromethyl)benzoic acid and 3-chlorophenylboronic acid (3bj, Figure 4, Entry 62)

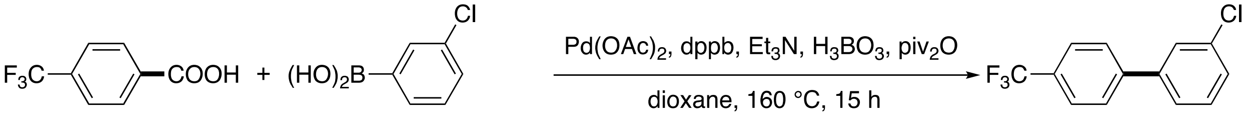

1d 2ad 3bj

According to the general procedure, the reaction of 4-(trifluoromethyl)benzoic acid (0.20 mmol), 3-chlorophenylboronic acid (2.0 equiv), $Pd(OAc)_2$ (5 mol%), 1,4-bis(diphenylphosphino)butane (10 mol%), triethylamine (1.5 equiv), H_3BO_3 (1.5 equiv) and trimethylacetic anhydride (1.5 equiv) in 1,4-dioxane (0.20 M) for 15 h at 160 °C, afforded after work-up and chromatography the title compound in 65% yield (33.4 mg). White solid. **^{1}H NMR (500 MHz, $CDCl_3$)** δ 7.74-7.68 (m, 4 H), 7.61 (s, 1 H), 7.51-7.49 (d, *J* = 7.1 Hz, 1 H), 7.43-7.40 (m, 2 H). **^{13}C NMR (125 MHz, $CDCl_3$)** δ 143.31, 141.58, 134.96, 130.24, 129.99 (q, J^F = 32.4 Hz), 128.23, 127.45,

127.29, 125.88 (q, J^F = 3.6 Hz), 125.45, 124.17 (q, J^F = 270.4 Hz). **<u>^{19}F NMR (471 MHz, CDCl$_3$)</u>** δ -62.50. The spectral data matched those reported in the literature (Sun et al., 2018).

4-(Trifluoromethyl)benzoic acid and 4-chlorophenylboronic acid (3bk, Figure 4, Entry 63)

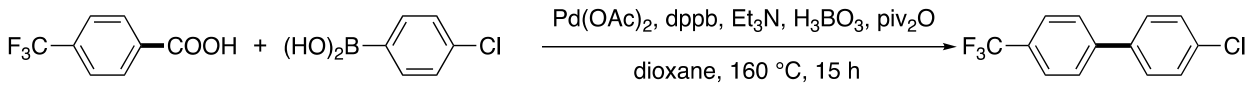

1d **2ae** **3bk**

According to the general procedure, the reaction of 4-(trifluoromethyl)benzoic acid (0.20 mmol), 4-chlorophenylboronic acid (2.0 equiv), Pd(OAc)$_2$ (5 mol%), 1,4-bis(diphenylphosphino)butane (10 mol%), triethylamine (1.5 equiv), H_3BO_3 (1.5 equiv) and trimethylacetic anhydride (1.5 equiv) in 1,4-dioxane (0.20 M) for 15 h at 160 °C, afforded after work-up and chromatography the title compound in 54% yield (27.8 mg). White solid. **<u>^{1}H NMR (500 MHz, CDCl$_3$)</u>** δ 7.73-7.67 (m, 4 H), 7.56-7.55 (d, *J* = 8.5 Hz, 2 H), 7.48-7.46 (d, *J* = 8.6 Hz, 2 H). **<u>^{13}C NMR (125 MHz, CDCl$_3$)</u>** δ 143.50, 138.20, 134.46, 129.20, 128.65 (d, J^F = 32.8 Hz), 128.53, 127.29, 125.86 (d, J^F = 3.5 Hz), 124.20 (d, J^F = 270.3 Hz). **<u>^{19}F NMR (471 MHz, CDCl$_3$)</u>** δ -62.48. The spectral data matched those reported in the literature (Keaveney et al., 2018).

3-Chlorobenzoic acid and *m*-tolylboronic acid (3bl, Figure 4, Entry 64)

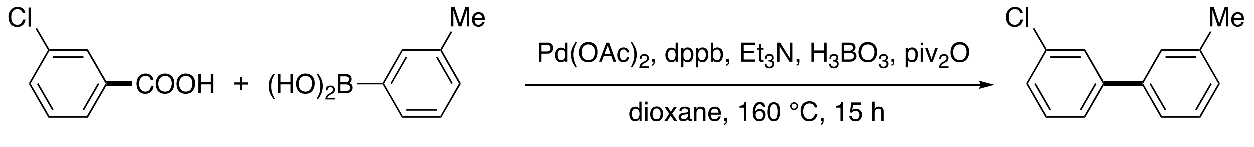

1x **2af** **3bl**

According to the general procedure, the reaction of 3-chlorobenzoic acid (0.20 mmol), *m*-tolylboronic acid (2.0 equiv), Pd(OAc)$_2$ (5 mol%), 1,4-bis(diphenylphosphino)butane (10 mol%), triethylamine (1.5 equiv), H_3BO_3 (1.5 equiv) and trimethylacetic anhydride (1.5 equiv) in 1,4-dioxane (0.20 M) for 15 h at 160 °C, afforded after work-up and chromatography the title compound in 73% yield (29.6 mg). White solid. **<u>^{1}H NMR (500 MHz, CDCl$_3$)</u>** δ 7.60 (s, 1 H), 7.50-7.48 (d, *J* = 7.6 Hz, 1 H), 7.41-7.33 (m, 5 H), 7.23-7.21 (d, *J* = 7.0 Hz, 1 H), 2.45 (s, 3 H). **<u>^{13}C NMR (125 MHz, CDCl$_3$)</u>** δ 143.23, 139.82, 138.55, 134.60, 129.93, 128.81, 128.62, 127.91, 127.31, 127.17, 125.32, 124.23, 21.52. The spectral data matched those reported in the literature (Goossen et al., 2004).

3-Chlorobenzoic acid and 3-fluorophenylboronic acid (3bm, Figure 4, Entry 65)

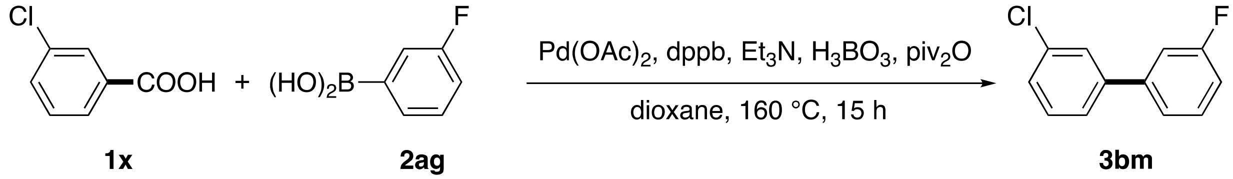

According to the general procedure, the reaction of 3-chlorobenzoic acid (0.20 mmol), 3-fluorophenylboronic acid (2.0 equiv), Pd(OAc)$_2$ (5 mol%), 1,4-bis(diphenylphosphino)butane (10 mol%), triethylamine (1.5 equiv), H_3BO_3 (1.5 equiv) and trimethylacetic anhydride (1.5 equiv) in 1,4-dioxane (0.20 M) for 15 h at 160 °C, afforded after work-up and chromatography the title compound in 77% yield (31.9 mg). White solid. **^{1}H NMR (500 MHz, CDCl$_3$)** δ 7.58 (s, 1 H), 7.48-7.36 (m, 5 H), 7.30-7.28 (m, 1 H), 7.11-7.08 (m, 1 H). **^{13}C NMR (125 MHz, CDCl$_3$)** δ 163.19 (d, J^F = 244.6 Hz), 142.06 (d, J^F = 7.6 Hz), 141.78 (d, J^F = 2.1 Hz), 134.82, 130.41 (d, J^F = 8.4 Hz), 130.12, 127.87, 127.29, 125.27, 122.76 (d, J^F = 2.8 Hz), 114.71 (d, J^F = 21.0 Hz), 114.07 (d, J^F = 22.1 Hz). **^{19}F NMR (471 MHz, CDCl$_3$)** δ -112.71. The spectral data matched those reported in the literature (Sun et al., 2018).

4-(Methoxycarbonyl)benzoic acid and 2,6-dimethoxyphenylboronic acid (3bn, Figure 4, Entry 66)

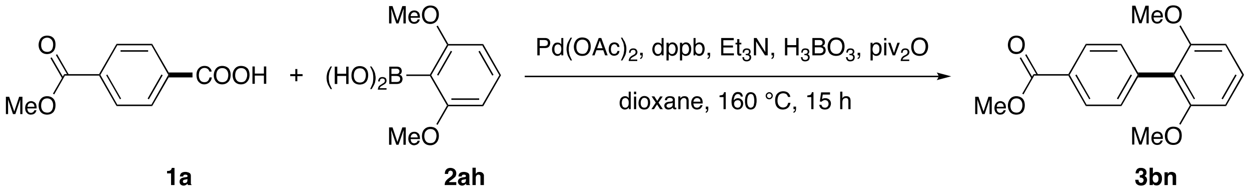

According to the general procedure, the reaction of 4-(methoxycarbonyl)benzoic acid (0.20 mmol), 2,6-dimethoxyphenylboronic acid (2.0 equiv), Pd(OAc)$_2$ (5 mol%), 1,4-bis(diphenylphosphino)butane (10 mol%), triethylamine (1.5 equiv), H_3BO_3 (1.5 equiv) and trimethylacetic anhydride (1.5 equiv) in 1,4-dioxane (0.20 M) for 15 h at 160 °C, afforded after work-up and chromatography the title compound in 90% yield (49.1 mg). White solid. **^{1}H NMR (500 MHz, CDCl$_3$)** δ 8.11-8.09 (d, J = 8.3 Hz, 2 H), 7.46-7.45 (d, J = 8.3 Hz, 2 H), 7.35-7.31 (t, J = 8.4 Hz, 1 H), 6.69-6.68 (d, J = 8.4 Hz, 2 H), 3.95 (s, 3 H), 3.76 (s, 6 H). **^{13}C NMR (125 MHz, CDCl$_3$)** δ 167.27, 157.52, 139.46, 131.11, 129.31, 128.94, 128.38, 118.53, 104.23, 55.91, 52.00. The spectral data matched those reported in the literature (Michelet et al., 2017).

4-Acetylbenzoic acid and 2,6-dimethoxyphenylboronic acid (3bo, Figure 4, Entry 67)

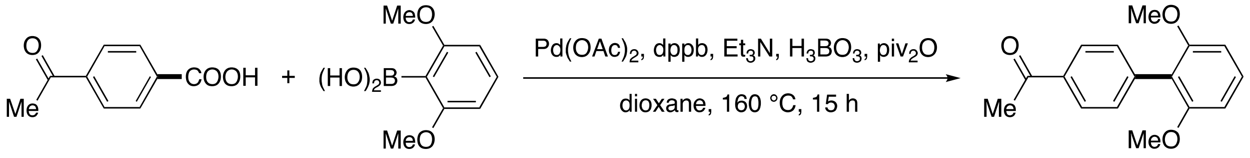

1b **2ah** **3bo**

According to the general procedure, the reaction of 4-acetylbenzoic acid (0.20 mmol), 2,6-dimethoxyphenylboronic acid (2.0 equiv), $Pd(OAc)_2$ (5 mol%), 1,4-bis(diphenylphosphino)butane (10 mol%), triethylamine (1.5 equiv), H_3BO_3 (1.5 equiv) and trimethylacetic anhydride (1.5 equiv) in 1,4-dioxane (0.20 M) for 15 h at 160 °C, afforded after work-up and chromatography the title compound in 90% yield (46.2 mg). White solid. **^{1}H NMR (500 MHz, $CDCl_3$)** δ 8.03-8.02 (d, *J* = 8.2 Hz, 2 H), 7.49-7.48 (d, *J* = 8.1 Hz, 2 H), 7.35-7.32 (t, *J* = 8.4 Hz, 1 H), 6.70-6.68 (d, *J* = 8.4 Hz, 2 H), 3.77 (s, 6 H), 2.65 (s, 3 H). **^{13}C NMR (125 MHz, $CDCl_3$)** δ 198.00, 157.51, 139.75, 135.44, 131.30, 129.40, 127.75, 118.37, 104.22, 55.92, 26.62. The spectral data matched those reported in the literature (Xu et al., 2010).

4-Formylbenzoic acid and 2,6-dimethoxyphenylboronic acid (3bp, Figure 4, Entry 68)

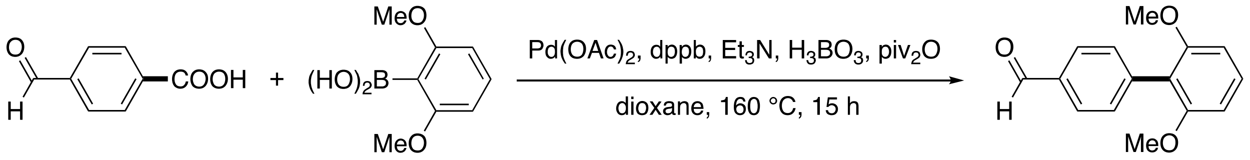

1c **2ah** **3bp**

According to the general procedure, the reaction of 4-formylbenzoic acid (0.20 mmol), 2,6-dimethoxyphenylboronic acid (2.0 equiv), $Pd(OAc)_2$ (5 mol%), 1,4-bis(diphenylphosphino)butane (10 mol%), triethylamine (1.5 equiv), H_3BO_3 (1.5 equiv) and trimethylacetic anhydride (1.5 equiv) in 1,4-dioxane (0.20 M) for 15 h at 160 °C, afforded after work-up and chromatography the title compound in 64% yield (31.0 mg). White solid. **^{1}H NMR (500 MHz, $CDCl_3$)** δ 10.07 (s, 1 H), 7.95-7.93 (d, *J* = 8.0 Hz, 2 H), 7.56-7.55 (d, *J* = 8.0 Hz, 2 H), 7.37-7.33 (t, *J* = 8.4 Hz, 1 H), 6.71-6.69 (d, *J* = 8.4 Hz, 2 H), 3.77 (s, 6 H). **^{13}C NMR (125 MHz, $CDCl_3$)** δ 192.26, 157.46, 141.27, 134.82, 131.81, 129.59, 129.09, 118.19, 104.21, 55.90. The spectral data matched those reported in the literature (Heijnen et al., 2019).

4-(Trifluoromethyl)benzoic acid and 2,6-dimethoxyphenylboronic acid (3bq, Figure 4, Entry 69)

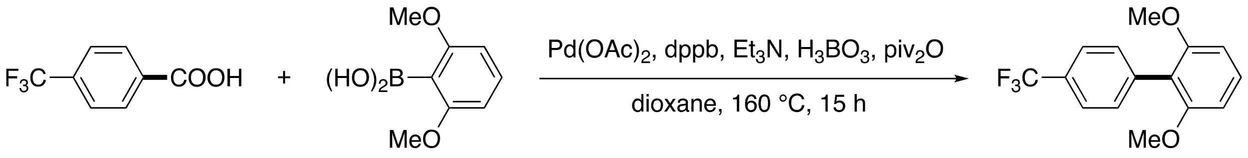

1d **2ah** **3bq**

According to the general procedure, the reaction of 4-(trifluoromethyl)benzoic acid (0.20 mmol), 2,6-dimethoxyphenylboronic acid (2.0 equiv), $Pd(OAc)_2$ (5 mol%), 1,4-bis(diphenylphosphino)butane (10 mol%), triethylamine (1.5 equiv), H_3BO_3 (1.5 equiv) and trimethylacetic anhydride (1.5 equiv) in 1,4-dioxane (0.20 M) for 15 h at 160 °C, afforded after work-up and chromatography the title compound in 85% yield (48.0 mg). White solid. **^{1}H NMR (500 MHz, $CDCl_3$)** δ 7.68-7.66 (d, *J* = 8.1 Hz, 2 H), 7.50-7.48 (d, *J* = 8.0 Hz, 2 H), 7.36-7.33 (t, *J* = 8.4 Hz, 1 H), 6.70-6.69 (d, *J* = 8.4 Hz, 2 H), 3.77 (s, 6 H). **^{13}C NMR (125 MHz, $CDCl_3$)** δ 157.52, 138.10, 131.37, 129.39, 128.67 (q, J^F = 31.9 Hz), 124.55 (q, J^F = 3.8 Hz), 124.48 (q, J^F = 270.2 Hz), 118.09, 104.20, 55.89. **^{19}F NMR (471 MHz, $CDCl_3$)** δ -62.38. The spectral data matched those reported in the literature (Pinxterhuis et al., 2016).

4-Cyanobenzoic acid and 2,6-dimethoxyphenylboronic acid (3br, Figure 4, Entry 70)

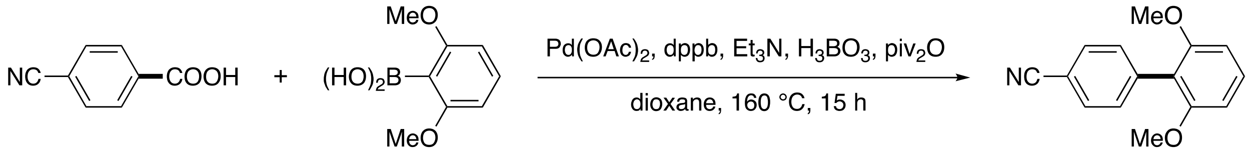

1f **2ah** **3br**

According to the general procedure, the reaction of 4-cyanobenzoic acid (0.20 mmol), 2,6-dimethoxyphenylboronic acid (2.0 equiv), $Pd(OAc)_2$ (5 mol%), 1,4-bis(diphenylphosphino)butane (10 mol%), triethylamine (1.5 equiv), H_3BO_3 (1.5 equiv) and trimethylacetic anhydride (1.5 equiv) in 1,4-dioxane (0.20 M) for 15 h at 160 °C, afforded after work-up and chromatography the title compound in 61% yield (29.2 mg). White solid. **^{1}H NMR (500 MHz, $CDCl_3$)** δ 7.70-7.68 (d, *J* = 8.0 Hz, 2 H), 7.49-7.48 (d, *J* = 8.0 Hz, 2 H), 7.36-7.33 (t, *J* = 8.3 Hz, 1 H), 6.69-6.68 (d, *J* = 8.4 Hz, 2 H), 3.76 (s, 6 H). **^{13}C NMR (125 MHz, $CDCl_3$)** δ 157.33, 139.54, 131.93, 131.38, 129.80, 119.38, 117.55, 110.29, 104.17, 55.87. The spectral data matched those reported in the literature (Liu et al., 2013).

4-(Tosyloxy)benzoic acid and 2,6-dimethoxyphenylboronic acid (3bs, Figure 4, Entry 71)

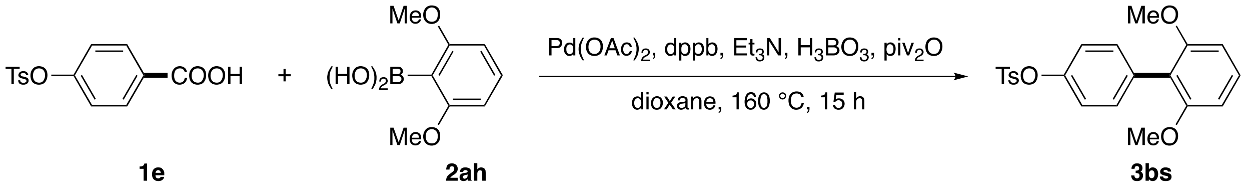

According to the general procedure, the reaction of 4-(tosyloxy)benzoic acid (0.20 mmol), 2,6-dimethoxyphenylboronic acid (2.0 equiv), $Pd(OAc)_2$ (5 mol%), 1,4-bis(diphenylphosphino)butane (10 mol%), triethylamine (1.5 equiv), H_3BO_3 (1.5 equiv) and trimethylacetic anhydride (1.5 equiv) in 1,4-dioxane (0.20 M) for 15 h at 160 °C, afforded after work-up and chromatography the title compound in 78% yield (60.0 mg). *New compound.* White solid. **Mp** = 146-147 °C. **^{1}H NMR (500 MHz, $CDCl_3$)** δ 7.81-7.79 (d, *J* = 8.1 Hz, 2 H), 7.36-7.34 (d, *J* = 8.1 Hz, 2 H), 7.29-7.28 (d, *J* = 8.4 Hz, 3 H), 7.04-7.02 (d, *J* = 8.5 Hz, 2 H), 6.67-6.65 (d, *J* = 8.4 Hz, 2 H), 3.74 (s, 6 H), 2.48 (s, 3 H). **^{13}C NMR (125 MHz, $CDCl_3$)** δ 157.54, 148.29, 145.12, 133.05, 132.89, 132.28, 129.69, 129.03, 128.57, 121.39, 118.17, 104.26, 55.87, 21.72. **HRMS** calcd for $C_{21}H_{20}O_5SNa$ (M^+ + Na) 407.0924, found 407.0925.

Quinoline-6-carboxylic acid and 2,6-dimethoxyphenylboronic acid (3bt, Figure 4, Entry 72)

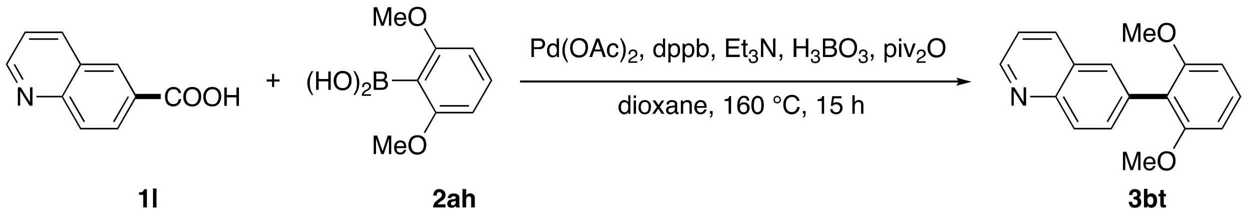

According to the general procedure, the reaction of quinoline-6-carboxylic acid (0.20 mmol), 2,6-dimethoxyphenylboronic acid (2.0 equiv), $Pd(OAc)_2$ (5 mol%), 1,4-bis(diphenylphosphino)butane (10 mol%), triethylamine (1.5 equiv), H_3BO_3 (1.5 equiv) and trimethylacetic anhydride (1.5 equiv) in 1,4-dioxane (0.20 M) for 15 h at 160 °C, afforded after work-up and chromatography the title compound in 75% yield (39.8 mg). *New compound.* White solid. **Mp** = 104-106 °C. **^{1}H NMR (500 MHz, $CDCl_3$)** δ 8.93-8.92 (d, *J* = 2.9 Hz, 1 H), 8.19-8.14 (m, 2 H), 7.84 (s, 1 H), 7.75-7.73 (dd, *J* = 8.7 Hz, 1 H), 7.42-7.34 (m, 2 H), 6.74-6.72 (d, *J* = 8.4 Hz, 2 H), 3.77 (s, 6 H). **^{13}C NMR (125 MHz, $CDCl_3$)** δ 157.77, 150.05, 147.41, 136.27, 133.26, 132.69, 129.59, 129.19, 128.37, 128.10, 120.84, 118.65, 104.29, 55.95. **HRMS** calcd for $C_{17}H_{15}NO_2$ (M^+) 265.1097, found 265.1093.

1-Naphthoic acid and 2,6-dimethoxyphenylboronic acid (3bu, Figure 4, Entry 73)

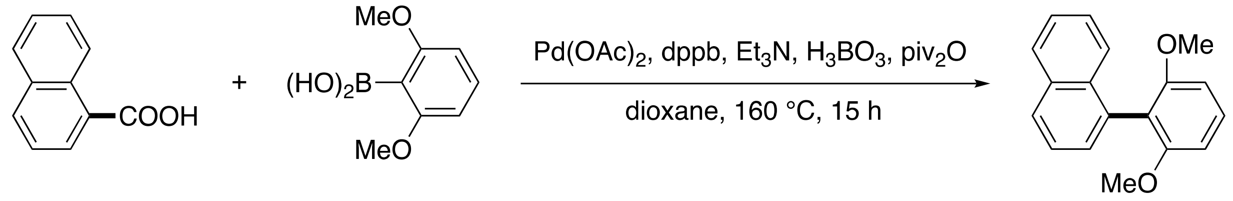

1j **2ah** **3bu**

According to the general procedure, the reaction of 1-naphthoic acid (0.20 mmol), 2,6-dimethoxyphenylboronic acid (2.0 equiv), $Pd(OAc)_2$ (5 mol%), 1,4-bis(diphenylphosphino)butane (10 mol%), triethylamine (1.5 equiv), H_3BO_3 (1.5 equiv) and trimethylacetic anhydride (1.5 equiv) in 1,4-dioxane (0.20 M) for 15 h at 160 °C, afforded after work-up and chromatography the title compound in 41% yield (21.7 mg). White solid. **^{1}H NMR (500 MHz, $CDCl_3$)** δ 7.91-7.87 (m, 2 H), 7.58-7.55 (m, 1 H), 7.50-7.35 (m, 5 H), 6.76-6.74 (d, *J* = 8.4 Hz, 2 H), 3.66 (s, 6 H). **^{13}C NMR (125 MHz, $CDCl_3$)** δ 158.50, 133.57, 132.68, 132.59, 129.11, 128.18, 128.05, 127.45, 126.02, 125.47, 125.42, 125.35, 117.73, 104.21, 55.95. The spectral data matched those reported in the literature (Pinxterhuis et al., 2016).

2-Methylbenzoic acid and 2-methoxyphenylboronic acid (3bv, Figure 4, Entry 74)

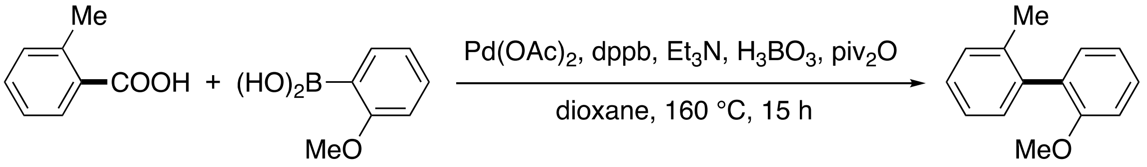

1g **2l** **3bv**

According to the general procedure, the reaction of 2-methylbenzoic acid (0.20 mmol), 2-methoxyphenylboronic acid (2.0 equiv), $Pd(OAc)_2$ (5 mol%), 1,4-bis(diphenylphosphino)butane (10 mol%), triethylamine (1.5 equiv), H_3BO_3 (1.5 equiv) and trimethylacetic anhydride (1.5 equiv) in 1,4-dioxane (0.20 M) for 15 h at 160 °C, afforded after work-up and chromatography the title compound in 31% yield (12.3 mg). White solid. **^{1}H NMR (500 MHz, $CDCl_3$)** δ 7.38-7.35 (m, 1 H), 7.28-7.23 (m, 3 H), 7.21-7.20 (d, *J* = 7.2 Hz, 1 H), 7.18-7.16 (dd, *J* = 7.4 Hz, 1 H), 7.05-7.02 (t, *J* = 7.4 Hz, 1 H), 7.00-6.98 (d, *J* = 8.3 Hz, 1 H), 3.79 (s, 3 H), 2.16 (s, 3 H). **^{13}C NMR (125 MHz, $CDCl_3$)** δ 156.62, 138.64, 136.83, 131.02, 130.88, 130.01, 129.58, 128.55, 127.29, 125.44, 120.45, 110.68, 55.42, 19.91. The spectral data matched those reported in the literature (Teng et al., 2018).

Probenecid and 6-methoxypyridin-3-ylboronic acid (3bw, Figure 4, Entry 75)

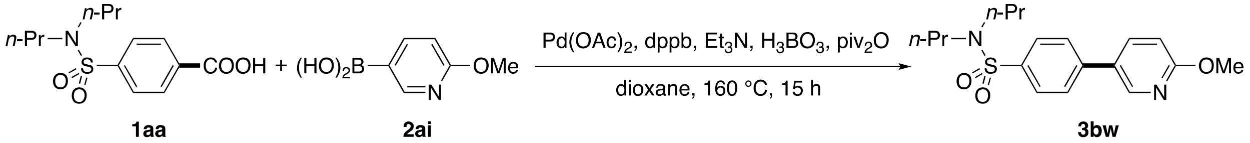

According to the general procedure, the reaction of probenecid (0.20 mmol), 6-methoxypyridin-3-ylboronic acid (2.0 equiv), $Pd(OAc)_2$ (5 mol%), 1,4-bis(diphenylphosphino)butane (10 mol%), triethylamine (1.5 equiv), H_3BO_3 (1.5 equiv) and trimethylacetic anhydride (1.5 equiv) in 1,4-dioxane (0.20 M) for 15 h at 160 °C, afforded after work-up and chromatography the title compound in 74% yield (51.6 mg). *New compound.* White solid. **Mp** = 108-110 °C. **^{1}H NMR (500 MHz, $CDCl_3$)** δ 8.44-8.44 (d, *J* = 1.7 Hz, 1 H), 7.90-7.88 (d, *J* = 8.2 Hz, 2 H), 7.84-7.82 (dd, *J* = 8.6 Hz, 1 H), 7.67-7.65 (d, *J* = 8.2 Hz, 2 H), 6.88-6.87 (d, *J* = 8.6 Hz, 1 H), 4.02 (s, 3 H), 3.15-3.12 (t, *J* = 7.6 Hz, 4 H), 1.65-1.57 (m, 4 H), 0.93-0.90 (t, *J* = 7.3 Hz, 6 H). **^{13}C NMR (125 MHz, $CDCl_3$)** δ 164.29, 145.38, 141.83, 138.90, 137.40, 128.34, 127.81, 126.97, 111.19, 53.71, 50.15, 22.13, 11.22. **HRMS** calcd for $C_{18}H_{24}N_2O_3SNa$ (M^+ + Na) 371.1400, found 371.1376.

Probenecid and 5-acetylthiophen-2-ylboronic acid (3bx, Figure 4, Entry 76)

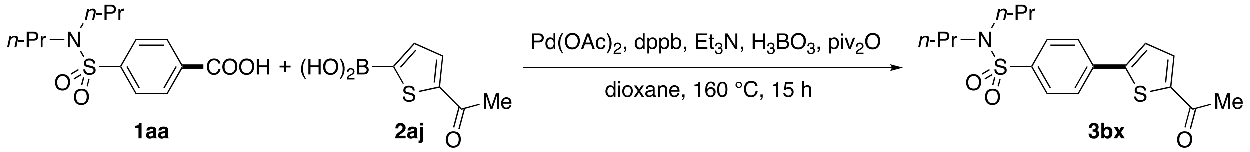

According to the general procedure, the reaction of probenecid (0.20 mmol), 5-acetylthiophen-2-ylboronic acid (2.0 equiv), $Pd(OAc)_2$ (5 mol%), 1,4-bis(diphenylphosphino)butane (10 mol%), triethylamine (1.5 equiv), H_3BO_3 (1.5 equiv) and trimethylacetic anhydride (1.5 equiv) in 1,4-dioxane (0.20 M) for 15 h at 160 °C, afforded after work-up and chromatography the title compound in 93% yield (68.0 mg). *New compound.* White solid. **Mp** = 148-150 °C. **^{1}H NMR (500 MHz, $CDCl_3$)** δ 7.87-7.85 (d, *J* = 8.5 Hz, 2 H), 7.78-7.77 (d, *J* = 8.4 Hz, 2 H), 7.71-7.70 (d, *J* = 3.9 Hz, 1 H), 7.44-7.43 (d, *J* = 3.9 Hz, 1 H), 3.14-3.11 (t, *J* = 7.7 Hz, 4 H), 2.60 (s, 3 H), 1.63-1.55 (m, 4 H), 0.92-0.89 (t, *J* = 7.4 Hz, 6 H). **^{13}C NMR (125 MHz, $CDCl_3$)** δ 190.54, 150.09, 144.61, 140.25, 136.95, 133.36, 127.90, 126.57, 125.40, 50.07, 26.67, 22.06, 11.21. **HRMS** calcd for $C_{18}H_{23}NO_3S_2Na$ (M^+ + Na) 388.1012, found 388.1011.

Probenecid and pyrene-1-boronic acid (3by, Figure 4, Entry 77)

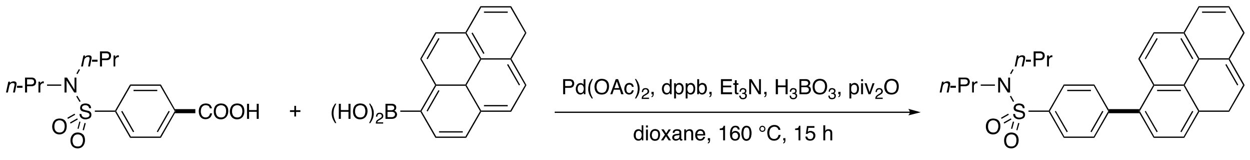

According to the general procedure, the reaction of probenecid (0.20 mmol), pyrene-1-boronic acid (2.0 equiv), $Pd(OAc)_2$ (5 mol%), 1,4-bis(diphenylphosphino)butane (10 mol%), triethylamine (1.5 equiv), H_3BO_3 (1.5 equiv) and trimethylacetic anhydride (1.5 equiv) in 1,4-dioxane (0.20 M) for 15 h at 160 °C, afforded after work-up and chromatography the title compound in 81% yield (71.6 mg). *New compound.* White solid. **Mp** = 363-365 °C. **^{1}H NMR (500 MHz, $CDCl_3$)** δ 8.27-8.25 (m, 2 H), 8.23-8.21 (d, *J* = 7.5 Hz, 1 H), 8.17-8.12 (m, 2 H), 8.09-8.05 (m, 3 H), 8.04-8.02 (d, *J* = 8.2 Hz, 2 H), 7.99-7.97 (d, *J* = 7.9 Hz, 1 H), 7.80-7.78 (d, *J* = 8.2 Hz, 2 H), 3.26-3.23 (t, *J* = 7.6 Hz, 4 H), 1.73-1.66 (m, 4 H), 1.00-0.97 (t, *J* = 7.4 Hz, 6 H). **^{13}C NMR (125 MHz, $CDCl_3$)** δ 145.44, 139.02, 135.68, 131.45, 131.19, 131.12, 130.87, 128.39, 128.11, 127.96, 127.34, 127.18, 126.26, 125.55, 125.20, 124.96, 124.79, 124.72, 124.49, 50.28, 22.25, 11.29. **HRMS** calcd for $C_{28}H_{29}NO_2S$ (M^+) 443.1914, found 443.1898.

Probenecid and 3-cyanophenylboronic acid (3bz, Figure 4, Entry 78)

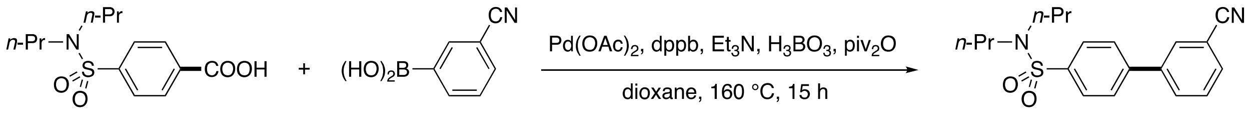

According to the general procedure, the reaction of probenecid (0.20 mmol), 3-cyanophenylboronic acid (2.0 equiv), $Pd(OAc)_2$ (5 mol%), 1,4-bis(diphenylphosphino)butane (10 mol%), triethylamine (1.5 equiv), H_3BO_3 (1.5 equiv) and trimethylacetic anhydride (1.5 equiv) in 1,4-dioxane (0.20 M) for 15 h at 160 °C, afforded after work-up and chromatography the title compound in 98% yield (67.2 mg). *New compound.* Colorless oil. **^{1}H NMR (500 MHz, $CDCl_3$)** δ 7.94-7.91 (t, *J* = 8.4 Hz, 3 H), 7.86-7.85 (d, *J* = 7.9 Hz, 1 H), 7.73-7.70 (t, *J* = 10.0 Hz, 3 H), 7.64-7.61 (t, *J* = 7.8 Hz, 1 H), 3.16-3.13 (t, *J* = 7.6 Hz, 4 H), 1.64-1.57 (m, 4 H), 0.93-0.90 (t, *J* = 7.4 Hz, 6 H). **^{13}C NMR (125 MHz, $CDCl_3$)** δ 142.62, 140.74, 140.15, 131.73, 131.62, 130.87, 129.95, 127.89, 127.67, 118.46, 113.37, 50.08, 22.07, 11.22. **HRMS** calcd for $C_{19}H_{22}N_2O_2SNa$ (M^+ + Na) 365.1294, found 365.1288.

Probenecid and *m*-tolylboronic acid (3ca, Figure 4, Entry 79)

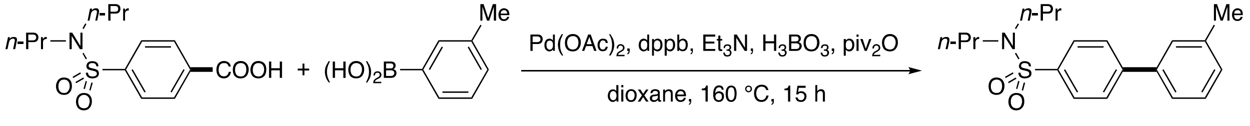

According to the general procedure, the reaction of probenecid (0.20 mmol), *m*-tolylboronic acid (2.0 equiv), $Pd(OAc)_2$ (5 mol%), 1,4-bis(diphenylphosphino)butane (10 mol%), triethylamine (1.5 equiv), H_3BO_3 (1.5 equiv) and trimethylacetic anhydride (1.5 equiv) in 1,4-dioxane (0.20 M) for 15 h at 160 °C, afforded after work-up and chromatography the title compound in 88% yield (58.4 mg). *New compound.* Colorless oil. **^{1}H NMR (500 MHz, $CDCl_3$)** δ 7.89-7.87 (d, *J* = 8.4 Hz, 2 H), 7.72-7.71 (d, *J* = 8.3 Hz, 2 H), 7.44-7.42 (d, *J* = 9.4 Hz, 2 H), 7.40-7.37 (t, *J* = 7.5 Hz, 1 H), 7.26-7.24 (d, *J* = 7.3 Hz, 1 H), 3.14-3.11 (t, *J* = 7.6 Hz, 4 H), 2.60 (s, 3 H), 1.63-1.55 (m, 4 H), 0.92-0.89 (t, *J* = 7.4 Hz, 6 H). **^{13}C NMR (125 MHz, $CDCl_3$)** δ 145.25, 139.41, 138.71, 138.64, 129.13, 128.94, 128.06, 127.58, 127.53, 124.41, 50.12, 22.11, 21.53, 11.23. **HRMS** calcd for $C_{19}H_{25}NO_2SNa$ (M^+ + Na) 354.1498, found 354.1485.

Probenecid and 4-hydroxyphenylboronic acid (3cb, Figure 4, Entry 80)

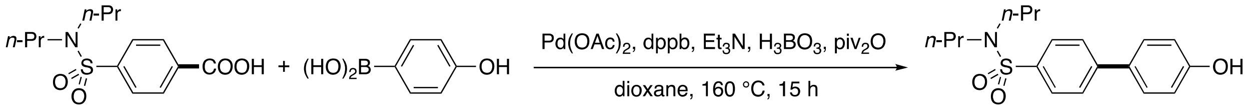

According to the general procedure, the reaction of probenecid (0.20 mmol), 4-hydroxyphenylboronic acid (2.0 equiv), $Pd(OAc)_2$ (5 mol%), 1,4-bis(diphenylphosphino)butane (10 mol%), triethylamine (1.5 equiv), H_3BO_3 (1.5 equiv) and trimethylacetic anhydride (1.5 equiv) in 1,4-dioxane (0.20 M) for 15 h at 160 °C, afforded after work-up and chromatography the title compound in 50% yield (33.4 mg). *New compound.* White solid. **Mp** = 82-84 °C. **^{1}H NMR (500 MHz, $CDCl_3$)** δ 8.35-8.34 (d, *J* = 8.4 Hz, 2 H), 7.98-7.96 (d, *J* = 8.4 Hz, 2 H), 7.49-7.46 (t, *J* = 7.8 Hz, 2 H), 7.25-7.24 (d, *J* = 7.8 Hz, 2 H), 3.17-3.14 (t, *J* = 7.7 Hz, 4 H), 1.63-1.56 (m, 4 H), 0.93-0.90 (t, *J* = 7.4 Hz, 6 H). **^{13}C NMR (125 MHz, $CDCl_3$)** δ 163.90, 144.92, 132.88, 130.81, 129.64, 127.19, 126.28, 121.52, 49.96, 21.96, 11.19. **HRMS** calcd for $C_{18}H_{23}NO_3SK$ (M^+ + K) 372.1036, found 372.1049.

Probenecid and 3,5-difluorophenylboronic acid (3cc, Figure 4, Entry 81)

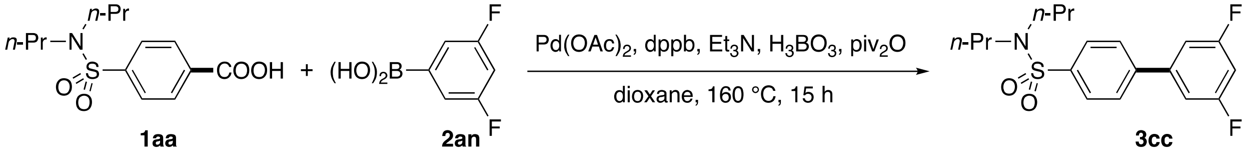

According to the general procedure, the reaction of probenecid (0.20 mmol), 3,5-difluorophenylboronic acid (2.0 equiv), $Pd(OAc)_2$ (5 mol%), 1,4-bis(diphenylphosphino)butane (10 mol%), triethylamine (1.5 equiv), H_3BO_3 (1.5 equiv) and trimethylacetic anhydride (1.5 equiv) in 1,4-dioxane (0.20 M) for 15 h at 160 °C, afforded after work-up and chromatography the title compound in 65% yield (46.0 mg). *New compound.* Colorless oil. **^{1}H NMR (500 MHz, $CDCl_3$)** δ 7.92-7.90 (d, J = 8.4 Hz, 2 H), 7.69-7.67 (d, J = 8.4 Hz, 2 H), 7.15-7.14 (d, J = 6.3 Hz, 2 H), 6.90-6.85 (m, 1 H), 3.15-3.12 (t, J = 7.6 Hz, 4 H), 1.63-1.56 (m, 4 H), 0.93-0.90 (t, J = 7.4 Hz, 6 H). **^{13}C NMR (125 MHz, $CDCl_3$)** δ 163.41 (dd, J^F = 247.6 Hz), 142.60 (d, J^F = 2.1 Hz), 140.16, 132.20, 128.00 (d, J^F = 238.7 Hz), 127.68 (d, J^F = 26.1 Hz), 110.29 (d, J^F = 26.0 Hz), 103.64 (dd, J^F = 25.2 Hz), 50.09, 22.07, 11.20. **^{19}F NMR (471 MHz, $CDCl_3$)** δ -108.81. **HRMS** calcd for $C_{18}H_{21}NO_2SF_2Na$ (M^+ + Na) 376.1153, found 376.1148.

Flufenamic acid and 2-methoxyphenylboronic acid (3cd, Figure 4, Entry 82)

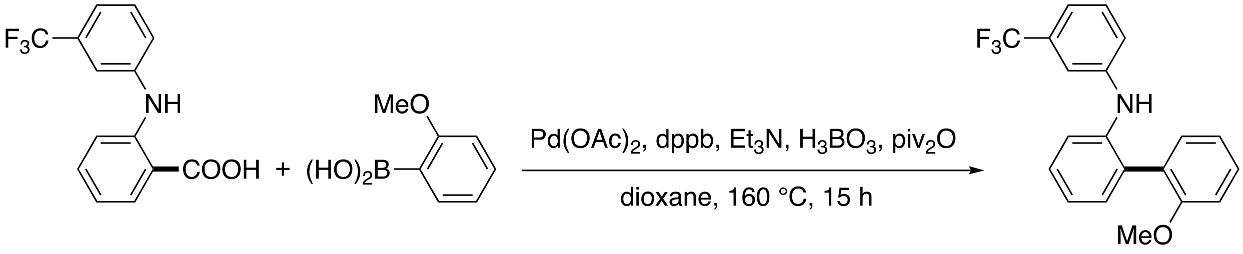

According to the general procedure, the reaction of flufenamic acid (0.20 mmol), 2-methoxyphenylboronic acid (2.0 equiv), $Pd(OAc)_2$ (5 mol%), 1,4-bis(diphenylphosphino)butane (10 mol%), triethylamine (1.5 equiv), H_3BO_3 (1.5 equiv) and trimethylacetic anhydride (1.5 equiv) in 1,4-dioxane (0.20 M) for 15 h at 160 °C, afforded after work-up and chromatography the title compound in 56% yield (38.5 mg). *New compound.* White solid. **Mp** = 135-137 °C. **^{1}H NMR (500 MHz, $CDCl_3$)** δ 7.42-7.39 (t, J = 7.6 Hz, 2 H), 7.38-7.27 (m, 4 H), 7.15-7.06 (m, 5 H), 7.02-7.01 (d, J = 8.2 Hz, 1 H), 5.98 (s, 1 H), 3.74 (s, 3 H). **^{13}C NMR (125 MHz, $CDCl_3$)** δ 156.41, 144.99, 139.95, 131.85, 131.80, 131.57 (q, J^F = 32.4 Hz), 130.81, 129.57, 129.32,

128.32, 128.01, 124.17 (q, J^F = 270.7 Hz), 122.60, 121.37, 119.53, 119.48, 116.21 (q, J^F = 3.7 Hz), 112.94 (q, J^F = 3.6 Hz), 111.16, 55.64. **<u>^{19}F NMR (471 MHz, $CDCl_3$)</u>** δ -62.83. **<u>HRMS</u>** calcd for $C_{20}H_{16}NOF_3$ (M^+) 343.1179, found 343.1165.

Flufenamic acid and 2-fluorophenylboronic acid (3ce, Figure 4, Entry 83)

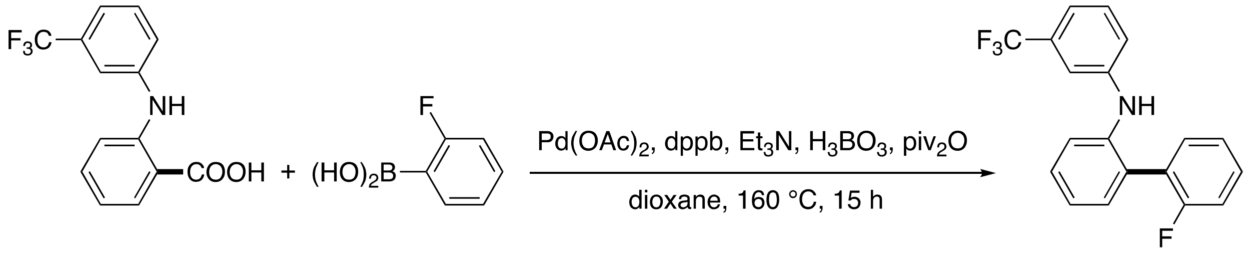

1ab **2j** **3ce**

According to the general procedure, the reaction of flufenamic acid (0.20 mmol), 2-fluorophenylboronic acid (2.0 equiv), $Pd(OAc)_2$ (5 mol%), 1,4-bis(diphenylphosphino)butane (10 mol%), triethylamine (1.5 equiv), H_3BO_3 (1.5 equiv) and trimethylacetic anhydride (1.5 equiv) in 1,4-dioxane (0.20 M) for 15 h at 160 °C, afforded after work-up and chromatography the title compound in 74% yield (49.1 mg). *<u>New compound.</u>* Colorless oil. **<u>^{1}H NMR (500 MHz, $CDCl_3$)</u>** δ 7.42-7.30 (m, 6 H), 7.25-7.11 (m, 6 H), 5.63 (s, 1 H). **<u>^{13}C NMR (125 MHz, $CDCl_3$)</u>** δ 159.87 (d, J^F = 245.0 Hz), 144.31, 139.82, 131.91 (d, J^F = 3.4 Hz), 131.72, 131.67 (q, J^F = 31.9 Hz), 129.79 (d, J^F = 8.1 Hz), 129.70, 129.12, 127.43, 126.21 (d, J^F = 15.9 Hz), 124.66 (d, J^F = 3.5 Hz), 124.08 (q, J^F = 270.8 Hz), 122.61, 120.11, 119.48, 116.94 (q, J^F = 3.7 Hz), 116.05 (d, J^F = 22.2 Hz), 113.63 (q, J^F = 3.8 Hz). **<u>^{19}F NMR (471 MHz, $CDCl_3$)</u>** δ -62.86, -114.15. **<u>HRMS</u>** calcd for $C_{19}H_{13}NF_4$ (M^+) 331.0979, found 331.0995.

Supplemental References

Frisch, M. J. et al. (2010). Gaussian 09, Revision D 01. (Gaussian Inc).

Becke, A. D. (1993). Density-functional thermochemistry. III. The role of exact exchange. J. Chem. Phys. *98*, 5648-5652.

Lee, C., Yang, W., and Parr, R. G. (1988). Development of the Colle-Salvetti correlation-energy formula into a functional of the electron density. Phys. Rev. B: Condens. Matter Mater. Phys. *37*, 785-789.

Hay, P. J., and Wadt, W. R. (1985). *Ab initio* effective core potentials for molecular calculations. Potentials for the transition metal atoms Sc to Hg. J. Chem. Phys. *82*, 270-283.

Wadt, W. R., and Hay, P. J. (1985). *Ab initio* effective core potentials for molecular calculations. Potentials for main group elements Na to Bi. J. Chem. Phys. *82*, 284-298.

Hay, P. J., and Wadt, W. R. (1985). *Ab initio* effective core potentials for molecular calculations. Potentials for K to Au including the outermost core orbitals. J. Chem. Phys. *82*, 299-310.

Zhao, Y., and Truhlar, D. G. (2008). The M06 suite of density functionals for main group thermochemistry, thermochemical kinetics, noncovalent interactions, excited etates, and transition elements: Two new functionals and systematic testing of four M06-class functionals and 12 other functionals. Theor. Chem. Acc. *120*, 215-241.

Zhao, Y., and Truhlar, D. G. (2008). Density functionals with broad applicability in chemistry. Acc. Chem. Res. *41*, 157-167.

von Szentpaly, L., Fuentealba, P., Preuss, H., and Stoll, H. (1982). Pseudopotential calculations on Rb^{+}_{2}, Cs^{+}_{2}, RbH^{+}, CsH^{+} and the mixed alkali dimer ions. Chem. Phys. Lett. *93*, 555-559.

Dolg, M., Wedig, U., Stoll, H., and Preuss, H. (1987). Energy-adjusted ab initio pseudopotentials for the first row transition elements. J. Chem. Phys. *86*, 866-872.

Schwerdtfeger, P., Dolg, M., Schwarz, W. H. E., Bowmaker, G. A., and Boyd, P. D. W. (1989). Relativistic effects in gold chemistry. I. Diatomic gold compounds. J. Chem. Phys. *91*, 1762-

1774.

Marenich, A. V., Cramer, C. J., and Truhlar, D. G. (2009). Universal solvation model based on solute electron density and on a continuum model of the solvent defined by the bulk dielectric constant and atomic surface tensions. J. Phys. Chem. B *113*, 6378-6396.

Legault, C. Y. (2009). CYLview, 1.0b. (Université de Sherbrooke).

Liu, C., Li, G., Shi, S., Meng, G., Lalancette, R., Szostak, R., and Szostak, M. (2018). Acyl and Decarbonylative Suzuki Coupling of N-Acetyl Amides: Electronic Tuning of Twisted, Acyclic Amides in Catalytic Carbon-Nitrogen Bond Cleavage. ACS Catal. *8*, 9131-9139.

Shi, S., Meng, G., and Szostak, M. (2016). Synthesis of Biaryls via Nickel Catalyzed Suzuki-Miyaura Coupling of Amides by Carbon-Nitrogen Cleavage. Angew. Chem. Int. Ed. *55*, 6959-6963.

Kadam, V. D., Feng, B., Chen, X., Liang, W., Zhou, F., Liu, Y., Gao, G., and You, J. (2018). Cascade C-H Annulation Reaction of Benzaldehydes, Anilines, and Alkynes toward Dibenzo[a,f]quinolizinium Salts: Discovery of Photostable Mitochondrial Trackers at the Nanomolar Level. Org. Lett. *20*, 7071-7075.

Lv, L., Zhu, D., Tang, J., Qiu, Z., Li, C. C., Gao, J., and Li, C. J. (2018). Cross-Coupling of Phenol Derivatives with Umpolung Aldehydes Catalyzed by Nickel. ACS Catal. *8*, 4622-4627.

Gan, Y., Wang, G., Xie, X., and Liu, Y. (2018). Nickel-Catalyzed Cyanation of Phenol Derivatives with $Zn(CN)_2$ Involving C-O Bond Cleavage. J. Org. Chem. *83*, 14036-14048.

Simpson, Q., Sinclair, M. J. G., Lupton, D. W., Chaplin, A. B., and Hooper, J. F. (2018). Oxidative Cross-Coupling of Boron and Antimony Nucleophiles via Palladium(I). Org. Lett. *20*, 5537-5540.

Sugahara, T., Murakami, K., Yorimitsu, H., and Osuka, A. (2014). Palladium-Catalyzed Amination of Aryl Sulfides with Anilines. Angew. Chem. Int. Ed. *53*, 9329-9333.

Muto, K., Yamaguchi, J., Musaev, D. G., and Itami, K. (2015). Decarbonylative organoboron cross-coupling of esters by nickel catalysis. Nat. Commun. *6*, 7508.

Okura, K., Teranishi, T., Yoshida, Y., and Shirakawa, E. (2018). Electron-Catalyzed Cross-Coupling of Arylboron Compounds with Aryl Iodides. Angew. Chem. Int. Ed. *57*, 7186-7190.

Nakamura, K., Yasui, K., Tobisu, M., and Chatani, N. (2015). Rhodium-catalyzed cross-coupling of aryl carbamates with arylboron reagents. Tetrahedron *71*, 4484-4489.

Veld, P. H. G., and Laarhoven, W. H. (1978). Substituent effects in the photocyclization of 2-styrylbiphenyls. J. Chem. Soc., Perkin, Trans. 2. 922-927.

Yadav, M. R., Nagaoka, M., Kashihara, M., Zhong, R. L., Miyazaki, T., Sakaki, S., and Nakao, Y. (2017). The Suzuki-Miyaura Coupling of Nitroarenes. J. Am. Chem. Soc. *139*, 9423-9426.

Song, H. J., Jiang, W. T., Zhou, Q. L., Xu, M. Y., and Xiao, B. (2018). Structure-Modified Germatranes for Pd-Catalyzed Biaryl Synthesis. ACS Catal. *8*, 9287-9291.

Keaveney, S. T., Kundu, G., and Schoenebeck, F. (2018). Modular Functionalization of Arenes in a Triply Selective Sequence: Rapid $C(sp^2)$ and $C(sp^3)$ Coupling of C-Br, C-OTf, and C-Cl Bonds Enabled by a Single Palladium(I) Dimer. Angew. Chem. Int. Ed. *57*, 12573-12577.

Liu, W., Liu, P., Lv, L., and Li, C. J. (2018). Metal-Free and Redox-Neutral Conversion of Organotrifluoroborates into Radicals Enabled by Visible Light. Angew. Chem. Int. Ed. *57*, 13499-13503.

Liu, J. B., Yan, H., Chen, H. X., Luo, Y., Weng, J., and Lu, G. (2013). Palladium-catalyzed Suzuki cross-coupling of N'-tosyl arylhydrazines. Chem. Commun. *49*, 5268-5270.

Van Alphen, J. (1931). Primary addition products in indirect substitution in the benzene nucleus. III. Addition products of 4,4'-dialkoxybiphenyls with nitric acid. *Recl. Trav. Chim. Pays-Bas. 50*, 657-658.

Huang, L., and Weix, D. J. (2016). Ruthenium-Catalyzed C-H Arylation of Diverse Aryl Carboxylic Acids with Aryl and Heteroaryl Halides. Org. Lett. *18*, 5432-5435.

Campo, M. A., Zhang, H., Yao, T., Ibdah, A., McCulla, R. D., Huang, Q., Zhao, J., Jenks, W. S., and Larock, R. C. (2007). Aryl to Aryl Palladium Migration in the Heck and Suzuki Coupling of o-Halobiaryls. J. Am. Chem. Soc. *129*, 6298-6307.

Kraszkiewicz, L., Sosnowski, M., and Skulski, L. (2006). Oxidative Iodination of Deactivated Arenes in Concentrated Sulfuric Acid with I_2/$NaIO_4$ and KI/$NaIO_4$ Iodinating Systems. Synthesis 1195-1199.

Barder, T. E., Walker, S. D., Martinelli, J. R., and Buchwald, S. L. (2005). Catalysts for Suzuki-Miyaura Coupling Processes: Scope and Studies of the Effect of Ligand Structure. J. Am. Chem. Soc. *127*, 4685-4696.

Liu, C., Ji, C. L., Hong, X., and Szostak, M. (2018). Palladium-Catalyzed Decarbonylative Borylation of Carboxylic Acids: Tuning Reaction Selectivity by Computation. Angew. Chem. Int. Ed. *57*, 16721-16726.

Crawford, A. G., Liu, Z., Mkhalid, I. A. I., Thibault, M. H., Schwarz, N., Alcaraz, G., Steffen, A., Collings, J. C., Batsanov, A. S., Howard, J. A. K., and Marder, T. B. (2012). Synthesis of 2- and 2,7-Functionalized Pyrene Derivatives: An Application of Selective C-H Borylation. Chem. Eur. J. *18*, 5022-5035.

Ni, J., Li, J., Fan, Z., and Zhang, A. (2016). Cobalt-Catalyzed Carbonylation of C(sp^2)-H Bonds with Azodicarboxylate as the Carbonyl Source. Org. Lett. *18*, 5960-5963.

Miguez, J. M. A., Adrio, L. A., Sousa-Pedrares, A., Vila, J. M., and Hii, K. K. (2007). A Practical and General Synthesis of Unsymmetrical Terphenyls. J. Org. Chem. *72*, 7771-7774.

Adak, L., and Yoshikai, N. (2011). Cobalt-Catalyzed Preparation of Arylindium Reagents from Aryl and Heteroaryl Bromides. J. Org. Chem. *76*, 7563-7568.

Rao, B., Chong, C. C., and Kinjo, R. (2018). Metal-Free Regio- and Chemoselective Hydroboration of Pyridines Catalyzed by 1,3,2-Diazaphosphenium Triflate. J. Am. Chem. Soc. *140*, 652-656.

Wang, D. Y., Wang, C., and Uchiyama, M. (2015). Stannyl-Lithium: A Facile and Efficient Synthesis Facilitating Further Applications. J. Am. Chem. Soc. *137*, 10488-10491.

Roesner, S., and Buchwald, S. L. (2016). Continuous-Flow Synthesis of Biaryls by Negishi Cross-Coupling of Fluoro- and Trifluoromethyl-Substituted (Hetero)arenes. Angew. Chem. Int. Ed. *55*, 10463-10467.

Zhang, Z., Tanaka, K., and Yu, J. Q. (2017). Remote site-selective C-H activation directed by a catalytic bifunctional template. Nature *543*, 538-542.

Kuriyama, M., Matsuo, S., Shinozawa, M., and Onomura, O. (2013). Ether-Imidazolium Carbenes for Suzuki-Miyaura Cross-Coupling of Heteroaryl Chlorides with Aryl/Heteroarylboron Reagents. Org. Lett. *15*, 2716-2719.

Lucas, S., Heim, R., Negri, M., Antes, I., Ries, C., Schewe, K. E., Bisi, A., Gobbi, S., and Hartmann, R. W. (2008). Novel Aldosterone Synthase Inhibitors with Extended Carbocyclic Skeleton by a Combined Ligand-Based and Structure-Based Drug Design Approach. J. Med. Chem. *51*, 6138-6149.

Ramakrishna, V., Rani, M. J., and Reddy, N. D. (2017). A Zwitterionic Palladium(II) Complex as a Precatalyst for Neat-Water-Mediated Cross-Coupling Reactions of Heteroaryl, Benzyl, and Aryl Acid Chlorides with Organoboron Reagents. Eur. J. Org. Chem. 7238-7255.

Kloss, F., Neuwirth, T., Haensch, V. G., and Hertweck, C. (2018). Metal-Free Synthesis of Pharmaceutically Important Biaryls by Photosplicing. Angew. Chem. Int. Ed. *57*, 14476-14481.

Miao, W., and Chan, T. H. (2003). Exploration of Ionic Liquids as Soluble Supports for Organic Synthesis. Demonstration with a Suzuki Coupling Reaction. Org. Lett. *5*, 5003-5005.

Luan, Y. X., Zhang, T., Yao, W. W., Lu, K., Kong, L. Y., Lin, Y. T., and Ye, M. (2017). Amide-Ligand-Controlled Highly para-Selective Arylation of Monosubstituted Simple Arenes with Arylboronic Acids. J. Am. Chem. Soc. *139*, 1786-1789.

Tang, R. J., He, Q., and Yang, L. (2015). Metal-free oxidative decarbonylative coupling of aromatic aldehydes with arenes: direct access to biaryls. Chem. Commun. *51*, 5925-5928.

Zhao, J., Yue, D., Campo, M. A., and Larock, R. C. (2007). An Aryl to Imidoyl Palladium Migration Process Involving Intramolecular C-H Activation. J. Am. Chem. Soc. *129*, 5288-5295.

Ishiyama, T., Nobuta, Y., Hartwig, J. F., and Miyaura, N. (2003). Room temperature borylation of arenes and heteroarenes using stoichiometric amounts of pinacolborane catalyzed by iridium complexes in an inert solvent. Chem. Commun. 2924-2925.

Wu, J., Zhang, D., Chen, L., Li, J., Wang, J., Ning, C., Yu, N., Zhao, F., Chen, D., Chen, X., Chen, K., Jiang, H., Liu, H., and Liu, D. (2013). Discovery and Mechanism Study of SIRT1 Activators that Promote the Deacetylation of Fluorophore-Labeled Substrate J. Med. Chem. *56*, 761-780.

Sun, K. X., He, Q. W., Xu, B. B., Wu, X. T., and Lu, J. M. (2018). Synthesis of N-Heterocyclic Carbene-Pd(II)-2-Methyl-4,5-dihydrooxazole Complexes and Their Application Toward Highly Chemoselective Mono-Suzuki-Miyaura Coupling of Dichlorobenzenes. Asian J. Org. Chem. *7*, 781-787.

Goossen, L. J., and Paetzold, J. (2004). New synthesis of biaryls via Rh-catalyzed decarbonylative Suzuki-coupling of carboxylic anhydrides with arylboroxines. Adv. Synth. Catal. *346*, 1665-1668.

Michelet, B., Deldaele, C., Kajouj, S., Moucheron, C., and Evano, G. (2017). A General Copper Catalyst for Photoredox Transformations of Organic Halides. Org. Lett. *19*, 3576-3579.

Xu, X., Xu, B., Li, Y., and Hong, S. H. (2010). Abnormal N-Heterocyclic Carbene Promoted Suzuki-Miyaura Coupling Reaction: A Comparative Study. Organometallics *29*, 6343-6349.

Heijnen, D., Helbert, H., Luurtsema, G., Elsinga, P. H., and Feringa, B. L. (2019). Synthesis of substituted benzaldehydes via a two-step, one-pot reduction/cross-coupling procedure. Chem. Commun. *21,* 4087-4091.

Pinxterhuis, E. B., Giannerini, M., Hornillos, V., and Feringa, B. L. (2016). Fast, greener and scalable direct coupling of organolithium compounds with no additional solvents. Nat. Commun. *7*, 11698.

Liu, Z., Dong, N., Xu, M., Sun, Z., and Tu, T. (2013). Mild Negishi Cross-Coupling Reactions Catalyzed by Acenaphthoimidazolylidene Palladium Complexes at Low Catalyst Loadings. J. Org. Chem. *78*, 7436-7444.

Teng, Q., Wu, W., Duong, H. A., and Huynh, H. V. (2018). Ring-expanded N-heterocyclic carbenes as ligands in iron-catalysed cross-coupling reactions of arylmagnesium reagents and aryl chlorides. Chem. Commun. *54*, 6044-6047.
